# Antibiotic Resistance Is Associated with Integrative and Conjugative Elements and Genomic Islands in Naturally Circulating *Streptococcus pneumoniae* Isolates from Adults in Liverpool, UK

**DOI:** 10.3390/genes11060625

**Published:** 2020-06-06

**Authors:** Elissavet Nikolaou, Alasdair T. M. Hubbard, João Botelho, Taylor A. M. Marschall, Daniela M. Ferreira, Adam P. Roberts

**Affiliations:** 1 Department of Clinical Sciences, Liverpool School of Tropical Medicine, Liverpool L3 5QA, UK; Elissavet.Nikolaou@lstmed.ac.uk (E.N.); Daniela.Ferreira@lstmed.ac.uk (D.M.F.); 2 Department of Tropical Disease Biology, Liverpool School of Tropical Medicine, Liverpool L3 5QA, UK; Alasdair.Hubbard@lstmed.ac.uk (A.T.M.H.); te.mf.marschall@gmail.com (T.A.M.M.); 3 Centre for Drugs and Diagnostics, Liverpool School of Tropical Medicine, Liverpool L3 5QA, UK; 4 Antibiotic Resistance Evolution Group, Max-Planck-Institute for Evolutionary Biology, 24306 Plön, Germany; botelho@evolbio.mpg.de; 5 Department of Evolutionary Ecology and Genetics, Christian-Albrechts-Universität zu Kiel, 24118 Kiel, Germany

**Keywords:** *Streptococcus pneumoniae*, mobile genetic elements, *tet*(32), Tn*2010*, Tn*6002*, Tn*6822*

## Abstract

Pneumonia is the sixth largest cause of death in the UK. It is usually caused by *Streptococcus pneumoniae*, which healthy individuals can carry in their nose without symptoms of disease. Antimicrobial resistance further increases mortality and morbidity associated with pneumococcal infection, although few studies have analysed resistance in naturally circulating pneumococcal isolates in adult populations. Here, we report on the resistome and associated mobile genetic elements within circulating pneumococcus isolated from adult volunteers enrolled in the experimental human pneumococcal colonisation (EHPC) research program at the Liverpool School of Tropical Medicine, UK. Pneumococcal isolates collected from 30 healthy asymptomatic adults who had volunteered to take part in clinical research were screened for antibiotic susceptibility to erythromycin and tetracycline, and whole-genome sequenced. The genetic context of resistance to one or both antibiotics in four isolates was characterised bioinformatically, and any association of the resistance genes with mobile genetic elements was determined. Tetracycline and macrolide resistance genes [*tet*(M), *erm*(B), *mef*(A), *msr(*D)] were detected on known Tn*916*-like integrative and conjugative elements, namely Tn*6002* and Tn*2010*, and *tet*(32) was found for the first time in *S. pneumoniae* located on a novel 50 kb genomic island. The widespread use of pneumococcal conjugate vaccines impacts on serotype prevalence and transmission within the community. It is therefore important to continue to monitor antimicrobial resistance (AMR) genes present in both vaccine types and non-vaccine types in response to contemporary antimicrobial therapies and characterise the genetic context of acquired resistance genes to continually optimise antibiotic therapies.

## 1. Introduction

Pneumonia is the largest cause of vaccine-preventable death in children under five [1] and a major problem in adults causing high levels of hospitalisations of at risks groups such as the elderly, people with chronic lung disease and asthmatics [2]. The bacterium *Streptococcus pneumoniae*, also known as pneumococcus and the major cause of pneumonia, naturally inhabits the nasopharynx of 40–95% of infants and 10–25% of adults without causing disease [3,4,5]. Pneumococcal carriage (or colonisation) varies amongst individuals, as multiple strains of pneumococcus may inhabit the nose at the same time (co-colonisation) [6] at different densities [7,8], especially in children [9]. Current pneumococcal conjugate vaccines (PCV) only protect against 13 of >90 types of pneumococcus that cause disease [10]. Although PCV vaccination has reduced carriage of vaccine-types (VT), carriage of non-vaccine types (NVT) has increased (serotype replacement). This indicates that the competition of different pneumococcal serotypes within the respiratory niche is a prerequisite for bacterial colonisation [11]. However, serotype prevalence varies in different age groups and geographic regions in each population exposed to antibiotics and pneumococcal vaccination [12]. Antimicrobial resistance (AMR) in *S. pneumoniae* further increases mortality and morbidity associated with infection [13], and its incidence is serotype-dependent [14]. Subsequently, a deeper understanding of circulating AMR genes in *S. pneumoniae* is a prerequisite for developing optimised antibiotic therapies. 

The Experimental Human Pneumococcal Challenge (EHPC) model of infection with pneumococcus is a safe, rapid and accurate tool to study the microbiology of the nasopharynx in humans [15]. Individuals are inoculated with a known dose of live pneumococcus to investigate pneumococcal carriage rates and longitudinally follow carriage episodes. Nasal wash samples are collected before (baseline) and after bacterial inoculation for a month. Using classical microbiology, we have obtained *S. pneumoniae* isolates from naturally colonised volunteers enrolled in the EHPC clinical studies from baseline samples [16]. Here, we analysed 30 of these isolates from this unique pool of pneumococci collected at baseline between 2010 and 2017 in Liverpool, UK, to ascertain any relationship between circulating AMR genes and mobile genetic elements.

## 2. Materials and Methods 

### 2.1. EHPC Clinical Studies

#### 2.1.1. Study Design

The methodology and inclusion/exclusion criteria for EHPC studies have been previously described [17]. Briefly, volunteers included healthy adults aged ≥18 years with no important risk factors for pneumococcal disease, colonisation or transmission (cigarette smoking; close contact with children aged <5 years; healthcare work or caring responsibilities; steroid therapy and respiratory or immunosuppressive comorbidities). Volunteers who received recent antibiotic therapy (within two weeks) and prior pneumococcal vaccination were also excluded. All studies were approved by the local National Health Service Research Ethics Committee (12/NW/0873, 14/NW/0355, 14/NW/1460, 16/NW/0031) and all participants provided written informed consent.

#### 2.1.2. Detection of Pneumococcal Carriage—Baseline Samples

Prior to the study commencing, all volunteers were screened for community-acquired pneumococcal colonisation by nasal wash as previously described [17]. Briefly, 20 mL of 0.9% sodium chloride solution in total (10 mL saline per nostril) was introduced using a syringe and held for a few seconds in volunteer’s nose before being expelled into a sterile container. Collected nasal wash samples were plated on Columbia blood agar supplemented with 5% horse blood (Oxoid, Basingstoke, UK) and 80 μL gentamycin 1 mg/mL (Sigma-Aldrich co Ltd., Dorset, UK) and incubated overnight at 37 °C in 5% CO_2_. *S. pneumoniae* positive samples were serogroup identified by latex agglutination test (Statens Serum Institute, Copenhagen, Denmark).

### 2.2. Susceptibility Testing

Susceptibility towards clarithromycin and doxycycline was determined for all isolates previously [16]. We confirmed susceptibility to erythromycin and tetracycline using concentrations of double the EUCAST breakpoint for resistance for *S. pneumoniae*, according to EUCAST [18]. Erythromycin (Sigma-Aldrich co Ltd., Dorset, UK) was used at 1 µg/mL (EUCAST breakpoint; 0.5 µg/mL), and tetracycline (Sigma-Aldrich co Ltd., Dorset, UK) was used at 4 µg/mL EUCAST breakpoint; 2 µg/mL). To confirm resistance, experiments were repeated three times. 

### 2.3. Filter-Mating Assays 

#### 2.3.1. Streptococcus pneumoniae Isolates

The resistant donor pneumococcal isolates used in this study were recovered from the nose of four different volunteers in Liverpool who participated in the EHPC clinical trials from 2010 to 2017.

The pneumococcal strain FP10 (a derivative of *S. pneumoniae* R6 constructed by Franco Iannelli and Francesco Santoro, University of Siena) was used in this study as a recipient in filter-mating experiments to determine transfer of resistance from the naturally circulating, antibiotic-resistant pneumococcal isolates. FP10’s genetic background is R6, and it is resistant to chloramphenicol (as a chloramphenicol resistance gene was used to knock out *comC*) and streptomycin (due to the presence of point mutations). FP10 lacks *comC* coding for the competence stimulating peptide (CSP), thus is not naturally competent for genetic transformation and has been shown to be a suitable recipient for transfer of mobile genetic elements between *S. pneumoniae* strains [19].

#### 2.3.2. Filter-Mating Procedure

The filter-mating experiments were performed with modifications, as described by [20]. The recipient (FP10) and each of the donors; *S. pneumoniae* 080217, 131016, 210415 and 291015 were grown 16 h on Columbia blood agar plates at 37 °C in 5% CO_2_. Next day, colonies were inoculated into 5 mL fresh Brain Heart Infusion (BHI) broth (Oxoid, Basingstoke, UK) and incubated 16 h at 37 °C in 5% CO_2_. An amount of 5 mL of each cultured donor broth was mixed with 5 mL of cultured recipient broth and harvested by centrifugation at 1503g for 10 m, and the supernatant was discarded. The pelleted cells were gently resuspended in 1 mL of BHI broth, mixed gently but thoroughly, and 100 µL aliquots were spread on 0.45 µm-pore-size sterile 47 mm cellulose nitrate (CN) membrane filters (Sartorius UK Ltd., Surrey, UK), which were previously placed on an antibiotic-free Columbia blood agar plates in five replicates. Plates were incubated 16 h at 37 °C in 5% CO_2_. Each filter was removed from the agar plate and placed in a 50 mL Falcon tube containing 1 mL fresh BHI broth and vortexed for 10 to 20 s [20]. Next, 100 µL aliquots were diluted and spread on Columbia blood agar supplemented with 200 µL antibiotic solution consisting of a final concentration of 10 µg/mL chloramphenicol, 100 µg/mL streptomycin and 4 µg/mL tetracycline in 10 replicates. The plates were incubated 16 h at 37 °C in 5% CO_2_. Next day the number of colonies per filter were counted and re-streaked to fresh Colombia blood agar plates with antibiotics. Plates were left in the incubator for one more night to account for any slow-growing organisms.

### 2.4. Whole Genome Sequencing and Bioinformatic Analysis

Whole-genome sequencing of all 30 pneumococcal isolates was performed by MicrobesNG (MicrobesNG, Birmingham, UK) using 2 × 250 bp paired-end reads on the Illumina MiSeq, which also included the trimming and quality filtering of the sequencing reads which were assembled using SPAdes [21] and annotated in Genbank. Assembly metrics were calculated using QUAST (Table S1).

We used ResFinder, version 3.2 [22] to look for AMR genes, using settings adjusted at 90% ID and 90% coverage cut-offs. The same settings were used to inspect the presence of plasmids in PlasmidFinder, version 2.1 [23]. To identify integrative and conjugative elements (ICEs), we used the standalone versions of ICEfinder [24] and CONJscan [25,26]. Sequences were analysed using PROKKA v.1.14.6 [27,28], and SNAPGENE [29] and annotated manually.

## 3. Results and Discussion

### 3.1. Determination of the Genetic Basis for Resistance

Growth at double the breakpoint in tetracycline or erythromycin was seen in three out of 30 *S. pneumoniae* isolates (291015, 210415 and 080217) and growth in only tetracycline was seen in *S. pneumoniae* 131016. The remaining 26 isolates did not grow at double breakpoint concentrations of antibiotics which agrees with the susceptibility previously reported [16]. 

The analysis of the 30 genome sequences identified resistance genes (Table 1) within the four resistant isolates, matching their resistance phenotype. No other acquired resistance genes were found in any of the isolates to any other antibiotic.

The tetracycline resistance genes *tet(M)* and *tet(32)* both encode ribosomal protection proteins (RPPs). There are currently 13 different genes encoding RPPs reported plus numerous mosaics resulting from recombination between these genes [30,31]. Tetracycline molecules bind to the A-site on the bacterial ribosome, which results in steric hindrance and blocking of the aminoacyl-tRNA binding site [32]. This prevents protein synthesis until the tetracycline molecule is removed. RPPs, such as Tet(M) and Tet(32) bind to the ribosome and causes tetracycline molecules to release. By far the most common RPP encoding gene is *tet(M)*, likely to be due to its association with the broad host range conjugative transposon Tn*916* and the related Tn*916*/Tn*1545* elements [33] (see below). 

Another, more recently identified gene encoding an RPP is *tet*(32) [34]. Originally described in a Clostridium-related human colonic anaerobe in 2001 it was subsequently found to be a mosaic gene with *tet(O)* [35] and designated *tet(*O/32/O*)*, with the full *tet*(32) sequence being reported in 2007 [36]. Subsequently, multiple *tet*(32) genes were shown to be associated with mobile DNA inferred by homology of the flanking regions which showed that they likely shared a common origin and complex evolutionary relationship although no transfer during filter-mating experiments has been observed. *S. pneumoniae* has never been shown to carry *tet(32)* previously.

The three pneumococcal isolates showing resistance to erythromycin all encoded *erm*(B). This gene encodes a methyltransferase which prevents binding of macrolides by methylating the ribosomal target site of the macrolide on the 23S ribosomal subunit [37]. The gene, *erm(B)* is increasingly common in pneumococcus due to a strong selective pressure from the use of macrolides [38] and its association with various mobile genetic elements which are predominantly inserted themselves into the Tn*916*/Tn*1545* family of ICEs [33] (see below). These mobile genetic elements also often contain the additional erythromycin resistance genes that we have detected here in *S. peumoniae* 080217, namely *mef(A)* and *mrs(D)* which mediate macrolide efflux by a two-component efflux pump [39] that is able to extrude 14- and 15-membered macrolides (erythromycin is a 14 membered macrolide) [40].

### 3.2. Characterisation of Resistance Associated ICEs

In *S. pneumoniae*, 291015 and 210515 both *tet*(M) and *erm*(B) are located on the same contig within the respective genomes and, by comparison with other genes and genomes found within Genbank, we show that both *tet*(M) and *erm*(B) are located on a truncated Tn*6002*-like element (accession number; AY898750.1). Tn*6002* is a Tn*916*-like element originally identified from *Streptococcus cristatus* [41] with *erm*(B) inserted within the conjugation module between the Tn*916* orfs; *orf20* and *orf19*. The sequence identity to Tn*6002* ends within *orf6* of Tn*6002*, which is located just downstream of *tet*(M). At this point, the sequence is almost identical (99%) to a putative, un-named, larger (68589 bp) ICE from *S. pneumoniae* strain R34-3225 (accession number; LK020687.1), which also contains the truncated Tn*6002*-like element in which *tet*(M) and *erm*(B) are found in isolates 291015 and 210515 described here. Further analysis of the *tet*(M) and *erm*(B) containing contigs in isolates 291015 and 210515 show that the entire putative ICE from strain R34–3225 is present with an identity of 99%. In *S. pneumoniae* isolate 291015 the putative ICE is located on contig 2 (accession number; JABAHF010000002.1, position 107004–175589) and in *S. pneumoniae* isolate 210415 the putative ICE is located on contig 1 (accession number; JABAHE010000001.1, position 19157–87744). The sequences of putative ICEs from isolates 291015 and 210515 are also provided in Figure S1.

*S. pneumoniae* 080217, which contains *mef*(A), *msr*(D), *erm*(B) and *tet*(M) contains a copy of Tn*2010* which is 99% (26340/26390 bp) identical to Tn*2010* from *S. pneumoniae* strain 05P294 from China [42] (accession number; AB426620.1). This Tn*2010*-like ICE is found in contig 4 (accession number; JABAHD010000004.1, position 49960–76348). The Tn*2010* element is another member of the Tn*916*/Tn*1545* family of ICEs [43] which contains both *erm*(B) and *mef*(A)/*msr*(D) as well as *tet*(M). It has been found in multiple *S. pneumoniae* isolates, most recently reported in isolates from Hacettepe University, Turkey [44]. As the entire ICE was able to be delineated by comparison with the Tn*2010* sequence (accession number; AB426620.1) and the nucleotide sequence was less than 100% identical to Tn*2010*, a new Tn number; Tn*6822* was assigned by The Transposon Registry [45] and the sequence of Tn*6822* deposited with Genbank (accession number; MT489699). Filter-mating experiments, selecting for transconjugants on tetracycline containing agar, with the three donors containing Tn*916*-like elements described above, did not yield transconjugants.

### 3.3. Characterisation of a Novel, Resistance Associated Genomic Island

**Figure 1:**
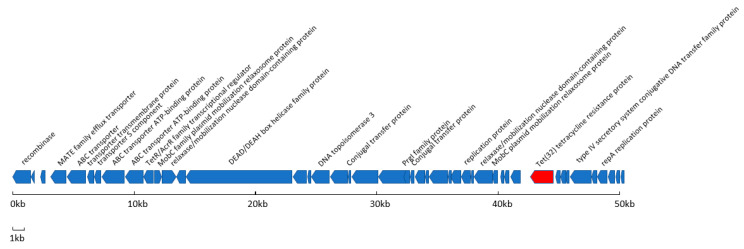
Schematic representation of the genomic island in *S. pneumoniae* 131016. Open Reading Frames (ORFs) which gave BLAST hits to proteins involved in antibiotic resistance, export functions, conjugation and replication have been labelled. The tetracycline resistance gene, *tet*(32), is coloured red.

Isolate 131016, which is tetracycline-resistant and includes *tet*(32), contains a novel 50,977 bp genomic island (GI) flanked by a direct repeat of TCTCAAC (Sequence provided in Figure S1). The GI is found within contig 4 (accession number; JABAHC010000004.1, position 141399-192388). The GI is inserted at the same TCTCAAC heptamer within an aminopeptidase gene which is consequentially insertionally inactivated in the host. The overall architecture of this GI is shown in Figure 1. The analysis of the GI reveals that it has not been reported in its entirety previously although a large region is found in both *Parvimonas micra* (accession number: CP031971.1) and *Streptococcus agalactiae* (accession number: CP022537.1), both of which are oral pathogens. There is a serine recombinase at one end of the GI, which is 99% identical to one found in a recently reported *S. pneumoniae* strain GPSC12 genome (accession number: CAAVIC010000000). Along the length of the GI are genes whose products are predicted to be involved in mobility and replication (Figure 1) suggesting that the GI may have derived from a conjugative plasmid. We could not detect transfer of the GI to the FP10 recipient strain during filter-mating experiments. The TCTCAAC target site heptamer would be expected to occur once every 16,384 bp however it is over-represented in the host *S. pneumoniae*. It is present 25 times in the almost 200kbp contig containing the 50,977 GI. The target sequence does not occur at all within the GI. This equates to an occurrence of one target site every 5921 bp in none GI DNA within the host contig. The fact that it is not present at all in the 50 kb GI and that it is inserted within an ORF is strong evidence that the GI is acquired.

## 4. Conclusions

Our investigation of 30 naturally circulating, non-clinical isolates of *S. pneumoniae* reveals that AMR against tetracyclines and macrolides is likely conferred by a variety of different genes, and these genes are exclusively located on mobile genetic elements. The three tetracycline and erythromycin-resistant isolates contain known Tn*916*-like ICEs circulating within *S. pneumoniae* strains on a global scale whilst the single tetracycline-resistant isolate contains a novel GI which has acquired the tetracycline resistance gene *tet*(32), the first time this gene has been found in *S. pneumoniae*. 

Our study, although only using a limited number of isolates, shows that there is likely to be further, as yet uncharacterised, combinations of AMR genes and associated mobile genetic elements within *S. pneumoniae*. The availability of antibiotic resistance data on local and national scales, in natural circulating *S. pneumoniae* strains, would allow clinicians to make informed prescription choices to avoid high levels of use of antibiotics where a background incidence of potentially mobile resistance exists. These considerations would be in addition to standard antimicrobial susceptibility testing to determine the resistance profile of clinical strains responsible for infection.

## Figures and Tables

**Table 1:** Tetracycline and erythromycin-resistant pneumococcal isolates, antibiotic resistance gene profile and accession numbers.

Bacterial Isolate	Date Isolated	Antibiotic Resistance Genes	GenBank Accession Numbers
*S. pneumoniae* 291015	29 October 2015	*erm*(B), *tet*(M)	JABAHF000000000
*S. pneumoniae* 210415	21 April 2015	*erm*(B), *tet*(M)	JABAHE000000000
*S. pneumoniae* 080217	8 February 2017	*mef*(A), *msr(*D), *erm*(B), *tet*(M)	JABAHD000000000
*S. pneumoniae* 131016	13 October 2016	*tet*(32)	JABAHC000000000

## Supplementary Materials

The following are available online at https://www.mdpi.com/2073-4425/11/6/625/s1, Figure S1: Nucleotide sequences, Table S1: Sequence assembly characteristics.
