# Supplementary material for: Antibiotic Resistance Is Associated with Integrative and Conjugative Elements and Genomic Islands in Naturally Circulating Streptococcus pneumoniae Isolates from Adults in Liverpool, UK
Source: Genes (Basel). 2020 Jun 6;11(6):625. doi: 10.3390/genes11060625 (PMC7348760; doi:10.3390/genes11060625)
Supplement: Supplementary file 1 [file genes-11-00625-s001.pdf]

Supplementary Table 1

Sample ID	Contigs (>=0bp)	Contigs (>=1000bp)	Total length (>=0bp)	Total length (>=1000bp)	Contigs	Largest contig	Total length	GC (%)	N50	N75	L50	L75	N's per 100 kbp
S. pneumoniae 291015	110	39	2114764	2091833	49	309950	2099643	39.49	93700	61735	6	13	0.00
S. pneumoniae 210415	79	44	2067672	2053514	52	194587	2058761	39.54	99827	54391	7	15	0.00
S. pneumoniae 080217	145	40	2107516	2064361	50	288995	2070701	39.66	84901	56642	8	15	0.00
S. pneumoniae 131016	51	16	2032041	2017540	20	390377	2020377	39.76	199003	142854	4	7	0.00

Supplementary figure 1.

A. Nucleotide sequence of *tet*(M) and *erm*(B) containing regions which share 99% sequence identity with the putative ICE from *S. pneumoniae* R34-3225 (accession number; LK020687.1)from *S. pneumoniae* strain 210415 (accession number; JABAHE000000000)

TTAAAAAAGCAGCAAACTATAAACTAAAAAGTTCCACACCAAATGTAACCCCATACTTCCCCATAAGTCAGATTT
ATAGCGCACCATACCTAAAAACATTCCAAGTGAAACGTACAGACACCAAGCTAGAATGGTTCCTGGATGATGTAC
TAAGGCAAATAAAACACTTGTCAAAGCAACTCGAATATCTAATTTTCTAACCAAGTTCCATAAAATTTCACGATA
CAGAAATTCTTCAACCATACTCGCATTGATTAAGAACAATAAAAATGAAAACCAAGGAACTTGATGTTGAAGGCC
AATTAAATTTGTTTGATTCGTGCTTCCTTGAGCATGAATCAGGCTAAAACATAGACTTATAATCAGTAGACTAGC
TAGTCCAATACCAAGGCATTTCATCCTAGTTTTCATATTGACCTTGACCACTTGTTTTCGTTGACCATACATCCA
TAAAAAAGAAAAAAGAGACGCACCATAGAGAACCTGTAGTATAGTTAACTCACCGATACAAAGAAATTTCAATAA
GTATAGAGATACCAATAGGACATTTACTTGTTGGAATATATAAACTGGAATTATTCTTTTCATAGTTACCTCCGA
AATAAATCTTCATAATCTAAATCTAATATCTGCACAATCCTTTCTACCCATGGACTTTGAGGCATTCGTTGTTCC
ATCTTGTAGTGGCGAATCTTTTGATATAAACGATTCAATTCACTTGGATAGTGAAACTCTCCCGCAAACATTTTT
CTGGTTAACTCAATCCAGCTGATATTTCTTTCAGCCAAAATAATGGACAAGTTCTCCCAAAATCGTTCAGCCATA
TTACTTCTCCTTTAGTTAGATAAATAATGTGTTTGTGCCATGTAAATCAATTGTTTCGTATCTCTTGGCAATAGA
GCTCTAGCCTCTTCCAAATTCAGACTTGGATAAACCCGCTTATTTGAAACCACAAAAGGAAGTCCGATGGTTAGT
TCAGGATTTTTTAAAATTATCTCAACGAAATCCGTTAATCTTAGATTGTCACGGTTCTTAAATCGTAATAAATTG
GGAGATAAAAACTCAAAACAATCTGAAGAATAGCTCATCATCTCAATTAATTTGTCCTTTGTCATTTCAGAAACT
GAATGACAAGATACCTCAATGCCATAGTTTTGGAAGAAGTCTAAAAGAAGTTGATTTCTTTGGCTATTTTTACTT
AGATAGAGATCAATCATGGGAGACCTCCAACAAATTTGCTTCCATTTGATATTCTGAGACGATTAAGGAATCTAA
TAAATTTGCGAAGTTAATCGGTTTCTTGTCTTCATCATAAGCTTTTACAGTTACTTGGGTTGTAAGTATTCCCTC
TTTTCCCTCGGCTCGATAGCCTTGTCCATATAAAACAAAAACGAGATTTTGATGATCATCTACAAAGGCATCAAC
CCCATTCTTTATGTCTTGACTTTCAAGGAATTCCATAACGTTTTGAAGATAGGATTCGTAAAATAGTGGGTAATT
ATGTTTTTTATGGTAATCATCTAAAAATGTCACCTCAAACTCACATGGAGAGTAATTTTGACTTTGAACAGCCTA
AAAGTGCCATCAAATTTGAATTGGAATAAATCAAATAAATAGCCCCATCCTCATCAATCCAAGCTTTGCTCAAAG
ACAACTCCAACCGATCTTTTAAAACTGAGTAAACCACCTTAACCTCCAGTTTCATATTCTTATACCGTTCACTCT
CAAATAAAAGTTTGGGGAGCTTATAATAACGCTCTGATGTCTGATATTGACTAGCGGTAATACGCTTCATGATTG
TCCCTCCAAGACTAAAATTCCAACATTTCCAAATTCATCAAATCGGATTAAACCTACTTGTTCCATTTCATCAAC
TAACTGAGTTGCTTTTACAATATCAACTCCCATGATAGTCATGAGATGACTCTTCACGAATTGACGTGATGACTG
TCCTTCCTTTTGCATAATTACCTCCGAAACACAAAAAAAGGGGTAGACAATCTAGTGTCTACCTCCGAAAATTTA
TTAAAAAGAAAAACCCTGCCAAAGAATTTTTGGTAGGGTTTTTGGTAGGAAACTAAATAAATTTATCAGTTTCTA
AAATGTGTTCACGATTCTAAAAGGCTGATACTATAGTATTCCGAATTCTAATTGGTATATGCCTCTTATTTATAA
TAACTATCTCCTCCTTTACACTTTAATTCAAATCTTTATTAAAGAATATTTCATCTTATTTAACAAGAAACCATA
TTTATATAACAACATAAAATACACTAAGTTATTTTATTGAACATATATCGTACTTTATCTATCCGACTATTTGGA
CGACGGGGCTGGCAAACAGGTTCACCGGTAGTAACATGGTACCCTTTTAACTCTGTTAAACAAACACTACGTCCA
TTTGTAAAGAAAGTTAAATCACTACGATATTCTTGAATACACCGAGCAGGGATTTCTCCACTAAGAATGACCTCA
TTATTTTTCAATTGAGTGTCTACGATGTTCGCACAATATTTAGGAGCATCGTTGTATGCTCGTGAAAGATATTCC
TGTGGCGCATAAATTTTAAAACTAAGATATGGCTCTAACAATTCTGTTCCAGCTTTTTTTAAGACTTGTTCCAAT
ACAATAGGAGCAAGCATCCGAAAATCTGCTGGGGTACTAACAGGGCTATAGTATAAGCCATACTTAAAACAGATT
TTACAGTCCGTCACATTCCAACCATACAATCCTTGTTCACAGCCATAGCGTATCCCCTCCATAACTGCATTTTGA
AACGATTGATTTAAGTATCCAAGAGAAACCGAGCTCTCATACTGTACTCCGCTCCCTAATGGAAGCTGTGCTACA
GATAGACCAATGGAAGCCCAGAAAGGATTCGGTGGAACTTCGATGTGAATGGTATACTCTGCTTTTTTTAACGGT
CTTTCCATATAAATGACTGTAGGCTCTTTTATTTCTATCTCCACATGATACTTTTCTTGCAGCAGAGCACAAGTC
ACTTCCATTTGTACTTTCCCTAAGAAAGAAAGTATGATTTCATGTGTCGCAGAATCCACATAATATCGCAGAAGC
GGGTCACTGTCGGAGATTTCTAAAAGTGCATCAAGTAACATTTCCCTTTGTTGAGGTTTGCTCGGTTCAACAGTC
GTTTGCAGCAGAGGGAGGGGATTTTCAATTCTCTCTCTCTGTGGCAATAGCTTTGTATCTCCAAGAACACTATTT
AACTTCAAAAACTCATTCTGCAAAATAACAATTTCCCCGGAATAAGCCTTATCAATTTTACATAATTCACCATTT
ATTGAAGTATACATTTCTGTAATTTTTATTTTTTCCTTTTCCGATATTCTAACCGAATCTCGCAAATGCAGTACG
CCACTATAAAGACGTATATATGCAAGACGCTGTCTTTTTTCCGAATACTCAATTTTGAAAACTTTTCCGCAAAGT
TCAGACTGACCTCGATGTGTTGATGAATAAAATTTATTCGTAATCACTTCTATAAGGTTATCAATCCCTATATTG
TTTTTTGCACTTCCGTGATAAACAGGGAACAGAGAACAATTCTGAAATCTTATGCTTTCCTCTTGTTCGAGTTCC
AATGCTTCTAATGATTTACCGGACATATATTTCTCTAAAAGGTCATCGTTTCCCTCTATTACCGTATCCCATTGT
TCAGATTCGGTAAAGTTCGTCACACACATATTAGGATACAGTTCTACCTTCTGTTTGATTACAATTTCGGCAGAA

AGTTTCTCTTTAATATCCTGATAAACCGTTGATAAATCAATTCCATTTTGGTCAATCTTATTGATAAAAAAGATT
GTGGGAATCCCCATTTTCCTAAGTGCATGAAATAATATACGAGTTTGTGCTTGTACGCCATCTTTTGCAGAAATC
AGTAGAATTGCCCCATCTAAAACTGATAATGAACGATATACTTCTGCTAAGAAATCCATATGTCCTGGCGTGTCT
ATGATGTTCACCTTCGTATTTTCCCACTGAAAAGAGGTTATTCCTGTCTGAATTGTAATTCCTCTCTGACGTTCT
AAAAGCGTATTATCCGTCCTCGTTGTACCTTTGTCCACGCTTCCTAATTCTGTAATCGCTCCACTGTTATATAAT
AAGCTTTCTGTTAAGGTAGTTTTTCCTGCATCAACATGAGCTAAAACTCCAATATTAATAATTTTCATGTGATTT
TCCTCCATTCAAAAACCCAAAAGGGCATAAAAATCCCAGTGATAAATACTTTTATCACTGGGATTTTTATGCATA
ACCATAGGCATACAAAGCATACAGATATTCTCCGGATACTTTAGAATCACATGATAAAGGTATTCTTAAACTGGG
TACAAAAAACTAAGCCCTCCTAAAAAAGGACATCCAATTATTTGTTCCCACTATCAAATTGACAGTTTATTTAAG
AATACCTTGCCGCATATTTATTAACTCCTTTTAAATAGATACTTAAATAATAGCACGTAAGAGCATATTTGTCAA
GGAATCTCCAATTTTTTATCAAAGAGAGTACGTGATTACAAAATAGCTGTAATAATGTACCAATATTTGTTATTC
TATAATCTTCCAATTACTCCCGTTCTTTTCAAGTACCAAATCAAATTGAGATACCTGCGTTGCTTTGGTCTGCTG
GTCGATATACTCCACTGTCAGCGATACCGTGACTTGATTATCCTTACGATTGTGAATAGGATTTACCAGTTCTTG
AAAGATGTACTCTTTTCCGATTGGTTTTAATATCCCGTCATTCACATAGTAGGAAAGTTCACTGGCTGTCGCTGT
AGGATAGAGCTTGAAGAACGTCGTTAAAAACTCATTGATTTCATTGGTTGTAATGGAATCAACCGTCCCCTCACT
TTCAATGGCTTTTGGTTTATAACTTGATTTCTTAGGTATGTTGGTAATGGTCGGATTCTTAACCAGTACCATATT
TCCAGAACCATCTACATAGACACTCACTATATAAGCAGAGTGGACGGTCTTTGTATTTTCTCCCTCTGTAATGAG
CTGGTCTACACTGTAGGTTACATTAAACTCATTGTCGCCAGTTGGCTCTACCGTCCATATCTGAAATCCTCTTAC
AGAAGACGATACAGGAATATCTTTGCGTACTGTATCAACATTGAGAGCTTGAAGTTCATCTGTCAGATAGCCTTT
TAGACTTTCCATTCGATTATCAATGGACTTATCGGATTGCTCCCATGAATAGTAGACTTTCGCAAAGTTCTCTAC
AAAATTTTCTACATGATGAGTATCAACGTATTCCTTTTCTATGATAGTTGTTTCGTGAATAGTATGAGTATCTAT
AGCTGTAAAGTGCTTGAATATCGCAAAGCTGAAACTAAGCCCTAAAAGTACCCACAAGGCAATCACAACCTTTTT
ATGAGGATTGACCTTATAGTAGACACGAGGTTTCTTTTCCTTTGGTATCTGTTTTTCTTTATTCTGATTTTTTCT
AAATTTCATCATTAAATCTTCCTTTCTCATTGTTTGATTCGTCCTGCTCCCACTAAATGCTGTTGCCAGTAGGGG
CTTGTTAAGTCGGCATAACCGATTGGGTCGCCTGCATGAAACATACGGTTATTGCCAAGGTATATCCCAACATGA
GTAATATAAGAGCCAGCGTTATAGGTAGAATGAAAGAAAACCAAATCGCCAGCTTGTGCTTCCGATAGTGGGATA
TGCTGGGTCACATCATATTGCTGTTGTGCGGTTCGTGGTAAGTTAATTCCAGCTTTTCCATACGTCCATTGTGTC
AGTCCGCTACAATCAAAAGAAGTAGTCGGGGAAGCTCCACCGTAAACGTATCGCCAGCCCTCATATTTCAGTGCT
TCGTCCATGATGGCTTGTACCGTATCATCATCAAACTCTGTTGTGACAAGATACTGCGTTACCAGTTGCACATAA
AACATATTGCCATAGTTGTATCGCCAGCCCCCATTGATAGGTATGGCTATGGGATTGGGGTAAGACACTTTTTCG
CCACCTGAATACTCTTTTGAGAAACTTTGAGCCAGTTCAAAGGTATATTTATTTCCACGATTAGCCACATACCCT
AAGAAACCACCACCATAATTGTAGGACTGGATAACCGATTCTAAATCTACACTGAGCCTTTCGCTACTGGCTAAT
AATTCACTGAAATACTTCACACCTTGCTTAATGGATTCTTCTGTACTCAATGAATTAGGTGGAAGACCGAGGGAT
TCCGAGGACTGCATAACATCTTCCGCAGTACCGCCCGATTCCACCTGTATAATCGCAAGAAGTATGTTGACATAT
TCTTCAACGCCATATTCTTTGGCATATTTTTCTACCATAGGCTTATGAGCCAGCACTTCTGCGGAAACATTCACA
CCTCCATAATGAATATTGGAAATTCCGCTGTCCTGTTCATCTGAAAATAAAATGGCAACAAACAGAAGCAGTGAG
AAGACCATCAAGAATAATCCAGAACCACCAATCACTAAAGTTTTCAACTTCATGGTTTCTTACCGACTTTCTTAA
TGGTGGCGGTTTTGATTGGTGGTCTACTTCTTGTATTTTGTAGTGGTACTCTTTGAACAGTAGACGGACGTTCTT
TTGTGATTGGACGTTGTGAAGTTCTATCTGCTGTAGTGGTTGAAGTTGCTGGCTTTTGAACGGTTTTTTCTTGAA
CGGTATTGCCTTGGCGTTCCACTTTTGGACTTGAAAAATCGGACTTAACTGCTGGACGCTCTTGTTTGGCTTGTT
GAGATTCCTTATATGAAGTCTGAATATTAGACTGTTTTGAGGTCTGTTCATCATGATATTGTTCTTGTCTTGTAG
TCGGTCTTTCATGAACAGAAGAAGCAGGCTGTTTTTTCTGTTTGACCTGTTCCATTTCAGAGCGACGCTTCGCAA
TGGTTTTTCGCCTTTGTTCCTGCTGTTCCTTGCGTCCACTGGCTCTGTCCGCTTTGGTTTGAGAAATACTACTGG
TTAAATCACGGACATTCTCTTTTACTTTGGATTTTCCTTGATATACTGCATATCTTGCATTGGTCGGCAAATCTT
TAACCTGTTCTTTCAAACCACTAGCAGTGTCTACCATTCTGTCTTTGGTATCAGCTACTGTACCGATGGTTTGAC
CGATACGTTTTCCAAGTGTTGATTTTTCCTTTCCGTCTGGTCGGGAGTGATCTGCTTGTGTCCTTGCAGAACTCC
CCGAACCCGACTGTCCTTTTTTACCTGTAACAATGGCAGACCCAGCCCCTAGAGTAGTCATGGAACGTCCAAGTT
TCCGCTGTAGACGGTGCATGTGAGCGTGCATAAGCATACGAGGTTTTCTCATCACACGACTTCCCACACTTTGAG
AATCGTTACTCTGTAGAGAAAACATACTCATTAAATCGCCCAGCTTGAAGTAGATTCCTGCAAAGGTCACAATCT
GTAGAAAAGCAATCAAAAAGAACGGATAACCAGCCGATAAGGTATAGAGCATGGTTGAAATACTAAATGCTGTCG
TAATAATCAATGTGATTCCAGCTCGTGTCAAAATGGTATTAAAGAGCTTTGTTATGGCTCGTTTTGACATACCAT
CAAATGATGGAATCATGCTTAAAATAAAGCTCACAGGCAGAAACATAGCATAGATGATAAAAAGTACCTGCGAGA
AAATCATGATTCCTGTTAATAGGAATACAAATATGGAAATCCCAATATTGAAGACAAATAGGAAGAAGACTGTAC
CTAAACGGTTAATGGTCTTTGTAATGGTTAGATTGGTATTGCTTCTGTCTTCAATTTCTTCCGCAACAATTTTTT
CTCTGTCTTCGCCATTGTTGGAATCTGGGCTGGTGGAGAGCAGGCTTTCCACACGGTCAATACCGATACTTTCAA
TGTCTGAACTGTTGTATTGAAGCAGTAGCCACGGTTGCTGAACCTGTATGGAAAACAGGCTATCTCTGATTAAGT
CCACGCTGTCCTTGCCTTGACTATCGGAATGGGGCATGACAATCTTCGTGCCAAGTGATAAACTGGCATTACTGA

TGTCTGATGAAAAGTCATTGATTTTTTTAATGTAGTCGGGAGCGTAGGCAATAAAGGAAGCCGATAGGATAAACA
CCAGCACAAAATTCATAATGGCATGAATTGCCTTTGTGGTTTCTCTCTTTATCAGTCCCGTATAGGCAACATAAA
CCCCAAGAACCAAAATCAAGAGTAAGAGGAATCCAACATAGAAACCCTCTGTTGAAAATCCGTTTGCACTCACAC
CAGCTAAGGTCTGCATATTCTTACCAATGGAATCTGCTGTAGCGGAAATGAAGTCTAAGGAATAGGCTTCCTGTA
CTAAGTAACCTGTCGCATTGGAAACATACAAACTGATTGTCCAAATAAAATTGGTAATGGCATATAGTCCATACA
TGACCTGTTTTCCAATCCCGTCCGACCAGTTCCACGGAAGCCAGCCCCAGCTATTATCCACATAAAAATCCAGTT
GATAGTTTTCAAGTGGGTATCGGCTGTATTCATTTGCCACATTGACCGTATCATCTACCAAGCCCGCAGCTTGAA
CCACCGTTCCCAGCATGGCTAAAAGAAAAATGGCAATCACAAGTGTGAAAGCCACTGTCATTGCCACTTTACCTA
GACGTTTCAGCGTCCAGTTTGATTTTATTCTGTTTACTATTGATGGTTTCACATTTACACCTCTTTTCGCACAGG
TGGTCTGGTATCAAAGGCATGGAGCAGTTCTTCAAATACAGGGTGGAACTGTATCACACCGACACGACCATATAA
ATCACTGATAAGGCATTGCCCGTTTTCCAAATCACGCAATCGCTTCTGATTGTTTTCGTCCTCTGGGTCTACACC
AAAAAAGGCTAAGGTCTTTTTAATCTCGTTAAGGTCAGTGGAACGAAATGCAAATTTTAAGCCGAGGTTATTTTT
CAGTTTTTCATCTAAGAGGTCGTCTGTATTTTGGGTCACGAAATATACCCCAGCGTTCATAGCACGACCAGCCCG
AACCAGCTTCATAGATAGTGTTTTTCCTTGTGCTACCTGTAAAAAGCTCCATGCTTCGTCTAAATCTACAATCTT
GAAAATGCTTCGGTCTGTATGGATAAAGTCTAAAGCAAAGGTACTAATGACAATCAGCATAGCAACGGATAAAAG
CTCCATAGTGGTATATTCCTCAAAGGAAGTTTCCTTGTCGGGAAGTACCAAGTCCGCAACCTGTATAATGTTCAG
TTGTTTTTCTAAGCTGATAGACTGCTCCACATAACCATTACTGAATAATAAATGTGCAAAGTCATAGTCTGTAAA
ACTTTCGATATGGTCGGCTATACTGGTACTTAGTGGCGTATTCTCAACCCGTAATTCCTCAATCACTTTCATCAA
CCCTCGTACTTCACTATTGGTTACTGCACGAATGGCTTTTCTAAGGATTGGGAAGCGTTCCCCATCACGAGAGGA
AATCCCCGTAAGGAATGTCAGAATATCAATAGCCAGTGATTCAGAATCTTTGGGATTTTTCATAATCACATAAGG
GTCAAGTAAGCCTTTGTTTTTCTCATCAGAAGTCAGAGTGACGATATTGATTTCATGGGAAATCTCTGGCAAGGT
TTCTTTCCATCTGCCACGTTCTGCTTTTGGGTCTACAATCACTGCTTGTGCCCCATAAAGCACCGCATAATAGAC
GATAAGGTTATTCGCAAAGGATTTACCACCACCCAGCGAACCAACAAAAGCCGACGCTAACGCATTGGTTACTGA
ACCCTTAACCCCTTGACTGGCAAGAGCAGGTTTCAGATAGACATTGCGTCCAGTATCTAAGCTGTAGCCAACATA
AATCCCCTCATTTTCCCCCAGCATTTGAGTAGCACCAAAACCTAAACCAGCGAGGAAATCAGAGGTCACGTATTG
AATATAATCATTCATATAACGCTTGCTGGCAGGTAAAAATTCTTCATGTAAGCCGAGCATATCCCCAAATGGTCG
TACCAGTTTTACGCTTAAATCGTCATAAAAATCTTTCACTTCATTACAACGACGTTTGAGTTCGTCAAGATCATT
TGCTGATACCCTTACCACATAAGACAGCTTGTACATAGATTCCTTGCTTTGGTCTAAATTGGTTTCCAGCTCATT
CACACTTTCCAGAGCTTCCGCCACATTGGAGCTGGTTTCATTATCACTTTGCCAAGCGTGGTTATCCAAGTCTTT
CAGTTCTTTCTTTTTATTGCGGACAGTAGATAGGGCTTTACGATTCGCTACAATTTCCACATTCATTGACGTATC
AATCGGGAATGTAAATTGCTGTTGCTGGTAGTAGAAGATTTCAGAGGACGGGAAGTCCAGTTCTCCGACAATGCT
GTTAATGGTAAAGTAAGCTACATAGACGGTTTCATCTTCCTGCTGGATTTTCAAATATCGCTGTTTTTCTTCCAC
CAAACAGCGAGTAGGCTTAATCAAGTCATAGTATTTAATCAGCGTTTCATTATCCAGCTTTTTCTTTGATAGATG
GTACTCATACTCTTCATAGGCAGTGCCTGTCTGTCCGTAAAGGTGTTCAATCAGATAGCCGAAGTCGTCCTTATC
TAACCTGCGGATTTTGAAACGACGAGAGATTTTATTTTCTAAGAGCTTTTCCATCTTCTGAAAACGCAGGATTTC
ATCATTACTCATACTAACAAAATCGCCCATCAGCTTATGGTTCACATCATAGACAAAATCAGACAAAGCATTTTT
TGCTTCAACGGTAAGACTTTTCATAGAAAACTCCTGATCGTTGAGAAGCAACTTAAAGCCGATAAAGAAACGGTA
GTTCACTTGATTTTCGCCAATCATGGATATTAAAGCGTCTGTCTGTTGGTCGATTTTGTCATAGGCAACCGCTTT
GAGCTTGCCAGTGACTTCATTTTTGGAACGCTCTTGTGCAGAACGTATGCTGGATTCTGTACTGATTTGTAAAGC
ATGAATTTTGCCATCACGATTTTGTGCGATAAGCTGTCTGAAAGAATCATGCACTTGTATTTTCTGTTCTGGACT
TAGAAATGAGTAATTGTAAGGAACAAGCTCATAGTAAGCATAACATTCCCCGTCTTTATTCCAGACGAGATTGTT
TTCAATGTATTTAATTGGATATGCCATAAAATTCACTCCTAACTGCTGTAATGGCTTCTTGTGGCTGGTTTCTGC
CAAGCGTTACTTTTTTTCCTGCATAGGTCAGCTTTGGTCGCAGTGCATAAGCAATGACAGACTTCAAAAATCCAT
AAGGCTTTTTACCATCAAAAGTTTTTGTAGACATAAACCATGTGAAAGCCACAGGAATCCCAAAGTATTTGAGAA
ATGCTCCCTCTATCATGGAAAGAGGGGGCAAGTTGCCAAGTATCATCACTGCAAAGAGTGACACGACAAACCATG
TCATTTGCGTAAAGGTTATGGGAAACGGAAGTCTAAAATCATTGATAGAATACAGTACCTTTTCCACAGACCAGA
TACTGGTATAGCTTCGTATTTTCTTCATGTAATCAATCCTTTCATAAAAAAATAGGGGTAGCTGATTGAGCCACCC
CGTAAAATAGAAAATCTGCCAGTAGTAATGTACCGACAGATTTAATAGACGATTTCAAAAATCCCATGATTGGTT
GAGATAAACGTTCCTGAAAGGTCTAAATCCCGACCATAGGCTTGATAATCAATATAGTTTTGAAGACTAGCTGGT
ACTTCGCCTAAAGCACCCGTTTCTTCAATGTAGTAGCGTGCCACGTCATACATATCATCACAATCGGAATGAATG
ATAATATCCTCTTGATGTTCGCTTAGTTCTTCAATGCTTGAAAAATGAGTGAGCAGAGCAGATAGCTCCGATTGT
AATTCTTCGGGTAATTCCGATACCATTTCCCATAGTCGATTGAGTTCGCCAATGGAAGTGTATTCGTCAACCGTA
AAGGGTAACTCGTAGTCATGAATGGCGTATTCCTCATATTCATCATTCAAGCCGATTTTCTCTTTGACTTCCTCA
AAGTCAATGGGAAAGGTAAACCACGCACCGACCAATTCGCCCTCATTGTATTTGCCTAAATTCGCAATATAGACT
TGCATATCGTCCATATATTCACGTCCTTTCTTTGTAGAGATTCAAAAATCCCTACCGCACTTCGTTTGGTGTACC
ATTCCTTTGCGGAACATAAGAAAACCACTTATATTCCACAAAAGAACGGTTTTATTTAAGCACCAATAATGCGAT
TGAATAGCTCTAGTAAAATGTCTTTTACTCCAGCAGCGTTGAAGACTAAGCCAACCGCAATAATCGCAATAATTA

AAAAGCCAATCAGTTTGCTAAACTCACGCTTGAAGCCAAGATACAAGCCAATCACAACGATTGCTAAAAGCACCA
GTGATTGAGCGTTTGATAGAAACCAGTTATAAAGGTTTTGTCCAAAATTCATAAAAATGTTCTCCTCTCTATATT
CAATGAATTTGTATTTGAGTTATTTTTTTGGGGTATACACTTTCTGGTATGGATAGAGATTCCATGCCAGATTTT
TTTATACAAAAAAGAGGACATTTGCTGTCCCCTCGTTATACAATCAATTCACCACAAAAATCATGAAAGAAGGTT
AAACAAATGTCCCATAATGATTCTATCCTAAATATTCTTGGAATTAAAGATAAAAATATTAAAATTATTTCTGTT
GAAGAAGCTGAACACAACAACGATTCTGTTAAAGAGTATATAACGCTAATAACAGCTACTCTTTCTTATCCGATT
AATCGTTGTCGTAACTGTGGCTTTCCCACAGTTAATAAGGATGGCTTTCGCAAAACTCATGTACGACTGGCAAGT
TTAAATGGGAGAAGATATGAACTAGAGCTTCGTAAACAACGCTATAAATGTAAATCATGCCATACTACTTTTGGT
GCTATTACTAATTTAACCAAAGAAAATCAAACCTTATCCAGTGATCTCAAAAATCAAATCATGCTTTTAGCTCGT
AAAGGCTTATCTGGTCAGCTTATTGCTGAAATGTGTCACTGCTCTCCTAGCAGTGTTCGTCGAACAATCTTAGAG
CGCATGGAACCACACTATCGTGTGGCTAAGTTGCCTAAGCATCTATGTTTTGACGAGTTTCGTTCAATTAAGTCT
GTGATGTCCTTTATCTGTTGTGACGCTGAAACCCACCAAATTGTCACAAAGTTACAGGATCGTCTATCACCTACC
ATTGTTGATTATTTTGAAAGTCGTTATTCAAAAGCCGAACGCGAATGCGTTCAATCAGTTGTAATTGATTTAAAT
GCTCAATATCAAAGTTTTATCTATCGCCTTTTCCCTAATGCCAATATCATTATTGATCGCTTCCACCTTGTACAA
TTAGCTGGTCGCGCTTTGGACAATTGTCGTATCTCTATCCTAAAGCAACTTGATAAACAGAGCCAAGAATATAAA
ATTATGAAGTCACATTGGAAGCTATTCCATAAAAAAGCTGAAGATCTTCACCCTGAAGAAGTAGTTTTTCTTCGC
GGCGTTAAACAATATATGACTCGCCAAAATGCTGTTGATCTCATTACTAGTAAATTTTCCAAGTTCGCTGAAGTA
TACCAAACTTACCAAGATATCACGAAAGCCCTAAACGAGCGCAATAGTGAATTACTAGAGTCAACCATCTTAGAC
TACCAAAAAACCAATACAGAAATGGATACTGCTATTCAAACCCTTCGTCAAAACAGAAAATATGTCTTAAATAGC
GCTAAATTTGAATACTCTAATGGTCCTTTAGAAGGCATCAATCGCAAAATCAAAACCCTAAAACGAACTTGTTAT
GGTTTTGCCAATCAAAAATTTTTCTTTTTAAGAATCGATTGTATTTTTTCGTAAAAAAATACCCCCTACATTTTC
GTAGGAAGTATTTTTAGTCAACCATACCAGTTGACAGATATCCCACTTAGGACATTTTCCTACAAGGGGTCCCGA
GCGCTTAGTGGGAATTTGTACCCCTTATCGATACAAATTCCCCGTAGGCGCTAGGGACCTCTTTAGCTTCTTGGA
AGCTGTCAGTAGTATATCTAATAATTTATCTCCATTCCCTTTAGTAACGTGTAACTTTCCAAATTTAAAAAAGCG
ACTCATAGAATTATTTCCTCCCGTTAAATAATAGATAACTATTAAAAATAGACAATACTTGCTCATAAGTAATGG
TACTTAAATTGTTTACTTTGGCGTGTTTCATTGCTTGATGAAACTGATTTTTAGTAAACAGTTGACGATATTCTC
GATTGACCCATTTTGAAACAAAGTACGTATATAGCTTCCAATATTTATCTGGAACATCTGTGGTATGGCGGGTAA
GTTTTATTAAGACACTGTTTACTTTTGGTTTAGGATGAAAGCATTCCGCTGGCAGCTTAAGCAATTGCTGAATCG
AGACTTGAGTGTGCAAGAGCAACCCTAGTGTTCGGTGAATATCCAAGGTACGCTTGTAGAATCCTTCTTCAACAA
TCAGATAGATGTCAGACGCACGGCTTTCAAAAACCACTTTTTTAATAATTTGTGTGCTTAAATGGTAAGGAATAT
TCCCAACAATTTTATACCTCTGTTTGTTAGGGAATTGAAACTGTAGAATATCTTGGTGAATTAAAGTGACACGAG
TATTCAGTTTTAATTTTTCTGACGATAAGTTGAATAGATGACTGTCTAATTCAATAGACGTTACCTGTTTACTTA
TTTTAGCCAGTTTCGTCGTTAAATGCCCTTTACCTGTTCCAATTTCGTAAACGGTATCGGTTTCTTTTAAATTCA
ATTGTTTTATTATTTGGTTGAGTACTTTTTCACTCGTTAAAAAGTTTTGAGAATATTTTATATTTTTGTTCATGT
AATCTCTCCTGAAGTGATTACATCTATAAACAAATACAGAAGTTAAACGATTTGTTTGTAATTTTAGTTATCTGT
TTAAAAAGTCATAAGATTAGTCACTGGTAGGAATTAATCTAACGTATTTATTTATCTGCGTAATCACTGTTTTTA
GTCTGTTTCAAAACAGTAGATGTTTTATCTACATTACGCATTTGGAATACCAACATGACGAATCCCTCCTTCTTA
ATTACAAATTTTTAGCATCTAATTTAACTTCAATTCCTATTATACAAAATTTTAAGATAATGCACTATCAACACA
CTCTTAAGTTTGCTTCTAAGTCTTATTTCCATAACTTCTTTTACGTTTCCGCCATTCTTTGCTGTTTCGATTTTT
ATGATATGGTGCAAGTCAGCACGAACACGAACCGTCTTATCTCCCATTATATCTTTTTTTGGATATCTGTCAACT
GGTATGGTTGACAGATATCCTTTTTTTGTTGTTATCACGTCCTGTTCTTTTACTGACTGTTGCTTCAAAATCTGC
TTGTGTCGGTCTGTCAGTTTCGCATGGTCGAGAATGTCTTTTACAACCTGCGTCTGGTTGATTTCATCAAGTTTA
ATCGCAACCTTTAAGGTCGGGGCAACTTGATGAGATAGCCAGTTCAGCGTCCTTTGGAAGGAGTAAGGCTCTGGT
TTTGTGGTTAGTTTTAATCGTTCACGATTGTTCCCAATAAACCAAGCCCATTCTTCATTCAGTTTCCAATCAGAA
CGAGGTTTGGAATCGTCTTTATCTACAAAACGGATATACCGATTGATAATTTTAAAGGCGGTATGCTCTGGATTG
TCATAGACGAGTAAATCACGGACTGCATAATAGGCACGCTCATTTTTCAATCGAATCTCAAAACGGTTTTTTACT
TCTGCGTCTTCAATGGGAATATCATTTTTCTTGTACTGCTCGTAGTCCTTTTCATAGATACAGAAATAAACTTCA
CTTTGTAATGAACCGATATAGAGGGTGTTTCCCATACATTCCTTTTCCTCTTTGCGTACCAGTTCGCCACTGCGA
TAGCTTTTAAAACTGCGGAAGACGGAGATACATTCTTCCTGTTGGCACTTTTCAGTGAGTACAGGGATATTCAAA
ATCCCTGTCTTATCGTTAATGGCAAGGTCAAGGCGTTTCATCACACCGCCAGCCACCAAAACGTCCATAAAGAAC
TCATACCAGCTTCTTTGTTGTGCCAGAAGATAGCTTTCAAATTGTCTGCACCCACGACCTTTCAATTCCACCAGA
ACTCCTTTGTCCAGTTCATGGGAGCAAAGGACGAATATGTCGCCTAAAGCATAATGCTCTGAATAAGAATAGAAA
CCATAGTCCTCATGAAGAAAATAGGACAGTTTCAGTTGTAAGATGTTTTCGACCACCTGCTGTACGTCTGTTGTC
GGAAAGCGAATCCTTACATAATCAAACAGCATTTCAAGGGGAGCGTCGGGATTGAAGCGTTCCAGAGCTTCCCAA
AGGGACTGCTGTAAATCCTCTGATGGCTTGACTTTTCCTGTTTCAATATCGCTTAGATACTGCCTTGTAATACCA
GTCGCAACAGCTAAACGGTTTTGAGATAGTCCATAAGCCAAGCGTTTTTCTTTTAAATGCTGTAACCAAGTTTGT
TCATTCAGTAAAAATCCCTCCAATCAAAAAGGCGTATGTCAACTTTTAAAGCCCATTTGACATACGCTGAAATTT

TGTAAATCCCTTGTAACCAAAGGATTTTCTAATGTTTTTTTGACTGTTTCCTGTCGATTTGTACCCCCCTGTTAG
ATACGGGGGGTTAAGTGCTGGCGTGGCTATTGCCACACCAGCCAGCAAGATCAGTCCACACCTGCGACTTCCGCT
TCGCACGTCGCCTGCGTGGACTGTCTGCTGTTGGATAACTTTTTAATTTCCTCCAAGAAATCATATCCTTTTGGT
ACAAGGGGAGTATAAAACTCTGATATGACACTTGTTCCTACATCAACATAGCCACGACCTTTGATTCGCTTTAAG
AAGAAATCCTTTTGTACGTCACTGCCAAACATCATGCCATAGCCCATTTCAGACATACGACCTAAAGCCACTCTG
AAATTAAACTGATCACGGATTCCGTCGCCTAAATATTTTGCGTCTGGACGTTGACAAGCCAGTATTAGAAAGAAG
CCAGCTTGACGACCTAACATGACAATCTGTTTCAGCTTATTCATAACTGCGGTGTTTTCTTTTGTTCCCAGCATT
TCCATGAAAGCGACGTATTCATCAAAGATTAAGAAGTGTGCCGGGAGACCTAAGTAAGCATAATTTTTGCCAGTC
TTATAGTTCTTCATCTGCTTCATTTCCTCACTACGTTTCATCATTTCTTCATAGAATGTTTCAATGCAAGAAAGC
AAGTCTTCTTTTCTATAGTAGACATTTGCCATCACAGAACCTAAGTCCGCAAGGTCAGCATTTTTCGGGTCAAGA
ATATACAGTTTTGAATCTGTATGAAGCAAGGCTTCAATCAGTGTCAGTATAAAGTAAGTTTTACCGCCACCTGTA
CCACCAGCAATCAACATATGAGGGAGCTTATCATATTCCCACCATACGTTTTTCATTAAGCGAAGTTTACCATCT
TTAGCTTCTACTTCATCAATAGAAATACGACTGGCTATGGTGTCATAGAGCAAAGTATATTCCACATAGGAATCC
TTTAACTCTTTATCCGTCAGCTCACAGTACAAGCCACTCTCTAATTTCTTTTCCAAGTGTAAGAGTTGGTCTTGA
TATTTTCCCAGCGTGATTTCCACCCGTATCTGTATCAAGCCATTTTTAAGTCGATAATACATTTTAGGGAAGTAG
GTTATCTTTTCCTTTGTACGACCAGCACTATCTTTAAAGAAACCCTCTGTTTTGACCTGTTCAGATTCATACCAC
TTGTTTTCAAGTATCATCTTTGCCAGTTTTTGACGGTGGTAAAGTTGTTTAACCGTATCATAGCGAACCCGTTTG
AATACAAACGCTACCAGCAAGCAGATAAGAATTGCGACACTGAAACTGATAATTAAATAGGGAATGTCAATCTTA
TCTGCTTGTGATAGGTTAAAATCCTGCCAGTTGATCTGCTGGATTGTCTTCACATGAAACAGTCCGACAACCAGC
AGGAAAACAGGCAGGAGTGACGCTATCGTAAAATGAAAGACTAAATCTTTACCAGATGGGCGAATCCTTTTACCA
CGCTGTTTCATGCGAAAAAGTCTCCTTTCTACCTAGCGACTATTTGTCTTGTGTCGGTTCTTTCTTTGCTTGTGG
TTGAGCTTTGAATGAACTAGAATCCTTTGTCAGCACAATATCGTCTGCCTTGATATACCAGTCAACATCTGCTCC
TTGATAGGTGGCAGTAGCAACGGTGTCCGCAATGGGATTGATAAGTTCCACCCGTGCGTTATAATCAAACTCTTT
CAAAGGCACGCTGGCAGGAATACTTACTTGAATCATGCGTCCTTGTCCTTTGGATTTTAAGTCATAGGTACGTTC
CTTGATTTCATCTGAAACCGACCCGTCTTCATTTTGGATTCTCACTTCACGACGTAGAGCAGAGAATTTCAATTC
TCCAAAAGTCGTGTCTTTATCTAATACAATGCCATTTGCTAATCTCATCATTTTTCCTCTCTTTCTTTATTCTTT
TATCATGTCGTCAGCATGTAAAAGGTAATTTGTAAAACCACGAGTGCCGATTTTGTAGCCCTCTGCGGTAATACG
TGGATTGACTAACTTCACACGTTCCTCAAAGCCGAAATGTTTTTCGCCAGCTTCAGCAGGAAGCACCACCACAAT
ATCATCTGCTCTTTGAACATCAGAATAGAGATTATAGCTTCTTGATAAGACAGTTAGCCGTCCGTTGATTCTTCG
CTGAACGACTTTATCCTCGCCAGCAAATTCTAAATTGCCGAATGTTTTTTCCATGTTGGGAATCACAAATTTAAG
TTCCATATTTTTACCTATCCTTTCTTTTTTATTGGCTGAATGAATGTTTGATGGTCTTAAAGAGTGGGGAACGAC
CTTTTGATTCTTGATTTTTTGTTTTCATAAGTTCACTTCCTTTCAAAATCGGGTAAAAAAATAGACACCTCATTT
TTTGAAGTGTCTACCTATTAAATATTCAAATTTTATTGGAAGTATCTTTATATCTTCACTTTTCAAGGATAAATC
GTCGTATCAAAGCTCATTCATAAGTAGTAAATTAGTAGTAAATTGAGTGGTTTTGACCTTGATAAAGTGTGATAA
GTCCAGTTTTTATGCGGATAACTAGATTTTTATGCTATTTTTCTAATTAAAAATTAGATAAAACAGAAATACCAT
ATAGTTAATTAGTATTTTCATCATTTAATCAACTAATCAGCCTTCCCAACATCAAGACCATTGGACTCCTTTTGT
GATTGCACTCGAATCTCCAATTGTTTATTCTTCTGAATAAACTGTTGGTAAGGAATTGTATTACTAAACATAAAA
ACACCTAGTCATGCAAATGTAACTAATTTTTTAATTCCACGTTTTTTTCTCATGCAATGAAACCCCTTTCTTTTT
CCTTGTTTTTATTCTAACATATGTAGTTTATTCAGTCAATTATTTTTATTGCATTTTGAACTTTTTTTTTTTTTA
TTACTATAAAGAATTATTTATTCTTTTAACTTGCTATTTTGAACTAAAAATTTTATAATGAAATTAAGCAAATAG
GAAGGTTTATTATCTTTAATGAATACACTCGCAGAGAAATTCAGATTAAAAAGAAAAGAGCTAGGACTCTCCCAA
CAAACTCTTGCAGAAGGAATTTGTGAACAAAGCCAGATTAGTAAAATTGAGAGAGGGCATTTCATTCCCTCCGCA
GACCTTTTGTTCAAACTCTCACAACGACTTGAAGTACCATTAGATTATTTTTTTAATGAACAAATTGAAATTAAA
TCTAACCTCTCTAATTTCAAGCAATTATCTGCTCGACTATTAGATGACAGAAATTATGAAGATTTGGAATATATT
TATAGAATAGAGATTGAACGAAGTACTTTTCTAACACTAGAAGACCGAACTTACCTTGAATGGATTAAAGCTATT
ATTGACTTCTATCAATATGACAGTAAGTGTGAGGCTATTTCTTCATTGGAAAATATATTATTAAAAGTCTCCTCA
AATACTCTGATTTATTTAAAGGCATTGAATACTCTATCTAATTTCTATTCCTTAGTGGGTCGTGAACAAGAATAT
GAGGCAAACTACTCTCATTTAATGGAGTTATATCAGACAAAAAATTTTGAGCATCAAGAGTTTTTATTTGGCTAC
ATCAGAGTTCGTTACAACTACTCTCACTACCTAGTGTCAAAGGAAAAATATAACGAAGCTATCCAAGAAGCTCTT
GAGACGATTGAACTCTGTAAACAAAGACAGACAAGCTACCAACTGGCTCCCCTACTTATTCTTGTAGGAAATGCT
GGAGCCAAATTTCTAGACAAAGAACAAGTCAAAAATTATTATATAGAAGCAAGAGAGTTATGTAAGATTTATAAC
AATCCTTTAATGTTGATGAAGATAGAAAATTATTTGAAGGAATTAGATACTGTATAGTTAATCTTGACATTATAG
TTTTTCTGAAACGTTAAATAACCTGTAAGGCTGATAGTGATATTAATACTATCAGCCTTATAATTTTTCCATGGT
ATCTCCAATAACTATACTGATTTTGTTTCCCGTTCTAATTTGGTAACATTTTGATTTCTTGTATTATCAAGTATA
TTGATAAAATTCTCTAATCTCCTAACCTTATATTCGTAATCATCTATCCATTTAGTCATAAATCTTTGATTTTCT
TCGATTTCTTTCTCCACCTGCTCCAGTTTGATAGTTGAAGTCAGGTTCATCTTGGCAAAGAGATTGAAAAGAAAA
ATGATAAATAAGAGTAAACGATAAGATAAAACCATTGAGCAGTTGAATTTATCCAACAAATCAATAAAAAAACTA

GTAAGGAAATAAGTGAATAAATATAAGGAGATATTTGCTCAAAATGATGATTTTTTTGTATCGTATGTGATAACT
AAATCTACGACTGATGCAATAAAAAATATCATAATTCCAAATATGTTTTATAAAAGATACACATTTTATATAATG
GTTGTCTGGGTTCCTATTTTTGAAAACATATCCCTTCTTCTTTTGAAATTTTCTTACTCAAACGATTGATAAACT
TATTCACTTTAAATACGATATTCTCATATCTATTGCTCTCTAATTCTATCTGGATACCTAACTCATTCAATTCCT
TCTCAACAACATCAATGCCAATTTTATCAGTTATATTCAATTTTGAAAGATTCAATTTCATTTTTTGCTTATTTT
CAACTGAAGCCAGTAAATATTCTGCTACTTGATTTAAGGTTGTCACTCGTTCTTGCAAAGCATTGATTCTCAATT
CTGAAGCTGCCAATTCATGAATTAAATCATCTTTAATTGTAATATAAGAGTGCTTATCCATATCCAGCTCAAGTA
AAGTATACACTTCCTCAATCTTCTCTTTAATCATATCGACTGTCGTTTTTCTGCGGAATGGAATCGTTTGATTTT
CAGAGCTGAACTGTCTAATCAAGGTTCGACCTTTCATGTAGCGATTCTTCTCAGGATCGTCTTTATGATAGACAT
AGTAGGAGCTAGTTTCTCTAATAAAAACTTTCACCTTCTCCTCTTCAATATCGAGCTGCATATTCGGCACAAAGA
TAAGCCCCTCCTGACGAATACCAAACTTGACCTTGATGTAAATACCATCATCTACTACTTTTTCAATTTGATTGA
GTGACAACTCTACCTCAAACTCATGAACGGCATCTCTGTTGCTCTTGAACTCTTGATAGAATTCCCAGAACTTCT
CATCTCTTGGGAGTTCTTTTTCTTTGGAAAATTTTTCTTCTTGATAAAATTGAACCAAATCCTCAATTTCTGGAT
TTTGCCAATCTTTTCTACTCTCAAAATAATCTTGGAAAAATTCTACACCATAAAGATTTTTCTGGTCTAGTTCTG
TCTCTTTCACCTCCACTCCTGCCAACTGGAAATACACATGCTTTTGTTTGGGAATGATAGTTAGATCAAAAAGTT
TCGCAGTTTCAATCAAGTCATCTATATTCTCAGCCTTTGATAAGAGAAATTCCATGAGTTCTTCCATTTCCCTTT
TGGCAAAGTAATTCTTAAAAAATTCTTCTGTATAAGGTTGCTTGCGATTGAGTTTATTGCCACGCACCACCTGTT
TCATGGATGAGTCCGTCATAAAAAAGGTCGCATGCTTGTGGCTAAAATCCATTTCTACATGTAGTAGTGGAGCAT
TCTTTTTGAAATCCTCAAAGTCTCTAGAATGTTCCATCAAAAAATAGAGTCGCTGCTTGAGTTCATACTTGTGAT
TGGTCTTGCGATAAACTTCATACTGCTGGTGAGAATAGCGATTCTCTATAATTTTCGCCCCTGCAATTTTAGAAA
ATCTATCTGAAATCATTCGAAGATTTCGCTCCACTTTATAATCCCACTTAAGTTTTTTGTCAGAATTGCTGTCAA
CCGAGTTTATAATGATGTGATTATGCAGATGGTCTTTATCAACGTGGGTCGCAACGATAAAGCGAAACTTTCCAC
TAGTCAATTCTTTCATGGTCTCATAACCAATCTGATTGATTTGTTCAGGAGTGAGATTGTCCTCTGGCGAAAAGG
ACTGAATAATATGGTGAGCATGGATTTGTCGTTGTTTCTCCTCCAGCCTATCGTGTCGAAAATTGTAAAGCGTGT
CATTGTTGATAAAATTTTCATGATACATCTGCACCATTTCCTCATAGCTAGGAAAGTCCAGAAAATTCCTCATAT
CATAATCCGACACTAAGGTAAGATTTTTTGTTTTATCAGGATTGAGAATATACTTGATGAGCTTTCTGCGATAAC
TCTTTCCATGAATGGCAAAATGCTTAGTGATGACCATGGAACTCCCTCAGCTTTTGAGCTTGTATTGTAAATTCT
TTCTCAACTTCTTTTATCAATTCAGCGATTCCCTTTTTCAACTCTTGCAATTCTTCGCTAGAAATCAACTGAGAT
TGGTTCACACTTCGAGCAATCTGATTGATGTTATTGCCAATCCTCTTCAACTCAAACACTAAGTCTTGGTAGCCA
TTTGTATCAATGGTGATGAAATTCATTCCAGGGTCAAGTAGGGTGCGTCTCGCATATTCAGAAAAAGATTGGCAG
CTACTTTGGTCGATATTTCTATTTAGTTGGTTCAACTCAATATCAGACAAAAACACTTTCTTGAGATTGATTCGG
TAACGATGTTCCATGTGTCTACCTCATATATTTGTCTTGGAAGTCTTGACTGAGAGGAATGGAGTTGCCAACTTC
TTTAATCAATTCCTGTACGCAGGTTAAGAGAATAGATACATGTTCCTGGGTGACTTGACGTTCTGACTGGATCGA
AATTAAAATCTCATGAACATCACGGCTGATTTGTTCTAGTTTTTGAGATTGCCAAAGGGTGAACCATATCTCTAT
CTGTTTTTGTAAATCAGAGGAGAGTAAACTTTTACGGAGAAAATCTGAAAAATTAGTATCTCCCTGCTCTCTCAT
CAAAGCTAAAATTTGCTTTTCCTCTTCATCTGTTAGGCGAAATTGTTTTCGAATATCACGCACGTCTCGTTTCAT
ACTACTACTTCCTTTCCAAAATACCTGTACTGACAATGTCTGCTACCGTACAATGTCAGTACACTTTTACACCAA
CTGATTTTCATTCTTGTAAGGCTTTGCGAGCTCAGATATTGTGTCCACAATATCCCAAAAATCATATCGCCAGCT
GACTAAAACCTTCCAGTTTTGACAGCTAACGATAAGATAACTTGGTGGCTCCGCCCCCAAACCCCCATAGAAAAT
CAAAAATTGATTTTCTATGAATGATATTAGGATAACAGGGAAGATTTGGAAAAGATATCACAGCTAATATAGTTA
TGATTGACTTTCTCTGATTCTATGATAAAATTTCTGTAAACTAATATTTGGAGAAATAAAAATGCTGAGTCTAGA
TCAAATACATTTACTATTGAATACTCCTGAAGATGAGTTTCATGATTTTAAACAAAAATGGCATCATTCTAAAAC
TGAATTGGTGCGTGATATCTTAAATTTTGTCAATACATCACATCATGAAGATTGTTATATCATCTTTGGAATTGA
TAATATCACTTTAGATATAATCGGTGTAAACAATGATGATAATAGAAGAAATGAAGAAGATCTAACAGATTTACT
ACATAAACTCTTTATATCAACAAATAATCAAATTAGAATTAGCATACAAACTGAAACTATAGACAACAAAGAAAT
TGACATCTTAATTATTCATGATACAGATAAAGTTCCTGTATTTTTAACAAAAGATTATAAGCCTAAGAAAGATAC
TGCATTACAAAAAGGATTAATATATGCTAGAAATGGCTCTATAAATACTCCTAAAGACTCCTCTGCTCCATTTGA
GTTAATAAATAAGTTGTTTCAAAAGTTTAATCATACCGACTTAAATATCAAAGAGCAGTATTTTCATGTTTTAAA
AGACTATAAAAATTGGTTCTTCGTTGAAAATGAAGACGGAAGATTTTTTATTTATAATCCTAATCCCGATTTTTA
TATTAAACTTACCGACGATGATGCAAATCGTTTCAAAACTATGCCTTATAGTTTAAATCAATACCAAACCAATGT
TGATTGGCAATTAGTACAACTTCGTTATCGACACCTTACAATTATCGACTTTATGGCTTTATATTTAGATCAGGG
AAATTGTCTAGTGCCCTCCCCTGATTTAGAAGATTTTGAATGTGGTTATTCTGACACTATTTATTACCACTGTTT
ATACAAAAATACTTTAAAATACCAATTGCTGAAAGTTTTCTCTTCAATATCTGGTCTCGAAAAATATCCTTTAGA
TAGATTTAAAAATAATATAGTAATTTATGATGATAAAATAGAATTGGAAAAGACTCACAACTTAATAAAAAGTAA
TTTTTCAACAGAAGAGATAATGAAGCAACTTGAAGTTACAGAAAAAGATTTCGATTTTTACTATAAAAAAGCTAG
ACAAAAAAATCCTGATTATAGTATCCAAGAAAATCAAGTAAATCTAACTGAATTAAACTTAGTTAGGTTATTGAA
AAGTTTTCAAAAAACATATCTTGATAACCCACTATAATCAATCTTTTTAACTAGTAAAAAAGATAAAACAAGGCA

TAGAAATTAGTTTATCTAAAAAACTAATTTCTATGCCTTAATCATTATTAATTAATAACATCAACTAAATTGGAT
TGAGTCAGCAATGAATATATCTCATTAATATTTCTATTATTTTGCGGACTATTATTTCTCAATTTACAATATGTG
AATATTAAGTGACTTTTTGCTCTTGAAAGTGCGACAAAGAATGCACTTTTATCTTCTTCCGGCTGATTATTAAAA
CTCCAGAATGCAGAATCCTCCAAGCCTAGAAAATAGACTACCTCATATTCTAATCCTTTACTCTTATGGATAGTC
ATAATTGGAATTGAGTTTTCACCCTTGAAACTAGAGACTGTATCTAGCCATTCACCTTGTGTTTGAGAATATTCT
ATGTATAATAACTTTGAAAAGTTCTTGACAATGATATCTAGGTCACTTTTTCCATTGTATGTTGAAAAATTGGAA
ATAATTCTTTTTTCATCTATTTTTTCAATAATACAATCAATTAATTTTAGCATACTTTCTTCATTGGGAATAAAA
TTTGAAATTAGGTAAGTAATATCACTAACTATATTGTCAATCTCCTTATAGGATTTTGCTAAAATTAATTCATCA
GTTAACTCATCTATTCCATTGATATTTCCATAAAAATTACTAATATTTTCCCAAATTAAAGGATCTCGTTTTCCT
TGACTGCATGATATTAAATCTAATAATAGGTTACACGTAGGATCTTTTAAAATATCCTGATATTCATTTTCAATT
CTTGCTTTAATCCCTTTGCTATTTAATATAGATATCAATTCGAAACTATAAATACCAACCTTTTGTTTTGCTAGG
ATACAAATTTCTGATGGTCGTATACCTCCTTGAATTTTTGATTCTATATCATTTGCAATTAACTTAGCTTCTAAA
CTTTCATTTTCAAATTCAAATAATGTTATTTCACCCTCTTGAAATTCTGGATAATTATTTGTCTGAATAGAACTG
TGATTACTATTTAATATCTGATGAACCTCTTTTTGAAATTCTACAAGCTTAGGTACAGAGCGGTGATTCATCAAC
AATTGATATTCATTTGGATTAAAGTCTCGAATATAGTCTGGAAAAAATATCAGGCTTTGCACCTGCCCATCTCATA
ATGGCTTGCTTATCATCTCCAACTGCTGTTAATTTACAAGACGAACCTAAAAAACAAGTTTTTAATAAATCATAC
TGGGCATATGTAGTATCCTGAAATTCATCTAGAAATACAAAATCGTATGTCATTTGAAGTGCTTTACGGATGTAT
TCGTTAGTATCTATTATTTGAGTACTCAACTTTGTTATTTGCCTATACAAGAGGACCGGCTTATTATCTTGTGTT
CCTTTTAGTAAGTCAGTTTTAAACTTGTGATTATCCCCATTGTTAAGTATGATGTTTTCGACATATCTTCTTATA
TCTGACATTCTCCATCCATTAACATTAATTCCATTCATAGAGAGTAGTTCTTTTATAGTATACCAATCTTCAATC
AAATAATCTCTTGATGGCCGTATATCCTCAGGTAGAACATCTCTAAATTGGTCTAAAATTCTCTTTTCAAAAGCC
GAATAGGTTAATGAGGTAAAACGAGAAGCATATTCATCACCATAACGTTTCTTAACTCTTTCTTTCAAATTTGAT
GCAGCATCCGTTTTGAAACTTAATGCTAGAATTTTTTTCGGAGAAACGCATTTATTTGTAGAGAATAGATAGTCT
AACTTTTGAGCTAGTAATTCAGTTTTCCCAGCACCTGGTCCAGCAATGACTAAACAGTTAGTCACATCTTTGACG
GCTCCTAATGCAGTCTCTTCTAAAATGATATCTCCTTTAGGAAACCACTCCTCACTCTTTACCATGTTGAATATC
TCCAAGTAATTCTTCAGCACTGTTAACTATTTTTTCAAATACAGGGGGTAGATTTCTTGTTAATTCATCATCACT
AATAGATGATAAAAATTGCATATGAGTTGTTGGTTTTCCTCTACCTAAAAAGAAATATTGATACCAAATCATTAA
TTCTTTTTCTTCTTCATTGAAACTATCGCCATGTCCACTCTTATCTTTTAAAGTAGCTCTTATTGCTTCCTCTTT
ACGTTTGTTGAAACCTTCTAACTGCAATCTATCAAGACAGTCTAAATCACTCAATTTCACTTTTTTACTACTTCC
ATCCGAATCATTATATGAAACAACTGGTCCTTCTATTGGGGATAAAGTATTCAGATAATTGTCTTTATAATGTTG
TAGCATCAAGAAATCAATATCTAAAGGCGATGAAAAGTAGACATTAAATTCTTCGAGTTTATTAAACCAATTAAT
CAGACGTTGAGCTCCTATCGATTCACAACCTCGTGACCCAATTTCATTAAAATCAAGTCTCTGAGTATGGAACCA
TTCTTGAAACTCTGTGTTCAGCTCATACAGATGTTGAGAAATGTATTTTATTCTTCCCCAACCACCTCCGTATCT
CTCATTATCAAAATCCAACAAGGTAATATGAGGAATTCTGAGAGCATTCAATAATTTCCAAAAATAGTTGACATG
TCTGCCCCCTAAAGGGACAATTGAAATCTGAGAGCTATCTACCTCTTTCCCAAGTAAATCAAAAAACTTTGGTAA
TAGCAGTTCCTCACTGTCGCCTTCACCTAGAACTACTAACTTCGCAAAATATAGTTCAGGATAAGCTTGAATTGC
TCCTTTAATATATTTATAAGATTCATCTATAGCTGGAGGTAGCTGTATATCAGAAACAATGGTTTGAAGAACTCT
GTCATTATTTTCTATTCTTAAATACTTCAAATCTTCTGGGTCAATTCTTTTTACAATAGCTGGAGAATGTGACGT
TAAAATTACTTGGGAGTTGTCATTATTGCCTAATTGCTTAAACCTTTTTATCAGTTTTCCAATGTGATGTGGAGC
AATATGATTTTCTGGTTCTTCTATTGCTAGGATAGTTAGTACTGGTGGAATCAACTTAAATCTTGGATTATCAGG
ATTTTCTTCACGGTCTTTGGTAATTTCTAACTCAATATCAAGTATCGAATCAACAAGTGAAAAATAAAAAATAGA
TCTCAGTCCATCTCCTAGATCTGAAACTGTAAATGCTTCTTCAGTGGTTGTTGGTGAAAATTTTAAGGCAATCTG
TCTAAGTGCTGCCGCCATCTCAGAAGAATTAATAATCAACTCTGCTTGAGAAAAACGGTTATCTTCATGATATAA
TTCCCATGACTTCTGAATCTCATTGTTGATCTGAGTCAATGCTCCATTTTCAGATAAAAAGGTGTTATTCAACTC
ATCAATTTTATCTGTAATTTCATTTATCTCATCTTCGGTCCAATTTATACTATTTACCAACCTACTTAACATACT
ACCTGAAGCATTACCCAACTCCTTCTCAGGCGTTCTTGAAGCAGGTACATAAAGTACCCTAATTTTATCCAAATC
CTTTCTAGGAGCACGATGTTTGTCTTCATCTCTAATAGTATCTTCATCAGATGAAATATAATAGAATTGAGTATC
AATACTTCCTTCAACTGTTCCATCATCTTCCCAAGAAGATTCTAACCTGATTCGTAGAAATGGTTTGGCACCATC
TTTAGAAACTGTAAAGTGTTCGAAAAACGATGGAATTGCTTGACTATAGGGAGTTCCATCAAGTTCATCAAACTC
AAAAATAGTTTCTATAAAGAGGTTTCTAGTATTTTCTCCGGGCCTTGAACCTTTTGGAAGGTGAAAATCACTTTT
TTTTATTATTCTATCATTTTGTTTGTCCGAAAACAGCTTACTCAATGCTTGTAATACAGTTGTTTTTCCCGAACT
ATTATTCCCAATCAGTACAGTTTGATTGTTTAATTCAATAATTTGACTTTCACCGAATGATCTAAAATTATTAAT
AATTACTTTTGTTAACTTCATTTCTTATCTCCATTACCTGAAACTAATTCACACAACCATTCCTGTCCCACCTTC
TACATCTCTTCCTCAACCCTACTCTATTACCCAAAAAGGCATTATTGAAGAGTGACAGAAGCTTAACCTTGATCT
ATTTTTGAAACATAGACACATCTTCTATGAATTCCTAGCAAATAGTTGAAATAGTACTTGTTCCGTTTTTATAAC
AGCTAAGGGCTATTGCGTAAAATTTATACAATTCGAGCAAATCTGTTTTTGTCAGGCCATGTGTCTTTCGTAGAA
TTTTATATTGTTACCAATTATAGCATGCTCTTTTTACTTGATAACACTATTATAACATTTAAAAATAAAAACAGA

TTCTGAACTAATAGAAAATATAATTCTAAAATTAGAATTTTTCTTGATAATCATATAAAATGCTACATTTCTAAT
AAACTTGTACTTATTTCCTTCAAACTACACAATTCTCGAGTTGTTGTAAGTTTGTAATTGACTCAAAAGTATTAG
ATATAATTTCAAAACAGTATTTTACTTTATAGTTGACCAATAACATTTAATTCTAACTAAAAAAACCATTATACA
AATCAAGCTACTAAAAATGACTTTATGGTATACTAGTGATAATAAATTGTTATCGGTAGGAGATAGCGAATATGA
CAAATAAAAAAATAAGAGTATTTTTAAGCAGACCTAATCCATTTACAGAGAATCAAAATTTATTTATTAAAGAAT
TGATTAAATTTTTAAGACGACACAATATTGAATGTGTAACATTACAAGCTGCTGAGTATACTCCTTACGAAGTCA
TGAATTCGTTGAGTGAGATGATACAAAGGAGTTACGGAATTATAATTGTTGCATTTGGTCAGACATTTATTTCTA
GTGGAACCAGAAAAATGGGAGCTGAGGATAATCCAGACTTCTTTGCATCCAAAGAGACCCACTTAAAGAATAAGT
GGGTTACAAGTGTTTATTGTCATATTGAAGGGATTTTAGCGTTAACTTATAATCTTCCAATGCTATCTATTCCTC
AAGAAAGTTTAACTGAAGAAGGGATTTTAAAGAAGGGAGAATATTCTATAACATCACCTGAATTCTCATTAGAAA
CAAAAGAAAATATATTAATGTACTTGCAATCAGAAGGGTTTCAAAAAAGTTTTCATGTATGGAAAAACCTTCTTG
ATGATAAGTATAACTTTATCAAAGGAGGGGAAATAAACTATTAATTACCATTTACAATTGCAGTTTTAATCCTCT
GGAGATCCTCACTTAACATAATACTACCTTCATTAATTGTTTTTTTCTGTCGTTCAGTTAAACCCTCAATATCAT
TAATAGGTTTTGAAAGAAGGCCTGAAATCAGCATTAGATCCTCTTGATTGGAAAGAAACCAAATGTCGTCATATT
CACTAGATTTACGATGTATTTTTTCTAAAGATCCTAAAATAGTTTTTGCTGTATAAACAGATGTATAGTAGAACT
CCTTTAAAACTTCAACATCTTTCTCAACTAAGGCTATTTTTAACAGGTTAGCACAGTAATTCCTTGCACAATAAA
TATTTCTATCTTCAAAATTCATGCCTAACCTATAGTAATTAATTGCAGACATTAAGTCACTCCTATTCTTTGTAA
TATAAAATATACGTAAGAAAATAGCTCCATAAATACCTAATAACTTATGATATGTTGTGGTGTTTATATCTATAT
ATTTACTAATATTTTGCTGTGCCATTTTTAAATTTGCTAGCGAAGATTCATTTTTCTTATAGGATGATAGTAAAT
AGCCTGCCAAAATTTCTTCATCAATATATCCACTTTCTAAAATTTGCTGAAATATTTCTTCTGCCTCAGGATACT
TTTCTGAATTCTTGAGTTCCGTGGCTTGATCATAAAGTTCAGCTATTGACTTGTCTAATGCTTCTTCACTTTGAA
TTTCAGTTTTATCGATTAAATCGTTAATTATTACACGGTATAGAGCAGATTCAAAGGCTGGACTATCAGTAAATG
TCTCATCTGACTTTATAGCGGAATCAATATATTCAGAAAGCAGTTTTTTCACCTTGTTAACTTCATCCACTTCGT
TTAACTTATCAGAATCATATCTGATTTGAGGTAAGTGTGCAATATCAAAAAAGCTAATCTTAGCTGTGTGATGAT
CACACAGTATAATGGTTGATTTGGGTTTCATAGCATGACGTAGACCTAACTCATAGATAGCATTTTGGTTTAAAG
TAGTAATATCTGCAATTACAATATCTGCTTTTAATATACCTTCGATAAATGTTTTCGTTATACTCTGTGTACTGA
AAACTTCATCAGCTCTAAAGTGGTTTTTACCATGAACAGAAACTAATTCTTTTTCTAAAATTGTTGGCTTAATTA
GATTATTATAGACTTGATTCAAGTCCACTTCTATATTTGTATTAGGAATTTTTTTAGTATTAAACCCCATAACTA
CAAAACAACTGGTCATTTCTCAACTTTCCTCCCGATAACAATTTATTATCACTATACAAAGCACTCAAATAGGTT
TATAATAGCATGATTATTGATAAAGGAGATTTTTATGGATTATAAGCTTATTTCTACTTACTTAGATTATTGCAA
AACTCATAAGCGTTTGAGTTCACACACGATTCGAGCTTATAAGAATGATCTTATGCAATTTTATAATTCAAACTA
TGATAATGTCGAATCCTATATCGAAGAGTTGACACAATCTAACATAAAAACGAATACATTAAGAAGAAAAATTGC
TTGTATAAAGGTGTTTTATAACTATCTAAAATACCAGCACATAATTGAAGAGAATCCCTTCAATCAATTACGCTT
TCAATTTAGAACTGAAAAAATATTGCCTAAAACGATTCCGTATGATATTCTGAAAAATATTTTTTCATATTTAGA
GCAGAGAGTAGTTATATCTAAAACTGACTATCAAAAACAAAAAGCTGAAAGAAATCTACTAATTATTTCCCTATT
ACTTTCAACAGGTATCAGAATTTCTGAACTTTGTCACATTCATCTCAAAGATATTAATCTTTCCAATAAGACACT
CCATATTATAGGAAAAGGTAAGAAAGAGCGTATCCTATTTTTAGGAGATCAAACAACCTTCAATTTATTAGAAAC
ATATATAAATAAAAACGGAAAGGAATCTAATGATTTCCTATTCCCAGGAAAACATTCGCCTAAACCATTGTCAGA
GCAAAGTGTACGTTTAATTTTAAAGAGAATCGTTGAACAAAATGGCTTATCTAAAACTATTACACCACATATGTT
TAGACATAGCTTTGCAACAATGCTTCTAGATAATGATGTAGATATTCGATATATTCAACAAATTCTTGGACACAG
TTCTATATCAATCACACAAATCTATACTCACGTATCTCATTCAAAACAAAAAGAAATACTTAGTTCTTTCAATCC
TATGTCAGTGATTCATTCTGAAATCGAGTAAGAGACATTTCCGAAGGTTACAGTTTACTTCATCTTAAATTAATG
TTTGTAGTTGACTTTAGTTAATAGATTTTATACTCAACAAAAGCAAAAAGCATGCAAAACTCCACTTTGAATCAG
CATTTATACTATATTCCGTATTAAGAACTCCATAATTGTTTTTCTAAAATATAACTTTCAAGTATCCTTAAATAT
AATGAAAAGAGATGGGCAAAATCTCAATTCCTGAAGAAAATGGAGTAAATCTTCCCACAAGAAAACGCATAATTT
CAAGTTTTTCAACACCTGAGACTATGCGTTTTTTGCTTCTAAAAAAGTTTCGCCCACCTTAAACAACTAACAAAA
GTTATAAGTAGACTATATTTTAAAACTCAAGATCTAACAACCATTTCGATTAGTTAAATCTTTCAATTTTCCTTC
TCCTGATTTAAGCATCTCTTTCTCAACTTGATTCCATTCGCCAAATAGTAAATCATGAAGAACGGTTGCTACTGA
AGTTTTATCTTCTTCTGAATCGTATACACAACTTCTATCTCGTTGGTAAATTTGAATTCTTTCAAAGATAGCTAG
TTTTTCCAATTGTCTTGTATTATCAACTAGATGATTTACAATGAAATCATGATGTTCTTTTGGAGTTGCGCGTGC
TTGATCTGGATTGATAGCATACAGTTTTTCATAACGGATAAGGGTGCTCAGATAGGACAGCTTAGGCTTTGTCGC
AATCAAAGCTAATTGTACTTCATATCCCATACTTTTCAAGAGTTGTGCTGTTTCCTTTGGAACATCAACGCCCTT
TAACTACCCTATTCTAACGCCAAATTGGAAGCGACTAATAATCTCATTAAACTCATCAAACGAAATGCCTTTGGT
TTTCGAAACTTTGAAAACTTCAAAAAACGGATTTTTATCGCTCTGAACATCAAAAAAGAAAGGACGAAATGTGTC
CTTTCAAGATCTTAGCTTTTCTTCAACCCACTGCAGTTGACAAAGAGCCGTAACATTTAGAAAAAAGAAATGCAC
CATTTTTGGTGCGTTACTTCTTGTTTTTCTAGCCTAAACTTTACTTCTAAAAAAGCCACCTTAAAGAGTAATTCC
TTTTTCGGTGGCTTTTTAAAATACTATACATTAAAATACTCTTGTTTAATTTTTGTAAGCAGATTGTTAGCAACC

GAAGTTACACCAACATGCATCAGTTCAGGATCAGAATAATCTAGATTACGAGCATAGCCTTCTGCAACAGTCTGA
GCAATACGCTTATCCAATTTATAATTAGAGGCTTTTAATAGTGCTTGAAATAATTCTGAACTATATAGTTTAATA
TTATTTTCCAATGATACTACTGCCATTTTTGTAACTCCTCTCCTGTTTTTGATTGATAAACCTTCTCTATTTTCA
TTATATCACAAAAAGATTGGTTGTGGATGGATAATTGTTTAAAAGAATATGGTTTTCTAAACTCAGCAAGCATTT
CTTCTTTTGTAATTTCATTCTGACGAAAACGAGCTAAAGCTTGGGTTGGATTGTCATCAGTCTGAACACCATAGA
TAAAATCAAAATGGTGTTGTAATTCATCTGGATTTTCCCGATTTTGAACGACAAATTCAGCAAAATCTAAATCTG
ACTCTGACTTTTTATGTTTTGCAAAATAGTGACAATGATAGTCAGGGTTCTTAAAAATTTCTATAAAATTTGAAA
TACGAAATTCTACTATAATTCCTACTTCTTCTTCTGAGTCAAAAATATTATTATTAGAGCTGCTTCGATTTAATA
CTTCAACTTGCTTGTTAATAAATTTTCTAGCTTGTTCAAAGTCAGGCGTGATATAGAAACCAGGACCAAAATCTA
GCTCACTTCCTAAATTAAATTTAACATCTATACCACTTTTTAAGGATTCTAAATGTCTCAATAATGTAGCATGAA
ACCACTGCGTCTGACCTAGTTGCTTTTCCTGCTTGGTAGTCAACTCCAAAATATTTACTTCCTTCTCTAGTCTAT
ACTAAAAGTATAACACAAGACTTTATTCAAGTAAAAGAAAATTGTATAAAACTATTCACTTCCTTCCGTCTTTCC
TAAATCTCTCTAAATTCTTCTCCAATTGTTCCTTATGAAGTTGATTCTTAGCTTCCTTTAATGTGTCGTCCAAAG
TTTCTTCCCTATAAATTGATTCTGTTTTTACTTGTTCTGATTCTTGATCTGGAGTGAAAATGATATCTAACAGTC
GGTCTATTTTTTCAAGATCTTGGACAGACATATCAGATAGTCTATGAATCAGCTTTTTAAATACATCACTATTGT
CGTGATTTTTCTTAAAAAATGTTGTAAACATCTATAACCTCTCAAAAAAAGCAGCCTATCAGGACTGCTTAGTGT
AAATCCGAAATCGCATCATAAATGGACTGTAATTTCTTATTGTCTTTATTCTTTGTGTCAATAAATGTATGTAGC
TCTTCAATTTTCTTTTGACTGCTTTGAATATCGCTATTGTTATCCGCAATAAGTCTTTTAAGATGTTGCTGATAA
CTTGTAGTGATATCTGTCTCTTTTCCTGTTCTAACCTCTGTAACTTTGGGTTTAGAACTAGAATTTGAATCCGTC
ACAGGTGCTTGTTTTCGCTTTTGAAAAGCTGCTTCATAATTTACATCATTACCTCCTTGATGATTTCTAAATAAG
GCCGCAATTTTATCTGGATCTTCTTGATTTTCTGATTTACTTTCTAATGGTTGATTAAAATTCCAATCTTTTGTC
ATTCACTCATTTCATCCTTTCTAAATTCAGAATCATCGAATTGTCTTTCTTTACCAAAGGCATGGTCAAGAGCTT
CTTCAAAACTCATGTGACTAATGCTTTGACGCCTTTTGAACGTCGCAAACATCGCTGTCTCCAGTTGTTCTTTGT
AAGCAGCGAGTTTTTCTGTCTCTCTTGCTACCTTACTTTCTTGCCTAGTGACCTTTTCTTCTAAGCGTTCAATAA
TGGTCATATACGATCCTCCTTCATTCTAATATTAGCTAAAACATCTTGATTTTGTTATCACAATTCTAAACATTG
AGAGGTTTTTCTTACTGGTTGATTCCTCAATGTCTCTTTTGCATTCTCGATCATCAATTGTAAATCTGATTTCTT
TTCTCTAAGGACATCATTCCAGTCTACTTTTTCTTTCCCAGATTTATTATCAGGGAAATCCAGAAAAACAGGAAA
TCCTGATTGAGATAACTTATCAGAAAAATCTTTTCCTGCATCATCACAATCTACCGCAAGTGTCAATAAATCAGG
ATGATTATCAAAATAGCTGGTGGTATCACGAATTGTATTGATTAAAGGCAATAACTTTGAAGGTATTACTGTATC
CAAAAATTCCAACCTCTGATTTTCTTCAGCTATCAGTCGTAAAGTTTGATAAGCAACAACAGACCTTTTTAATCC
TTCCATAGATACCAAACGAACATCAGTTAGACTTTGTTGATGAAGTTCGTAATAGCTCATTAAGTCGATGAACGA
TTCACAAAAGACCAGTCTATTAGGTTTACCAATATCAAAGGATATTCCAACATGTCCATGGCTTCCTTTTAGAAT
CGTTTTTAACCTCTCTCTAGGAAGAGAGTGATTCTTATAAATCCCTTGGAGACTTGCTGCCTGCAGCTTGTGACG
ATGATTAAAGCTTTTAAAAACAATAACAGGTTCAACTGTTTCATTTGTTTTCCAACTAGCTTGTACTATCAAACC
TTGTTGAATCATCTTTTGTACGATTTCTTCTGAAATTCCTCTACATTCTGTTAAGTAATATCTGGCCAGACTACA
GTGAGAATCTTCTATTCTCTTTAAAGGATAATAAAATGGTCTCTCTCTTTTTTCTTGAACAGTTTCTTTTTGAAA
AGGTTCTTCAGAAAGAAAGGCTAGAGCTTCTTTAAAGGAAATTCCCTTAACAAGTCGAACAAAATCAATGACATC
ACCTTGAATATCTCTTGAAAACCATTTAAAAGTATTGGTAGTTGAAAAAATCCGAAATGAATCGTGTTCAGGATG
TTCATAGACACTGCTCGAAACTTGTTTAAAGGAGATACCTAAACGACTTGCTACATCAAGAATTGAAATTTTCTT
ACATTCTTCTATTTCCATGCAATATCATCATTTTGTCGTAGTATTTGGCAGTGATGGAACAGATGAGGATTCTTC
TAAAGTTTTAGGAGTTGCCTTATCTAAATCATCTAACCCAGATTTCCAAACCTGTACTTGGTTGACCAATAACTT
ACCAGGTAGTTTAGAATAGGATAACTTAACTGTTCTTGTTTCTGTCTGATTCGTCTTTGATTGGTTGGCATTCTT
TAAATCAGATACATAGGTTACATTATAAGAGACCATGGCAATAGCTTGATTCGTAGTCTGATTGACAAAGATATC
AGCTTTTTCGAAATGATAATCTAAAATATAATCCTTATACACTTGGTTCATGGCATCATTTTGACTTGACAATTC
TTGAGAATAAGCCGATTCAGTCATATAAGGTTGAATACGTGTATTATTTTCTCCGAGCTTTTCTTTCGTATAGTA
CTGTGTCAAAAATTCTTTTACAGTATCTGATAACAAAATACTTGCTTTATCTTCTGCTTGTTTGTCCTCTACAAG
TTTAGCTGCAGCCAATTCAATCTCTTTACGACTTTGTTTAGCAGTAGAATGTTGACCAGCAGTATATCCCATCAT
GAGAATAAAGCTAGTTGCAGCCACTGCTCCAACACTAATTAAGGCTTTAGTTTTGACTTTATTTAACATCTTAGA
CCTTCTTTCTATAAAAACTAGGTCAAGAATAACAAAAAGAACTTGTAAGTTTTATCACATCACAACGAAAGGTAA
TTCCTACATTTCAGGCACAAAATCGTACAGTTCAGAGAAAAAAAAAAACTAGCTGTCTTTGACAGTATAATAGACT
TATTGGAAAAAAATAAAATGCATACTGTACTATGCTATACTATAGTTAAGAAAACTATTTACAAAAGATCATGCA
ACTAACTTCTGAGAACTTTAAATTATTTATTAGTGTAAAAAAGGACACCTTCCCAACGAAGATATCCTGTCTAAA
TAAAGCTTATAAAACCAAACACTATAAAGGAGGACGTTCAAAAACGCTAAGTCTATAAAAACAGTTGATGCTAAA
CTTGCGTTATTTATTCTATTGAACAACTCTACGTCTCCTAGCTTTTGATTTTGGTCTTGGATTAGCGACAGTCAC
CGAACGATAATATTGGTAGAAGATACTCTTCGTTCTTCAGGTATATTTGCCTTAGACAACTTAAAAGCAGCAAGT
GTCACTATAGCTATTGATGTCATAGAAAGTCCTATTTCAATGTCCTAAAGAAACCAAAGTAAGAATTATCCTGGA
AAAAGATATACACCATAAAGACACAATCATACTCGATTTAACGACACTTCGTGCCTGTCAATTTTTTTTTTGAGA

TGGTCATACACATGATTTTACTCTTTTTAAAGAAAGTATAGGTCAAGTTTGTCAGAGCAATCACTTGTTTTTGTT
ATTTAGATACTGTGAATATTCATCAGATATCTTTATTCCTATAAAAAGTTCAAAAAGGCATGAATTGACTGTAGA
GGATAAGAAATTTAATAGAGATAGTTATAATACGTATTAAAATTGAGCGTTTAAATGCTAAGTTTAAAACTTTTC
AATTTGTGGAATCATCAACTATGAGTTAAAATAATTTACGAACAGAGGCTGTCCAAAAAACTTGATATCATACGT
TTTATTGTGGAAATATTCATTTTTTCTCCTGAAATTGAGTTTTTTCCCACACTCATTAATGTTATTGATATAATC
CAATAATAATGATAATATCAAATAGTAATAAAAAAATTTGATAGTATTTTTTCATAATCGGTCACATTTTCTAAT
GATTGTTTATGGTAAATGGAGATAAGTATGATTTTAGGTGAAACTTATAGGAAAATAAGAGAAGAAAAGGGGATT
TCTATTTCTTCATTAGCAGGTGCTGAAATTTCAAAATCTCAAATATCTAGGTTTGAATTAGGAGAAACAGAGATT
TCTGTCTTTAAGTTATTGTATCTTCTTGAAAGAATAGGAGTAACACTAGAAGAATTTCTGCTTATCTGTAATCAT
TATCAACCTTCGGATTTTAATACCTTAATAGCTTCTGTTAAACAGGCCGCATATAATGAAGATACTCAAACATTA
CTAGATATGGTAGAGAAAGAAATGGAACTTTTTCGTGGGACAAATTCTCATTACCATAAATTAAATGCTATTTTC
ATTGAAAGTATTGTTTCGGGAATGGATAAGAATCATCAATTGAGTCGTCAGGACGCCTCTTATCTTACAAATTAT
TTATTTTCTGTTGAAAACTGGGGTTATTACGAAACGCTCATCCTTGGAAATTGCTGTCGTGTTCTATCTCCAGAT
TTATTATTTAGATATACCAAAGAGGCGCTCAAAAAAGGGAAGTTGTATAGTCCTATACCTAGAAATAGACAATCT
CTCATTCAACTACTTCTCAATTCTTTTATTATAATGATTGAGAACGACTTATATGAGGAATCTTTATTTTTAAAA
CAGGCTACGAAGAATATATTAGCGGATTCCACAGATTTTTTTGAACAAACTATTTTGCTATATTTAGATGGATAT
TTGGAACTGAAATTCTATCATAATCAAAAATCACTTTTAAAAATCAAAGAAGCTTTAAAAATTTTCGAACTCTTC
AATAAAACAATCTACAAAAATTACAAACAATACTTTGAGAAACACATCATTCATTTAGTAGACTAGGCCATTTAT
CTATAAGGTAATAACGTGATCAAACTCTTCCAATATTTCTGGATTTTGATCATGAGTAATGACAATAACAATTGC
ATCTTTAATGGATAGAATGTAGTCCATTATTTCATTTGCTTTTATTGGATCAAGGTTACTTGTAGGTTCGTCAAA
TATATAAACTGAATATGTTTTGACTAAAAACCTAGCTAAATCAATTCGTTGCTTTTCTCCTTCTGAAATGATTTT
GTTTGATTGAGCTAGGTTTTTACGAAGGAAGTCTTCATTAAAACCTAAGGATTTTCTTAATTTTTCAGTTAAATG
AAGGTTTCTAGCCAATTTGATATTCTCTAGAATATTGCCTTCAATTAGAAAGTCATTTTTTTGCACAAACGCAAT
ATCTTTATATAACTCCTCTGAGGGAATATCAATATTCTGTTTTCCGTTGATTTTTATTTCTCCATCATAAAGATT
TTTTGGATAATAATTTAGTATTAATTTTACTAAAGTTGTTTTCCCAGTTCCTGAAGCACCGATAATCGCATAGCG
CTTGCCTTTTTTAAATTCATAGGAAAAATGATCAAATAATATTCTATTTTCAATTTCATAATATAATTGGCTAAT
AGAAATAGTCGAAATGTTATCACAATCAATTGTACTTGTTTTATGGAAAGTTCCATGATTTGTCTCATATAGATT
TTCTTGGATATTATCAATAATGGACTTGCTACTGCTGATTAAATTTTTATTGTAAAGAATAGATTGTAGTGGAGC
AAAAACACCGTTTAATAATTGAATACTTGCTACTAGTAGTCCAATAGTCAGTAAGTTATTTCTTACAAAAAAGAT
ACCAGCGACCATACAGGATAGTTGAGAGCCAAAACTTAGTAAAATTCCAGTAGTTGAAGCTAAGTCTTTTAAGAA
TTGATATGTCTTTCTGGACTCTTCAAATTCTAAACTGATTCCCTGAATTTTTTCTTGGGTCCAGCTTTGAATATT
TAATAACTTAATTTGTTCAAAACCATTAATAAAGTTTGTCATTTTTGACAAATAGTGACTATTTTGTAGGGAATA
ATTATTAGTTGCTTTAGTCATTAACTTTCCAGGAATTTGAGATAGAAAAATCGTTATACTCGACAATAAAAGAAA
GACTAATGCTAATCGCCATTCTATATAGATAATGGCAACCACACTCATGATAAGGGTCCCTAAATTTGCAATTAG
AGAGATTCTAGGAATCAATAAATTTTCTTGAATAAGGTCACTATTTTTGGTGATATTATTTAAGAAATCTGAATG
GTTACGGTTGTCAATTTGGTTAAATTCACGCGATAAATAATGTAATAAGAGACCCTTTTTTAGATCTATAATAAC
TTTTTTTACATATTGATTTTTAAGATAGGAATAAGAACAAGAGATAAAGAACCACAGTAAAATTAAACTTACACA
AATAGTGATATGAAAGAGTAATCCACTATTTGAATTAGTTATACTATCAAGTATTCTTCCTAATTGAAGAGAGAA
GAATGTCATTAAAAGAGCACTTGTCAAGCTTAATAAAATAATCAAGAAATATAGAAATTTATATTGTTTCATAAT
CTGGTTCATATCTTACTCCTTAAGTAACTCTGATGAGAATATTATAATCCAAATATGAAAATCAGAAACAAACTT
CCCGCATTTGGGACGCAATGAATAATTTACACTAAATATAAAAAATTATAAAATAATTATTTTTTAAAATAAGAG
GAGAACTCATATTTTTATTTAACAATGAATATATCACACTAAACCAGATAAAATATACAAGTTAATTTGTACCGA
AATTACTATATATTATAAGAAAAATAGAAACTCTTAAGCATTCTGTACACTTTAATTTTAATTCATCTCTTTTTG
TTTCTCACACTAAGTACTAAGACGGGATTATAAGCACATCAACAAAAAGACTTTATTCAAACTTACCTTTGAATA
AAGTCTTTTAACTATCTAAAAAGTTCACATTTTATGCTTATTTATGGAGTTAATGAAGCGCCAACTAAATCCTAG
AACCAACATCAGATTGATGAAAAGGTACACAGCCGTAAAACTAGTTAAACCATATTGTCTGATATCATCAAAATA
TAAACTAGGTTCAGTAAAGAACAGATAAATTCCATAGACCTCTACGAAAATAAGAAAACTGACAAAACCAATAAG
GAAAAGTCCACTGGCAATAAGAAAGAGTCTCCATAAAAGAATGATAATTCATCCTACTGCTAAAAAAATTACTGG
GATAAGGAGTAAATCATTCATGACTTTCTCCTTTCTAATCAATCAACTGACGATAATGGGTGGCAAGAGAGGAAT
CAAACTCTTCTAATTTTTGAACTAAGTCCAAATACTCTTCTTTTTCATGTTCTTCAAAGTCCACACCTTGATGAT
TCTGAAGAGCGATTTCCAATTGACTAGTGAGCTCTCGTTTAGAAAAACTGTAATCCCCTGTCACTTCCTCATAAT
GTTCCTTTAACTCCTTGTAGGCTTGGTATTCTTCAGGTGTTTGAAATCCCTGTACCAAAGCCTCTTCTAATTGAT
AATAGGTATCTTCCATCATAATCGTTACCTCGTTTCTTTCTATCTTTATTCTACCGCGCTTTTCTTTTCTTGTCC
GCTATTTATATCTATTTTTTAATCTAATTCAAAAGAAAGTTGTTCTTGCTTTTCTTTTCCTTTTTCGTGGAATTG
TCGTAAGGCCTGATCAATAATATCTAAATCCGTTTCTGTTTCAATCAATGGCGCGAGTACATCGTATTCGGCCTT
TTTAGTTTGATAATCCTCTTCCTTTGGAAAATTTTTCTCAATTTCTACCTTAGCAGTAGTCCATTTATCCTTTAA
TTCATCCAATAAGTTCTGAGTTTTCACTTGGTCATCTTTAATGTGGTCTATCGTATGCTGAAGCCTTTGAATTGT

CCCCAAAGGAGAATACAAATCTAAACTGACAGAATATTGGTTTTCTCCTACAATCTTAACAGAGAAGGTTTCAGG
AAGAGGTTGATTTGTTGCAAGACTAAGCATTTTAATGTCAAATCCTCGATAACTTGCTAGGGTTCGAAATTCTTT
GCTGTCAGATTGATTATGACGGATAAGACGGTGTAGGGATTCACCTGCTTCAGCTCGTTGCTCAAAAACTTGTTT
ACCTATCGTCATAGAAAATGATTGGTCTTTCGACATTTCAGACTGTTGAATGTCGCCTTCATACTTGCTTAATCG
TTTCTCAAGAATGGGCATATTTTCTTCACAGTAAGAGATTGTATGACGATAGTGATCCTTGCTGCGTTGAAAGGC
GCGTCTTTGATTTTCTAATAGAGTTAGATCATTCTCTAGTTCCATCTTATATTTGAGATAAGGATTACCTGTTGC
TAGTGCCTTAAAATCAGAAGCTGTCATAGTCTGTTCATCAATATCTTCCGCAGCACGAATCGGCTCCTTAGAAGT
CATAATCTGCTTAATATAACGGAGTTTGTTCTCCTGAGTTGCCCATAGATAATTATCAAACGAACCTTTGGTAAT
ATAGTGGTAAATATCCACTTCCTTGTTTTCATTTCCCTGTCGGATAATACGTCCATTACGTTGCTGAATGTCACT
TGGTCTCCACGGTACATCCAGATGGTGAACTGCTTTCATCTTGCTCTGAACATTTAAACCTGTTCCTCCTTTTTC
AGTTGAGGCAAGAAGAACCCGAACCTCTCCTGCATTAACCTTTCGAGACAAGCTATTCTTCTTTTCATCACTATT
GGCATCATGTACAAAGGCAATTTCCATACTAGGGATTCCTCTATCAACTAATAAAGCCTTAATCTCAGAATAAAC
ATCAAAGCCATTATCTTTTTTCTTAGGTGTGCCAATATCTGAAAAAATCATCTGAGTAGCCTTATTTTCCATTCC
CTCACGATAAATTCTTTCAACATTATCCACTACCTGAAGCAGTTTATGATTGTCTGCTAGACTATAACTAGAGTC
CAATAAACGCATATCAATAGCTAATTTTCGTGCCTCACCCGTAATTTTTAACATGTTATCCTGACTTGGATCAAC
TGTTCCACATTTGACCGCATCTGATCTCATAACCAATTCTTCTAGATAGAGTTTCTGGTTTTCAGTTAACTCACT
CTCAATAGGGATAATATGGGCTTCTGGTACAGGTAAATCCAACATATCTTGTGTTTGAATGTCGGCTGTTTCTTT
ATAGATTTTCATCAACTCAGGTAGATTGACAAACTTTTTAAATCGTTTCTTAGGCTGGTACTTATCCCCTGTAGG
AGCTAATTCCATAGAGTTTTGAATTTCTCCAAAAGCACCTACCCAAGAGTCAAAATAATCAACTTGATAGCGTTT
TAAGATATCCGGTTGAATGTAGTTCATCATAGTATACAGCTCACTAATTGAATTTGAAACAGGTGTTCCTGTCGC
AAAGACAATATTTTTAAAATCATGTTCTTCCTGAATCTGTCGAACCTTCATTTCCATATCCACATTCTTCTTAGA
CGTTGTATTGGTAATCCCTGCTACATTTCCAAGTCCAGTAATTGGACGTATATTTTTAAAGTGATGTGCTTCATC
CACAAAGAGAAAATCAATTCCTAAGTTCTCAAAATCAATAAAACTATCACGATTAAAGCGTTGGAGTTCTTCCAA
TTGTTTCTCAAGACCACTTATTGATTGCTCTGCTTCTTTAACGGTGTACTTATTTTCAGAATGTGTTTTAATCTC
TCGTAGTTCATTGAGTTTATCCTCGATATAATTCATCTGTCTTTCCTTACTGACAGGGATTTTTTCAAATTGAGA
ATCCCCAATGACAATGGCATCGTAATCTCCTGTAATAATACGTGACACAAACTGTTTTCTTCTTGCCTTCACAAA
ATCTTTCTTAGTGGTCACAAAGACTTTTTTAGTAGGGAAAAATTTCATGATTTCTTGGCCAAACTGAGCAGACAA
ACTAGAGGGCACCACATACAAGGGCTTATGAACCATCCCCAACTCCTTTAATTTAAAGCCAGCACCAAGCATGGT
CAAGGTCTTTCCTGAACCTACCTCATGAGCTAACAAGGCTCTTTTTTCTTCTACAATTCTTTGAATGGCATTCTC
TTGATGAGGACGAAGACTGATGTTTTGTGCCAAGCCATCAATGACTAAATGGCTACCGTCATACTCTCGACTAAC
CGTTCGATTATAAAGACGATTATAACTTTCTTCAATGACTTGTTGAACTTCTGGATACCGTGAGACAAAGTCTTG
AAAGAGTTCTTGTAAATGCTGCTCTTTTGCTCTTAGAACAGAGGTTTTTTCCAAATCTGTGATGGTCTTTTTCTT
TTCCCCTTCCGTAACAGTCATAGTAATAGTCGGTTGGTTCGAATTAAGTAAATTCTCAAAAATCTTTCTTCCTGT
ATCATAACGTGAGCCACTGACTCCAAGACTACTATCTTTGGCACTTGGATAGCGATAAGCAAATGGTGTCCTTAA
ATGAACCTGCCCATCGACAGGATTCACTTCAATGACTTGTTCAACATCAGGCGAAGACAATTCAAATTCACGGTT
GGTAAAACATTCAAAGGCAAATTTACCATAAACGGATTGAGGAATCCAACGTGACCCTATTTTAAACTCAATATC
TGCCAGATGAATCCTTGGAGGGCGAACAGATTCTAACAAATCTAACGCATGAGTCCAATCACATTCTTGGTTGTT
TTCCTCTACTAATAGTTGAACTACTTCTATCTTGTTGAGAATATCTCCTGACAAAAACTGGTTCTTAGAAAGATA
ATTTCTTTCCCCTCTTAAATAGCTTTCTGGATCCATTAAGATCTGGTCACCCAACTCATCTAAAATAGCTGCTTG
GCTATGTTCAGGGTAAATTGATACCATAAAGTCTATATCAACCCCTCTACCATCTGATAAACTGGAGTTTAAGGC
ATCTAAAGCCGTTGAAACTCTTGTAATCACTCTCTCCGGCCTAACCAATGCTTTCTCAAAGGCTAAAGATTTTTT
ATATTTTACTTTCTGATCTTTAGAATCAATGTATTCATCTTCTAAACTTGCTAGTAAAGAATACTTATCGTCACT
ATCAAATAAGTTTCGATTGACTGAGACATTCAAGTATCCAAATTGGCTTACAAACCGATCATAGTCACGATTGAG
TTTACTAAGTAATACCTGAAAATCTGTCCGACTATAATCTGGATGGCGTTGAATTTCAATTAAGGCTTGATAGGT
CTCTCTCAAATCAACCATGCCCTTAATGCGACTAATATCCTTATCCGATAAGGGGCTTTCATAAAAGACCGTTTT
TTTGAACAAGCCCTTATATTTCCCTCTTTTACTCGCTTCTTCTGACTTGTATACATCTAGTGCTTCCTCATCTGT
CAAATGAAGTTGCACGAATCGATCTATTTTATGTTCAGACAAGGAACTGTCCCAAGCTTTAAAATCTCCCTTCTC
ATCTACATAATAACTAATTTCGTCTACTTTTGAACTTCTCCGAATGCCATGCGTATCTCGATAATAAATTTGATT
TCCCTCATATCCAAAAGAATAGAGCGCTAAGTTCTCACGTATATGACTTGGGATAGAATTATCCACTTCTTCTTG
GATAAAAAACAGGTGCTTTCAAAGAATTGTCAATTTGTTTAGGTGCTTCTACATTCTCTAATGCTTTCATTATGTC
AGTAGCTAATGTTTCTGATTCCCCCTTAACATTGAGGGTTCCTCCATTAAAATTACGTACCTCATATTCACCCAA
AACTTGTGTATTGTATTTCCCATCAAAATAAGGATTGATCCAGACACGCTTATCCTCCTCAAAGGGAACAGAGCC
ACTAAAGACAAGTTCCTCCTCATTAAGATTCTTTGCTTGATCCTTTTGAAAGAATAGGAGATCTGTGGTCACTCG
GGTACCTGCAATCTTTTTAAAAGCCGTATCCGGCAACCGAACTCCCCCTAAAAAATGAGTATTGGATTTAATCTC
TTGTAAGACATTATCTGTCCGCTTATCCATTGTCCCAATAGATGAGATAATCGACACTTGTCCTCCGTCTCTTAC
TAAATCAAGTGAGTGTTTGACAAAGTAATCATGAATCATATAAGGTTTATCATAGTTTTTATCGGCAATGCGAAA
ATTTCCAAAAGGAACATTCGTTAAGACTAAATCAAAACTATTATTTGATAGGGAACTTCTTCAAATCCTCGCAC

TTCAATATGGGTATTGGGGTGGAGTTGTTTTGCGATTGCACCAGTCACACTGTCTAATTCAACCCCATAGAGTTC
TGATTTCTCTCGTATACTTCTAGGCATCGCCGCAAAGAAGTTCCCAGTCCCCATAGAAGGATCTAATATCCTTCC
TCCCTCAAAACCATCATCCAGTAATTTTTGCCAAATCTGGCGAATAATCATTGGGTCTGTATAATAGGCTGTGAG
AGAACTTTGTTTTATGGTCGAGTATTCTGATTTACTTACTAAGCTCTTAAGAGTTAAACGTTCTGTTTCATACTT
TGGATTGAGTTCATCGAAAAATTCATTGGCAAGACCGCCCCAGCCGACATACTTGGCGAGTAGCTCTTGTTCTTC
TGGATTCGCTTGTCGTCTCTCTTTTTCTAATCTTTTAACAAGTTCAATTGCGGCGATATTCGTTTCAATCTTTTC
TCGATTTGTCTTAGGATAAAAGTCCTCTAAATCATCTGGAAAAACAAAATCTAGAACAGGGACATCCGTCTCTTC
TATACCTGAAATTAAAGATTTTGTTTCCTTATCCTTATCCTTTTCATTTACATTTTCTTCTTCCAGGTATGAAAA
CAAATCTATTTCCTGACTTTCACTTGATGAATCAATCTCAATCTCTGAATCTTCTTTTTCAAGTTCTAAATGAGA
CAATACTTGTTCAATCTCTTCCAAACTGTTCAAGTATAAGATAGGATTCTCTTCAAATAACTGGTTGGAATCATT
GAATAGCTCTAGGCGAACTAAGTCATTTAACTGCGCATTTTCAATCGAAACCAACTGAAATACTTGTCCTTTATA
ACTTACTTGTGAACCGATTGGATATTCCCTCAAAGCTTCTTCTACAATTTTATCGACATTAGAAAAGGAGTTAGT
TTCTTCCGTTTCCTGTTCAATATCACTTGAAGGAGATAAAATTTTCTCTACTTGATTAGACTTTTCGCTTTTAGC
TTTTACTTCATCAAAATGAGTAATAATCTTCAGCTTTTGTGTCAGTGGGAGTTGTATGTAGTTTTCTTCATGATG
GACAAGATCCGTAACGATATCTAAAGTAGCCATCAATTCTGAACCCAAATAGGCTAGAACATCTCCCTCTTTTTC
TTCAAAATGAATCTCAGGAACTTCTTTTTTAACCTCTTTTGAGTCAACCATTAGAAGAAATTCCTCTATATCTCT
ACTAATACGGTCAGATAGATTATTCGCAACCCGATAGACATTGACTAAATTAACATCCTGATTCTGATGAAATAC
AAGCTCACTTAATGGTGTTAGTTTCTCTTTTTCAGATTGGATGTAAAATCGAGTGGAAAGGTTATAGCTTGCGAC
TTTTAGCACCAAATTTTTCTCAAAATTACTTAATTGAGATAATTTATCTAGTCCTGCTTGTCCAAAATTTCTTGT
GTAACTTTCTAGATCTTGGTCATTATTTTCAGAACTGGCCTCTAGGAAAGAAACAATGTCTTGATGAAACGAATA
GACATGAGGTATCTCTTCTATCTTCTCCTCTTCAGTCAAAAAACTCTCTACCAAGTCCTGCCATTTCTCATCAAA
ATGTTGCGCCTGATAGCGAAGAAATAGGTCCATTTTATCCCTATCTTTTGGAATTGTTCCACGAAACATAGCCAA
TAATTGTATTACTTCCATACTCGCTCCTTTCATCTATACGAAAATAGGAGAATCACATGATTCCCCTACAACTCT
ATCTCCATTTCAACCTCTTGAGGACTGGTTTCTAAATCACTAGATAAAAGAGAAACGGCATCCATTCTCATGTAG
TAAGTATATAAGTCTTCTAGGTCTTGTTGGTTTTCAATCCCTGTTTCAATACTAATCAGAGCCAGTATAAATTCC
TTGTAGTGACTCTCCATCATATCTGAAATCTTTTGTTTCATTTCCATAAATACTGATGACGATTCAGTGATACTT
GTTTGGTCTATGTCTTGTAAATCCTTTTCTAAGGGATTGGTTCCTTCTTCTATTTCCACTAAATCTGAACGTCCA
TCACCATCAGAATCTGAGCTAAGAGGATTGGTTCCTAGGGCCAATTCTTGAGCATCAGTTAATCCATCTTGATCC
GAATCACGTTGATAAATGGCTTCCATATACTTCCTTCTTTCCTAGTTAATTTTTTTTCACTCCAATCCTACCAAA
TATTCCTTGAAAGAAGTATCACAACAAAAAAAGAGAACGAGCCAAAACTGCGTTCTCCTGAAAATGATAAAAATA
TATTTTATACCACAAAAGATATGTAAACTAATCTTTTAAATAATAAAAAAGTTCCGATGTTTGTAAATGTTTTGA
GTAAATTGAAATTACTTTTTGACCTTTTGGAAATACTGTCTTTCTCTTCAAATCTAATAAAGATTGAGGAACAAT
TTTCCTAGAGGTCTCTTTCAATGTTAATGCTATTATCTGTTTTCTTGTACGTAGAGCATAATCAAACTCCAAATA
CAAATAACGTCCTGAACCAGTTAGATGAACATGCTTTCCCAATAACTCCCCAATGGATCCTAAAACTTGTAACTT
TTGCATCGTGGTTCCATCTTTTTTTATTTTCAACTCATCAATTATGACATGTCTATTTAAACAATCGTCAAAGAA
ACTGCCTGCACCTTTTCGGTAATCAACACCACATAGGTGCATGAAATTAGTAGGTGAAAAATGAAGTTCAACACT
TTCAATTTCCGTTTCATAATACATAATTTTGCCAACAAAGTGTTCTTTAAAAAAAGAAGCCGCAATTTGTAACTG
TGTAAAAAAACGCTGAAGTTTTATTGTTTCGGTGTAATTTGGATTACGATAATCTCTAGAATTTGCCATATTTTC
CTCACAAAAAAAGGTGGTTTACAGAGTCTGTAAGCCACCTAGTCAGTCGGTTTATTCTGGTGTCTGCCACCGCTT
GGCCCTTACGTCCAAGATTGCTATCGGATTTCGTTCTGGTGTCCGCCACCGCTTGGCCCTTACGTCCAAGATTAC
TATCAGATTGACCATGAGCCGACCACTCATCTACACTATTTATTTTACCTGAAAATAGCTAAAATTGCAAGTGTA
AACTTCTTTATTTCGTAGTATAGGCAAAACTAATGGCACTATCTGCATGAGAGATTTTTCTAAAGGTATAGTTAG
GGTTGCCACCATAGTTAGTTTCAGACACAAGGAAAGAACCATCATCATAGACCTTCTCTACAAAAGCCACATGAC
CGTAGATAGCTGGTGTACCATGTGTACCTCCTACAAAAGAAACAATAGCACCTGCTCTTGGTGTGGAACCCGTTT
CTCCACCAAGACTTGAAGCTGTCGCAACCCAGTCCTGACCATTTCCCATGGTATTAATGATTGAAATCTTCTCTC
CATTGCTTCCTTTTAATTTTAAGCCTAATTGATTCATACGAGCCGCAACACCCCATGTACATTGTCCATAGGCAT
AGGCCATACCATCTCCACCACCAGGAACAGAATGTTCATACAAGTCTCCACGAACACCTTCAAGGGATTGCGGGT
CACTTTTTGCCTGTCCTCCATTTGTTTGGCTGAAGCCTTTTTCAATTTGGTAATACCATTCCGTTGCTCTGGTTT
GTCTTTCCAGTAGTTTGTCACCAGAATTTCCCTCCCAATAGGTGAGGAAGAGTTGGGCGAGATTGGCTGCACTGC
CCGTATTTTTAAAGAAATCCTTTAACCAACTTTGATAGTAAGGACTATCCCCATGAAGCATAAAATCAAGTTGTA
GGTCTAAATCATACCATTTCTTATTTTGGGTGCGTGCATAATTTAACAAAGCTGTATGACGTGTTGAACCATCTG
CGGTATCCGTCCATTGACCTAACCCCAAACCTCTATGAAGGATATTAGGATAAGCACCACTATAAATGGCTGGCC
CTCCTATCGCTAACCAGGTTTCATCATCCCATGAGGAATCGGTAGCGCCAACTGGAGGAGATAAATAATCCCCTT
CAGCTCGTTTAGGATTGATAGAAGACTCTACCGACCAATTTCCTAAAATAGCCGCAATGGCTTGGGGACTTGCCC
CTTGAGATTTCAAAAAATCATAAATATGTTTTGCTCGTTCAAACTCATCTCCACCAAACTGACCAATGGCAGGTA
AGATAGTTGTCTGAAGTTGAATGACTTTTGGAAAATAAAATTGCGGATTGACATATACCAATTTTTCTTTCTTGT
TCTTATACTTTTGATAGGAAACTTTCAAACCTGTATCATCTGGTGTTTCACCAATAACATCACCCGTTAAGACCC

TTGTCCCCTCAATCGCACGGCCATTATGAATGGAATATAAGGTTAAACGACTCTCATTCTCACCTTTTCCGTTAG
TGAGAATAACATCATCTCCATCTAGAGATACAACTCCATCCATTGGTGCGACAATCGTTTGGTGAGCCTTCGCTT
CTAATAGAATGTACTCCTGAAGAGTAGGTTTTCCGTCTAAATCATAGTATCCATAACGATAAGTCATGGTCAAAC
TATCTTCGTTACTTTTCCCCTCAAATGGATTGTCCAATTCCTGCATGGAAGCATACACACCTTCTTCTTTTAGTT
CCTTTATTTCCTCTTGATCGTCTTTCGAGAGTTTATACTTAGGAGTTTCATAAAGGTCTTGCATGGATTTCAAAT
CTTCCCCATCGTTTAAATCATGCCACAAAGCAGATAGATAATCCTTGTAAGTTTCTGAACTAAACAAATGAACTG
GTTTCTGTAACTCATAGTCATGGAATTTAAAGTTCATATAGCCCATCACATCATCAACTTTTGTGTAATAGGTAA
TTCCTTTGTCATTTGTACGAGTATGTTCTGCATCTTCCCAAGTTAGGTGGGTATAAGCTTTTGTTAATTCAAATT
CATCTTGTTGAATCAAACTAGCAGATGAAAATCCTAAAAAGAAGCTCATCATAAGTAAAAGAAGAAAGACTATTC
CTCCAACTATCCAGATTACAGGATTTCCAGCCACAAATGTAAAGAAGGAAAAGGCTCCTTTTAATTTTTGATAGA
TATTTCGGACACTTAAAAGACCTTGTTTCTTTAATTTTCGATACCGATTTTTAAAGGAACTTGGATTATCTTTAG
TTAGTTTCCATCCTTTTCCATCCTTAAAATGATGGTATCGCTCTTTTGTGTTGGTCAGTCTTTTCTTGGTAAAAC
GACCTGTTGCTTGTCCTGTTTTGACACTAGCTTTTCCAAGATTATAAGAAAGCCGACTGTAGCGTTTCCCTTTTC
TAATGGTCTCATGAAGTGTGCGATAACTTTCTAAATCTTCATTTTCTGAAGCTAACTCTCCACCTTCACGTCCAA
GGACATAAAGAAAGGTTTTGGCTTTCCTACTGATTTTTTTGAACTTATAGGCTTGCTTAGTAGATTTTAGATTCT
CTTTTGCGGCTTTGACTTCTTTCTTAGCTTTTAATTCTTCTAGATTCTTCCCTTGAAGGAAAAAATTGGATTTTA
CTTTCGTTTCCTGACCGTAGAAAAATTTTTGATTGGTTTTTCTTTCTTTACGACTCTCTTTTCTTTCTTCTTTTG
CTTCAACCTTAGCTTCCTTAAATTGTTTCTTGGCTATTTTCAATCGTTTCCTAGCATGAGGCAATCGTCTATCTC
TTAATTCTTTCCGATTCAAAAGTGATGGAGGACTATTTTGAAGAATATGATTGTAGTCTTCATTTGCTTGTTTTA
CTCTAGCCTTTGAAGCTTCTCTCATATTCTCCAGCTTTTGCTTTATCTCATTTTTCCATGCTTTTTCATCCAGTA
CAGCGGAATCTTTTTTCTGTTTTCTCACCTCCTTCTTTCCTTGTTTTAAGAATTTCTTCTCATCTTTTAGACTTC
TTCTAAATGCCTTTCGGGCACGTATGATTTCTCTCTTATCCTTCATTTACCTTCCCCTTAATTAGAAGCCATTTT
ATCAGGATCTGTACTCATGATATCAAACAATTGTGTACCTTGGGGAATCTTATTTTTAAAGGGAACGACAACTGA
ACCAGCTTTTATTAGTCCTGCCCCTTTTTCTGGATTGACTAGGTATTTTTCGAGTTCTTTTGACAAGCCTAAGAG
TTGAACTAGTTCTTCTCGGTCATTTTTTGCTTGCTTGAGGAGAATCATAAATTCACTATTGGCAATAATCCGTCT
ACCATTTGGATCTAACAATAAGGTTTCGACATTTTGGGTTATCCCAGTCGGACTGGCTCCATATTTTCTGACACG
ACTCCACAATTTAAAGAAGAAATCACTGGCATATTTATCTAATAAAAGAAGCTGCATTTCATCAAAATAAATCCA
AGTCTTCTTCCCTAATTTTTGGTTCCGAACGACACGATTCCATATCTGATCAAAAACAACCATAAGAGCGATTTG
TTTCAGCTCATCTCCTAACTTTTTAACGTTATAGATTAAGAAGTTAGATCCTGTCTGAATATTGGTCTTATGAGA
AAAAATATCAAGAGAACCTTCAACATACAGTTCCATATCAAGTGCCAAATTCTGCGCTTCTTCTTCTGGTTGTTG
GCTCAAGACAAAGACCCATTCTTCCAAAGAAGGCTCTTTAAATGACTGATAGGTGAGTCTGGTGACTCGGTCTAT
AATCGATTTTTCTCTCCCATCCATTTTTCTATCCAATAACTTGCCAATAAAAGATAAAAGAAATTCTGATTTTAC
CTTTACAGGATCCTCATCCATATTTTCCTCAGACAAGTCAAGGACATTGAGATAAGTTTGGGAATCGGGCGCAAT
ATCAATCATTTCTCCCCCAAAAGCTCGTCCAATGACACTGTATTCTGCTTCTGGATCCACGATGATAATTTCAGT
ATTTTCACCAGATTCCTTGATTTTGGTCGTGATAATTTCATGCTTAGTTGCCATCCCTTTCCCAGCTCCAGATGT
TCCTAAAATCAGACCAGACGGTGTATTTAATAGGCTGCGATCAATGGTAATAATATTGCTTGAGATTTGATTGAT
ACCATAATATTTCCCACTACGGTCTTGTAAGTCTACTGAAGTCCAAGGTGAGTTCACTGCTATATTGGACGTTAA
TAAACTCCGTGATACTCCCTCTAAAAAATCACAACCAAATGGCAGCAAACTATTAAAGGCTGCTTCTTGCATATA
TGGAAGTTTATCAATCATTAGGTCATTTGAGCCGGCCACTTGTTGGATAGTGTCTAGGGCTTGTTTGAGTTCTTC
TTCATCCTGACCAAAGACCCCAATCAAGAAGACCGTTTGAAATAGTTTATCTCCTGTCTCGGTCATGGTTTTTAA
GAGTTCCTCAGCTTCATCGATATTGCTTTCTAATACATGACCTACTTTTTCCAAATAGATACCTGTACGAGCTAG
TTTTTGTTGTTCCCCAATCTTTTGGGATTCCATTAAGGTTTTCTTTGTTCGTAGTTTCTTCATGGCATCTGCCTT
GGTCGAACTTTGAGCATGAAGGCTTACAATCAATTCCAGATCTCCTTGCATGAGGTCTCGGATAAACTGATCCCC
TAATTCCATACCGTAGTCTCTCACATAGACAATCTGTAATAAGCGGTCATTGATTTGTAGGTAATTCTTGTTTTT
AAAATCCAAGAGATTAGGTGCTATGAAGTGACGAGTTGTCTGGCCAGATCTCGTTAAATCACGATAAGAAAAAGG
AAGATGGTGTTCTCCTCTAAGCATATCTGCCAACAAGTTCACCCGTTCTTCTCCAGTCAAGGATCCAAATCGAGC
ATCAATTTCTGAGAAACCACTCTTGAAATATTCGCCTATTTGAGACAAGGAACGATAAGCTTGTTTGGGATTAGA
ATCCTTTCTACCAAAACTAATCAGTTTCACAGCCGAAAAGTTATTTTCACCACTATCTAAATTTTGATTCATCAT
CCGATTCAATTCTTTACGATAGCTATCGTACCCATCTTCTTTTTCCTCATACAAAACACTGTGTCTGAATTTTTC
TAAATTCAATCTTTTATTAAAAATGGTCAATTGGAAGTTGGTTTGGTCATCTAAAGAGTTAATCAAATCAGAATA
CTTCTCAATGATTGCTCCCTTATCTTCTAAACCAACGGTCTGGTAATTGACATCACCAAGTAAATAGCTTTGTGA
AAAATAATCTTCTTTTACCTGCATCAGACCATTTTGATACAAGGCTTGATAGGAAAGAGTATTAGCCGTTGATGG
TAACACTTCCTCTTTTTTATCTTTAACTTCTTCCTTTTTATTAGTCGTTGAAGTCTTTTGTTTCTTTAATGTATT
TGATTTTCTTTTCATGTTCAAGTCCTTTCTTTCCTGAAATTGTGCGTAGGGGTATCGTTAGTTCAAAATGAAGAC
GGTATTTCAAATAATGTTCAAAATATAAATCATTGGGTTTATAGACTCCAAAAAGCAAGAGGGGGATGGTAAAAG
CAAACACAAAACCGTAAACAAACCAATCTCCAAATTGCCAGAAAAAGAGATTCAAGCCCAAAACAATAATTGTGA
CAATAAAGGCTGGTAAAACAAAGATGATTTGTCTTGTGGTGAAACCTAACCAAGCCCTGTGTTGGTATTTTGAGA

TGTCTTTAAAGACACGTGTATTCATGACTTTCCTTTCTAAAAAGGCTAAGAAGCAACCACTTCCTAGCCTTTATC
TAATTACATACCTAAGATTGAGCGAGCCGTACGTTGAGAACCAACGAGGGCAATAATCAGTAAGATAGCTTGTAC
CAAACTACCAAACATAATCGCAAGTGATTGCAGGACTCCTGCACCATTTGAAACAGCTATTTTACCAGCAGATTC
AAACAAAGGAACAAGAGAAACAATCAGAAAAATAAGAACCCCTTGTACCGCATAGACCATAATATTTTTTAAATA
GCCTAAACCAATAGACTTCCATTCATCACTTAAAAATGTTGGAATCGTAAGAGGGGCAAATGGGATCATAAGGTA
GAGTTGAATAAATCGAATAGATACCAAAAGATTAACCATGGCTGCACTTACTATCCGAACAAGCCAAATGAGGAG
GGCGAAAAAGCCCACAATCATCCGGCCAATAAATCCTGACCCTTTTAATCCAGAGAGTGTATCATACTTTGCCCC
ACCGTGAGCCACAATCGAGGCCACTTGTTCAATGGCATGACTCGCAATCCCGATGATGGCTTCTACAATGACGGT
AGTGTTGGTAATTACAACTGCGACCATAATATAACTAATCAGCATTGGCGCTAAGGCTTCAAAGGTCATCGCTCC
ACCAGAGTTAGCAATTTTCTTAGCCATCTTCGAAAATTCTAGGATGAGAACAACCGATAAAATCGCAACTCCAAG
GGGCTGCATGACACTTTTAGTAATACTAGACATATAAGTCCAAACGGTTGGATTGTAGTTAGATAGTGATTTAAT
CAGATCTACCGTAGATTGTAAATCTACATTAAATCCTTCAAATAAATTTTCAGCTGATATTTTTTCAGATGCAAG
GTAAACAAAGGGTGAGACTAAACTAAGATTCATGTCATTGTTTATCCTCCTAAATTGAAATCTGGGTTACAAAGG
CTCCAGCAGCCCCGACCATAACTCCACCGACAATTTCAAGAATGGCATTCCGAACACCTGGTCCTCCATCTTTAA
TGTTGGTTGCAAGATTGACAATCCCCACAACAACGAGAAAGGCACCAACGGCAATCAATCCCTTCTGTAACAAAG
ACATAGCTTGTGCAAACATAGCACTTGCGTCTACTCCATAAACAAAACCTTTAAAATGCGTAAACATGTATTTCC
TCTTTTCTATTTTTATTTTAAACTAGATTCAAAAGTTAAATCACGAATTCTAAGGCCTTCAAGATGATTTTCTTG
ATTTTGATTCAAAGGATTGATTTGATAGTTCCACCACCGTTCATCGCTTTCTTGATTGGCTAGGTACCTCCAGTT
TGGATGCTTGGTTGAATTGTATTTTTTGCTTTTAAAGACAGGCATATTGGCAATTCGAACCAAGCATTCATGCCG
TTTCATATTTCCGACCTCATCGGGTGTCATTAGATCACGAGCAATCTTTTGATGAGAAAGGGATCCTGAACCTGT
CTGGCCAAAGGAACGACTAGTATTTCGAACATCAATGGTTTGTTTACCGAGTAAACCACTCATAAATTTAAAGGT
ATCTTCATCATTACCACCTAAGTATACTAAGCTATCACAGTTCCCAAGAATGGTTTTCCAAGCTTCTTTTTCTTT
ATAGAGCCCTTGAAGTTGGGCAATATTTTGTAGAATAGGAACGAGACTCATATTTCGAGAACGGACTGTTGAGGT
TTGTTCAGCAAAATCTGGGATTTCTCCGATATTTGCGAATTCATCTAAGTAGACTCTCACATGAAGAGGTAATTG
CCCCTTAAAATCAATATCTGCTTGTCTTGTTAGAGTTTGAAATACTGTTGAAAAAAAGAGGGCTGAAAGAAAGCG
AAAGGTACTATCGTTATCTGGGATAACTAAGTAAACCATTGATTTTTCCTTGCCCCATGTCTTCATATCAAGAGT
ATCTCTTTTGGTCAAATCCATGACACTCTGAATATTGAAGAGGGCAAATTTAGCAGTGGTTACAGCTATAACAGA
ATCCAGAGTCTTATCCTTATAATTTTGAAAATCTGCCCAATTTCGCATGGTAAAATTTTCAGTCCCATACTTTTT
AGCATAATTTTCAAATAGAATTTCTAAGACACTTTTTTCTTGGTTTTCACCCTTGGATAAGTGTTTAATGAGTTT
TGAGATTTCAGCAAAACTTGGATAACGCCCTCGTTTTTTTCGCTCCTCCACTTCTTTTTTTTGACGTTTCAACAA
GTTTTTGTATTCTTTTTGACTTAAACGACTTTCTTCTATGAGCTGTTCTCTTGTTTTGGGTGGATTATAGAAATC
GACCAAGTAGGAGGCTAAAGCTCGGACCAAAGTCATAGAAGCTTCATCCCAAAATGGATCACTACGAGAGCCAGA
GCCTTTGGTGTTATTGAAATAAACCGTCAGCATGCGATTCAAATCATTTTCTGTCTCTATATAGCGAAAAGGATT
GAAGCCATCTGAGTTCTTCATATTGACTAAATCTAACACCTTTACTTGGTAGCCATGTTCTAAAAAGAGTTTTCC
TGTTTTCTCGGCCAAGTGATCTTTAGGATCCACTACAATATTAGAACTATTCATCTGAATTAGATTGGGTTTCAC
AAAGCGAAATGTCTTCCCACTTCCTGAACCTCCGATCACTGCAATATTCTTATTTCTATCATATTGGGGTGGTTT
TTTATCTAATAATGTCAAACGAACATCTTGTGCTAAGATCGTATCATGTAAAAATTCCTTACCGTAAAAGAGCTT
CTTTTCTTTTAGAGTTCCAAAACGGGCGCTCCCGTATTCTACCCCTTCTCGGTATTGTTTTTTACCAGTCTCTAG
ATAGAGATAAACCAGCAACATCATCACAAAGCCTAGTAGAAAAAAAGCACTTGATTTTCCAGTAAAGGAAACATT
CCATGGCGACTGAAGAACTTCATCTTGACCTTCCATCAGAAGATGAATCCATTTATCTAATGTATTTCCAGTATA
GGAATCATACAAAAGCGTCAAACGATGAAAAAGATAGCCTAGTAAGATACCTAACAGTGAGAATAGTATGAATTT
CTTTCCACTGTACATCATCTCACCATCTCTTTCTGTTTGACGGCACCTTCTTGTCTAAAGGTAATTTGGGATTTA
GCCTCATCAATTGCATCGTCTAATGACTTATCCATGATAAAATCAGCTAATTTCTCCGGATCATTAACCATTTTT
TCTAACAGATGGTCTAAATGATTGTCTAGAATCGAACGGTCTTTCGTGTAGAAATGCAGAGAATCCCCTTGCCAA
GCGATGGCTAAAGGAATCTCTTCTTTTTCTAAAAAAGCTTTAAATTTCTCTATATCAATTGGTTTGTCTAAAAAA
TCTTTTTTCAGATTAATCGTATCAATCGAATAGGGAGATTGTAGCAATTCTTCTAATTTCTGCACCCCTATCTTA
TAGGCGGAATCTTGTGCTAAAGCCTGACGTCTAGACCATTCTAGAATCTTTAAAAGACTTTTTACAGTAAATAAA
AGACTACGCTCAGCATATTGAACTGCCATTCGTTCCTGTTGTTCAGAGGACATCTGATGCCTCCTTCTTAACAAA
TAGCAGCTTTCCTTCTTTATAACGATAAGCTATCAATTTCTGACGTTGCTTAATCGACTTGACAACCTGCAGTAG
ATCTCTCGAATAAGGTTCTCGAAGAGTAACTTCTACTGCTTTCCCATCAAAGATACCAACACGTTTAAATTCAAT
GTAGTCTCTTTTGAGATGTCCTAAGCCCATACCAAAAGGAAAGTGATGAATCAATTGGATGGTACAACCACCTTT
ATTTCCCATTTGTTTCAAATCATACAAGTTTACTACCTTCATGATTAACTCCTTTATCTAGTTTGTCGCATCGTT
TAAGTCTGGTAACGATAAACTCCGTGTCTGTTAGAAAATTCTCACACACGTCTTGTGCCAGTCGCCCTTCACAGG
GAAATACTCTCAGTCCCTACTTACACAGGCACGCTAATCAAGACGGAGTGGATTCAATTTTCAAAGAACAGGTAG
CTTTATTATAGATAAGAGTAGTTGAAATTTTTATCACATTTTTGGGGTTGTTGGAATAATAAGGTAAGTGCTAAT
TTCTAGCTCCTCTCACAACACCGTACGTACCTTATATGTTTTATGAACAGTAGCTATCATTTACATTTTCAAACT
ACCATTCTATCAAACTAAAAGTCAAAATAGACGCTTCTACTTCGAGAGATACTCATTTAATGGTTCAGCAATTCG

CTCTACTTCCGTTACAGAAATTTCATCACTATGACTTCTAATGATTTGTTATTAATTCACTTTGAGGGGCTTGCT
CATGAAACCAAACAGTGTAAGTCGTAGAAGAAAAAAGCAGTTCAATTGAACTGCTTTTACGGATTCAGATAAAAT
AACTTTAAAATATAGAAGATATCTATTGACTGTAACTATAATTGATTTAAATTTATAACTTCATCTGCTTGATTC
CAAATATCGGGATTATGGGTTGCAATAATGATTATACGCTCTTGATTCTTTAACATTAAGAGCAAGTCCATCACC
TCTTGTGAGGTTTCTGGATCCAGTGCTGCAGTTAGTTCATCTGCCAAAATTAAAGGTGGATCTTTTAAAATAACT
TTAGCTAATGCGACACGTTGCGCCTCTCCCCCAGATAATTCAAAAATCTTTTGATCCAGAGCAAGGTAAGCCAAT
CCTACTTTTTTGAGCACTTCTTCTTCTTGCTGCTTCTTCTCTTTTTTCGTTAATTTTTGCCCAATCAATCCCAAA
TCTAAATTTGTAGCGATAGTCTCATTTTCAAGTAGGCCAAAGTTTTGAAAGAGATAGCCTAATTCATGCTTAAAG
AAGTGATGCTGTTTGATTTGTTTCAATTCTTGCCCTTGATAGCTGATACTTCCCTTTTCATAAGGCTCTAACTTT
GCCAAGATATTAAGCAAGGTTGTTTTACCACACCCGCTATTTCCGATCAGAGCATAGACCTTACCTTCTGTAAAT
TGCAAGCTCAAATCTTGAAAGACCGTTCGTTGGCCAAATGATTTGGTTAAATGTTCTATGCTAATCATATTAGGC
TCCTTTCAGTACAGTGGCTAACAAGTGGTCTTCTTTATGAGACCGGTACAGGAGAATAAACCAAGCATTAGTAAT
AAATAGCAAGAGAGTGACAAGAGCAATCCACCATTCTTGAGTGAGAAGGAATGTCAGGAAGCTTCCTAATAGTAA
GAGGATACTTTCTGAGAGCAAAATCATTTGGTGAATCTTTACAAATCCCAGACCTGCAATCTTCTTCAAGAAGAT
TGGTCTTCTAAACTCTTCAAAATAGAGGAAATTCATGGTGTTAAAGAGTAGTATGGAAGTAGCTATTGCAAAGAT
TCCTCCTGCAATGGTAATGAGATTCTCTAATTGAATGGATTGGATCATCTGCTGGTATACAGACGCCGCATAGTC
ATATTGACTAACATTTTTTTCAAGCCCATTCTCAACTACTAGTTTCTTGCTTTTTTCCAGACCATCAAAGTAAAG
ATAAGAATTAAGATGAGAGAAATAAGGATTATCGTAGCCACCAAAGCTTCTAGGTAGAACAACAACCAAAATCGG
GTCTGTCAAGAATTGCTGATAGATCATCGGTGTATTATTATAGATAAATCGTTTTTGGTTGTTAGGAAGATAGGT
CACCCGTGCTTTCATAGGCAACTGACTCTTTCCTTGTTCATCACTAGGAGTCAGATAATCTTCATAGCGTTTTTT
CAACTCCTCTTCTTGCCCTTTCAACTTTTCAGGAAGCAAGAGTCCAAATTCTCCAGCTTGCAAGTGATTCAAGCG
CTCTTTTTCCTCAGGGGAAACTAAGATCCTTTGGATATCAAGGTAATTTGGTGTTACATACAGGGTGTTGGCAAG
AGGATCGTAATCTGTGATCGAAAGCTCTCTCCCTGTTCTAGGATCGTTCATGAAGCCTTTTGAATTAAATGCGGC
AAGCTGATGATGGACCAAAAGTCCACCTTTTTCAATGCCAGACTCGATCAGTTTAGACCATTTAGCTATTTGTTC
AATTTGCATTTCTTTGTTTGTCGTTTGTAAAACATTTTCCCGATTCAAACTAATTTGTATCCAATTTGTCTCTTT
AGACCAAGCCAAACTTCCCTCTTGATAAGTCTGCCAGATAGAGCCATAGATGCTGACACGATGAATTGAGAGACC
AATGACAGTGATTGCAAGAAATTGACATGTAAAGAGAAAAAACAAGACTCTTTTTAATGGAATTTTCCCCTTTAA
AAGGCTAACCAAGTGTACTGTCTGAATACTGAATGCAAAGAAAAAGGATAGGAAAGCAGATAAAATAAATAAAAG
TGTATTGTAAATAATACTAGCTGAAATGATGAGAGAATAGGCAAAAGGAGTCAATTGTAAATAATAAATCAGAAT
AGCTCCTAGGACACTTCCACCAATACACCCTAAAAACAATTCTTTACCGTCTTCCATGAGAGAATGTCCAAAGAG
CTGGTATCGTCTTATTCCAGAAATGTAACGAATCCCTGCACTTCTCATTTCAAGTGTTTTTTGAATAATGGTCAA
TGCAGCAAAGCTGATGATAAAAATAACCAAGGCTAGAGATTGGGAACCCGAACTAAAAAATACCATAAAATTTTG
CAGGGGATTGGGCTTATTCATGAAACTTTTTGAAAAACCAAGATCGTGGAGTTTTTGATCTAATTTTTCAAGGGT
TAAATTCCCAGATAGTATATAGTAATTGGTAAGCAAACTTTCCTTTGCAACGAATTCTTTCTCTTCTTTTTTGAT
GCCATTAGGAAGTGCTCCTTCTCCGTAGATATCATACGAAAACTTGACTTGCCCTTTAGAATCCGTTTTTTGAAT
CTGACGAGCAATCAAACTATTGTTTTCTTTTGCTAACTGATCTAAGCTAGATGAAAGCTCTTCATAAACAACTTC
CCTTTCTTGTTGAAGAATCCCTATTACAGGTAGGCTTCGATGGATGACCGTATTAGGTGAAATAAAGGCAATCCA
AAGCAGAAAGATTAGAATAAAAAGGTTCGAGAAATGTATAAATAAACGTTTCATAATTCTACTCTTTCTAGTTTA
CCCTAGAGAGATTTCTTCTCTCTAGGATAAACTTTAGCAATTAAAATCCATAATTAAAGTATGCAGTTTCACCAA
AACTTGTATTTATGAACGATCTTGATGTGTAATGAGCAGAAGCATAACCTACATTAGCTTTGCTGTCACTTGCTC
GAGTCACACTTGACCAATGCCATCGATAGTCGTGGAAATAATTTGAGAATGCTCCCCAATTGCCTGGATTATGAT
AACCTCCGTAACTCCAGTTACCACCAGATACCCAATCAGCAAATACAGGACACGCTAAGCTACAACTAATAATTA
CAGTTAAGACAGAAAGGATAATTTTACTCGTTTTCATGACTGTTCACCTTCCATAATTAGAATTAAAAGCTTTAT
TTAACAATAAAATTATGAATTGCTCAAAATATAGTTTCCGTTTTTTCTCCTTTCCAGAGCAACCCTTTTCGCCTG
CATTATAACACTTACAACATATAATTTCAACTAATTATATAATAGATTTTTGAATATTTAATAATCAAAACAATA
TAGCTGTTTATTCATAAGCTATCAACCAAATTCTAAAACTATTAAAGTTTTTGTATTAAAAAACATTAAGATAGC
ACCTCCGTATAAGATTGTAACTTATCAGTTACTTTAAATAGCTGGGCATAGACATTCAACCAAAACTCGTTCAAC
TTCATAAACTTTAATAAATTGACCAGGAAGGCGCTCTATTTCAATAATTCCTTCACTATAGTGAGAGCGTAAAAT
AATAGGACATATATCTGCTTCCTTAATGTTTTTGGTATTCGTACCATAAGGAAAACTCATCGTCATGTTAAAAGG
GATAGTCAAAGATAAACCATGTAACCAAAGTGCTGTCTCCAAAGAAAAGATTCCCTTTGGAAATCGATACTGAAG
GACAAACCACTCATCTAAATAAATATCAGGTAAGCGATATAACCCTTTTTCTTCAGCATCGATTTTTCCTTCTGC
AAGCATTTTCAATAAGGTTTTGTAGTGTAAATTTTCATCTATTACTTGTTTAAAAGTTAAAAAACCATACTGATT
GAAAGAGTTCATCAGTTTTTCTCTTTTATCAACCATTTCATTCTCCTTTCTTAGAAATAAAATGCTAACATCTTA
TATATCTGTTAGCATTTTATTTCTTTAAAATCAATTCATTTAGTTTTTTATTTCAAACTGAATCTATAAGTAAGC
TAGACCACCTATTATAAAGAAGAAGTAAGCCATGGACTAATAGTTTCCACTCCAAATGAAGCCCTAGAAACATTT
CTAGTAAAAACATACAAACAGCAAGATAAAATGCTTGTCAAAGAAACTCTGATATCTAATTTTCTGATTAACTTT
CATAAAATTTCTCGATACAGAAATTCTTCGACAATACTCACATTTGTTAAGAAAAGAATAGTGATGTAATCATGA

TTTCAATTTTTAAATCATGATGGCCATTTCACCTTTCTTGCCTTAACTGGCTTAAATCTAATCTTATTAATGAGT
CTAAGATCTATTTCTCTACAATCTGTAACGGAGGTATCATTGATTTTAAGGCTTCTTGGAAAGTATTGATTACCT
TGTTCACGTGATAGTTCTACTGTATAACTAATACCATTACTTAATTTAAAGACTACAAAATAAATATTATCTCTT
CTAAATCGGCTACCAACTCTTCTATTCAAAATGCCATCAGTTGTATAGAATTTACACTCCATTACATGTAACATT
TGATAAAGCAACAAGGTTCCAACTATTTTGAATTTATTTTCGTTGAACCAACCATTATCAGCTTTATTCAGGCTT
TCCAATGTCCAATCATCTTTTAATCCTTCAAAAATACTCCCTGCATTATTAGTTGGTAAATTAACATGATGTTCT
CTAGATAATCCTAATAGATGAGGAATGGAGGAGTTTGGGACTGATAGAATAATTTCAGGACAGTTCTCCATATTT
GTCTGATAAATATATTTGTACTGAGAAACATTTTTCTCAATAGTATTGGCCAAGCAATTCATCTTAATATTGAGA
ATATCAATCTCTCCATCAAAAGTCTTTAGCCATTCCAAAGTATAAGTCATTTGAATTTTCTCCTAAAAAAGAGAA
GGAAGCTCCTGCTTCCTTCTCTCCCACGTATTGAGTTTCACCTCACGCTAGATATGCCCTATCTATTCCTCAAGG
GTAGGCAGTCCTGCTATTCTTTTTCTCAACAAACAGTCTGAACTGAGAGCGGAGCCCACAGGCGGGTGCAGACCA
TATCCACTAGCCCACAAGTAATTATATCATTTAATTATCTTCATTATACGCAATTAGTCCATATTTGTCAACAAT
TACATTCATTATGAATATCTAAAAATCAGGTCACCTATTTTAAAAAAGCAGCAAACTATAAACTAGTAGGTTCCA
CACCAAATGTAGCCCCATACTGCCCCATAAGTCCGATTTGTAGCGTACAAGCCCTAAAAACATCCCAAGTGAAAC
ATATAAACACCAAGCTAGAATGGTTCCTGGATGATGTGCTAAGGCAAATAAAACACTTGTCAAAGCAACTCGAAT
ATCTAATTTTCTAACCAAGTTCCATAAAATTTCTCGATACAGAAATTCTTCAACCATACTCGCATTGATTAAGAA
CAATAAAAATGAAAACCAAGGGACTTGATGTTGAAGGCCAATTAAGTTTGCTTGATTCGTAGTTCCTTGAGCATG
GATTAGGCTAAAACATAGACTTATAATCAGTAGGCTAACGAATCCAATACCAAGCCATTTCATCCTAGATTTCAT
ATTGACCTTATGAGCTTGTTTGCGTTGACCATACATCCATAAAAAAGAAATGAGTGACGAACTATAGAGAATCTG
TAGTATAGTTAACTCACCGATACAAAGAAATTTCAATAAGTATAGAGATACCAATAGGACATTTACTTGTTGGAA
TATATAAACTGGAATTATTCTTTTCATAGTTACCTCCGAAATAAATCTTCATAATCTAAATCTAATACCTGCACA
ATCCTTTCTACCCATGGACTTTGAGGCATTCGTTGTTCCATCTTGTAGTGACGAATCTTTTGATACAAACGATTC
AATTCACTTGGATAGTGAAACTCTCCCGCAAACATTTTTCTGGTTAACTCAATCCAGCTAATATTTCTTTCAGCT
AAAATAATGGACAAGTTCTCCCAAAATCGTTCAGCCATATTGCTTCTCCTTTAGTTAGATAAATAATGTGTTTGT
GCCATGTAAATCAATTGTTTCGTATCTCTTGGCAATAGAGCTCTAGCCTCTTCCAAATTCAGATTTGGATAAACT
CTCTTATTTGAAACTGCAAGAGGAAGTCTGATGCTTAGTTCAGGATTTTTTAATATCATTTCGATGAAATCCGTT
AATCTTAGATTGTCACGGTTCTTAAATCGTAATAAATTAGGAGATAAAAACTCAAAACAATCTGAAGAATAGCTC
ATCATCTCAATTAATTTGTCCTTTGTCATTTCAGAAATCGAATGACAAGATACCTCTATTCCGTAGTTTTGAAAG
AAATCTAAAAGAATTTGATTTCTATGGTTATTTTTACTTAGATAGAGATCAATCATGGGAGACCTCCCAAAGATT
CGGTTCCATTTGATATTCTGAGACGATTAAGGAATCTAATAGATCTGAGAAGTTAATTGATTTCTTGTCTTCATC
ATAGGCTTTTACAGTTACTTGGGTTGTAAGTATTCCCTCTTTTCCCTCGGCTCGATAGCCTTGTCCATATAAAAC
AAAAACAAGATTCTGATTATCATCTATAAAGGCATCAGCTCCGTTCTTTATATCCTGACTTTCGAGGAATTCCAT
AACGTTTTGAAGATAGGATTCATAAAATAGTGGATAGTTATGTTTTTTATGGTAATCATCTAAAAATGTCACTTC
AAACTCACATAGATAATTGGGCATTAAAAATATTTGTTCATCCAGCTGTTTGATTTCTGCATCATGTAATTCTGT
TTCTAATTCGTCACAATCTAGTATTGACTCTTTATTTAAAGCTTTCATCTTTTTCCTCTATTTCTTTTAATTTCT
TTGCGATTGCAGCAATCACAGGAACGGTTACACTATTACCAGCTTGTTTATAGAGCTGACTATTACTAGAGACTT
TTCTAGCAGCTTCAAAAGCCCAATCAGGAAACCCCTGCAATCGAAAACACTCTTTAGGAGTGATTCGTCGTATTC
TCAAACGGTAAAATTGTCCATTAATTAAAACACCGGCTACATGGTAAACTTGCTTATCTTCTCCTTCATAGCTAG
CCACTACTACTCCCATTTGACCACTAGTTGTCAACGTATTCGCTATACCTTTTCCAACTCTACCACGCCGATACT
GAGAACTTGGTCTTTCTAAATTGATTGAATCTCCAATCTCTGCTTGAGCGTACCCTTTTTTAGTTGCTTCCCTGA
TTTTTAGAAAATGGATTGGTTCTGGGATCAGTATTTTGGGAATTTTATCTCCACCTTGCATCGTAGTTAGTGTTG
GAGATAACCCATCACTTCCATATACGCGACCAGTCTCCTTAAAGCTAGTTGGTAAATCTCCAACAACAACAATAC
CATGGAGATCCTGAGTATTCAAAGTAAACATTGGCTCTTGGTTTTCCTTAAATCGTCTACCATTTTGTCTTTTTT
CTAATCTGTCTGGTGTCATACAAGGAATCGCAACTTTAAATCCTTCTCCTTTTCCACGAACTAATGTTGGCGCTA
ACCCCTCTGAATAATAGACTTTACCGCTCATGCCACTTTTTGATGGATTCAAATTTCCTAGTGTTTTTAAAGTCT
CAGAGTTAGTTGCTTGACCTTCTCGTCTGAAAGGAAATAGGAGTCGGGTACCTTTCTTTCTAGAATGTCCGATAA
TAAACACCCTCTCTCTGTTTTGGGGAACGCCAAAATCCTTACTGTTAAGCACCTGCCACTCAACATCAAACCCCA
ACTCATCAAGTGTGGTAAGGATTGTGGTGAACGTCCGTCCCTTATCGTGATTGAGTAAGCCTTTAACATTTTCAA
GAAAAAGAAAACGTGGTTGGATTTGTTTGGCCGCCCGAGCAATTTCGAAGAACAAAGTTCCTCTAATATCTTCAA
ATCCCAATCGTCTTCCTGCGATTGAAAATGCTTGACAAGGGAATCCCCCACAGATGATATCGACTTTCCCTCTAA
GTTTTTTAAATTCGTCATCTGAAACATCTCGTATGTCATGAAATTCGATTTCTCCTTCCGTTTGAAAAATGGATT
TATAAGATTCTCTAGCAAATTTATCAATCTCACAAAATCCAATACACTCGTGTCCTACACTTTCCATTCCAAGTC
GAAAACCACCTATACCTGAAAATAAATCAATAAATCTCATTTCTTGATTACCCCTTTCTGATCGACTATCAATAT
CCAAAGTGTCACACCACTTGAAATGAAAATGAGCAAAACTAATATCCACTCGATTGACGACATCTAACCACCTCC
TTTTCTAACACGGTTATTCCAAACAGAATACAGGCCATTAAACACAAATTCAGCAAGTACTTCCGCTCTTCTAAC
AAAGCGAACATTGTCAAGAGCATACTGATAGATTCTCTCAAAATCAGTCATAGCAATTTCACTGGCTGTTTCAGA
AAAACCTTCTCGTCTAAATTGATCTTGTACCAATCCCCAAATATAATCTTGATCATATTTTGTGACCTTTTCTAC

TTTTCTTTTCAAGATAGGTTGAGTATACCTCTCCTCCTCATCCTCAATAAATAAAGAATCAGTCTCACTATATTT
AGTCTCACTAACTTCAGTCTCACTAGGGGCTGAATGTGAGACGGGGGCCGTTTCATTTTCATCCTGCCCTAGTCT
TTTTTTAACACTAGGCCTGTTTGAATTAGCTACTGGGGTAGAAGACAATTCCCCTAAATAAATCTTATTAGCAAG
TCTCCCTTTCTCACTTGAAGACTGTTGAACTTCATCAATTAAGTCATATTCTTTAAGAATTTTTTTGATAGACAG
TAATTTTGACTTAGAACAACCTAACAACTTCATCAGTTTAGAATTAGAAAATACTAAATAAACCGCCCCTTCTTC
ATCTATCCAACCACGACTGAGAGATAATTCTAAACGATCTTTTAAAATAGAATAAGCCACCTTTACTTCTAGTTT
CATATCCATATATTTTTCATCCTCAAAAAGAATTTTAGGTAATTTATAATACCGTTCTGAAGTATGGTATTGATT
TGCGGTAATTCGTTTCATAGAGCTCCTCCTAGTTCTTTGAGTATTAAATCTCCACTTGATTCCAATCGAACCAAG
CCACTTTTTTCTAATTCTGCCATAAGAGAGATGGCTTCAACAATATCAATTCCCATTTCACGTACTAAAAATGAA
ATGACAATATAGCGTGATGATTGTAATTCTTTTGTCAT

B. A. Nucleotide sequence of *tet*(M) and *erm*(B) containing regions which share 99% sequence identity with the putative ICE from *S. pneumoniae* R34-3225 (accession number; LK020687.1)from *S. pneumoniae* strain 291015 (accession number; JABAHF000000000)

TTAAAAAAGCAGCAAACTATAAACTAAAAAGTTCCACACCAAATGTAACCCCATACTTCCCCATAAGTCAGATTT
ATAGCGCACCATACCTAAAAACATTCCAAGTGAAACGTACAGACACCAAGCTAGAATGGTTCCTGGATGATGTAC
TAAGGCAAATAAAACACTTGTCAAAGCAACTCGAATATCTAATTTTCTAACCAAGTTCCATAAAATTTCACGATA
CAGAAATTCTTCAACCATACTCGCATTGATTAAGAACAATAAAAATGAAAACCAAGGAACTTGATGTTGAAGGCC
AATTAAATTTGTTTGATTCGTGCTTCCTTGAGCATGAATCAGGCTAAAACATAGACTTATAATCAGTAGACTAGC
TAGTCCAATACCAAGGCATTTCATCCTAGTTTTCATATTGACCTTGACCACTTGTTTTCGTTGACCATACATCCA
TAAAAAAGAAAAAAGAGACGCACCATAGAGAACCTGTAGTATAGTTAACTCACCGATACAAAGAAATTTCAATAA
GTATAGAGATACCAATAGGACATTTACTTGTTGGAATATATAAACTGGAATTATTCTTTTCATAGTTACCTCCGA
AATAAATCTTCATAATCTAAATCTAATATCTGCACAATCCTTTCTACCCATGGACTTTGAGGCATTCGTTGTTCC
ATCTTGTAGTGGCGAATCTTTTGATATAAACGATTCAATTCACTTGGATAGTGAAACTCTCCCGCAAACATTTTT
CTGGTTAACTCAATCCAGCTGATATTTCTTTCAGCCAAAATAATGGACAAGTTCTCCCAAAATCGTTCAGCCATA
TTACTTCTCCTTTAGTTAGATAAATAATGTGTTTGTGCCATGTAAATCAATTGTTTCGTATCTCTTGGCAATAGA
GCTCTAGCCTCTTCCAAATTCAGACTTGGATAAACCCGCTTATTTGAAACCACAAAAGGAAGTCCGATGGTTAGT
TCAGGATTTTTTAAAATTATCTCAACGAAATCCGTTAATCTTAGATTGTCACGGTTCTTAAATCGTAATAAATTG
GGAGATAAAAACTCAAAACAATCTGAAGAATAGCTCATCATCTCAATTAATTTGTCCTTTGTCATTTCAGAAACT
GAATGACAAGATACCTCAATGCCATAGTTTTGGAAGAAGTCTAAAAGAAGTTGATTTCTTTGGCTATTTTTACTT
AGATAGAGATCAATCATGGGAGACCTCCAACAAATTTGCTTCCATTTGATATTCTGAGACGATTAAGGAATCTAA
TAAATTTGCGAAGTTAATCGGTTTCTTGTCTTCATCATAAGCTTTTACAGTTACTTGGGTTGTAAGTATTCCCTC
TTTTCCCTCGGCTCGATAGCCTTGTCCATATAAAACAAAAACGAGATTTTGATGATCATCTACAAAGGCATCAAC
CCCATTCTTTATGTCTTGACTTTCAAGGAATTCCATAACGTTTTGAAGATAGGATTCGTAAAATAGTGGGTAATT
ATGTTTTTTATGGTAATCATCTAAAAATGTCACCTCAAACTCACATGGAGAGTAATTTTGACTTTGAACAGCCTA
AAAGTGCCATCAAATTTGAATTGGAATAAATCAAATAAATAGCCCCATCCTCATCAATCCAAGCTTTGCTCAAAG
ACAACTCCAACCGATCTTTTAAAACTGAGTAAACCACCTTAACCTCCAGTTTCATATTCTTATACCGTTCACTCT
CAAATAAAAGTTTGGGGAGCTTATAATAACGCTCTGATGTCTGATATTGACTAGCGGTAATACGCTTCATGATTG
TCCCTCCAAGACTAAAATTCCAACATTTCCAAATTCATCAAATCGGATTAAACCTACTTGTTCCATTTCATCAAC
TAACTGAGTTGCTTTTACAATATCAACTCCCATGATAGTCATGAGATGACTCTTCACGAATTGACGTGATGACTG
TCCTTCCTTTTGCATAATTACCTCCGAAACACAAAAAAAGGGGTAGACAATCTAGTGTCTACCTCCGAAAATTTA
TTAAAAAGAAAAACCCTGCCAAAGAATTTTTGGTAGGGTTTTTGGTAGGAAACTAAATAAATTTATCAGTTTCTA
AAATGTGTTCACGATTCTAAAAGGCTGATACTATAGTATTCCGAATTCTAATTGGTATATGCCTCTTATTTATAA
TAACTATCTCCTCCTTTACACTTTAATTCAAATCTTTATTAAAGAATATTTCATCTTATTTAACAAGAAACCATA
TTTATATAACAACATAAAATACACTAAGTTATTTTATTGAACATATATCGTACTTTATCTATCCGACTATTTGGA
CGACGGGGCTGGCAAACAGGTTCACCGGTAGTAACATGGTACCCTTTTAACTCTGTTAAACAAACACTACGTCCA
TTTGTAAAGAAAGTTAAATCACTACGATATTCTTGAATACACCGAGCAGGGATTTCTCCACTAAGAATGACCTCA
TTATTTTTCAATTGAGTGTCTACGATGTTCGCACAATATTTAGGAGCATCGTTGTATGCTCGTGAAAGATATTCC
TGTGGCGCATAAATTTTAAAACTAAGATATGGCTCTAACAATTCTGTTCCAGCTTTTTTTAAGACTTGTTCCAAT
ACAATAGGAGCAAGCATCCGAAAATCTGCTGGGGTACTAACAGGGCTATAGTATAAGCCATACTTAAAACAGATT
TTACAGTCCGTCACATTCCAACCATACAATCCTTGTTCACAGCCATAGCGTATCCCCTCCATAACTGCATTTTGA
AACGATTGATTTAAGTATCCAAGAGAAACCGAGCTCTCATACTGTACTCCGCTCCCTAATGGAAGCTGTGCTACA
GATAGACCAATGGAAGCCCAGAAAGGATTCGGTGGAACTTCGATGTGAATGGTATACTCTGCTTTTTTTAACGGT
CTTTCCATATAAATGACTGTAGGCTCTTTTATTTCTATCTCCACATGATACTTTTCTTGCAGCAGAGCACAAGTC
ACTTCCATTTGTACTTTCCCTAAGAAAGAAAGTATGATTTCATGTGTCGCAGAATCCACATAATATCGCAGAAGC
GGGTCACTGTCGGAGATTTCTAAAAGTGCATCAAGTAACATTTCCCTTTGTTGAGGTTTGCTCGGTTCAACAGTC
GTTTGCAGCAGAGGGAGGGGATTTTCAATTCTCTCTCTGTGGCAATAGCTTTGTATCTCCAAGAACACTATTTAA
CTTCAAAAACTCATTCTGCAAAATAACAATTTCCCCGGAATAAGCCTTATCAATTTTACATAATTCACCATTTAT
TGAAGTATACATTTCTGTAATTTTTATTTTTTCCTTTTCCGATATTCTAACCGAATCTCGCAAATGCAGTACGCC
ACTATAAAGACGTATATATGCAAGACGCTGTCTTTTTTCCGAATACTCAATTTTGAAAACTTTTCCGCAAAGTTC
AGACTGACCTCGATGTGTTGATGAATAAAATTTATTCGTAATCACTTCTATAAGGTTATCAATCCCTATATTGTT
TTTTGCACTTCCGTGATAAACAGGGAACAGAGAACAATTCTGAAATCTTATGCTTTCCTCTTGTTCGAGTTCCAA
TGCTTCTAATGATTTACCGGACATATATTTCTCTAAAAGGTCATCGTTTCCCTCTATTACCGTATCCCATTGTTC
AGATTCGGTAAAGTTCGTCACACACATATTAGGATACAGTTCTACCTTCTGTTTGATTACAATTTCGGCAGAAAG
TTTCTCTTTAATATCCTGATAAACCGTTGATAAATCAATTCCATTTTGGTCAATCTTATTGATAAAAAAGATTGT
GGGAATCCCCATTTTCCTAAGTGCATGAAATAATATACGAGTTTGTGCTTGTACGCCATCTTTTGCAGAAATCAG

TAGAATTGCCCCATCTAAAACTGATAATGAACGATATACTTCTGCTAAGAAATCCATATGTCCTGGCGTGTCTAT
GATGTTCACCTTCGTATTTTCCCACTGAAAAGAGGTTATTCCTGTCTGAATTGTAATTCCTCTCTGACGTTCTAA
AAGCGTATTATCCGTCCTCGTTGTACCTTTGTCCACGCTTCCTAATTCTGTAATCGCTCCACTGTTATATAATAA
GCTTTCTGTTAAGGTAGTTTTTCCTGCATCAACATGAGCTAAAACTCCAATATTAATAATTTTCATGTGATTTTC
CTCCATTCAAAAACCCAAAAGGGCATAAAAATCCCAGTGATAAATACTTTTATCACTGGGATTTTTATGCATAAC
CATAGGCATACAAAGCATACAGATATTCTCCGGATACTTTAGAATCACATGATAAAGGTATTCTTAAACTGGGTA
CAAAAAACTAAGCCCTCCTAAAAAAGGACATCCAATTATTTGTTCCCACTATCAAATTGACAGTTTATTTAAGAA
TACCTTGCCGCATATTTATTAACTCCTTTTAAATAGATACTTAAATAATAGCACGTAAGAGCATATTTGTCAAGG
AATCTCCAATTTTTTATCAAAGAGAGTACGTGATTACAAAATAGCTGTAATAATGTACCAATATTTGTTATTCTA
TAATCTTCCAATTACTCCCGTTCTTTTCAAGTACCAAATCAAATTGAGATACCTGCGTTGCTTTGGTCTGCTGGT
CGATATACTCCACTGTCAGCGATACCGTGACTTGATTATCCTTACGATTGTGAATAGGATTTACCAGTTCTTGAA
AGATGTACTCTTTTCCGATTGGTTTTAATATCCCGTCATTCACATAGTAGGAAAGTTCACTGGCTGTCGCTGTAG
GATAGAGCTTGAAGAACGTCGTTAAAAACTCATTGATTTCATTGGTTGTAATGGAATCAACCGTCCCCTCACTTT
CAATGGCTTTTGGTTTATAACTTGATTTCTTAGGTATGTTGGTAATGGTCGGATTCTTAACCAGTACCATATTTC
CAGAACCATCTACATAGACACTCACTATATAAGCAGAGTGGACGGTCTTTGTATTTTCTCCCTCTGTAATGAGCT
GGTCTACACTGTAGGTTACATTAAACTCATTGTCGCCAGTTGGCTCTACCGTCCATATCTGAAATCCTCTTACAG
AAGACGATACAGGAATATCTTTGCGTACTGTATCAACATTGAGAGCTTGAAGTTCATCTGTCAGATAGCCTTTTA
GACTTTCCATTCGATTATCAATGGACTTATCGGATTGCTCCCATGAATAGTAGACTTTCGCAAAGTTCTCTACAA
AATTTTCTACATGATGAGTATCAACGTATTCCTTTTCTATGATAGTTGTTTCGTGAATAGTATGAGTATCTATAG
CTGTAAAGTGCTTGAATATCGCAAAGCTGAAACTAAGCCCTAAAAGTACCCACAAGGCAATCACAACCTTTTTAT
GAGGATTGACCTTATAGTAGACACGAGGTTTCTTTTCCTTTGGTATCTGTTTTTCTTTATTCTGATTTTTTCTAA
ATTTCATCATTAAATCTTCCTTTCTCATTGTTTGATTCGTCCTGCTCCCACTAAATGCTGTTGCCAGTAGGGGCT
TGTTAAGTCGGCATAACCGATTGGGTCGCCTGCATGAAACATACGGTTATTGCCAAGGTATATCCCAACATGAGT
AATATAAGAGCCAGCGTTATAGGTAGAATGAAAGAAAACCAAATCGCCAGCTTGTGCTTCCGATAGTGGGATATG
CTGGGTCACATCATATTGCTGTTGTGCGGTTCGTGGTAAGTTAATTCCAGCTTTTCCATACGTCCATTGTGTCAG
TCCGCTACAATCAAAAGAAGTAGTCGGGGAAGCTCCACCGTAAACGTATCGCCAGCCCTCATATTTCAGTGCTTC
GTCCATGATGGCTTGTACCGTATCATCATCAAACTCTGTTGTGACAAGATACTGCGTTACCAGTTGCACATAAAA
CATATTGCCATAGTTGTATCGCCAGCCCCCATTGATAGGTATGGCTATGGGATTGGGGTAAGACACTTTTTCGCC
ACCTGAATACTCTTTTGAGAAACTTTGAGCCAGTTCAAAGGTATATTTATTTCCACGATTAGCCACATACCCTAA
GAAACCACCACCATAATTGTAGGACTGGATAACCGATTCTAAATCTACACTGAGCCTTTCGCTACTGGCTAATAA
TTCACTGAAATACTTCACACCTTGCTTAATGGATTCTTCTGTACTCAATGAATTAGGTGGAAGACCGAGGGATTC
CGAGGACTGCATAACATCTTCCGCAGTACCGCCCGATTCCACCTGTATAATCGCAAGAAGTATGTTGACATATTC
TTCAACGCCATATTCTTTGGCATATTTTTCTACCATAGGCTTATGAGCCAGCACTTCTGCGGAAACATTCACACC
TCCATAATGAATATTGGAAATTCCGCTGTCCTGTTCATCTGAAAATAAAATGGCAACAAACAGAAGCAGTGAGAA
GACCATCAAGAATAATCCAGAACCACCAATCACTAAAGTTTTCAACTTCATGGTTTCTTACCGACTTTCTTAATG
GTGGCGGTTTTGATTGGTGGTCTACTTCTTGTATTTTGTAGTGGTACTCTTTGAACAGTAGACGGACGTTCTTTT
GTGATTGGACGTTGTGAAGTTCTATCTGCTGTAGTGGTTGAAGTTGCTGGCTTTTGAACGGTTTTTTCTTGAACG
GTATTGCCTTGGCGTTCCACTTTTGGACTTGAAAAATCGGACTTAACTGCTGGACGCTCTTGTTTGGCTTGTTGA
GATTCCTTATATGAAGTCTGAATATTAGACTGTTTTGAGGTCTGTTCATCATGATATTGTTCTTGTCTTGTAGTC
GGTCTTTCATGAACAGAAGAAGCAGGCTGTTTTTTCTGTTTGACCTGTTCCATTTCAGAGCGACGCTTCGCAATG
GTTTTTCGCCTTTGTTCCTGCTGTTCCTTGCGTCCACTGGCTCTGTCCGCTTTGGTTTGAGAAATACTACTGGTT
AAATCACGGACATTCTCTTTTACTTTGGATTTTCCTTGATATACTGCATATCTTGCATTGGTCGGCAAATCTTTA
ACCTGTTCTTTCAAACCACTAGCAGTGTCTACCATTCTGTCTTTGGTATCAGCTACTGTACCGATGGTTTGACCG
ATACGTTTTCCAAGTGTTGATTTTTCCTTTCCGTCTGGTCGGGAGTGATCTGCTTGTGTCCTTGCAGAACTCCCC
GAACCCGACTGTCCTTTTTTACCTGTAACAATGGCAGACCCAGCCCCTAGAGTAGTCATGGAACGTCCAAGTTTC
CGCTGTAGACGGTGCATGTGAGCGTGCATAAGCATACGAGGTTTTCTCATCACACGACTTCCCACACTTTGAGAA
TCGTTACTCTGTAGAGAAAACATACTCATTAAATCGCCCAGCTTGAAGTAGATTCCTGCAAAGGTCACAATCTGT
AGAAAAGCAATCAAAAAGAACGGATAACCAGCCGATAAGGTATAGAGCATGGTTGAAATACTAAATGCTGTCGTA
ATAATCAATGTGATTCCAGCTCGTGTCAAAATGGTATTAAAGAGCTTTGTTATGGCTCGTTTTGACATACCATCA
AATGATGGAATCATGCTTAAAATAAAGCTCACAGGCAGAAACATAGCATAGATGATAAAAAGTACCTGCGAGAAA
ATCATGATTCCTGTTAATAGGAATACAAATATGGAAATCCCAATATTGAAGACAAATAGGAAGAAGACTGTACCT
AAACGGTTAATGGTCTTTGTAATGGTTAGATTGGTATTGCTTCTGTCTTCAATTTCTTCCGCAACAATTTTTTCT
CTGTCTTCGCCATTGTTGGAATCTGGGCTGGTGGAGAGCAGGCTTTCCACACGGTCAATACCGATACTTTCAATG
TCTGAACTGTTGTATTGAAGCAGTAGCCACGGTTGCTGAACCTGTATGGAAAACAGGCTATCTCTGATTAAGTCC
ACGCTGTCCTTGCCTTGACTATCGGAATGGGGCATGACAATCTTCGTGCCAAGTGATAAACTGGCATTACTGATG
TCTGATGAAAAGTCATTGATTTTTTTAATGTAGTCGGGAGCGTAGGCAATAAAGGAAGCCGATAGGATAAACACC
AGCACAAAATTCATAATGGCATGAATTGCCTTTGTGGTTTCTCTCTTTATCAGTCCCGTATAGGCAACATAAACC

CCAAGAACCAAAATCAAGAGTAAGAGGAATCCAACATAGAAACCCTCTGTTGAAAATCCGTTTGCACTCACACCA
GCTAAGGTCTGCATATTCTTACCAATGGAATCTGCTGTAGCGGAAATGAAGTCTAAGGAATAGGCTTCCTGTACT
AAGTAACCTGTCGCATTGGAAACATACAAACTGATTGTCCAAATAAAATTGGTAATGGCATATAGTCCATACATG
ACCTGTTTTCCAATCCCGTCCGACCAGTTCCACGGAAGCCAGCCCCAGCTATTATCCACATAAAAATCCAGTTGA
TAGTTTTCAAGTGGGTATCGGCTGTATTCATTTGCCACATTGACCGTATCATCTACCAAGCCCGCAGCTTGAACC
ACCGTTCCCAGCATGGCTAAAAGAAAAATGGCAATCACAAGTGTGAAAGCCACTGTCATTGCCACTTTACCTAGA
CGTTTCAGCGTCCAGTTTGATTTTATTCTGTTTACTATTGATGGTTTCACATTTACACCTCTTTTCGCACAGGTG
GTCTGGTATCAAAGGCATGGAGCAGTTCTTCAAATACAGGGTGGAACTGTATCACACCGACACGACCATATAAAT
CACTGATAAGGCATTGCCCGTTTTCCAAATCACGCAATCGCTTCTGATTGTTTTCGTCCTCTGGGTCTACACCAA
AAAAGGCTAAGGTCTTTTTAATCTCGTTAAGGTCAGTGGAACGAAATGCAAATTTTAAGCCGAGGTTATTTTTCA
GTTTTTCATCTAAGAGGTCGTCTGTATTTTGGGTCACGAAATATACCCCAGCGTTCATAGCACGACCAGCCCGAA
CCAGCTTCATAGATAGTGTTTTTCCTTGTGCTACCTGTAAAAAGCTCCATGCTTCGTCTAAATCTACAATCTTGA
AAATGCTTCGGTCTGTATGGATAAAGTCTAAAGCAAAGGTACTAATGACAATCAGCATAGCAACGGATAAAAGCT
CCATAGTGGTATATTCCTCAAAGGAAGTTTCCTTGTCGGGAAGTACCAAGTCCGCAACCTGTATAATGTTCAGTT
GTTTTTCTAAGCTGATAGACTGCTCCACATAACCATTACTGAATAATAAATGTGCAAAGTCATAGTCTGTAAAAC
TTTCGATATGGTCGGCTATACTGGTACTTAGTGGCGTATTCTCAACCCGTAATTCCTCAATCACTTTCATCAACC
CTCGTACTTCACTATTGGTTACTGCACGAATGGCTTTTCTAAGGATTGGGAAGCGTTCCCCATCACGAGAGGAAA
TCCCCGTAAGGAATGTCAGAATATCAATAGCCAGTGATTCAGAATCTTTGGGATTTTTCATAATCACATAAGGGT
CAAGTAAGCCTTTGTTTTTCTCATCAGAAGTCAGAGTGACGATATTGATTTCATGGGAAATCTCTGGCAAGGTTT
CTTTCCATCTGCCACGTTCTGCTTTTGGGTCTACAATCACTGCTTGTGCCCCATAAAGCACCGCATAATAGACGA
TAAGGTTATTCGCAAAGGATTTACCACCACCCAGCGAACCAACAAAAGCCGACGCTAACGCATTGGTTACTGAAC
CCTTAACCCCTTGACTGGCAAGAGCAGGTTTCAGATAGACATTGCGTCCAGTATCTAAGCTGTAGCCAACATAAA
TCCCCTCATTTTCCCCCAGCATTTGAGTAGCACCAAAACCTAAACCAGCGAGGAAATCAGAGGTCACGTATTGAA
TATAATCATTCATATAACGCTTGCTGGCAGGTAAAAATTCTTCATGTAAGCCGAGCATATCCCCAAATGGTCGTA
CCAGTTTTACGCTTAAATCGTCATAAAAATCTTTCACTTCATTACAACGACGTTTGAGTTCGTCAAGATCATTTG
CTGATACCCTTACCACATAAGACAGCTTGTACATAGATTCCTTGCTTTGGTCTAAATTGGTTTCCAGCTCATTCA
CACTTTCCAGAGCTTCCGCCACATTGGAGCTGGTTTCATTATCACTTTGCCAAGCGTGGTTATCCAAGTCTTTCA
GTTCTTTCTTTTTATTGCGGACAGTAGATAGGGCTTTACGATTCGCTACAATTTCCACATTCATTGACGTATCAA
TCGGGAATGTAAATTGCTGTTGCTGGTAGTAGAAGATTTCAGAGGACGGGAAGTCCAGTTCTCCGACAATGCTGT
TAATGGTAAAGTAAGCTACATAGACGGTTTCATCTTCCTGCTGGATTTTCAAATATCGCTGTTTTTCTTCCACCA
AACAGCGAGTAGGCTTAATCAAGTCATAGTATTTAATCAGCGTTTCATTATCCAGCTTTTTCTTTGATAGATGGT
ACTCATACTCTTCATAGGCAGTGCCTGTCTGTCCGTAAAGGTGTTCAATCAGATAGCCGAAGTCGTCCTTATCTA
ACCTGCGGATTTTGAAACGACGAGAGATTTTATTTTCTAAGAGCTTTTCCATCTTCTGAAAACGCAGGATTTCAT
CATTACTCATACTAACAAAATCGCCCATCAGCTTATGGTTCACATCATAGACAAAATCAGACAAAGCATTTTTTG
CTTCAACGGTAAGACTTTTCATAGAAAACTCCTGATCGTTGAGAAGCAACTTAAAGCCGATAAAGAAACGGTAGT
TCACTTGATTTTCGCCAATCATGGATATTAAAGCGTCTGTCTGTTGGTCGATTTTGTCATAGGCAACCGCTTTGA
GCTTGCCAGTGACTTCATTTTTGGAACGCTCTTGTGCAGAACGTATGCTGGATTCTGTACTGATTTGTAAAGCAT
GAATTTTGCCATCACGATTTTGTGCGATAAGCTGTCTGAAAGAATCATGCACTTGTATTTTCTGTTCTGGACTTA
GAAATGAGTAATTGTAAGGAACAAGCTCATAGTAAGCATAACATTCCCCGTCTTTATTCCAGACGAGATTGTTTT
CAATGTATTTAATTGGATATGCCATAAAATTCACTCCTAACTGCTGTAATGGCTTCTTGTGGCTGGTTTCTGCCA
AGCGTTACTTTTTTTCCTGCATAGGTCAGCTTTGGTCGCAGTGCATAAGCAATGACAGACTTCAAAAATCCATAA
GGCTTTTTACCATCAAAAGTTTTTGTAGACATAAACCATGTGAAAGCCACAGGAATCCCAAAGTATTTGAGAAAT
GCTCCCTCTATCATGGAAAGAGGGGGCAAGTTGCCAAGTATCATCACTGCAAAGAGTGACACGACAAACCATGTC
ATTTGCGTAAAGGTTATGGGAAACGGAAGTCTAAAATCATTGATAGAATACAGTACCTTTTCCACAGACCAGATA
CTGGTATAGCTTCGTATTTTCTTCATGTAATCAATCCTTTCATAAAAAATAGGGGTAGCTGATTGAGCCACCCCG
TAAAATAGAAAATCTGCCAGTAGTAATGTACCGACAGATTTAATAGACGATTTCAAAAATCCCATGATTGGTTGA
GATAAACGTTCCTGAAAGGTCTAAATCCCGACCATAGGCTTGATAATCAATATAGTTTTGAAGACTAGCTGGTAC
TTCGCCTAAAGCACCCGTTTCTTCAATGTAGTAGCGTGCCACGTCATACATATCATCACAATCGGAATGAATGAT
AATATCCTCTTGATGTTCGCTTAGTTCTTCAATGCTTGAAAAATGAGTGAGCAGAGCAGATAGCTCCGATTGTAA
TTCTTCGGGTAATTCCGATACCATTTCCCATAGTCGATTGAGTTCGCCAATGGAAGTGTATTCGTCAACCGTAAA
GGGTAACTCGTAGTCATGAATGGCGTATTCCTCATATTCATCATTCAAGCCGATTTTCTCTTTGACTTCCTCAAA
GTCAATGGGAAAGGTAAACCACGCACCGACCAATTCGCCCTCATTGTATTTGCCTAAATTCGCAATATAGACTTG
CATATCGTCCATATATTCACGTCCTTTCTTTGTAGAGATTCAAAAATCCCTACCGCACTTCGTTTGGTGTACCAT
TCCTTTGCGGAACATAAGAAAACCACTTATATTCCACAAAAGAACGGTTTTATTTAAGCACCAATAATGCGATTG
AATAGCTCTAGTAAAATGTCTTTTACTCCAGCAGCGTTGAAGACTAAGCCAACCGCAATAATCGCAATAATTAAA
AAGCCAATCAGTTTGCTAAACTCACGCTTGAAGCCAAGATACAAGCCAATCACAACGATTGCTAAAAGCACCAGT
GATTGAGCGTTTGATAGAAACCAGTTATAAAGGTTTTGTCCAAAATTCATAAAAATGTTCTCCTCTCTATATTCA

ATGAATTTGTATTTGAGTTATTTTTTTGGGGTATACACTTTCTGGTATGGATAGAGATTCCATGCCAGATTTTTT
TATACAAAAAAGAGGACATTTGCTGTCCCCTCGTTATACAATCAATTCACCACAAAAATCATGAAAGAAGGTTAA
ACAAATGTCCCATAATGATTCTATCCTAAATATTCTTGGAATTAAAGATAAAAATATTAAAATTATTTCTGTTGA
AGAAGCTGAACACAACAACGATTCTGTTAAAGAGTATATAACGCTAATAACAGCTACTCTTTCTTATCCGATTAA
TCGTTGTCGTAACTGTGGCTTTCCCACAGTTAATAAGGATGGCTTTCGCAAAACTCATGTACGACTGGCAAGTTT
AAATGGGAGAAGATATGAACTAGAGCTTCGTAAACAACGCTATAAATGTAAATCATGCCATACTACTTTTGGTGC
TATTACTAATTTAACCAAAGAAAATCAAACCTTATCCAGTGATCTCAAAAATCAAATCATGCTTTTAGCTCGTAA
AGGCTTATCTGGTCAGCTTATTGCTGAAATGTGTCACTGCTCTCCTAGCAGTGTTCGTCGAACAATCTTAGAGCG
CATGGAACCACACTATCGTGTGGCTAAGTTGCCTAAGCATCTATGTTTTGACGAGTTTCGTTCAATTAAGTCTGT
GATGTCCTTTATCTGTTGTGACGCTGAAACCCACCAAATTGTCACAAAGTTACAGGATCGTCTATCACCTACCAT
TGTTGATTATTTTGAAAGTCGTTATTCAAAAGCCGAACGCGAATGCGTTCAATCAGTTGTAATTGATTTAAATGC
TCAATATCAAAGTTTTATCTATCGCCTTTTCCCTAATGCCAATATCATTATTGATCGCTTCCACCTTGTACAATT
AGCTGGTCGCGCTTTGGACAATTGTCGTATCTCTATCCTAAAGCAACTTGATAAACAGAGCCAAGAATATAAAAT
TATGAAGTCACATTGGAAGCTATTCCATAAAAAAGCTGAAGATCTTCACCCTGAAGAAGTAGTTTTTCTTCGCGG
CGTTAAACAATATATGACTCGCCAAAATGCTGTTGATCTCATTACTAGTAAATTTTCCAAGTTCGCTGAAGTATA
CCAAACTTACCAAGATATCACGAAAGCCCTAAACGAGCGCAATAGTGAATTACTAGAGTCAACCATCTTAGACTA
CCAAAAAACCAATACAGAAATGGATACTGCTATTCAAACCCTTCGTCAAAACAGAAAATATGTCTTAAATAGCGC
TAAATTTGAATACTCTAATGGTCCTTTAGAAGGCATCAATCGCAAAATCAAAACCCTAAAACGAACTTGTTATGG
TTTTGCCAATCAAAAATTTTTCTTTTTAAGAATCGATTGTATTTTTTCGTAAAAAAATACCCCCTACATTTTCGT
AGGAAGTATTTTTAGTCAACCATACCAGTTGACAGATATCCCACTTAGGACATTTTCCTACAAGGGGTCCCGAGC
GCTTAGTGGGAATTTGTACCCCTTATCGATACAAATTCCCCGTAGGCGCTAGGGACCTCTTTAGCTTCTTGGAAG
CTGTCAGTAGTATATCTAATAATTTATCTCCATTCCCTTTAGTAACGTGTAACTTTCCAAATTTAAAAAAGCGAC
TCATAGAATTATTTCCTCCCGTTAAATAATAGATAACTATTAAAAATAGACAATACTTGCTCATAAGTAATGGTA
CTTAAATTGTTTACTTTGGCGTGTTTCATTGCTTGATGAAACTGATTTTTAGTAAACAGTTGACGATATTCTCGA
TTGACCCATTTTGAAACAAAGTACGTATATAGCTTCCAATATTTATCTGGAACATCTGTGGTATGGCGGGTAAGT
TTTATTAAGACACTGTTTACTTTTGGTTTAGGATGAAAGCATTCCGCTGGCAGCTTAAGCAATTGCTGAATCGAG
ACTTGAGTGTGCAAGAGCAACCCTAGTGTTCGGTGAATATCCAAGGTACGCTTGTAGAATCCTTCTTCAACAATC
AGATAGATGTCAGACGCACGGCTTTCAAAAACCACTTTTTTAATAATTTGTGTGCTTAAATGGTAAGGAATATTC
CCAACAATTTTATACCTCTGTTTGTTAGGGAATTGAAACTGTAGAATATCTTGGTGAATTAAAGTGACACGAGTA
TTCAGTTTTAATTTTTCTGACGATAAGTTGAATAGATGACTGTCTAATTCAATAGACGTTACCTGTTTACTTATT
TTAGCCAGTTTCGTCGTTAAATGCCCTTTACCTGTTCCAATTTCGTAAACGGTATCGGTTTCTTTTAAATTCAAT
TGTTTTATTATTTGGTTGAGTACTTTTTCACTCGTTAAAAAGTTTTGAGAATATTTTATATTTTTGTTCATGTAA
TCTCTCCTGAAGTGATTACATCTATAAACAAATACAGAAGTTAAACGATTTGTTTGTAATTTTAGTTATCTGTTT
AAAAAGTCATAAGATTAGTCACTGGTAGGAATTAATCTAACGTATTTATTTATCTGCGTAATCACTGTTTTTAGT
CTGTTTCAAAACAGTAGATGTTTTATCTACATTACGCATTTGGAATACCAACATGACGAATCCCTCCTTCTTAAT
TACAAATTTTTAGCATCTAATTTAACTTCAATTCCTATTATACAAAATTTTAAGATAATGCACTATCAACACACT
CTTAAGTTTGCTTCTAAGTCTTATTTCCATAACTTCTTTTACGTTTCCGCCATTCTTTGCTGTTTCGATTTTTAT
GATATGGTGCAAGTCAGCACGAACACGAACCGTCTTATCTCCCATTATATCTTTTTTTGGATATCTGTCAACTGG
TATGGTTGACAGATATCCTTTTTTTGTTGTTATCACGTCCTGTTCTTTTACTGACTGTTGCTTCAAAATCTGCTT
GTGTCGGTCTGTCAGTTTCGCATGGTCGAGAATGTCTTTTACAACCTGCGTCTGGTTGATTTCATCAAGTTTAAT
CGCAACCTTTAAGGTCGGGGCAACTTGATGAGATAGCCAGTTCAGCGTCCTTTGGAAGGAGTAAGGCTCTGGTTT
TGTGGTTAGTTTTAATCGTTCACGATTGTTCCCAATAAACCAAGCCCATTCTTCATTCAGTTTCCAATCAGAACG
AGGTTTGGAATCGTCTTTATCTACAAAACGGATATACCGATTGATAATTTTAAAGGCGGTATGCTCTGGATTGTC
ATAGACGAGTAAATCACGGACTGCATAATAGGCACGCTCATTTTTCAATCGAATCTCAAAACGGTTTTTTACTTC
TGCGTCTTCAATGGGAATATCATTTTTCTTGTACTGCTCGTAGTCCTTTTCATAGATACAGAAATAAACTTCACT
TTGTAATGAACCGATATAGAGGGTGTTTCCCATACATTCCTTTTCCTCTTTGCGTACCAGTTCGCCACTGCGATA
GCTTTTAAAACTGCGGAAGACGGAGATACATTCTTCCTGTTGGCACTTTTCAGTGAGTACAGGGATATTCAAAAT
CCCTGTCTTATCGTTAATGGCAAGGTCAAGGCGTTTCATCACACCGCCAGCCACCAAAACGTCCATAAAGAACTC
ATACCAGCTTCTTTGTTGTGCCAGAAGATAGCTTTCAAATTGTCTGCACCCACGACCTTTCAATTCCACCAGAAC
TCCTTTGTCCAGTTCATGGGAGCAAAGGACGAATATGTCGCCTAAAGCATAATGCTCTGAATAAGAATAGAAACC
ATAGTCCTCATGAAGAAAATAGGACAGTTTCAGTTGTAAGATGTTTTCGACCACCTGCTGTACGTCTGTTGTCGG
AAAGCGAATCCTTACATAATCAAACAGCATTTCAAGGGGAGCGTCGGGATTGAAGCGTTCCAGAGCTTCCCAAAG
GGACTGCTGTAAATCCTCTGATGGCTTGACTTTTCCTGTTTCAATATCGCTTAGATACTGCCTTGTAATACCAGT
CGCAACAGCTAAACGGTTTTGAGATAGTCCATAAGCCAAGCGTTTTTCTTTTAAATGCTGTAACCAAGTTTGTTC
ATTCAGTAAAAATCCCTCCAATCAAAAAGGCGTATGTCAACTTTTAAAGCCCATTTGACATACGCTGAAATTTTG
TAAATCCCTTGTAACCAAAGGATTTTCTAATGTTTTTTTGACTGTTTCCTGTCGATTTGTACCCCCCTGTTAGAT
ACGGGGGGTTAAGTGCTGGCGTGGCTATTGCCACACCAGCCAGCAAGATCAGTCCACACCTGCGACTTCCGCTTC

GCACGTCGCCTGCGTGGACTGTCTGCTGTTGGATAACTTTTTAATTTCCTCCAAGAAATCATATCCTTTTGGTAC
AAGGGGAGTATAAAACTCTGATATGACACTTGTTCCTACATCAACATAGCCACGACCTTTGATTCGCTTTAAGAA
GAAATCCTTTTGTACGTCACTGCCAAACATCATGCCATAGCCCATTTCAGACATACGACCTAAAGCCACTCTGAA
ATTAAACTGATCACGGATTCCGTCGCCTAAATATTTTGCGTCTGGACGTTGACAAGCCAGTATTAGAAAGAAGCC
AGCTTGACGACCTAACATGACAATCTGTTTCAGCTTATTCATAACTGCGGTGTTTTCTTTTGTTCCCAGCATTTC
CATGAAAGCGACGTATTCATCAAAGATTAAGAAGTGTGCCGGGAGACCTAAGTAAGCATAATTTTTGCCAGTCTT
ATAGTTCTTCATCTGCTTCATTTCCTCACTACGTTTCATCATTTCTTCATAGAATGTTTCAATGCAAGAAAGCAA
GTCTTCTTTTCTATAGTAGACATTTGCCATCACAGAACCTAAGTCCGCAAGGTCAGCATTTTTCGGGTCAAGAAT
ATACAGTTTTGAATCTGTATGAAGCAAGGCTTCAATCAGTGTCAGTATAAAGTAAGTTTTACCGCCACCTGTACC
ACCAGCAATCAACATATGAGGGAGCTTATCATATTCCCACCATACGTTTTTCATTAAGCGAAGTTTACCATCTTT
AGCTTCTACTTCATCAATAGAAATACGACTGGCTATGGTGTCATAGAGCAAAGTATATTCCACATAGGAATCCTT
TAACTCTTTATCCGTCAGCTCACAGTACAAGCCACTCTCTAATTTCTTTTCCAAGTGTAAGAGTTGGTCTTGATA
TTTTCCCAGCGTGATTTCCACCCGTATCTGTATCAAGCCATTTTTAAGTCGATAATACATTTTAGGGAAGTAGGT
TATCTTTTCCTTTGTACGACCAGCACTATCTTTAAAGAAACCCTCTGTTTTGACCTGTTCAGATTCATACCACTT
GTTTTCAAGTATCATCTTTGCCAGTTTTTGACGGTGGTAAAGTTGTTTAACCGTATCATAGCGAACCCGTTTGAA
TACAAACGCTACCAGCAAGCAGATAAGAATTGCGACACTGAAACTGATAATTAAATAGGGAATGTCAATCTTATC
TGCTTGTGATAGGTTAAAATCCTGCCAGTTGATCTGCTGGATTGTCTTCACATGAAACAGTCCGACAACCAGCAG
GAAAACAGGCAGGAGTGACGCTATCGTAAAATGAAAGACTAAATCTTTACCAGATGGGCGAATCCTTTTACCACG
CTGTTTCATGCGAAAAAGTCTCCTTTCTACCTAGCGACTATTTGTCTTGTGTCGGTTCTTTCTTTGCTTGTGGTT
GAGCTTTGAATGAACTAGAATCCTTTGTCAGCACAATATCGTCTGCCTTGATATACCAGTCAACATCTGCTCCTT
GATAGGTGGCAGTAGCAACGGTGTCCGCAATGGGATTGATAAGTTCCACCCGTGCGTTATAATCAAACTCTTTCA
AAGGCACGCTGGCAGGAATACTTACTTGAATCATGCGTCCTTGTCCTTTGGATTTTAAGTCATAGGTACGTTCCT
TGATTTCATCTGAAACCGACCCGTCTTCATTTTGGATTCTCACTTCACGACGTAGAGCAGAGAATTTCAATTCTC
CAAAAGTCGTGTCTTTATCTAATACAATGCCATTTGCTAATCTCATCATTTTTCCTCTCTTTCTTTATTCTTTTA
TCATGTCGTCAGCATGTAAAAGGTAATTTGTAAAACCACGAGTGCCGATTTTGTAGCCCTCTGCGGTAATACGTG
GATTGACTAACTTCACACGTTCCTCAAAGCCGAAATGTTTTTCGCCAGCTTCAGCAGGAAGCACCACCACAATAT
CATCTGCTCTTTGAACATCAGAATAGAGATTATAGCTTCTTGATAAGACAGTTAGCCGTCCGTTGATTCTTCGCT
GAACGACTTTATCCTCGCCAGCAAATTCTAAATTGCCGAATGTTTTTTCCATGTTGGGAATCACAAATTTAAGTT
CCATATTTTTACCTATCCTTTCTTTTTTATTGGCTGAATGAATGTTTGATGGTCTTAAAGAGTGGGGAACGACCT
TTTGATTCTTGATTTTTTGTTTTCATAAGTTCACTTCCTTTCAAAATCGGGTAAAAAAATAGACACCTCATTTTT
TGAAGTGTCTACCTATTAAATATTCAAATTTTATTGGAAGTATCTTTATATCTTCACTTTTCAAGGATAAATCGT
CGTATCAAAGCTCATTCATAAGTAGTAAATTAGTAGTAAATTGAGTGGTTTTGACCTTGATAAAGTGTGATAAGT
CCAGTTTTTATGCGGATAACTAGATTTTTATGCTATTTTTCTAATTAAAAATTAGATAAAACAGAAATACCATAT
AGTTAATTAGTATTTTCATCATTTAATCAACTAATCAGCCTTCCCAACATCAAGACCATTGGACTCCTTTTGTGA
TTGCACTCGAATCTCCAATTGTTTATTCTTCTGAATAAACTGTTGGTAAGGAATTGTATTACTAAACATAAAAAC
ACCTAGTCATGCAAATGTAACTAATTTTTTAATTCCACGTTTTTTTCTCATGCAATGAAACCCCTTTCTTTTTCC
TTGTTTTTATTCTAACATATGTAGTTTATTCAGTCAATTATTTTTATTGCATTTTGAACTTTTTTTTTTTTATTA
CTATAAAGAATTATTTATTCTTTTAACTTGCTATTTTGAACTAAAAATTTTATAATGAAATTAAGCAAATAGGAA
GGTTTATTATCTTTAATGAATACACTCGCAGAGAAATTCAGATTAAAAAGAAAAGAGCTAGGACTCTCCCAACAA
ACTCTTGCAGAAGGAATTTGTGAACAAAGCCAGATTAGTAAAATTGAGAGAGGGCATTTCATTCCCTCCGCAGAC
CTTTTGTTCAAACTCTCACAACGACTTGAAGTACCATTAGATTATTTTTTTAATGAACAAATTGAAATTAAATCT
AACCTCTCTAATTTCAAGCAATTATCTGCTCGACTATTAGATGACAGAAATTATGAAGATTTGGAATATATTTAT
AGAATAGAGATTGAACGAAGTACTTTTCTAACACTAGAAGACCGAACTTACCTTGAATGGATTAAAGCTATTATT
GACTTCTATCAATATGACAGTAAGTGTGAGGCTATTTCTTCATTGGAAAATATATTATTAAAAGTCTCCTCAAAT
ACTCTGATTTATTTAAAGGCATTGAATACTCTATCTAATTTCTATTCCTTAGTGGGTCGTGAACAAGAATATGAG
GCAAACTACTCTCATTTAATGGAGTTATATCAGACAAAAAATTTTGAGCATCAAGAGTTTTTATTTGGCTACATC
AGAGTTCGTTACAACTACTCTCACTACCTAGTGTCAAAGGAAAAATATAACGAAGCTATCCAAGAAGCTCTTGAG
ACGATTGAACTCTGTAAACAAAGACAGACAAGCTACCAACTGGCTCCCCTACTTATTCTTGTAGGAAATGCTGGA
GCCAAATTTCTAGACAAAGAACAAGTCAAAAATTATTATATAGAAGCAAGAGAGTTATGTAAGATTTATAACAAT
CCTTTAATGTTGATGAAGATAGAAAATTATTTGAAGGAATTAGATACTGTATAGTTAATCTTGACATTATAGTTT
TTCTGAAACGTTAAATAACCTGTAAGGCTGATAGTGATATTAATACTATCAGCCTTATAATTTTTCCATGGTATC
TCCAATAACTATACTGATTTTGTTTCCCGTTCTAATTTGGTAACATTTTGATTTCTTGTATTATCAAGTATATTG
ATAAAATTCTCTAATCTCCTAACCTTATATTCGTAATCATCTATCCATTTAGTCATAAATCTTTGATTTTCTTCG
ATTTCTTTCTCCACCTGCTCCAGTTTGATAGTTGAAGTCAGGTTCATCTTGGCAAAGAGATTGAAAAGAAAAATG
ATAAATAAGAGTAAACGATAAGATAAAACCATTGAGCAGTTGAATTTATCCAACAAATCAATAAAAAAACTAGTA
AGGAAATAAGTGAATAAATATAAGGAGATATTTGCTCAAAATGATGATTTTTTTGTATCGTATGTGATAACTAAA
TCTACGACTGATGCAATAAAAAATATCATAATTCCAAATATGTTTTATAAAAGATACACATTTTATATAATGGTT

GTCTGGGTTCCTATTTTTGAAAACATATCCCTTCTTCTTTTGAAATTTTCTTACTCAAACGATTGATAAACTTAT
TCACTTTAAATACGATATTCTCATATCTATTGCTCTCTAATTCTATCTGGATACCTAACTCATTCAATTCCTTCT
CAACAACATCAATGCCAATTTTATCAGTTATATTCAATTTTGAAAGATTCAATTTCATTTTTTGCTTATTTTCAA
CTGAAGCCAGTAAATATTCTGCTACTTGATTTAAGGTTGTCACTCGTTCTTGCAAAGCATTGATTCTCAATTCTG
AAGCTGCCAATTCATGAATTAAATCATCTTTAATTGTAATATAAGAGTGCTTATCCATATCCAGCTCAAGTAAAG
TATACACTTCCTCAATCTTCTCTTTAATCATATCGACTGTCGTTTTTCTGCGGAATGGAATCGTTTGATTTTCAG
AGCTGAACTGTCTAATCAAGGTTCGACCTTTCATGTAGCGATTCTTCTCAGGATCGTCTTTATGATAGACATAGT
AGGAGCTAGTTTCTCTAATAAAAACTTTCACCTTCTCCTCTTCAATATCGAGCTGCATATTCGGCACAAAGATAA
GCCCCTCCTGACGAATACCAAACTTGACCTTGATGTAAATACCATCATCTACTACTTTTTCAATTTGATTGAGTG
ACAACTCTACCTCAAACTCATGAACGGCATCTCTGTTGCTCTTGAACTCTTGATAGAATTCCCAGAACTTCTCAT
CTCTTGGGAGTTCTTTTTCTTTGGAAAATTTTCTTCTTGATAAAATTGAACCAAATCCTCAATTTCTGGATTTT
GCCAATCTTTTCTACTCTCAAAATAATCTTGGAAAAATTCTACACCATAAAGATTTTTCTGGTCTAGTTCTGTCT
CTTTCACCTCCACTCCTGCCAACTGGAAATACACATGCTTTTGTTTGGGAATGATAGTTAGATCAAAAAGTTTCG
CAGTTTCAATCAAGTCATCTATATTCTCAGCCTTTGATAAGAGAAATTCCATGAGTTCTTCCATTTCCCTTTTGG
CAAAGTAATTCTTAAAAAATTCTTCTGTATAAGGTTGCTTGCGATTGAGTTTATTGCCACGCACCACCTGTTTCA
TGGATGAGTCCGTCATAAAAAAGGTCGCATGCTTGTGGCTAAAATCCATTTCTACATGTAGTAGTGGAGCATTCT
TTTTGAAATCCTCAAAGTCTCTAGAATGTTCCATCAAAAAATAGAGTCGCTGCTTGAGTTCATACTTGTGATTGG
TCTTGCGATAAACTTCATACTGCTGGTGAGAATAGCGATTCTCTATAATTTTCGCCCCTGCAATTTTAGAAAATC
TATCTGAAATCATTCGAAGATTTCGCTCCACTTTATAATCCCACTTAAGTTTTTTGTCAGAATTGCTGTCAACCG
AGTTTATAATGATGTGATTATGCAGATGGTCTTTATCAACGTGGGTCGCAACGATAAAGCGAAACTTTCCACTAG
TCAATTCTTTCATGGTCTCATAACCAATCTGATTGATTTGTTCAGGAGTGAGATTGTCCTCTGGCGAAAAGGACT
GAATAATATGGTGAGCATGGATTTGTCGTTGTTTCTCCTCCAGCCTATCGTGTCGAAAATTGTAAAGCGTGTCAT
TGTTGATAAAATTTTCATGATACATCTGCACCATTTCCTCATAGCTAGGAAAGTCCAGAAAATTCCTCATATCAT
AATCCGACACTAAGGTAAGATTTTTTGTTTTATCAGGATTGAGAATATACTTGATGAGCTTTCTGCGATAACTCT
TTCCATGAATGGCAAAATGCTTAGTGATGACCATGGAACTCCCTCAGCTTTTGAGCTTGTATTGTAAATTCTTTC
TCAACTTCTTTTATCAATTCAGCGATTCCCTTTTTCAACTCTTGCAATTCTTCGCTAGAAATCAACTGAGATTGG
TTCACACTTCGAGCAATCTGATTGATGTTATTGCCAATCCTCTTCAACTCAAACACTAAGTCTTGGTAGCCATTT
GTATCAATGGTGATGAAATTCATTCCAGGGTCAAGTAGGGTGCGTCTCGCATATTCAGAAAAAGATTGGCAGCTA
CTTTGGTCGATATTTCTATTTAGTTGGTTCAACTCAATATCAGACAAAAACACTTTCTTGAGATTGATTCGGTAA
CGATGTTCCATGTGTCTACCTCATATATTTGTCTTGGAAGTCTTGACTGAGAGGAATGGAGTTGCCAACTTCTTT
AATCAATTCCTGTACGCAGGTTAAGAGAATAGATACATGTTCCTGGGTGACTTGACGTTCTGACTGGATCGAAAT
TAAAATCTCATGAACATCACGGCTGATTTGTTCTAGTTTTTGAGATTGCCAAAGGGTGAACCATATCTCTATCTG
TTTTTGTAAATCAGAGGAGAGTAAACTTTTACGGAGAAAATCTGAAAAATTAGTATCTCCCTGCTCTCTCATCAA
AGCTAAAATTTGCTTTTCCTCTTCATCTGTTAGGCGAAATTGTTTTCGAATATCACGCACGTCTCGTTTCATACT
ACTACTTCCTTTCCAAAATACCTGTACTGACAATGTCTGCTACCGTACAATGTCAGTACACTTTTACACCAACTG
ATTTTCATTCTTGTAAGGCTTTGCGAGCTCAGATATTGTGTCCACAATATCCCAAAAATCATATCGCCAGCTGAC
TAAAACCTTCCAGTTTTGACAGCTAACGATAAGATAACTTGGTGGCTCCGCCCCCAAACCCCCATAGAAAATCAA
AAATTGATTTTCTATGAATGATATTAGGATAACAGGGAAGATTTGGAAAAGATATCACAGCTAATATAGTTATGA
TTGACTTTCTCTGATTCTATGATAAAATTTCTGTAAACTAATATTTGGAGAAATAAAAATGCTGAGTCTAGATCA
AATACATTTACTATTGAATACTCCTGAAGATGAGTTTCATGATTTTAAACAAAAATGGCATCATTCTAAAACTGA
ATTGGTGCGTGATATCTTAAATTTTGTCAATACATCACATCATGAAGATTGTTATATCATCTTTGGAATTGATAA
TATCACTTTAGATATAATCGGTGTAAACAATGATGATAATAGAAGAAATGAAGAAGATCTAACAGATTTACTACA
TAAACTCTTTATATCAACAAATAATCAAATTAGAATTAGCATACAAACTGAAACTATAGACAACAAAGAAATTGA
CATCTTAATTATTCATGATACAGATAAAGTTCCTGTATTTTTAACAAAAGATTATAAGCCTAAGAAAGATACTGC
ATTACAAAAAGGATTAATATATGCTAGAAATGGCTCTATAAATACTCCTAAAGACTCCTCTGCTCCATTTGAGTT
AATAAATAAGTTGTTTCAAAAGTTTAATCATACCGACTTAAATATCAAAGAGCAGTATTTTCATGTTTTAAAAGA
CTATAAAAATTGGTTCTTCGTTGAAAATGAAGACGGAAGATTTTTTATTTATAATCCTAATCCCGATTTTTATAT
TAAACTTACCGACGATGATGCAAATCGTTTCAAAACTATGCCTTATAGTTTAAATCAATACCAAACCAATGTTGA
TTGGCAATTAGTACAACTTCGTTATCGACACCTTACAATTATCGACTTTATGGCTTTATATTTAGATCAGGGAAA
TTGTCTAGTGCCCTCCCCTGATTTAGAAGATTTTGAATGTGGTTATTCTGACACTATTTATTACCACTGTTTATA
CAAAAATACTTTAAAATACCAATTGCTGAAAGTTTTCTCTTCAATATCTGGTCTCGAAAAATATCCTTTAGATAG
ATTTAAAAATAATATAGTAATTTATGATGATAAAATAGAATTGGAAAAGACTCACAACTTAATAAAAAGTAATTT
TTCAACAGAAGAGATAATGAAGCAACTTGAAGTTACAGAAAAAGATTTCGATTTTTACTATAAAAAAGCTAGACA
AAAAAATCCTGATTATAGTATCCAAGAAAATCAAGTAAATCTAACTGAATTAAACTTAGTTAGGTTATTGAAAAG
TTTTCAAAAAAACATATCTTGATAACCCACTATAATCAATCTTTTTAACTAGTAAAAAAGATAAAACAAGGCATAG
AAATTAGTTTATCTAAAAAACTAATTTCTATGCCTTAATCATTATTAATTAATAACATCAACTAAATTGGATTGA
GTCAGCAATGAATATATCTCATTAATATTTCTATTATTTTGCGGACTATTATTTCTCAATTTACAATATGTGAAT

ATTAAGTGACTTTTTGCTCTTGAAAGTGCGACAAAGAATGCACTTTTATCTTCTTCCGGCTGATTATTAAAACTC
CAGAATGCAGAATCCTCCAAGCCTAGAAAATAGACTACCTCATATTCTAATCCTTTACTCTTATGGATAGTCATA
ATTGGAATTGAGTTTTCACCCTTGAAACTAGAGACTGTATCTAGCCATTCACCTTGTGTTTGAGAATATTCTATG
TATAATAACTTTGAAAAGTTCTTGACAATGATATCTAGGTCACTTTTTCCATTGTATGTTGAAAAATTGGAAATA
ATTCTTTTTTCATCTATTTTTTCAATAATACAATCAATTAATTTTAGCATACTTTCTTCATTGGGAATAAAATTT
GAAATTAGGTAAGTAATATCACTAACTATATTGTCAATCTCCTTATAGGATTTTGCTAAAATTAATTCATCAGTT
AACTCATCTATTCCATTGATATTTCCATAAAAATTACTAATATTTTCCCAAATTAAAGGATCTCGTTTTCCTTGA
CTGCATGATATTAAATCTAATAATAGGTTACACGTAGGATCTTTTAAAATATCCTGATATTCATTTTCAATTCTT
GCTTTAATCCCTTTGCTATTTAATATAGATATCAATTCGAAACTATAAATACCAACCTTTTGTTTTGCTAGGATA
CAAATTTCTGATGGTCGTATACCTCCTTGAATTTTTGATTCTATATCATTTGCAATTAACTTAGCTTCTAAACTT
TCATTTTCAAATTCAAATAATGTTATTTCACCCTCTTGAAATTCTGGATAATTATTTGTCTGAATAGAACTGTGA
TTACTATTTAATATCTGATGAACCTCTTTTTGAAATTCTACAAGCTTAGGTACAGAGCGGTGATTCATCAACAAT
TGATATTCATTTGGATTAAAGTCTCGAATATAGTCTGGAAAAATATCAGGCTTTGCACCTGCCCATCTCATAATG
GCTTGCTTATCATCTCCAACTGCTGTTAATTTACAAGACGAACCTAAAAAACAAGTTTTTAATAAATCATACTGG
GCATATGTAGTATCCTGAAATTCATCTAGAAATACAAAATCGTATGTCATTTGAAGTGCTTTACGGATGTATTCG
TTAGTATCTATTATTTGAGTACTCAACTTTGTTATTTGCCTATACAAGAGGACCGGCTTATTATCTTGTGTTCCT
TTTAGTAAGTCAGTTTTAAACTTGTGATTATCCCCATTGTTAAGTATGATGTTTTCGACATATCTTCTTATATCT
GACATTCTCCATCCATTAACATTAATTCCATTCATAGAGAGTAGTTCTTTTATAGTATACCAATCTTCAATCAAA
TAATCTCTTGATGGCCGTATATCCTCAGGTAGAACATCTCTAAATTGGTCTAAAATTCTCTTTTCAAAAGCCGAA
TAGGTTAATGAGGTAAAACGAGAAGCATATTCATCACCATAACGTTTCTTAACTCTTTCTTTCAAATTTGATGCA
GCATCCGTTTTGAAACTTAATGCTAGAATTTTTTTCGGAGAAACGCATTTATTTGTAGAGAATAGATAGTCTAAC
TTTTGAGCTAGTAATTCAGTTTTCCCAGCACCTGGTCCAGCAATGACTAAACAGTTAGTCACATCTTTGACGGCT
CCTAATGCAGTCTCTTCTAAAATGATATCTCCTTTAGGAAACCACTCCTCACTCTTTACCATGTTGAATATCTCC
AAGTAATTCTTCAGCACTGTTAACTATTTTTTCAAATACAGGGGGTAGATTTCTTGTTAATTCATCATCACTAAT
AGATGATAAAAATTGCATATGAGTTGTTGGTTTTCCTCTACCTAAAAAGAAATATTGATACCAAATCATTAATTC
TTTTTCTTCTTCATTGAAACTATCGCCATGTCCACTCTTATCTTTTAAAGTAGCTCTTATTGCTTCCTCTTTACG
TTTGTTGAAACCTTCTAACTGCAATCTATCAAGACAGTCTAAATCACTCAATTTCACTTTTTTACTACTTCCATC
CGAATCATTATATGAAACAACTGGTCCTTCTATTGGGGATAAAGTATTCAGATAATTGTCTTTATAATGTTGTAG
CATCAAGAAATCAATATCTAAAGGCGATGAAAAGTAGACATTAAATTCTTCGAGTTTATTAAACCAATTAATCAG
ACGTTGAGCTCCTATCGATTCACAACCTCGTGACCCAATTTCATTAAAATCAAGTCTCTGAGTATGGAACCATTC
TTGAAACTCTGTGTTCAGCTCATACAGATGTTGAGAAATGTATTTTATTCTTCCCCAACCACCTCCGTATCTCTC
ATTATCAAAATCCAACAAGGTAATATGAGGAATTCTGAGAGCATTCAATAATTTCCAAAAATAGTTGACATGTCT
GCCCCCTAAAGGGACAATTGAAATCTGAGAGCTATCTACCTCTTTCCCAAGTAAATCAAAAAACTTTGGTAATAG
CAGTTCCTCACTGTCGCCTTCACCTAGAACTACTAACTTCGCAAAATATAGTTCAGGATAAGCTTGAATTGCTCC
TTTAATATATTTATAAGATTCATCTATAGCTGGAGGTAGCTGTATATCAAAAACAATGGTTTGAAGAACTCTGTC
ATTATTTTCTATTCTTAAATACTTCAAATCTTCTGGGTCAATTCTTTTTACAATAGCTGGAGAATGTGACGTTAA
AATTACTTGGGAGTTGTCATTATTGCCTAATTGCTTAAACCTTTTTATCAGTTTTCCAATGTGATGTGGAGCAAT
ATGATTTTCTGGTTCTTCTATTGCTAGGATAGTTAGTACTGGTGGAATCAACTTAAATCTTGGATTATCAGGATT
TTCTTCACGGTCTTTGGTAATTTCTAACTCAATATCAAGTATCGAATCAACAAGTGAAAAATAAAAAATAGATCT
CAGTCCATCTCCTAGATCTGAAACTGTAAATGCTTCTTCAGTGGTTGTTGGTGAAAATTTTAAGGCAATCTGTCT
AAGTGCTGCCGCCATCTCAGAAGAATTAATAATCAACTCTGCTTGAGAAAAACGGTTATCTTCATGATATAATTC
CCATGACTTCTGAATCTCATTGTTGATCTGAGTCAATGCTCCATTTTCAGATAAAAAGGTGTTATTCAACTCATC
AATTTTATCTGTAATTTCATTTATCTCATCTTCGGTCCAATTTATACTATTTACCAACCTACTTAACATACTACC
TGAAGCATTACCCAACTCCTTCTCAGGCGTTCTTGAAGCAGGTACATAAAGTACCCTAATTTTATCCAAATCCTT
TCTAGGAGCACGATGTTTGTCTTCATCTCTAATAGTATCTTCATCAGATGAAATATAATAGAATTGAGTATCAAT
ACTTCCTTCAACTGTTCCATCATCTTCCCAAGAAGATTCTAACCTGATTCGTAGAAATGGTTTGGCACCATCTTT
AGAAACTGTAAAGTGTTCGAAAAACGATGGAATTGCTTGACTATAGGGAGTTCCATCAAGTTCATCAAACTCAAA
AATAGTTTCTATAAAGAGGTTTCTAGTATTTTCTCCGGGCCTTGAACCTTTTGGAAGGTGAAAATCACTTTTTTT
TATTATTCTATCATTTTGTTTGTCCGAAAACAGCTTACTCAATGCTTGTAATACAGTTGTTTTTCCCGAACTATT
ATTCCCAATCAGTACAGTTTGATTGTTTAATTCAATAATTTGACTTTCACCGAATGATCTAAAATTATTAATAAT
TACTTTTGTTAACTTCATTTCTTATCTCCATTACCTGAAACTAATTCACACAACCATTCCTGTCCCACCTTCTAC
ATCTCTTCCTCAACCCTACTCTATTACCCAAAAAGGCATTATTGAAGAGTGACAGAAGCTTAACCTTGATCTATT
TTTGAAACATAGACACATCTTCTATGAATTCCTAGCAAATAGTTGAAATAGTACTTGTTCCGTTTTTATAACAGC
TAAGGGCTATTGCGTAAAATTTATACAATTCGAGCAAATCTGTTTTTGTCAGGCCATGTGTCTTTCGTAGAATTT
TATATTGTTACCAATTATAGCATGCTCTTTTTACTTGATAACACTATTATAACATTTAAAAATAAAAACAGATTC
TGAACTAATAGAAAATATAATTCTAAAATTAGAATTTTTCTTGATAATCATATAAAATGCTACATTTCTAATAAA
CTTGTACTTATTTCCTTCAAACTACACAATTCTCGAGTTGTTGTAAGTTTGTAATTGACTCAAAAGTATTAGATA

TAATTTCAAAACAGTATTTTACTTTATAGTTGACCAATAACATTTAATTCTAACTAAAAAAACCATTATACAAAT
CAAGCTACTAAAAATGACTTTATGGTATACTAGTGATAATAAATTGTTATCGGTAGGAGATAGCGAATATGACAA
ATAAAAAAATAAGAGTATTTTTAAGCAGACCTAATCCATTTACAGAGAATCAAAATTTATTTATTAAAGAATTGA
TTAAATTTTTAAGACGACACAATATTGAATGTGTAACATTACAAGCTGCTGAGTATACTCCTTACGAAGTCATGA
ATTCGTTGAGTGAGATGATACAAAGGAGTTACGGAATTATAATTGTTGCATTTGGTCAGACATTTATTTCTAGTG
GAACCAGAAAAATGGGAGCTGAGGATAATCCAGACTTCTTTGCATCCAAAGAGACCCACTTAAAGAATAAGTGGG
TTACAAGTGTTTATTGTCATATTGAAGGGATTTTAGCGTTAACTTATAATCTTCCAATGCTATCTATTCCTCAAG
AAAGTTTAACTGAAGAAGGGATTTTAAAGAAGGGAGAATATTCTATAACATCACCTGAATTCTCATTAGAAACAA
AAGAAAATATATTAATGTACTTGCAATCAGAAGGGTTTCAAAAAAGTTTTCATGTATGGAAAAACCTTCTTGATG
ATAAGTATAACTTTATCAAAGGAGGGGAAATAAACTATTAATTACCATTTACAATTGCAGTTTTAATCCTCTGGA
GATCCTCACTTAACATAATACTACCTTCATTAATTGTTTTTTTCTGTCGTTCAGTTAAACCCTCAATATCATTAA
TAGGTTTTGAAAGAAGGCCTGAAATCAGCATTAGATCCTCTTGATTGGAAAGAAACCAAATGTCGTCATATTCAC
TAGATTTACGATGTATTTTTTCTAAAGATCCTAAAATAGTTTTTGCTGTATAAACAGATGTATAGTAGAACTCCT
TTAAAACTTCAACATCTTTCTCAACTAAGGCTATTTTTAACAGGTTAGCACAGTAATTCCTTGCACAATAAATAT
TTCTATCTTCAAAATTCATGCCTAACCTATAGTAATTAATTGCAGACATTAAGTCACTCCTATTCTTTGTAATAT
AAAATATACGTAAGAAAATAGCTCCATAAATACCTAATAACTTATGATATGTTGTGGTGTTTATATCTATATATT
TACTAATATTTTGCTGTGCCATTTTTAAATTTGCTAGCGAAGATTCATTTTTCTTATAGGATGATAGTAAATAGC
CTGCCAAAATTTCTTCATCAATATATCCACTTTCTAAAATTTGCTGAAATATTTCTTCTGCCTCAGGATACTTTT
CTGAATTCTTGAGTTCCGTGGCTTGATCATAAAGTTCAGCTATTGACTTGTCTAATGCTTCTTCACTTTGAATTT
CAGTTTTATCGATTAAATCGTTAATTATTACACGGTATAGAGCAGATTCAAAGGCTGGACTATCAGTAAATGTCT
CATCTGACTTTATAGCGGAATCAATATATTCAGAAAGCAGTTTTTTCACCTTGTTAACTTCATCCACTTCGTTTA
ACTTATCAGAATCATATCTGATTTGAGGTAAGTGTGCAATATCAAAAAAGCTAATCTTAGCTGTGTGATGATCAC
ACAGTATAATGGTTGATTTGGGTTTCATAGCATGACGTAGACCTAACTCATAGATAGCATTTTGGTTTAAAGTAG
TAATATCTGCAATTACAATATCTGCTTTTAATATACCTTCGATAAATGTTTTCGTTATACTCTGTGTACTGAAAA
CTTCATCAGCTCTAAAGTGGTTTTTACCATGAACAGAAACTAATTCTTTTTCTAAAATTGTTGGCTTAATTAGAT
TATTATAGACTTGATTCAAGTCCACTTCTATATTTGTATTAGGAATTTTTTTAGTATTAAACCCCATAACTACAA
AACAACTGGTCATTTCTCAACTTTCCTCCCGATAACAATTTATTATCACTATACAAAGCACTCAAATAGGTTTAT
AATAGCATGATTATTGATAAAGGAGATTTTTATGGATTATAAGCTTATTTCTACTTACTTAGATTATTGCAAAAC
TCATAAGCGTTTGAGTTCACACACGATTCGAGCTTATAAGAATGATCTTATGCAATTTTATAATTCAAACTATGA
TAATGTCGAATCCTATATCGAAGAGTTGACACAATCTAACATAAAAACGAATACATTAAGAAGAAAAATTGCTTG
TATAAAGGTGTTTTATAACTATCTAAAATACCAGCACATAATTGAAGAGAATCCCTTCAATCAATTACGCTTTCA
ATTTAGAACTGAAAAAATATTGCCTAAAACGATTCCGTATGATATTCTGAAAAATATTTTTTCATATTTAGAGCA
GAGAGTAGTTATATCTAAAACTGACTATCAAAAACAAAAAGCTGAAAGAAATCTACTAATTATTTCCCTATTACT
TTCAACAGGTATCAGAATTTCTGAACTTTGTCACATTCATCTCAAAGATATTAATCTTTCCAATAAGACACTCCA
TATTATAGGAAAAGGTAAGAAAGAGCGTATCCTATTTTTAGGAGATCAAACAACCTTCAATTTATTAGAAACATA
TATAAATAAAAACGGAAAGGAATCTAATGATTTCCTATTCCCAGGAAAACATTCGCCTAAACCATTGTCAGAGCA
AAGTGTACGTTTAATTTTAAAGAGAATCGTTGAACAAAATGGCTTATCTAAAACTATTACACCACATATGTTTAG
ACATAGCTTTGCAACAATGCTTCTAGATAATGATGTAGATATTCGATATATTCAACAAATTCTTGGACACAGTTC
TATATCAATCACACAAATCTATACTCACGTATCTCATTCAAAACAAAAAGAAATACTTAGTTCTTTCAATCCTAT
GTCAGTGATTCATTCTGAAATCGAGTAAGAGACATTTCCGAAGGTTACAGTTTACTTCATCTTAAATTAATGTTT
GTAGTTGACTTTAGTTAATAGATTTTATACTCAACAAAAGCAAAAAGCATGCAAAACTCCACTTTGAATCAGCAT
TTATACTATATTCCGTATTAAGAACTCCATAATTGTTTTTCTAAAATATAACTTTCAAGTATCCTTAAATATAAT
GAAAAGAGATGGGCAAAATCTCAATTCCTGAAGAAAATGGAGTAAATCTTCCCACAAGAAAACGCATAATTTCAA
GTTTTTCAACACCTGAGACTATGCGTTTTTTGCTTCTAAAAAAGTTTCGCCCACCTTAAACAACTAACAAAAGTT
ATAAGTAGACTATATTTTAAAACTCAAGATCTAACAACCATTTCGATTAGTTAAATCTTTCAATTTTCCTTCTCC
TGATTTAAGCATCTCTTTCTCAACTTGATTCCATTCGCCAAATAGTAAATCATGAAGAACGGTTGCTACTGAAGT
TTTATCTTCTTCTGAATCGTATACACAACTTCTATCTCGTTGGTAAATTTGAATTCTTTCAAAGATAGCTAGTTT
TTCCAATTGTCTTGTATTATCAACTAGATGATTTACAATGAAATCATGATGTTCTTTTGGAGTTGCGCGTGCTTG
ATCTGGATTGATAGCATACAGTTTTTCATAACGGATAAGGGTGCTCAGATAGGACAGCTTAGGCTTTGTCGCAAT
CAAAGCTAATTGTACTTCATATCCCATACTTTTCAAGAGTTGTGCTGTTTCCTTTGGAACATCAACGCCCTTTAA
CTACCCTATTCTAACGCCAAATTGGAAGCGACTAATAATCTCATTAAACTCATCAAACGAAATGCCTTTGGTTTT
CGAAACTTTGAAAACTTCAAAAAACGGATTTTTATCGCTCTGAACATCAAAAAAGAAAGGACGAAATGTGTCCTT
TCAAGATCTTAGCTTTTCTTCAACCCACTGCAGTTGACAAAGAGCCGTAACATTTAGAAAAAAGAAATGCACCAT
TTTTGGTGCGTTACTTCTTGTTTTTCTAGCCTAAACTTTACTTCTAAAAAAGCCACCTTAAAGAGTAATTCCTTT
TTCGGTGGCTTTTTAAAATACTATACATTAAAATACTCTTGTTTAATTTTTGTAAGCAGATTGTTAGCAACCGAA
GTTACACCAACATGCATCAGTTCAGGATCAGAATAATCTAGATTACGAGCATAGCCTTCTGCAACAGTCTGAGCA
ATACGCTTATCCAATTTATAATTAGAGGCTTTTAATAGTGCTTGAAATAATTCTGAACTATATAGTTTAATATTA

TTTTCCAATGATACTACTGCCATTTTTGTAACTCCTCTCCTGTTTTTGATTGATAAACCTTCTCTATTTTCATTA
TATCACAAAAAGATTGGTTGTGGATGGATAATTGTTTAAAAGAATATGGTTTTCTAAACTCAGCAAGCATTTCTT
CTTTTGTAATTTCATTCTGACGAAAACGAGCTAAAGCTTGGGTTGGATTGTCATCAGTCTGAACACCATAGATAA
AATCAAAATGGTGTTGTAATTCATCTGGATTTTCCCGATTTTGAACGACAAATTCAGCAAAATCTAAATCTGACT
CTGACTTTTTATGTTTTGCAAAATAGTGACAATGATAGTCAGGGTTCTTAAAAATTTCTATAAAATTTGAAATAC
GAAATTCTACTATAATTCCTACTTCTTCTTCTGAGTCAAAAATATTATTATTAGAGCTGCTTCGATTTAATACTT
CAACTTGCTTGTTAATAAATTTTCTAGCTTGTTCAAAGTCAGGCGTGATATAGAAACCAGGACCAAAATCTAGCT
CACTTCCTAAATTAAATTTAACATCTATACCACTTTTTAAGGATTCTAAATGTCTCAATAATGTAGCATGAAACC
ACTGCGTCTGACCTAGTTGCTTTTCCTGCTTGGTAGTCAACTCCAAAATATTTACTTCCTTCTCTAGTCTATACT
AAAAGTATAACACAAGACTTTATTCAAGTAAAAGAAAATTGTATAAAACTATTCACTTCCTTCCGTCTTTCCTAA
ATCTCTCTAAATTCTTCTCCAATTGTTCCTTATGAAGTTGATTCTTAGCTTCCTTTAATGTGTCGTCCAAAGTTT
CTTCCCTATAAATTGATTCTGTTTTTACTTGTTCTGATTCTTGATCTGGAGTGAAAATGATATCTAACAGTCGGT
CTATTTTTTCAAGATCTTGGACAGACATATCAGATAGTCTATGAATCAGCTTTTTAAATACATCACTATTGTCGT
GATTTTTCTTAAAAAATGTTGTAAACATCTATAACCTCTCAAAAAAAGCAGCCTATCAGGACTGCTTAGTGTAAA
TCCGAAATCGCATCATAAATGGACTGTAATTTCTTATTGTCTTTATTCTTTGTGTCAATAAATGTATGTAGCTCT
TCAATTTTCTTTTGACTGCTTTGAATATCGCTATTGTTATCCGCAATAAGTCTTTTAAGATGTTGCTGATAACTT
GTAGTGATATCTGTCTCTTTTCCTGTTCTAACCTCTGTAACTTTGGGTTTAGAACTAGAATTTGAATCCGTCACA
GGTGCTTGTTTTCGCTTTTGAAAAGCTGCTTCATAATTTACATCATTACCTCCTTGATGATTTCTAAATAAGGCC
GCAATTTTATCTGGATCTTCTTGATTTTCTGATTTACTTTCTAATGGTTGATTAAAATTCCAATCTTTTGTCATT
CACTCATTTCATCCTTTCTAAATTCAGAATCATCGAATTGTCTTTCTTTACCAAAGGCATGGTCAAGAGCTTCTT
CAAAACTCATGTGACTAATGCTTTGACGCCTTTTGAACGTCGCAAACATCGCTGTCTCCAGTTGTTCTTTGTAAG
CAGCGAGTTTTTCTGTCTCTCTTGCTACCTTACTTTCTTGCCTAGTGACCTTTTCTTCTAAGCGTTCAATAATGG
TCATATACGATCCTCCTTCATTCTAATATTAGCTAAAACATCTTGATTTTGTTATCACAATTCTAAACATTGAGA
GGTTTTTCTTACTGGTTGATTCCTCAATGTCTCTTTTGCATTCTCGATCATCAATTGTAAATCTGATTTCTTTTC
TCTAAGGACATCATTCCAGTCTACTTTTTCTTTCCCAGATTTATTATCAGGGAAATCCAGAAAAACAGGAAATCC
TGATTGAGATAACTTATCAGAAAAATCTTTTCCTGCATCATCACAATCTACCGCAAGTGTCAATAAATCAGGATG
ATTATCAAAATAGCTGGTGGTATCACGAATTGTATTGATTAAAGGCAATAACTTTGAAGGTATTACTGTATCCAA
AAATTCCAACCTCTGATTTTCTTCAGCTATCAGTCGTAAAGTTTGATAAGCAACAACAGACCTTTTTAATCCTTC
CATAGATACCAAACGAACATCAGTTAGACTTTGTTGATGAAGTTCGTAATAGCTCATTAAGTCGATGAACGATTC
ACAAAAGACCAGTCTATTAGGTTTACCAATATCAAAGGATATTCCAACATGTCCATGGCTTCCTTTTAGAATCGT
TTTTAACCTCTCTCTAGGAAGAGAGTGATTCTTATAAATCCCTTGGAGACTTGCTGCCTGCAGCTTGTGACGATG
ATTAAAGCTTTTAAAAACAATAACAGGTTCAACTGTTTCATTTGTTTTCCAACTAGCTTGTACTATCAAACCTTG
TTGAATCATCTTTTGTACGATTTCTTCTGAAATTCCTCTACATTCTGTTAAGTAATATCTGGCCAGACTACAGTG
AGAATCTTCTATTCTCTTTAAAGGATAATAAAATGGTCTCTCTCTTTTTTCTTGAACAGTTTCTTTTTGAAAAGG
TTCTTCAGAAAGAAAGGCTAGAGCTTCTTTAAAGGAAATTCCCTTAACAAGTCGAACAAAATCAATGACATCACC
TTGAATATCTCTTGAAAACCATTTAAAAGTATTGGTAGTTGAAAAAATCCGAAATGAATCGTGTTCAGGATGTTC
ATAGACACTGCTCGAAACTTGTTTAAAGGAGATACCTAAACGACTTGCTACATCAAGAATTGAAATTTTCTTACA
TTCTTCTATTTCCATGCAATATCATCATTTTGTCGTAGTATTTGGCAGTGATGGAACAGATGAGGATTCTTCTAA
AGTTTTAGGAGTTGCCTTATCTAAATCATCTAACCCAGATTTCCAAACCTGTACTTGGTTGACCAATAACTTACC
AGGTAGTTTAGAATAGGATAACTTAACTGTTCTTGTTTCTGTCTGATTCGTCTTTGATTGGTTGGCATTCTTTAA
ATCAGATACATAGGTTACATTATAAGAGACCATGGCAATAGCTTGATTCGTAGTCTGATTGACAAAGATATCAGC
TTTTTCGAAATGATAATCTAAAATATAATCCTTATACACTTGGTTCATGGCATCATTTTGACTTGACAATTCTTG
AGAATAAGCCGATTCAGTCATATAAGGTTGAATACGTGTATTATTTTCTCCGAGCTTTTCTTTCGTATAGTACTG
TGTCAAAAATTCTTTTACAGTATCTGATAACAAAATACTTGCTTTATCTTCTGCTTGTTTGTCCTCTACAAGTTT
AGCTGCAGCCAATTCAATCTCTTTACGACTTTGTTTAGCAGTAGAATGTTGACCAGCAGTATATCCCATCATGAG
AATAAAGCTAGTTGCAGCCACTGCTCCAACACTAATTAAGGCTTTAGTTTTGACTTTATTTAACATCTTAGACCT
TCTTTCTATAAAAACTAGGTCAAGAATAACAAAAAGAACTTGTAAGTTTTATCACATCACAACGAAAGGTAATTC
CTACATTTCAGGCACAAAATCGTACAGTTCAGAGAAAAAAAAAAACTAGCTGTCTTTGACAGTATAATAGACTTA
TTGGAAAAAAATAAAATGCATACTGTACTATGCTATACTATAGTTAAGAAAACTATTTACAAAAGATCATGCAAC
TAACTTCTGAGAACTTTAAATTATTTATTAGTGTAAAAAAGGACACCTTCCCAACGAAGATATCCTGTCTAAATA
AAGCTTATAAAACCAAACACTATAAAGGAGGACGTTCAAAAACGCTAAGTCTATAAAAACAGTTGATGCTAAACT
TGCGTTATTTATTCTATTGAACAACTCTACGTCTCCTAGCTTTTGATTTTGGTCTTGGATTAGCGACAGTCACCG
AACGATAATATTGGTAGAAGATACTCTTCGTTCTTCAGGTATATTTGCCTTAGACAACTTAAAAGCAGCAAGTGT
CACTATAGCTATTGATGTCATAGAAAGTCCTATTTCAATGTCCTAAAGAAACCAAAGTAAGAATTATCCTGGAAA
AAGATATACACCATAAAGACACAATCATACTCGATTTAACGACACTTCGTGCCTGTCAATTTTTTTTTGAGATG
GTCATACACATGATTTTACTCTTTTTAAAGAAAGTATAGGTCAAGTTTGTCAGAGCAATCACTTGTTTTTGTTAT
TTAGATACTGTGAATATTCATCAGATATCTTTATTCCTATAAAAAGTTCAAAAAGGCATGAATTGACTGTAGAGG

ATAAGAAATTTAATAGAGATAGTTATAATACGTATTAAAATTGAGCGTTTAAATGCTAAGTTTAAAACTTTTCAA
TTTGTGGAATCATCAACTATGAGTTAAAATAATTTACGAACAGAGGCTGTCCAAAAAACTTGATATCATACGTTT
TATTGTGGAAATATTCATTTTTTCTCCTGAAATTGAGTTTTTTCCCACACTCATTAATGTTATTGATATAATCCA
ATAATAATGATAATATCAAATAGTAATAAAAAAATTTGATAGTATTTTTTCATAATCGGTCACATTTTCTAATGA
TTGTTTATGGTAAATGGAGATAAGTATGATTTTAGGTGAAACTTATAGGAAAATAAGAGAAGAAAAGGGGATTTC
TATTTCTTCATTAGCAGGTGCTGAAATTTCAAAATCTCAAATATCTAGGTTTGAATTAGGAGAAACAGAGATTTC
TGTCTTTAAGTTATTGTATCTTCTTGAAAGAATAGGAGTAACACTAGAAGAATTTCTGCTTATCTGTAATCATTA
TCAACCTTCGGATTTTAATACCTTAATAGCTTCTGTTAAACAGGCCGCATATAATGAAGATACTCAAACATTACT
AGATATGGTAGAGAAAGAAATGGAACTTTTTCGTGGGACAAATTCTCATTACCATAAATTAAATGCTATTTTCAT
TGAAAGTATTGTTTCGGGAATGGATAAGAATCATCAATTGAGTCGTCAGGACGCCTCTTATCTTACAAATTATTT
ATTTTCTGTTGAAAACTGGGGTTATTACGAAACGCTCATCCTTGGAAATTGCTGTCGTGTTCTATCTCCAGATTT
ATTATTTAGATATACCAAAGAGGCGCTCAAAAAAGGGAAGTTGTATAGTCCTATACCTAGAAATAGACAATCTCT
CATTCAACTACTTCTCAATTCTTTGATTATAATGATTGAGAACGACTTATATGAGGAATCTTTATTTTTAAAACA
GGCTACGAAGAATATATTAGCGGATTCCACAGATTTTTTTGAACAAACTATTTTGCTATATTTAGATGGATATTT
GGAACTGAAATTCTATCATAATCAAAAATCACTTTTAAAAATCAAAGAAGCTTTAAAAATTTTCGAACTCTTCAA
TAAAACAATCTACAAAAATTACAAACAATACTTTGAGAAACACATCATTCATTTAGTAGACTAGGCCATTTATCT
ATAAGGTAATAACGTGATCAAACTCTTCCAATATTTCTGGATTTTGATCATGAGTAATGACAATAACAATTGCAT
CTTTAATGGATAGAATGTAGTCCATTATTTCATTTGCTTTTATTGGATCAAGGTTACTTGTAGGTTCGTCAAATA
TATAAACTGAATATGTTTTGACTAAAAACCTAGCTAAATCAATTCGTTGCTTTTCTCCTTCTGAAATGATTTTGT
TTGATTGAGCTAGGTTTTTACGAAGGAAGTCTTCATTAAAACCTAAGGATTTTCTTAATTTTTCAGTTAAATGAA
GGTTTCTAGCCAATTTGATATTCTCTAGAATATTGCCTTCAATTAGAAAGTCATTTTTTTGCACAAACGCAATAT
CTTTATATAACTCCTCTGAGGGAATATCAATATTCTGTTTTCCGTTGATTTTTATTTCTCCATCATAAAGATTTT
TTGGATAATAATTTAGTATTAATTTTACTAAAGTTGTTTTCCCAGTTCCTGAAGCACCGATAATCGCATAGCGCT
TGCCTTTTTTAAATTCATAGGAAAAATGATCAAATAATATTCTATTTTCAATTTCATAATATAATTGGCTAATAG
AAATAGTCGAAATGTTATCACAATCAATTGTACTTGTTTTATGGAAAGTTCCATGATTTGTCTCATATAGATTTT
CTTGGATATTATCAATAATGGACTTGCTACTGCTGATTAAATTTTTATTGTAAAGAATAGATTGTAGTGGAGCAA
AAACACCGTTTAATAATTGAATACTTGCTACTAGTAGTCCAATAGTCAGTAAGTTATTTCTTACAAAAAAGATAC
CAGCGACCATACAGGATAGTTGAGAGCCAAAACTTAGTAAAATTCCAGTAGTTGAAGCTAAGTCTTTTAAGAATT
GATATGTCTTTCTGGACTCTTCAAATTCTAAACTGATTCCCTGAATTTTTTCTTGGGTCCAGCTTTGAATATTTA
ATAACTTAATTTGTTCAAAACCATTAATAAAGTTTGTCATTTTTGACAAATAGTGACTATTTTGTAGGGAATAAT
TATTAGTTGCTTTAGTCATTAACTTTCCAGGAATTTGAGATAGAAAAATCGTTATACTCGACAATAAAAGAAAGA
CTAATGCTAATCGCCATTCTATATAGATAATGGCAACCACACTCATGATAAGGGTCCCTAAATTTGCAATTAGAG
AGATTCTAGGAATCAATAAATTTTCTTGAATAAGGTCACTATTTTTGGTGATATTATTTAAGAAATCTGAATGGT
TACGGTTGTCAATTTGGTTAAATTCACGCGATAAATAATGTAATAAGAGACCCTTTTTTAGATCTATAATAACTT
TTTTTACATATTGATTTTTAAGATAGGAATAAGAACAAGAGATAAAGAACCACAGTAAAATTAAACTTACACAAA
TAGTGATATGAAAGAGTAATCCACTATTTGAATTAGTTATACTATCAAGTATTCTTCCTAATTGAAGAGAGAAGA
ATGTCATTAAAAGAGCACTTGTCAAGCTTAATAAAATAATCAAGAAATATAGAAATTTATATTGTTTCATAATCT
GGTTCATATCTTACTCCTTAAGTAACTCTGATGAGAATATTATAATCCAAATATGAAAATCAGAAACAAACTTCC
CGCATTTGGGACGCAATGAATAATTTACACTAAATATAAAAAATTATAAAATAATTATTTTTTAAAATAAGAGGA
GAACTCATATTTTTATTTAACAATGAATATATCACACTAAACCAGATAAAATATACAAGTTAATTTGTACCGAAA
TTACTATATATTATAAGAAAAATAGAAACTCTTAAGCATTCTGTACACTTTAATTTTAATTCATCTCTTTTTGTT
TCTCACACTAAGTACTAAGACGGGATTATAAGCACATCAACAAAAAGACTTTATTCAAACTTACCTTTGAATAAA
GTCTTTTAACTATCTAAAAAGTTCACATTTTATGCTTATTTATGGAGTTAATGAAGCGCCAACTAAATCCTAGAA
CCAACATCAGATTGATGAAAAGGTACACAGCCGTAAAACTAGTTAAACCATATTGTCTGATATCATCAAAATATA
AACTAGGTTCAGTAAAGAACAGATAAATTCCATAGACCTCTACGAAAATAAGAAAACTGACAAAACCAATAAGGA
AAAGTCCACTGGCAATAAGAAAGAGTCTCCATAAAAGAATGATAATTCCTCCTACTGCTAAAAAAATTACTGGGA
TAAGGAGTAAATCATTCATGACTTTCTCCTTTCTAATCAATCAACTGACGATAATGGGTGGCAAGAGAGGAATCA
AACTCTTCTAATTTTTGAACTAAGTCCAAATACTCTTCTTTTTCATGTTCTTCAAAGTCCACACCTTGATGATTC
TGAAGAGCGATTTCCAATTGACTAGTGAGCTCTCGTTTAGAAAAACTGTAATCCCCTGTCACTTCCTCATAATGT
TCCTTTAACTCCTTGTAGGCTTGGTATTCTTCAGGTGTTTGAAATCCCTGTACCAAAGCCTCTTCTAATTGATAA
TAGGTATCTTCCATCATAATCGTTACCTCGTTTCTTTCTATCTTTATTCTACCGCGCTTTTCTTTTCTTGTCCGC
TATTTATATCTATTTTTTAATCTAATTCAAAAGAAAGTTGTTCTTGCTTTTCTTTTCCTTTTTCGTGGAATTGTC
GTAAGGCCTGATCAATAATATCTAAATCCGTTTCTGTTTCAATCAATGGCGCGAGTACATCGTATTCGGCCTTTT
TAGTTTGATAATCCTCTTCCTTTGGAAAATTTTTCTCAATTTCTACCTTAGCAGTAGTCCATTTATCCTTTAATT
CATCCAATAAGTTCTGAGTTTTCACTTGGTCATCTTTAATGTGGTCTATCGTATGCTGAAGCCTTTGAATTGTCC
CCAAAGGAGAATACAAATCTAAACTGACAGAATATTGGTTTTCTCCTACAATCTTAACAGAGAAGGTTTCAGGAA
GAGGTTGATTTGTTGCAAGACTAAGCATTTTAATGTCAAATCCTCGATAACTTGCTAGGGTTCGAAATTCTTTGC

TGTCAGATTGATTATGACGGATAAGACGGTGTAGGGATTCACCTGCTTCAGCTCGTTGCTCAAAAACTTGTTTAC
CTATCGTCATAGAAAATGATTGGTCTTTCGACATTTCAGACTGTTGAATGTCGCCTTCATACTTGCTTAATCGTT
TCTCAAGAATGGGCATATTTTCTTCACAGTAAGAGATTGTATGACGATAGTGATCCTTGCTGCGTTGAAAGGCGC
GTCTTTGATTTTCTAATAGAGTTAGATCATTCTCTAGTTCCATCTTATATTTGAGATAAGGATTACCTGTTGCTA
GTGCCTTAAAATCAGAAGCTGTCATAGTCTGTTCATCAATATCTTCCGCAGCACGAATCGGCTCCTTAGAAGTCA
TAATCTGCTTAATATAACGGAGTTTGTTCTCCTGAGTTGCCCATAGATAATTATCAAACGAACCTTTGGTAATAT
AGTGGTAAATATCCACTTCCTTGTTTTCATTTCCCTGTCGGATAATACGTCCATTACGTTGCTGAATGTCACTTG
GTCTCCACGGTACATCCAGATGGTGAACTGCTTTCATCTTGCTCTGAACATTTAAACCTGTTCCTCCTTTTTCAG
TTGAGGCAAGAAGAACCCGAACCTCTCCTGCATTAACCTTTCGAGACAAGCTATTCTTCTTTTCATCACTATTGG
CATCATGTACAAAGGCAATTTCCATACTAGGGATTCCTCTATCAACTAATAAAGCCTTAATCTCAGAATAAACAT
CAAAGCCATTATCTTTTTTCTTAGGTGTGCCAATATCTGAAAAAATCATCTGAGTAGCCTTATTTTCCATTCCCT
CACGATAAATTCTTTCAACATTATCCACTACCTGAAGCAGTTTATGATTGTCTGCTAGACTATAACTAGAGTCCA
ATAAACGCATATCAATAGCTAATTTTCGTGCCTCACCCGTAATTTTTAACATGTTATCCTGACTTGGATCAACTG
TTCCACATTTGACCGCATCTGATCTCATAACCAATTCTTCTAGATAGAGTTTCTGGTTTTCAGTTAACTCACTCT
CAATAGGGATAATATGGGCTTCTGGTACAGGTAAATCCAACATATCTTGTGTTTGAATGTCGGCTGTTTCTTTAT
AGATTTTCATCAACTCAGGTAGATTGACAAACTTTTTAAATCGTTTCTTAGGCTGGTACTTATCCCCTGTAGGAG
CTAATTCCATAGAGTTTTGAATTTCTCCAAAAGCACCTACCCAAGAGTCAAAATAATCAACTTGATAGCGTTTTA
AGATATCCGGTTGAATGTAGTTCATCATAGTATACAGCTCACTAATTGAATTTGAAACAGGTGTTCCTGTCGCAA
AGACAATATTTTTAAAATCATGTTCTTCCTGAATCTGTCGAACCTTCATTTCCATATCCACATTCTTCTTAGACG
TTGTATTGGTAATCCCTGCTACATTTCCAAGTCCAGTAATTGGACGTATATTTTTAAAGTGATGTGCTTCATCCA
CAAAGAGAAAATCAATTCCTAAGTTCTCAAAATCAATAAAACTATCACGATTAAAGCGTTGGAGTTCTTCCAATT
GTTTCTCAAGACCACTTATTGATTGCTCTGCTTCTTTAACGGTGTACTTATTTTCAGAATGTGTTTTAATCTCTC
GTAGTTCATTGAGTTTATCCTCGATATAATTCATCTGTCTTTCCTTACTGACAGGGATTTTTTCAAATTGAGAAT
CCCCAATGACAATGGCATCGTAATCTCCTGTAATAATACGTGACACAAACTGTTTTCTTCTTGCCTTCACAAAAT
CTTTCTTAGTGGTCACAAAGACTTTTTTAGTAGGGAAAAATTTCATGATTTCTTGGCCAAACTGAGCAGACAAAC
TAGAGGGCACCACATACAAGGGCTTATGAACCATCCCCAACTCCTTTAATTTAAAGCCAGCACCAAGCATGGTCA
AGGTCTTTCCTGAACCTACCTCATGAGCTAACAAGGCTCTTTTTTCTTCTACAATTCTTTGAATGGCATTCTCTT
GATGAGGACGAAGACTGATGTTTTGTGCCAAGCCATCAATGACTAAATGGCTACCGTCATACTCTCGACTAACCG
TTCGATTATAAAGACGATTATAACTTTCTTCAATGACTTGTTGAACTTCTGGATACCGTGAGACAAAGTCTTGAA
AGAGTTCTTGTAAATGCTGCTCTTTTGCTCTTAGAACAGAGGTTTTTTCCAAATCTGTGATGGTCTTTTTTCTTTT
CCCCTTCCGTAACAGTCATAGTAATAGTCGGTTGGTTCGAATTAAGTAAATTCTCAAAAATCTTTCTTCCTGTAT
CATAACGTGAGCCACTGACTCCAAGACTACTATCTTTGGCACTTGGATAGCGATAAGCAAATGGTGTCCTTAAAT
GAACCTGCCCATCGACAGGATTCACTTCAATGACTTGTTCAACATCAGGCGAAGACAATTCAAATTCACGGTTGG
TAAAACATTCAAAGGCAAATTTACCATAAACGGATTGAGGAATCCAACGTGACCCTATTTTAAACTCAATATCTG
CCAGATGAATCCTTGGAGGGCGAACAGATTCTAACAAATCTAACGCATGAGTCCAATCACATTCTTGGTTGTTTT
CCTCTACTAATAGTTGAACTACTTCTATCTTGTTGAGAATATCTCCTGACAAAAACTGGTTCTTAGAAAGATAAT
TTCTTTCCCCTCTTAAATAGCTTTCTGGATCCATTAAGATCTGGTCACCCAACTCATCTAAAATAGCTGCTTGGC
TATGTTCAGGGTAAATTGATACCATAAAGTCTATATCAACCCCTCTACCATCTGATAAACTGGAGTTTAAGGCAT
CTAAAGCCGTTGAAACTCTTGTAATCACTCTCTCCGGCCTAACCAATGCTTTCTCAAAGGCTAAAGATTTTTTAT
ATTTTACTTTCTGATCTTTAGAATCAATGTATTCATCTTCTAAACTTGCTAGTAAAGAATACTTATCGTCACTAT
CAAATAAGTTTCGATTGACTGAGACATTCAAGTATCCAAATTGGCTTACAAACCGATCATAGTCACGATTGAGTT
TACTAAGTAATACCTGAAAATCTGTCCGACTATAATCTGGATGGCGTTGAATTTCAATTAAGGCTTGATAGGTCT
CTCTCAAATCAACCATGCCCTTAATGCGACTAATATCCTTATCCGATAAGGGGCTTTCATAAAAGACCGTTTTTT
TGAACAAGCCCTTATATTTCCCTCTTTTACTCGCTTCTTCTGACTTGTATACATCTAGTGCTTCCTCATCTGTCA
AATGAAGTTGCACGAATCGATCTATTTTATGTTCAGACAAGGAACTGTCCCAAGCTTTAAAATCTCCCTTCTCAT
CTACATAATAACTAATTTCGTCTACTTTTGAACTTCTCCGAATGCCATGCGTATCTCGATAATAAATTTGATTTC
CCTCATATCCAAAAGAATAGAGCGCTAAGTTCTCACGTATATGACTTGGGATAGAATTATCCACTTCTTCTTGGA
TAAAAACAGGTGCTTTCAAAGAATTGTCAATTTGTTTAGGTGCTTCTACATTCTCTAATGCTTTCATTATGTCAG
TAGCTAATGTTTCTGATTCCCCCTTAACATTGAGGGTTCCTCCATTAAAATTACGTACCTCATATTCACCCAAAA
CTTGTGTATTGTATTTCCCATCAAAATAAGGATTGATCCAGACACGCTTATCCTCCTCAAAGGGAACAGAGCCAC
TAAAGACAAGTTCCTCCTCATTAAGATTCTTTGCTTGATCCTTTTGAAAGAATAGGAGATCTGTGGTCACTCGGG
TACCTGCAATCTTTTTAAAAGCCGTATCCGGCAACCGAACTCCCCCTAAAAAATGAGTATTGGATTTAATCTCTT
GTAAGACATTATCTGTCCGCTTATCCATTGTCCCAATAGATGAGATAATCGACACTTGTCCTCCGTCTCTTACTA
AATCAAGTGAGTGTTTGACAAAGTAATCATGAATCATATAAGGTTTATCATAGTTTTTATCGGCAATGCGAAAAT
TTCCAAAAGGAACATTCGTTAAGACTAAATCAAAACTATTATTTTGATAGGGAACTTCTTCAAATCCTCGCACTT
CAATATGGGTATTGGGGTGGAGTTGTTTTGCGATTGCACCAGTCACACTGTCTAATTCAACCCCATAGAGTTCTG
ATTTCTCTCGTATACTTCTAGGCATCGCCGCAAAGAAGTTCCCAGTCCCCATAGAAGGATCTAATATCCTTCCTC

CCTCAAAACCATCATCCAGTAATTTTTGCCAAATCTGGCGAATAATCATTGGGTCTGTATAATAGGCTGTGAGAG
AACTTTGTTTTATGGTCGAGTATTCTGATTTACTTACTAAGCTCTTAAGAGTTAAACGTTCTGTTTCATACTTTG
GATTGAGTTCATCGAAAAATTCATTGGCAAGACCGCCCCAGCCGACATACTTGGCGAGTAGCTCTTGTTCTTCTG
GATTCGCTTGTCGTCTCTCTTTTTCTAATCTTTTAACAAGTTCAATTGCGGCGATATTCGTTTCAATCTTTTCTC
GATTTGTCTTAGGATAAAAGTCCTCTAAATCATCTGGAAAAACAAAATCTAGAACAGGGACATCCGTCTCTTCTA
TACCTGAAATTAAAGATTTTGTTTCCTTATCCTTATCCTTTTCATTTACATTTTCTTCTTCCAGGTATGAAAACA
AATCTATTTCCTGACTTTCACTTGATGAATCAATCTCAATCTCTGAATCTTCTTTTTCAAGTTCTAAATGAGACA
ATACTTGTTCAATCTCTTCCAAACTGTTCAAGTATAAGATAGGATTCTCTTCAAATAACTGGTTGGAATCATTGA
ATAGCTCTAGGCGAACTAAGTCATTTAACTGCGCATTTTCAATCGAAACCAACTGAAATACTTGTCCTTTATAAC
TTACTTGTGAACCGATTGGATATTCCCTCAAAGCTTCTTCTACAATTTTATCGACATTAGAAAAGGAGTTAGTTT
CTTCCGTTTCCTGTTCAATATCACTTGAAGGAGATAAAATTTTCTCTACTTGATTAGACTTTTCGCTTTTAGCTT
TTACTTCATCAAAATGAGTAATAATCTTCAGCTTTTGTGTCAGTGGGAGTTGTATGTAGTTTTCTTCATGATGGA
CAAGATCCGTAACGATATCTAAAGTAGCCATCAATTCTGAACCCAAATAGGCTAGAACATCTCCCTCTTTTTCTT
CAAAATGAATCTCAGGAACTTCTTTTTTAACCTCTTTTGAGTCAACCATTAGAAGAAATTCCTCTATATCTCTAC
TAATACGGTCAGATAGATTATTCGCAACCCGATAGACATTGACTAAATTAACATCCTGATTCTGATGAAATACAA
GCTCACTTAATGGTGTTAGTTTCTCTTTTTCAGATTGGATGTAAAATCGAGTGGAAAGGTTATAGCTTGCGACTT
TTAGCACCAAATTTTTCTCAAAATTACTTAATTGAGATAATTTATCTAGTCCTGCTTGTCCAAAATTTCTTGTGT
AACTTTCTAGATCTTGGTCATTATTTTCAGAACTGGCCTCTAGGAAAGAAACAATGTCTTGATGAAACGAATAGA
CATGAGGTATCTCTTCTATCTTCTCCTCTTCAGTCAAAAAACTCTCTACCAAGTCCTGCCATTTCTCATCAAAAT
GTTGCGCCTGATAGCGAAGAAATAGGTCCATTTTATCCCTATCTTTTGGAATTGTTCCACGAAACATAGCCAATA
ATTGTATTACTTCCATACTCGCTCCTTTCATCTATACGAAAATAGGAGAATCACATGATTCCCCTACAACTCTAT
CTCCATTTCAACCTCTTGAGGACTGGTTTCTAAATCACTAGATAAAAGAGAAACGGCATCCATTCTCATGTAGTA
AGTATATAAGTCTTCTAGGTCTTGTTGGTTTTCAATCCCTGTTTCAATACTAATCAGAGCCAGTATAAATTCCTT
GTAGTGACTCTCCATCATATCTGAAATCTTTTGTTTCATTTCCATAAATACTGATGACGATTCAGTGATACTTGT
TTGGTCTATGTCTTGTAAATCCTTTTCTAAGGGATTGGTTCCTTCTTCTATTTCCACTAAATCTGAACGTCCATC
ACCATCAGAATCTGAGCTAAGAGGATTGGTTCCTAGGGCCAATTCTTGAGCATCAGTTAATCCATCTTGATCCGA
ATCACGTTGATAAATGGCTTCCATATACTTCCTTCTTTCCTAGTTAATTTTTTTTCACTCCAATCCTACCAAATA
TTCCTTGAAAGAAGTATCACAACAAAAAAAGAGAACGAGCCAAAACTGCGTTCTCCTGAAAATGATAAAAATATA
TTTTATACCACAAAAGATATGTAAACTAATCTTTTAAATAATAAAAAAGTTCCGATGTTTGTAAATGTTTTGAGT
AAATTGAAATTACTTTTTGACCTTTTGGAAATACTGTCTTTCTCTTCAAATCTAATAAAGATTGAGGAACAATTT
TCCTAGAGGTCTCTTTCAATGTTAATGCTATTATCTGTTTTCTTGTACGTAGAGCATAATCAAACTCCAAATACA
AATAACGTCCTGAACCAGTTAGATGAACATGCTTTCCCAATAACTCCCCAATGGATCCTAAAACTTGTAACTTTT
GCATCGTGGTTCCATCTTTTTTTATTTTCAACTCATCAATTATGACATGTCTATTTAAACAATCGTCAAAGAAAC
TGCCTGCACCTTTTCGGTAATCAACACCACATAGGTGCATGAAATTAGTAGGTGAAAAATGAAGTTCAACACTTT
CAATTTCCGTTTCATAATACATAATTTTGCCAACAAAGTGTTCTTTAAAAAAAGAAGCCGCAATTTGTAACTGTG
TAAAAAAACGCTGAAGTTTTATTGTTTCGGTGTAATTTGGATTACGATAATCTCTAGAATTTGCCATATTTTCCT
CACAAAAAAAGGTGGTTTACAGAGTCTGTAAGCCACCTAGTCAGTCGGTTTATTCTGGTGTCTGCCACCGCTTGG
CCCTTACGTCCAAGATTGCTATCGGATTTCGTTCTGGTGTCCGCCACCGCTTGGCCCTTACGTCCAAGATTACTA
TCAGATTGACCATGAGCCGACCACTCATCTACACTATTTATTTTACCTGAAAATAGCTAAAATTGCAAGTGTAAA
CTTCTTTATTTCGTAGTATAGGCAAAACTAATGGCACTATCTGCATGAGAGATTTTTCTAAAGGTATAGTTAGGG
TTGCCACCATAGTTAGTTTCAGACACAAGGAAAGAACCATCATCATAGACCTTCTCTACAAAAGCCACATGACCG
TAGATAGCTGGTGTACCATGTGTACCTCCTACAAAAGAAACAATAGCACCTGCTCTTGGTGTGGAACCCGTTTCT
CCACCAAGACTTGAAGCTGTCGCAACCCAGTCCTGACCATTTCCCATGGTATTAATGATTGAAATCTTCTCTCCA
TTGCTTCCTTTTAATTTTAAGCCTAATTGATTCATACGAGCCGCAACACCCCATGTACATTGTCCATAGGCATAG
GCCATACCATCTCCACCACCAGGAACAGAATGTTCATACAAGTCTCCACGAACACCTTCAAGGGATTGCGGGTCA
CTTTTTGCCTGTCCTCCATTTGTTTGGCTGAAGCCTTTTTCAATTTGGTAATACCATTCCGTTGCTCTGGTTTGT
CTTTCCAGTAGTTTGTCACCAGAATTTCCCTCCCAATAGGTGAGGAAGAGTTGGGCGAGATTGGCTGCACTGCCC
GTATTTTTAAAGAAATCCTTTAACCAACTTTGATAGTAAGGACTATCCCCATGAAGCATAAAATCAAGTTGTAGG
TCTAAATCATACCATTTCTTATTTTGGGTGCGTGCATAATTTAACAAAGCTGTATGACGTGTTGAACCATCTGCG
GTATCCGTCCATTGACCTAACCCCAAACCTCTATGAAGGATATTAGGATAAGCACCACTATAAATGGCTGGCCCT
CCTATCGCTAACCAGGTTTCATCATCCCATGAGGAATCGGTAGCGCCAACTGGAGGAGATAAATAATCCCCTTCA
GCTCGTTTAGGATTGATAGAAGACTCTACCGACCAATTTCCTAAAATAGCCGCAATGGCTTGGGGACTTGCCCCT
TGAGATTTCAAAAAATCATAAATATGTTTTGCTCGTTCAAACTCATCTCCACCAAACTGACCAATGGCAGGTAAG
ATAGTTGTCTGAAGTTGAATGACTTTTGGAAAATAAAATTGCGGATTGACATATACCAATTTTTCTTTCTTGTTC
TTATACTTTTGATAGGAAACTTTCAAACCTGTATCATCTGGTGTTTCACCAATAACATCACCCGTTAAGACCCTT
GTCCCCTCAATCGCACGGCCATTATGAATGGAATATAAGGTTAAACGACTCTCATTCTCACCTTTTCCGTTAGTG
AGAATAACATCATCTCCATCTAGAGATACAACTCCATCCATTGGTGCGACAATCGTTTGGTGAGCCTTCGCTTCT

AATAGAATGTACTCCTGAAGAGTAGGTTTTCCGTCTAAATCATAGTATCCATAACGATAAGTCATGGTCAAACTA
TCTTCGTTACTTTTCCCCTCAAATGGATTGTCCAATTCCTGCATGGAAGCATACACACCTTCTTCTTTTAGTTCC
TTTATTTCCTCTTGATCGTCTTTCGAGAGTTTATACTTAGGAGTTTCATAAAGGTCTTGCATGGATTTCAAATCT
TCCCCATCGTTTAAATCATGCCACAAAGCAGATAGATAATCCTTGTAAGTTTCTGAACTAAACAAATGAACTGGT
TTCTGTAACTCATAGTCATGGAATTTAAAGTTCATATAGCCCATCACATCATCAACTTTTGTGTAATAGGTAATT
CCTTTGTCATTTGTACGAGTATGTTCTGCATCTTCCCAAGTTAGGTGGGTATAAGCTTTTGTTAATTCAAATTCA
TCTTGTTGAATCAAACTAGCAGATGAAAATCCTAAAAAGAAGCTCATCATAAGTAAAAGAAGAAAGACTATTCCT
CCAACTATCCAGATTACAGGATTTCCAGCCACAAATGTAAAGAAGGAAAAGGCTCCTTTTAATTTTTGATAGATA
TTTCGGACACTTAAAAGACCTTGTTTCTTTAATTTTCGATACCGATTTTTAAAGGAACTTGGATTATCTTTAGTT
AGTTTCCATCCTTTTCCATCCTTAAAATGATGGTATCGCTCTTTTGTGTTGGTCAGTCTTTTCTTGGTAAAACGA
CCTGTTGCTTGTCCTGTTTTGACACTAGCTTTTCCAAGATTATAAGAAAGCCGACTGTAGCGTTTCCCTTTTCTA
ATGGTCTCATGAAGTGTGCGATAACTTTCTAAATCTTCATTTTCTGAAGCTAACTCTCCACCTTCACGTCCAAGG
ACATAAAGAAAGGTTTTGGCTTTCCTACTGATTTTTTTGAACTTATAGGCTTGCTTAGTAGATTTTAGATTCTCT
TTTGCGGCTTTGACTTCTTTCTTAGCTTTTAATTCTTCTAGATTCTTCCCTTGAAGGAAAAAAATTGGATTTTACT
TTCGTTTCCTGACCGTAGAAAAATTTTTGATTGGTTTTTCTTTCTTTACGACTCTCTTTTCTTTCTTCTTTTGCT
TCAACCTTAGCTTCCTTAAATTGTTTCTTGGCTATTTTCAATCGTTTCCTAGCATGAGGCAATCGTCTATCTCTT
AATTCTTTCCGATTCAAAAGTGATGGAGGACTATTTTGAAGAATATGATTGTAGTCTTCATTTGCTTGTTTTACT
CTAGCCTTTGAAGCTTCTCTCATATTCTCCAGCTTTTGCTTTATCTCATTTTTCCATGCTTTTTCATCCAGTACA
GCGGAATCTTTTTTCTGTTTTCTCACCTCCTTCTTTCCTTGTTTTAAGAATTTCTTCTCATCTTTTAGACTTCTT
CTAAATGCCTTTCGGGCACGTATGATTTCTCTCTTATCCTTCATTTACCTTCCCCTTAATTAGAAGCCATTTTAT
CAGGATCTGTACTCATGATATCAAACAATTGTGTACCTTGGGGAATCTTATTTTTAAAGGGAACGACAACTGAAC
CAGCTTTTATTAGTCCTGCCCCTTTTTCTGGATTGACTAGGTATTTTTCGAGTTCTTTTGACAAGCCTAAGAGTT
GAACTAGTTCTTCTCGGTCATTTTTTGCTTGCTTGAGGAGAATCATAAATTCACTATTGGCAATAATCCGTCTAC
CATTTGGATCTAACAATAAGGTTTCGACATTTTGGGTTATCCCAGTCGGACTGGCTCCATATTTTCTGACACGAC
TCCACAATTTAAAGAAGAAATCACTGGCATATTTATCTAATAAAAGAAGCTGCATTTCATCAAAATAAATCCAAG
TCTTCTTCCCTAATTTTTGGTTCCGAACGACACGATTCCATATCTGATCAAAAACAACCATAAGAGCGATTTGTT
TCAGCTCATCTCCTAACTTTTTAACGTTATAGATTAAGAAGTTAGATCCTGTCTGAATATTGGTCTTATGAGAAA
AAATATCAAGAGAACCTTCAACATACAGTTCCATATCAAGTGCCAAATTCTGCGCTTCTTCTTCTGGTTGTTGGC
TCAAGACAAAGACCCATTCTTCCAAAGAAGGCTCTTTAAATGACTGATAGGTGAGTCTGGTGACTCGGTCTATAA
TCGATTTTTCTCTCCCATCCATTTTTCTATCCAATAACTTGCCAATAAAAGATAAAAGAAATTCTGATTTTACCT
TTACAGGATCCTCATCCATATTTTCCTCAGACAAGTCAAGGACATTGAGATAAGTTTGGGAATCGGGCGCAATAT
CAATCATTTCTCCCCCAAAAGCTCGTCCAATGACACTGTATTCTGCTTCTGGATCCACGATGATAATTTCAGTAT
TTTCACCAGATTCCTTGATTTTGGTCGTGATAATTTCATGCTTAGTTGCCATCCCTTTCCCAGCTCCAGATGTTC
CTAAAATCAGACCAGACGGTGTATTTAATAGGCTGCGATCAATGGTAATAATATTGCTTGAGATTTGATTGATAC
CATAATATTTCCCACTACGGTCTTGTAAGTCTACTGAAGTCCAAGGTGAGTTCACTGCTATATTGGACGTTAATA
AACTCCGTGATACTCCCTCTAAAAAATCACAACCAAATGGCAGCAAACTATTAAAGGCTGCTTCTTGCATATATG
GAAGTTTATCAATCATTAGGTCATTTGAGCCGGCCACTTGTTGGATAGTGTCTAGGGCTTGTTTGAGTTCTTCTT
CATCCTGACCAAAGACCCCAATCAAGAAGACCGTTTGAAATAGTTTATCTCCTGTCTCGGTCATGGTTTTTAAGA
GTTCCTCAGCTTCATCGATATTGCTTTCTAATACATGACCTACTTTTTCCAAATAGATACCTGTACGAGCTAGTT
TTTGTTGTTCCCCAATCTTTTGGGATTCCATTAAGGTTTTCTTTGTTCGTAGTTTCTTCATGGCATCTGCCTTGG
TCGAACTTTGAGCATGAAGGCTTACAATCAATTCCAGATCTCCTTGCATGAGGTCTCGGATAAACTGATCCCCTA
ATTCCATACCGTAGTCTCTCACATAGACAATCTGTAATAAGCGGTCATTGATTTGTAGGTAATTCTTGTTTTTAA
AATCCAAGAGATTAGGTGCTATGAAGTGACGAGTTGTCTGGCCAGATCTCGTTAAATCACGATAAGAAAAAGGAA
GATGGTGTTCTCCTCTAAGCATATCTGCCAACAAGTTCACCCGTTCTTCTCCAGTCAAGGATCCAAATCGAGCAT
CAATTTCTGAGAAACCACTCTTGAAATATTCGCCTATTTGAGACAAGGAACGATAAGCTTGTTTGGGATTAGAAT
CCTTTCTACCAAAACTAATCAGTTTCACAGCCGAAAAGTTATTTTCACCACTATCTAAATTTTGATTCATCATCC
GATTCAATTCTTTACGATAGCTATCGTACCCATCTTCTTTTTCCTCATACAAAACACTGTGTCTGAATTTTTCTA
AATTCAATCTTTTATTAAAAATGGTCAATTGGAAGTTGGTTTGGTCATCTAAAGAGTTAATCAAATCAGAATACT
TCTCAATGATTGCTCCCTTATCTTCTAAACCAACGGTCTGGTAATTGACATCACCAAGTAAATAGCTTTGTGAAA
AATAATCTTCTTTTACCTGCATCAGACCATTTTGATACAAGGCTTGATAGGAAAGAGTATTAGCCGTTGATGGTA
ACACTTCCTCTTTTTTTATCTTTAACTTCTTCCTTTTTATTAGTCGTTGAAGTCTTTTGTTTCTTTAATGTATTTG
ATTTTCTTTTCATGTTCAAGTCCTTTCTTTCCTGAAATTGTGCGTAGGGGTATCGTTAGTTCAAAATGAAGACGG
TATTTCAAATAATGTTCAAAATATAAATCATTGGGTTTATAGACTCCAAAAAGCAAGAGGGGGATGGTAAAAGCA
AACACAAAACCGTAAACAAACCAATCTCCAAATTGCCAGAAAAAGAGATTCAAGCCCAAAACAATAATTGTGACA
ATAAAGGCTGGTAAAACAAAGATGATTTGTCTTGTGGTGAAACCTAACCAAGCCCTGTGTTGGTATTTTGAGATG
TCTTTAAAGACACGTGTATTCATGACTTTCCTTTCTAAAAAGGCTAAGAAGCAACCACTTCCTAGCCTTTATCTA
ATTACATACCTAAGATTGAGCGAGCCGTACGTTGAGAACCAACGAGGGCAATAATCAGTAAGATAGCTTGTACCA

AACTACCAAACATAATCGCAAGTGATTGCAGGACTCCTGCACCATTTGAAACAGCTATTTTACCAGCAGATTCAA
ACAAAGGAACAAGAGAAACAATCAGAAAAATAAGAACCCCTTGTACCGCATAGACCATAATATTTTTTAAATAGC
CTAAACCAATAGACTTCCATTCATCACTTAAAAATGTTGGAATCGTAAGAGGGGCAAATGGGATCATAAGGTAGA
GTTGAATAAATCGAATAGATACCAAAAGATTAACCATGGCTGCACTTACTATCCGAACAAGCCAAATGAGGAGGG
CGAAAAAGCCCACAATCATCCGGCCAATAAATCCTGACCCTTTTAATCCAGAGAGTGTATCATACTTTGCCCCAC
CGTGAGCCACAATCGAGGCCACTTGTTCAATGGCATGACTCGCAATCCCGATGATGGCTTCTACAATGACGGTAG
TGTTGGTAATTACAACTGCGACCATAATATAACTAATCAGCATTGGCGCTAAGGCTTCAAAGGTCATCGCTCCAC
CAGAGTTAGCAATTTTCTTAGCCATCTTCGAAAATTCTAGGATGAGAACAACCGATAAAATCGCAACTCCAAGGG
GCTGCATGACACTTTTAGTAATACTAGACATATAAGTCCAAACGGTTGGATTGTAGTTAGATAGTGATTTAATCA
GATCTACCGTAGATTGTAAATCTACATTAAATCCTTCAAATAAATTTTCAGCTGATATTTTTTCAGATGCAAGGT
AAACAAAGGGTGAGACTAAACTAAGATTCATGTCATTGTTTATCCTCCTAAATTGAAATCTGGGTTACAAAGGCT
CCAGCAGCCCCGACCATAACTCCACCGACAATTTCAAGAATGGCATTCCGAACACCTGGTCCTCCATCTTTAATG
TTGGTTGCAAGATTGACAATCCCCACAACAACGAGAAAGGCACCAACGGCAATCAATCCCTTCTGTAACAAAGAC
ATAGCTTGTGCAAACATAGCACTTGCGTCTACTCCATAAACAAAACCTTTAAAATGCGTAAACATGTATTTCCTC
TTTTCTATTTTTATTTTAAACTAGATTCAAAAGTTAAATCACGAATTCTAAGGCCTTCAAGATGATTTTCTTGAT
TTTGATTCAAAGGATTGATTTGATAGTTCCACCACCGTTCATCGCTTTCTTGATTGGCTAGGTACCTCCAGTTTG
GATGCTTGGTTGAATTGTATTTTTTGCTTTTAAAGACAGGCATATTGGCAATTCGAACCAAGCATTCATGCCGTT
TCATATTTCCGACCTCATCGGGTGTCATTAGATCACGAGCAATCTTTTGATGAGAAAGGGATCCTGAACCTGTCT
GGCCAAAGGAACGACTAGTATTTCGAACATCAATGGTTTGTTTACCGAGTAAACCACTCATAAATTTAAAGGTAT
CTTCATCATTACCACCTAAGTATACTAAGCTATCACAGTTCCCAAGAATGGTTTTCCAAGCTTCTTTTTCTTTAT
AGAGCCCTTGAAGTTGGGCAATATTTTGTAGAATAGGAACGAGACTCATATTTCGAGAACGGACTGTTGAGGTTT
GTTCAGCAAAATCTGGGATTTCTCCGATATTTGCGAATTCATCTAAGTAGACTCTCACATGAAGAGGTAATTGCC
CCTTAAAATCAATATCTGCTTGTCTTGTTAGAGTTTGAAATACTGTTGAAAAAAAGAGGGCTGAAAGAAAGCGAA
AGGTACTATCGTTATCTGGGATAACTAAGTAAACCATTGATTTTTCCTTGCCCCATGTCTTCATATCAAGAGTAT
CTCTTTTGGTCAAATCCATGACACTCTGAATATTGAAGAGGGCAAATTTAGCAGTGGTTACAGCTATAACAGAAT
CCAGAGTCTTATCCTTATAATTTTGAAAATCTGCCCAATTTCGCATGGTAAAATTTTCAGTCCCATACTTTTTAG
CATAATTTTCAAATAGAATTTCTAAGACACTTTTTTCTTGGTTTTCACCCTTGGATAAGTGTTTAATGAGTTTTG
AGATTTCAGCAAAACTTGGATAACGCCCTCGTTTTTTTCGCTCCTCCACTTCTTTTTTTTGACGTTTCAACAAGT
TTTTGTATTCTTTTTGACTTAAACGACTTTCTTCTATGAGCTGTTCTCTTGTTTTGGGTGGATTATAGAAATCGA
CCAAGTAGGAGGCTAAAGCTCGGACCAAAGTCATAGAAGCTTCATCCCAAAATGGATCACTACGAGAGCCAGAGC
CTTTGGTGTTATTGAAATAAACCGTCAGCATGCGATTCAAATCATTTTCTGTCTCTATATAGCGAAAAGGATTGA
AGCCATCTGAGTTCTTCATATTGACTAAATCTAACACCTTTACTTGGTAGCCATGTTCTAAAAAGAGTTTTCCTG
TTTTCTCGGCCAAGTGATCTTTAGGATCCACTACAATATTAGAACTATTCATCTGAATTAGATTGGGTTTCACAA
AGCGAAATGTCTTCCCACTTCCTGAACCTCCGATCACTGCAATATTCTTATTTCTATCATATTGGGGTGGTTTTT
TATCTAATAATGTCAAACGAACATCTTGTGCTAAGATCGTATCATGTAAAAATTCCTTACCGTAAAAGAGCTTCT
TTTCTTTTAGAGTTCCAAAACGGGCGCTCCCGTATTCTACCCCTTCTCGGTATTGTTTTTTACCAGTCTCTAGAT
AGAGATAAACCAGCAACATCATCACAAAGCCTAGTAGAAAAAAAGCACTTGATTTTCCAGTAAAGGAAACATTCC
ATGGCGACTGAAGAACTTCATCTTGACCTTCCATCAGAAGATGAATCCATTTATCTAATGTATTTCCAGTATAGG
AATCATACAAAAGCGTCAAACGATGAAAAAGATAGCCTAGTAAGATACCTAACAGTGAGAATAGTATGAATTTCT
TTCCACTGTACATCATCTCACCATCTCTTTCTGTTTGACGGCACCTTCTTGTCTAAAGGTAATTTGGGATTTAGC
CTCATCAATTGCATCGTCTAATGACTTATCCATGATAAAATCAGCTAATTTCTCCGGATCATTAACCATTTTTTC
TAACAGATGGTCTAAATGATTGTCTAGAATCGAACGGTCTTTCGTGTAGAAATGCAGAGAATCCCCTTGCCAAGC
GATGGCTAAAGGAATCTCTTCTTTTTCTAAAAAAGCTTTAAATTTCTCTATATCAATTGGTTTGTCTAAAAAATC
TTTTTTCAGATTAATCGTATCAATCGAATAGGGAGATTGTAGCAATTCTTCTAATTTCTGCACCCCTATCTTATA
GGCGGAATCTTGTGCTAAAGCCTGACGTCTAGACCATTCTAGAATCTTTAAAAGACTTTTTACAGTAAATAAAAG
ACTACGCTCAGCATATTGAACTGCCATTCGTTCCTGTTGTTCAGAGGACATCTGATGCCTCCTTCTTAACAAATA
GCAGCTTTCCTTCTTTATAACGATAAGCTATCAATTTCTGACGTTGCTTAATCGACTTGACAACCTGCAGTAGAT
CTCTCGAATAAGGTTCTCGAAGAGTAACTTCTACTGCTTTCCCATCAAAGATACCAACACGTTTAAATTCAATGT
AGTCTCTTTTGAGATGTCCTAAGCCCATACCAAAAGGAAAGTGATGAATCAATTGGATGGTACAACCACCTTTAT
TTCCCATTTGTTTCAAATCATACAAGTTTACTACCTTCATGATTAACTCCTTTATCTAGTTTGTCGCATCGTTTA
AGTCTGGTAACGATAAACTCCGTGTCTGTTAGAAAATTCTCACACACGTCTTGTGCCAGTCGCCCTTCACAGGGA
AATACTCTCAGTCCCTACTTACACAGGCACGCTAATCAAGACGGAGTGGATTCAATTTTCAAAGAACAGGTAGCT
TTATTATAGATAAGAGTAGTTGAAATTTTTATCACATTTTTGGGGTTGTTGGAATAATAAGGTAAGTGCTAATTT
CTAGCTCCTCTCACAACACCGTACGTACCTTATATGTTTTATGAACAGTAGCTATCATTTACATTTTCAAACTAC
CATTCTATCAAACTAAAAGTCAAAATAGACGCTTCTACTTCGAGAGATACTCATTTAATGGTTCAGCAATTCGCT
CTACTTCCGTTACAGAAATTTCATCACTATGACTTCTAATGATTTGTTATTAATTCACTTTGAGGGGCTTGCTCA
TGAAACCAAACAGTGTAAGTCGTAGAAGAAAAAAGCAGTTCAATTGAACTGCTTTTACGGATTCAGATAAAATAA

CTTTAAAATATAGAAGATATCTATTGACTGTAACTATAATTGATTTAAATTTATAACTTCATCTGCTTGATTCCA
AATATCGGGATTATGGGTTGCAATAATGATTATACGCTCTTGATTCTTTAACATTAAGAGCAAGTCCATCACCTC
TTGTGAGGTTTCTGGATCCAGTGCTGCAGTTAGTTCATCTGCCAAAATTAAAGGTGGATCTTTTAAAATAACTTT
AGCTAATGCGACACGTTGCGCCTCTCCCCCAGATAATTCAAAAATCTTTTGATCCAGAGCAAGGTAAGCCAATCC
TACTTTTTTGAGCACTTCTTCTTCTTGCTGCTTCTTCTCTTTTTTCGTTAATTTTTGCCCAATCAATCCCAAATC
TAAATTTGTAGCGATAGTCTCATTTTCAAGTAGGCCAAAGTTTTGAAAGAGATAGCCTAATTCATGCTTAAAGAA
GTGATGCTGTTTGATTTGTTTCAATTCTTGCCCTTGATAGCTGATACTTCCCTTTTCATAAGGCTCTAACTTTGC
CAAGATATTAAGCAAGGTTGTTTTACCACACCCGCTATTTCCGATCAGAGCATAGACCTTACCTTCTGTAAATTG
CAAGCTCAAATCTTGAAAGACCGTTCGTTGGCCAAATGATTTGGTTAAATGTTCTATGCTAATCATATTAGGCTC
CTTTCAGTACAGTGGCTAACAAGTGGTCTTCTTTATGAGACCGGTACAGGAGAATAAACCAAGCATTAGTAATAA
ATAGCAAGAGAGTGACAAGAGCAATCCACCATTCTTGAGTGAGAAGGAATGTCAGGAAGCTTCCTAATAGTAAGA
GGATACTTTCTGAGAGCAAAATCATTTGGTGAATCTTTACAAATCCCAGACCTGCAATCTTCTTCAAGAAGATTG
GTCTTCTAAACTCTTCAAAATAGAGGAAATTCATGGTGTTAAAGAGTAGTATGGAAGTAGCTATTGCAAAGATTC
CTCCTGCAATGGTAATGAGATTCTCTAATTGAATGGATTGGATCATCTGCTGGTATACAGACGCCGCATAGTCAT
ATTGACTAACATTTTTTTCAAGCCCATTCTCAACTACTAGTTTCTTGCTTTTTTCCAGACCATCAAAGTAAAGAT
AAGAATTAAGATGAGAGAAATAAGGATTATCGTAGCCACCAAAGCTTCTAGGTAGAACAACAACCAAAATCGGGT
CTGTCAAGAATTGCTGATAGATCATCGGTGTATTATTATAGATAAATCGTTTTTGGTTGTTAGGAAGATAGGTCA
CCCGTGCTTTCATAGGCAACTGACTCTTTCCTTGTTCATCACTAGGAGTCAGATAATCTTCATAGCGTTTTTTCA
ACTCCTCTTCTTGCCCTTTCAACTTTTCAGGAAGCAAGAGTCCAAATTCTCCAGCTTGCAAGTGATTCAAGCGCT
CTTTTTCCTCAGGGGAAACTAAGATCCTTTGGATATCAAGGTAATTTGGTGTTACATACAGGGTGTTGGCAAGAG
GATCGTAATCTGTGATCGAAAGCTCTCTCCCTGTTCTAGGATCGTTCATGAAGCCTTTTGAATTAAATGCGGCAA
GCTGATGATGGACCAAAAGTCCACCTTTTTCAATGCCAGACTCGATCAGTTTAGACCATTTAGCTATTTGTTCAA
TTTGCATTTCTTTGTTTGTCGTTTGTAAAACATTTTCCCGATTCAAACTAATTTGTATCCAATTTGTCTCTTTAG
ACCAAGCCAAACTTCCCTCTTGATAAGTCTGCCAGATAGAGCCATAGATGCTGACACGATGAATTGAGAGACCAA
TGACAGTGATTGCAAGAAATTGACATGTAAAGAGAAAAAACAAGACTCTTTTTAATGGAATTTTCCCCTTTAAAA
GGCTAACCAAGTGTACTGTCTGAATACTGAATGCAAAGAAAAAGGATAGGAAAGCAGATAAAATAAATAAAAGTG
TATTGTAAATAATACTAGCTGAAATGATGAGAGAATAGGCAAAAGGAGTCAATTGTAAATAATAAATCAGAATAG
CTCCTAGGACACTTCCACCAATACACCCTAAAAACAATTCTTTACCGTCTTCCATGAGAGAATGTCCAAAGAGCT
GGTATCGTCTTATTCCAGAAATGTAACGAATCCCTGCACTTCTCATTTCAAGTGTTTTTTGAATAATGGTCAATG
CAGCAAAGCTGATGATAAAAATAACCAAGGCTAGAGATTGGGAACCCGAACTAAAAAATACCATAAAATTTTGCA
GGGGATTGGGCTTATTCATGAAACTTTTTGAAAAACCAAGATCGTGGAGTTTTTGATCTAATTTTTCAAGGGTTA
AATTCCCAGATAGTATATAGTAATTGGTAAGCAAACTTTCCTTTGCAACGAATTCTTTCTCTTCTTTTTTGATGC
CATTAGGAAGTGCTCCTTCTCCGTAGATATCATACGAAAACTTGACTTGCCCTTTAGAATCCGTTTTTTGAATCT
GACGAGCAATCAAACTATTGTTTTCTTTTGCTAACTGATCTAAGCTAGATGAAAGCTCTTCATAAACAACTTCCC
TTTCTTGTTGAAGAATCCCTATTACAGGTAGGCTTCGATGGATGACCGTATTAGGTGAAATAAAGGCAATCCAAA
GCAGAAAGATTAGAATAAAAAGGTTCGAGAAATGTATAAATAAACGTTTCATAATTCTACTCTTTCTAGTTTACC
CTAGAGAGATTTCTTCTCTCTAGGATAAACTTTAGCAATTAAAATCCATAATTAAAGTATGCAGTTTCACCAAAA
CTTGTATTTATGAACGATCTTGATGTGTAATGAGCAGAAGCATAACCTACATTAGCTTTGCTGTCACTTGCTCGA
GTCACACTTGACCAATGCCATCGATAGTCGTGGAAATAATTTGAGAATGCTCCCCAATTGCCTGGATTATGATAA
CCTCCGTAACTCCAGTTACCACCAGATACCCAATCAGCAAATACAGGACACGCTAAGCTACAACTAATAATTACA
GTTAAGACAGAAAGGATAATTTTACTCGTTTTCATGACTGTTCACCTTCCATAATTAGAATTAAAAGCTTTATTT
AACAATAAAATTATGAATTGCTCAAAATATAGTTTCCGTTTTTTCTCCTTTCCAGAGCAACCCTTTTCGCCTGCA
TTATAACACTTACAACATATAATTTCAACTAATTATATAATAGATTTTTGAATATTTAATAATCAAAACAATATA
GCTGTTTATTCATAAGCTATCAACCAAATTCTAAAACTATTAAAGTTTTTGTATTAAAAAACATTAAGATAGCAC
CTCCGTATAAGATTGTAACTTATCAGTTACTTTAAATAGCTGGGCATAGACATTCAACCAAAACTCGTTCAACTT
CATAAACTTTAATAAATTGACCAGGAAGGCGCTCTATTTCAATAATTCCTTCACTATAGTGAGAGCGTAAAATAA
TAGGACATATATCTGCTTCCTTAATGTTTTTGGTATTCGTACCATAAGGAAAACTCATCGTCATGTTAAAAGGGA
TAGTCAAAGATAAACCATGTAACCAAAGTGCTGTCTCCAAAGAAAAGATTCCCTTTGGAAATCGATACTGAAGGA
CAAACCACTCATCTAAATAAATATCAGGTAAGCGATATAACCCTTTTTCTTCAGCATCGATTTTTCCTTCTGCAA
GCATTTTCAATAAGGTTTTGTAGTGTAAATTTTCATCTATTACTTGTTTAAAAGTTAAAAAACCATACTGATTGA
AAGAGTTCATCAGTTTTTCTCTTTTATCAACCATTTCATTCTCCTTTCTTAGAAATAAAATGCTAACATCTTATA
TATCTGTTAGCATTTTATTTCTTTAAAATCAATTCATTTAGTTTTTTATTTCAAACTGAATCTATAAGTAAGCTA
GACCACCTATTATAAAGAAGAAGTAAGCCATGGACTAATAGTTTCCACTCCAAATGAAGCCCTAGAAACATTTCT
AGTAAAAACATACAAACAGCAAGATAAAATGCTTGTCAAAGAAACTCTGATATCTAATTTTCTGATTAACTTTCA
TAAAATTTCTCGATACAGAAATTCTTCGACAATACTCACATTTGTTAAGAAAAGAATAGTGATGTAATCATGATT
TCAATTTTTAAATCATGATGGCCATTTCACCTTTCTTGCCTTAACTGGCTTAAATCTAATCTTATTAATGAGTCT
AAGATCTATTTCTCTACAATCTGTAACGGAGGTATCATTGATTTTAAGGCTTCTTGGAAAGTATTGATTACCTTG

TTCACGTGATAGTTCTACTGTATAACTAATACCATTACTTAATTTAAAGACTACAAAATAAATATTATCTCTTCT
AAATCGGCTACCAACTCTTCTATTCAAAATGCCATCAGTTGTATAGAATTTACACTCCATTACATGTAACATTTG
ATAAAGCAACAAGGTTCCAACTATTTTGAATTTATTTTCGTTGAACCAACCATTATCAGCTTTATTCAGGCTTTC
CAATGTCCAATCATCTTTTAATCCTTCAAAAATACTCCCTGCATTATTAGTTGGTAAATTAACATGATGTTCTCT
AGATAATCCTAATAGATGAGGAATGGAGGAGTTTGGGACTGATAGAATAATTTCAGGACAGTTCTCCATATTTGT
CTGATAAATATATTTGTACTGAGAAACATTTTTCTCAATAGTATTGGCCAAGCAATTCATCTTAATATTGAGAAT
ATCAATCTCTCCATCAAAAGTCTTTAGCCATTCCAAAGTATAAGTCATTTGAATTTTCTCCTAAAAAAGAGAAGG
AAGCTCCTGCTTCCTTCTCTCCCACGTATTGAGTTTCACCTCACGCTAGATATGCCCTATCTATTCCTCAAGGGT
AGGCAGTCCTGCTATTCTTTTTCTCAACAAACAGTCTGAACTGAGAGCGGAGCCCACAGGCGGGTGCAGACCATA
TCCACTAGCCCACAAGTAATTATATCATTTAATTATCTTCATTATACGCAATTAGTCCATATTTGTCAACAATTA
CATTCATTATGAATATCTAAAAATCAGGTCACCTATTTTAAAAAAGCAGCAAACTATAAACTAGTAGGTTCCACA
CCAAATGTAGCCCCATACTGCCCCATAAGTCCGATTTGTAGCGTACAAGCCCTAAAAACATCCCAAGTGAAACAT
ATAAACACCAAGCTAGAATGGTTCCTGGATGATGTGCTAAGGCAAATAAAACACTTGTCAAAGCAACTCGAATAT
CTAATTTTCTAACCAAGTTCCATAAAATTTCTCGATACAGAAATTCTTCAACCATACTCGCATTGATTAAGAACA
ATAAAAATGAAAACCAAGGGACTTGATGTTGAAGGCCAATTAAGTTTGCTTGATTCGTAGTTCCTTGAGCATGGA
TTAGGCTAAAACATAGACTTATAATCAGTAGGCTAACGAATCCAATACCAAGCCATTTCATCCTAGATTTCATAT
TGACCTTATGAGCTTGTTTGCGTTGACCATACATCCATAAAAAAGAAATGAGTGACGAACTATAGAGAATCTGTA
GTATAGTTAACTCACCGATACAAAGAAATTTCAATAAGTATAGAGATACCAATAGGACATTTACTTGTTGGAATA
TATAAACTGGAATTATTCTTTTCATAGTTACCTCCGAAATAAATCTTCATAATCTAAATCTAATACCTGCACAAT
CCTTTCTACCCATGGACTTTGAGGCATTCGTTGTTCCATCTTGTAGTGACGAATCTTTTGATACAAACGATTCAA
TTCACTTGGATAGTGAAACTCTCCCGCAAACATTTTTCTGGTTAACTCAATCCAGCTAATATTTCTTTCAGCTAA
AATAATGGACAAGTTCTCCCAAAATCGTTCAGCCATATTGCTTCTCCTTTAGTTAGATAAATAATGTGTTTGTGC
CATGTAAATCAATTGTTTCGTATCTCTTGGCAATAGAGCTCTAGCCTCTTCCAAATTCAGATTTGGATAAACTCT
CTTATTTGAAACTGCAAGAGGAAGTCTGATGCTTAGTTCAGGATTTTTTAATATCATTTCGATGAAATCCGTTAA
TCTTAGATTGTCACGGTTCTTAAATCGTAATAAATTAGGAGATAAAAACTCAAAACAATCTGAAGAATAGCTCAT
CATCTCAATTAATTTGTCCTTTGTCATTTCAGAAATCGAATGACAAGATACCTCTATTCCGTAGTTTTGAAAGAA
ATCTAAAAGAATTTGATTTCTATGGTTATTTTTACTTAGATAGAGATCAATCATGGGAGACCTCCCAAAGATTCG
GTTCCATTTGATATTCTGAGACGATTAAGGAATCTAATAGATCTGAGAAGTTAATTGATTTCTTGTCTTCATCAT
AGGCTTTTACAGTTACTTGGGTTGTAAGTATTCCCTCTTTTCCCTCGGCTCGATAGCCTTGTCCATATAAAACAA
AAACAAGATTCTGATTATCATCTATAAAGGCATCAGCTCCGTTCTTTATATCCTGACTTTCGAGGAATTCCATAA
CGTTTTGAAGATAGGATTCATAAAATAGTGGATAGTTATGTTTTTTATGGTAATCATCTAAAAATGTCACTTCAA
ACTCACATAGATAATTGGGCATTAAAAATATTTGTTCATCCAGCTGTTTGATTTCTGCATCATGTAATTCTGTTT
CTAATTCGTCACAATCTAGTATTGACTCTTTATTTAAAGCTTTCATCTTTTTCCTCTATTTCTTTTAATTTCTTT
GCGATTGCAGCAATCACAGGAACGGTTACACTATTACCAGCTTGTTTATAGAGCTGACTATTACTAGAGACTTTT
CTAGCAGCTTCAAAAGCCCAATCAGGAAACCCCTGCAATCGAAAACACTCTTTAGGAGTGATTCGTCGTATTCTC
AAACGGTAAAATTGTCCATTAATTAAAACACCGGCTACATGGTAAACTTGCTTATCTTCTCCTTCATAGCTAGCC
ACTACTACTCCCATTTGACCACTAGTTGTCAACGTATTCGCTATACCTTTTCCAACTCTACCACGCCGATACTGA
GAACTTGGTCTTTCTAAATTGATTGAATCTCCAATCTCTGCTTGAGCGTACCCTTTTTTAGTTGCTTCCCTGATT
TTTAGAAAATGGATTGGTTCTGGGATCAGTATTTTGGGAATTTTATCTCCACCTTGCATCGTAGTTAGTGTTGGA
GATAACCCATCACTTCCATATACGCGACCAGTCTCCTTAAAGCTAGTTGGTAAATCTCCAACAACAACAATACCA
TGGAGATCCTGAGTATTCAAAGTAAACATTGGCTCTTGGTTTTCCTTAAATCGTCTACCATTTTGTCTTTTTTCT
AATCTGTCTGGTGTCATACAAGGAATCGCAACTTTAAATCCTTCTCCTTTTCCACGAACTAATGTTGGCGCTAAC
CCCTCTGAATAATAGACTTTACCGCTCATGCCACTTTTTGATGGATTCAAATTTCCTAGTGTTTTTAAAGTCTCA
GAGTTAGTTGCTTGACCTTCTCGTCTGAAAGGAAATAGGAGTCGGGTACCTTTCTTTCTAGAATGTCCGATAATA
AACACCCTCTCTCTGTTTTGGGGAACGCCAAAATCCTTACTGTTAAGCACCTGCCACTCAACATCAAACCCCAAC
TCATCAAGTGTGGTAAGGATTGTGGTGAACGTCCGTCCCTTATCGTGATTGAGTAAGCCTTTAACATTTTCAAGA
AAAAGAAAACGTGGTTGGATTTGTTTGGCCGCCCGAGCAATTTCGAAGAACAAAGTTCCTCTAATATCTTCAAAT
CCCAATCGTCTTCCTGCGATTGAAAATGCTTGACAAGGGAATCCCCCACAGATGATATCGACTTTCCCTCTAAGT
TTTTTAAATTCGTCATCTGAAACATCTCGTATGTCATGAAATTCGATTTCTCCTTCCGTTTGAAAAATGGATTTA
TAAGATTCTCTAGCAAATTTATCAATCTCACAAAATCCAATACACTCGTGTCCTACACTTTCCATTCCAAGTCGA
AAACCACCTATACCTGAAAATAAATCAATAAATCTCATTTCTTGATTACCCCTTTCTGATCGACTATCAATATCC
AAAGTGTCACACCACTTGAAATGAAAATGAGCAAAACTAATATCCACTCGATTGACGACATCTAACCACCTCCTT
TTCTAACACGGTTATTCCAAACAGAATACAGGACATTAAACACAAATTCAGCAAGTACTTCCGCTCTTCTAACAA
AGCGAACATTGTCAAGAGCATACTGATAGATTCTCTCAAAATCAGTCATAGCAATTTCACTGGCTGTTTCAGAAA
AACCTTCTCGTCTAAATTGATCTTGTACCAATCCCCAAATATAATCTTGATCATATTTTGTGACCTTTTCTACTT
TTCTTTTCAAGATAGGTTGAGTATACCTCTCCTCCTCATCCTCAATAAATAAAGAATCAGTCTCACTATATTTAG
TCTCACTAACTTCAGTCTCACTAGGGGCTGAATGTGAGACGGGGGCCGTTTCATTTTCATCCTGCCCTAGTCTTT

TTTTAACACTAGGCCTGTTTGAATTAGCTACTGGGGTAGAAGACAATTCCCCTAAATAAATCTTATTAGCAAGTC
TCCCTTTCTCACTTGAAGACTGTTGAACTTCATCAATTAAGTCATATTCTTTAAGAATTTTTTTGATAGACAGTA
ATTTTGACTTAGAACAACCTAACAACTTCATCAGTTTAGAATTAGAAAATACTAAATAAACCGCCCCTTCTTCAT
CTATCCAACCACGACTGAGAGATAATTCTAAACGATCTTTTAAAATAGAATAAGCCACCTTTACTTCTAGTTTCA
TATCCATATATTTTTCATCCTCAAAAAGAATTTTAGGTAATTTATAATACCGTTCTGAAGTATGGTATTGATTTG
CGGTAATTCGTTTCATAGAGCTCCTCCTAGTTCTTTGAGTATTAAATCTCCACTTGATTCCAATCGAACCAAGCC
ACTTTTTTCTAATTCTGCCATAAGAGAGATGGCTTCAACAATATCAATTCCCATTTCACGTACTAAAAATGAAAT
GACAATATAGCGTGATGATTGTAATTCTTTTGTCAT

C. The *tet*(32)containing genomic island from strain *S. pneumoniae* 131016 (accession number; JABAHC000000000)

TCTCAACGTGCTCATCATTGTCCAAACCTATGTTGAGATTTTCTTCTATAATCGGAAGCTTGAAAGTAATTGATT
TTAGCCATTGTCCGTTAGGCTGTTTTTCTTCATAAACTTGAATTTCAGAAATCAAAGCTGTAATTAACTGTCTAC
GCTCTACATCATTCATGACTTTATAGAGCTTATCAAAATAGATCAGAACCTTATATATGTTATCTCCTGTAAGCT
TTTCAGCTTCAATAGTCTGTTTCTTTGCTTTCGCATCAATTAGTGATGATTCTAATTCATCTATTTTGTCATACA
TGCGATAAAGTCTGTCGTCTAAATCCTGTTTCCTTCTCTTATAGTGCTTATCTTCAACATCTAAATTATCTATTT
CCTCAATTAGCTTAAACTTTGTAGAATGACACTTCCTCAATTCCTTTTGGTAATTATCTATTTCTGTTTCTATTT
CAGAGGTATCCACCTTCATGTTGATTTTCTCTTGCATCATAGAAGCAAATTTCGGATTACTTACAATCTTGACAA
TTACCTCTGCAACAGCATCATCTAACAATTCTTCTCTAATTTGCTTACGGAAAGTACACTTATGACCTCTTATCA
TCTGCCTATGTTTACAACCATAGTAATAAAAATCTTTATACTTTGTGCCATCTTTCTTTTTCTTGATACACTTGT
TTCCAAACATTCCCACTCCACATATCGGGCATTTTACAATTCCAGAAAGCAAGTGTGTGCGTGTATCTTTTCCTT
TATTCACATGCTCATATTTCTTTGCTTGAGATTTTAGCTTAACCTGAGCAGCTTGCCAAACTTCATCGGAAACTA
TAGCTTCATGTATCCCTTCAGATATTAGATACTCATCTTGTTCAACCTGCCTATATTCATTTCTTGTACCATGAA
CTTTTTCTAAAGTTCTTCTTCCAAATGCTATTTTCCCATTATATACAGGATTCTTTAATATCTTTCTTATAAGAC
CTGCATCAAACAAAGGATTCTTACCATTCTGTCTTGGGATTTTTCTAATTCCATGATTCTCTAAGTATTTAGATA
TCCCATTGGCTCCTATCGTAGTATTTACATACTGGTCGAAAATCGTTCTTATGGCAATTGCCTCTTCCTCATTTA
TAAATAGCTTGCCATCTTCAAGTTTATATCCATACGGAGCAAAGCCGCCATTCCACTTTCCTTCCCTTGCTTTTT
GAATGCGACCTTCCATTGTTTGAATACGTATGTTTCTCTTTCTATTTCAGCCACAGCTGATAAAACAGAAATCA
TTAGTTTCCCGGCATCTTTAGATGAATCAATGCCATCTTCAACGCAAATAAGATTAACTCCATAATCCTGCATTA
TCTGAAGTGTAGAAAGAACATCAGCGGCATTTCTTGCAAATCTTGATAACTTAAACACAAGAACAAAAGACACTC
CATCTTTTCCAGATTTTATATCTTCCATCATTCGATTGAACTGAATTCTACCTTCAATAGACTTGCCAGACTTTC
CGGCATCTTCATACTCTCCAACAATTTCATAATCGTTGTAAATAGCAAAAGCTTTCATTCTTGATTTTTGTGCCT
CTAACGAATACCCCTCTATCTGTATTGACGTAGATACTCGTGTATAGAGGTATACTTTTATTTTTTCTTTTGACA
TAGCATTAACCTCAATATAATTTTTCTATATCATATATAAATTTTTGAATTTAAGTTTGGACTATCATTTCAAGT
ATATTATAACACCTTTATTAGTCCGCCTCAATTAGTGTTTTTGCCATGTCAAAACTATTTTTAATCTCTTGATTT
TTTGCTGGCGTTGGATCGGGCAGATTATCTAAATCTAAAACACCAGCATATTTTGTAATCAGATTTGCTATTAAA
TCAGCCAATCCATTCCAGTCACTATCCACAGACACCCCTCCTTCCTTTACAAACTTTCTATAATCAAAATATGTT
TTCTTCTATATTTTAAGTTTAACGACTTTGGTAGGTGCTTGGCAGCAACATAGGAATCTCACCTCCGCCTCTTAT
TCCAGACGAGCCGGCTTAAACTATTGAAGTATCATTATCACTACTTGCTTCATTGAACAGGTTGCCAAATCTGTT
TTTATGTATTCTTATTTATCGCTCGCTTTCTTGTTTCCTTGACTCACTCAGTATTCATTAAACCGAAATTATTCT
CAGCAGAAAGGTCATGGCGTAAGTCATAATCCTCCACAAGTAGATAAAGTCCTACCTTGGTCTATTCAGTTTTCA
AGGAACAATAGCCTAACGGCTTTAAGAGAGATTCTCTTCAACTTTATGGTTTTCATCACTTCCTTAAATAAAATA
TAAGGACAGATCTCCCTTCAATTTATAAAGGAAAATCTGCCCCCATCTACAAGGCGTTTACTTTATAAATTCTTT
CAAAAGCACCTTTAGCTTTTTAAGAATATTGTTTTTTCGCCATTGTATAGCTTGATAGCTCACTTTCTTACCTCT
CGCTATGCTTGATAGCGTTTCATCATTGAAATACAAACGCTCTATGATGTCCCTTTCCTCATCGTTTAGTTTTGA
TATAGCATTTCTGACTGCCTCAATCATCATCTGTGTTTCAACAATCTTTTCTACATCAACGCTTTCATCAGCAAG
GTTATCTATAAAATGTCCATCATGATCCAATGATGAAAAAAAGAGCAAGTGGTTTTTCTTGTCCACCTGCTCTAA
ATACTTTTCGTGTTCTTTTTCTCGCCAGTAGACTTTATAAATATCTTCACTGACTTTTACCTTTTGCCCTCTGAC
ATAAAGGTAATACTCTTTTACCATAAAGTTTCCTCCATTTCTTTTTTGTCTATTTGTTTTTCAGACAAAAAAGGA
GGACTCCTGCATCTTGGCATAAGAAGTCCTCTGAAATGAAAGAAACCTGTTCTTTCTTTTACTATATTTAATTGT
TTAGACGAGAATATGGAAAGACAGACAACTGTAAATCTTATAAGAAAAAGGAAGGATTTTGTTTTGAGTTATAAC
TTCAAAGAAATATATAGCCTTTATCATATCCTGTCCCTCCATTTTACAACTAACTATTCCTCATAAATTAAGCCC
TTCTACTATTTCAGGTATCCACGCACATAATAAAAAATCTTATGCTTGTACTTTTTCTTTGCTCATCTTATTTAG
TTCTCCAAGCATCATGAAGATTCCAACAACAATCATAATGAAATCAACAAGGGGCTGTGTGAACCAAACAGCTTT
TACTCCAAATACCATTGGCAGAAGAATCATTGCAGGAACAAATAGGAATAGCTGTCTAAGCATAACAATAATCCC
CGCTTTCTTTCCGTTCCCAATAGATTGGAAAAATGTAATGCTCATGACCATTACTCCATATAAAATAAATACGGA
ATAGAACAGTCTGAAATTCCCAACTCCCTGTGTTATGATACTTGCTTCTACTCCAAATAAGGAAAGTATCTGACT
TGATAACAGCAATGATGGAATCCAAAAGACTGCTGCAAGAACAAGTCCTCCGATAGAAAATACCTTCATTGCTTG
CCTTACTCTGTAATATTGCTTTGCTCCGAAGTTTGTTCCGACAACAGGCTGCAATCCTTGACTCATTCCCCAAAG
TGGAATAAACGAAAAGGCATATACACGAAGCGATGCTGCCATCAAAATTCCGTTTGGATCTCCGCCATACTTAAA
TGCCATTTTATAAAGCATAGTCTGTTGAATCATGAACAGAAGCTGCATCATCATAGCTGATGAGCCTACTCCAAA
CATTTCTTTTTTTATTGCTTCATCGGACTTAATCTTATGAATTTTAACGACTTTACTCTTTTTCAGGAAGTAGTG
CAGCGTTACAACCGCCTGAACAAACTGTGCTGTAATCGTTGCAAGGGCAGCACCTTCAATGGCGTATTCACCCAT
AACTGTCATTAAAATCGGATCAAGAATGATATTCAGTAAAGCTCCAAGTCCCATTATCATCATGGCTTTTTTCAT
TAGACCTTCGCCACGCATAACCATGTTTGCGGACTGTGTAAAATTTACAAAGAGGGAACCGATAAAGATTACTCT
TAGGTATCTGATACCATAAGCTTTGATCTCACCTGTTGCACCGACCATATCTAAAAAATGTGGTGCAAGTAGTAT
TCCGCCAACTGTTATGATCGCCGAAAACAAAATCACCCAGAAAATCAGGTTGCCCATGATTTTATCTACGGTGTT
CTGATCTCCTTTACCAATCGCTCGTGATAAAACAGATGCAGAACCTACACCAAGAAGTGTAGACACCCCGCTGTT
AAAAAAAGTAAGTGGCATCGCAACGCCGCAGGCAGTCATTGCAGTTTGCCCTATAATATTTCCCGCAAAGATTCC

ATCCATTAGTGGATAAAGACCTATTACTATCATTCCGATAACCGCCGGAATAGATAGCTGAAATAGTAAATCTAT
TGGTCTTTTGATCAATAGTTGTTCTTTCATATCTTGTTTCATTCTTTGTCCTCCCTTGTTTTGATTTATATTTTT
ATAGGCAATTATTTGTTTGATTGAAAAATTCCTTAATTGCCTTTATATTTTTTTCATTCTTACCACCGTGCCATT
TTAATTTTCCATTCTCCATAAACATAAAATAATCACAACAACATTCTACTAATTCCGGATCATGTGTAATAATAA
AAATAGTTTTTCCGTCCTCTTTAAGCCTCATCATTTCATTAGAAACTTCTTTCATATTCTTAAAATCCAAACCAC
TTGTAGGTTCGTCCAAAATCAGTATTGTCCTATCAGAAGCGATAGCACTTGCAATTGCAACCCTTTGTTTTTGCC
CTCCGGATAATGACATTGGATGGCAATCTATAAAATCCATTAAATTTAACTTTTTACATATTTCATCGACCTTTT
CTTCTTTATTTTCATCGTTCTCTATACTCAAAAGAATTTCTTCTTTAACTGATTCGGTAAATAATTGGTGATTAA
CATCCTGCATCACCATAAAACATTTTTTCAATCTTTCTTTTGTATCATAATTTTTTGTTTCTATCTTCAAGGTTC
CTTTTGCTTTTTCTTCAACTCCACATAAGCATTTTGAAAAAGTTGATTTTCCAGAACCATTATTTCCTAAAACAG
CCACAATAGATTTTTGAGGTAATACAGCATCATGAATTTCAAGAAACGGTTTTTTCCCATAACTAAACATAAAAT
CCTTAATCTCTATTTGTTCCTTAGATTCTATATTTTTCATACGGCTATTGGTGTCAATAGTGTGTATACTCCTTA
ATCCCATATCATTTAAAGTGCTATTAGACAATTCTTTGAACTCAACACAAGAAAATTCTTTGTTAATTTCCCCAT
CTTTCATATAAATAAATCTATCTGCAATATCTATTAAATATTGTAGTCTATGCTCTGCAATAACCACAGTTGACT
TTTTTTCTTTCCATTTTTTTATGATATCTTTTAACATTTTTATAGTTTGTATATCTAAATTAGAAGATGGCTCAT
CTAATACATAGATTTCAGGCTCCATAACAGCAGCAGAAGCACAAGCAATTTTTTGTTTTTCTCCTCCTGACATAG
AAAACAAACTCTTATCAAGCAAATCTTTTATATTTAATTCTTTTGTTACCTTGCCAATTCTTCTACAAATTTCTT
CTCTTGAAATCCCAAAATTTTCACAGGCGAATGCAATTTCACTTGTTGAATCTACATTGAAAAATTGAGTTCTTG
GATTTTGAAATACTGATCCTACATATTGACTCAATTCATTAATAGAGTTTTTAGAAATTTCATGTTCATTTACAA
TAACGCTACCAGTCAAATTTCCGGAATAATACCCGGGAATTAACCCATTTATCAACCTACTTATTGTAGTTTTTC
CGCAACCGGACTCACCACAAAATACAACTACTTCACCAGCATTGATATTTAAATTAATATTATAAATTCCAGCAT
TCTTATTCCCGCTATCATAAGTAAATGACACGTCTTTAAATTCAATCAATGCTATCCCCTCCTATATAAAAATAG
TCCTGTAAAGGCAATTGAAAAGAGTAAAATGAGCAAATCATATATAGTAAACTTTACTTTTACTACAGTAGTTCT
CTTTTTCAAAGCTCCCAATCCTCTACTTATTGCCGCCGCCGACAATTCATCACCTATTTTTGCAATAGAAACGAT
TAAAGGTATTGTTACATATTCCAAGTATAAGCTTGGATTTTTCCACGCCTTTTTATTTTTAAAACTAATTCCTCT
CATTTTCATAGCAGTCTTAATAGAGTTGTGTTCTTCTTTCAAAGTTGGAATAAACCTGAATGCAACTGCTGTTGG
AATAATAAATGCATTGGGAATTTTCCACTTTACCATGGCTGCTATAAACTCGCTCGTTTTTGTGGACATTACAAC
ATAATATCCCAACATAAAAATTGGAAATAGTCTTAAAATTAAGGCATTCAACAATACTGAAATTGTATTTATGAG
TGTAGGCAATGTGTACATATCTTTCGTAAAGTGTGCCATAATAGCCAAGGTAAATAGCCCTCCATAAATGAAAGA
TATCTTAAATTGTTTATTAGATAATAATAATGCTATTGCCACAATGGCGGATATAAGGCTAACTAAAATATCCTT
ATATCCGACAGACATTATGGTTGTTATAAATATCATAAATAAAATTTTAGTACGAGGATCTAAGAAAAAGCCCTT
TGGTTTATTCGGTTTGTATACCTCATACTCTTCCATTAAATGATACCCGCTCTCTCAAAGTGTTTCTTAAGCATT
TTCCTCCCTAAATTTGCACCAATAACTGAGCCGATAAAAATAATTCCAAATGCAGCAATCCCCATCCAAGAAGGC
ATATATTTTGCGAGTGCAAGTGCATATTGTTCTCCCATTTGTTGCTTAACACCTTCCATATAGGTATCTGCCATT
ACCCACATTGGCATTTGACAACCTATCATTCCGCAAGAAAATAGCCAAAATCCAATAACATTCTTTTTAAAACTC
TTAAAACCACCTGATTTCATAACCAGATCACTGATAATTCCAAATGGAATATATCCCACTAACGCTAACCATGTA
TAGCCCATAAAATACCAAAACACACCGATAATAATTGACATAATCGTAACCATGCCAAATTTTTCTACTTTAGTT
AAAAACAACATAAAGGGAATTCCTGTGACTATAGGAATTAGAAGATAGATAGCTGGGTATAAGATAGGTATTGCA
TTCATCATCCCTATTACAAAAAACAGAACTAAATAAATAGCTGTATAAATTCCTACATTGATTAGATCTTTAGCT
GACAATCCCTTTTTCATTTTTTTTAATCCTCCTTAAATTAAAATAATATAAATCTACATGAGCTATATTTGAATA
ATTCTCATATAAATCATTTTCACTTATGACAGTTTCCAATTAACTGCCTTTTGTCGTGCCTCAACAAAATTTTTA
TAGATACCTTCTGCTTGAATTAGCTCTTCATGTGTGCCTTTTTGATTTATTTTTCCATTTGCTAAAACAAGTATT
TGGTTTGCATTTTTTATTGTCTTTAGTCTGTGTGCTATCATTATTACTGTTTTATTTCTTGTGAGTTCCTCCATA
GCAACCTGCAATTTATCCTCATTTTCAGGGTCTACATTTGCAGTAGCTTCATCGAAAATAATAATAGGTGCATTC
TTTATCATTGCTCTTGCAATTGATATTCTTTGTCTTTCTCCACCAGATAAACTTGCACCTCCCTCTCCTATAACT
GTTTCATATTGTTCAGGAAGCTTCATAATAAATTCATGACAACACGCTTTCTTTGCAGCTTCTATAACTTCATCA
TGTGTAGCATCTGGCTTGGCAAACTTTATATTGTTCTCAATAGTGTCTTGAAATAAATAAACATTTGAAAAACC
ATACTAATTTGTCTCATTAATGACTCCAATGAATATTCTTTAATATTATGTTTACCTATGCTGATTTCTCCTTTC
TCTACATCCCAAAATCTCGCTATCAAATTGCAAAATGTAGTTTTTCCAGCTCCTGAAGGTCCAACTATAGCTGTC
ATTTGGTTTTGTGGTATCTTGACAGAAATATCATCAAGGATTTTTTTGCCTGAATAAGAAAACTCTACATTATCA
AAAGAAATATCGAAAGTAGTAGGCTTTATGTCTTTTCCATCAATATCCATCTGTGGAAATTTCTCTAACTTCTTT
GTTTGCTCAATAGATCCACTAACAACTCTGAGAGAAGTTGTTGCACTTCCTGCCAATTTTATTTGAGAAAAAGCA
AGAAAAGAAATTATAATCGTCATAACTGTATTTAAGAATGAGAGTTCTCCATTGATATAAAAGAAAATACCTGCA
CCTATTATCAAAATGCTAAACAAATCCAAAAGAATATTTTGCAAAATCGTATAAGGTGTAAACAGTTTTTCACAA
TCAAGATTTATTTTTCTACTATTTTCAATAGCACCTCTGACTTTTTCATCTCCTTTCCCTGTTAAATTGAAGGAT
TTTATAACAGACATTCCTCCAATTTGTTCTAAAATGGCATCAACTAATTTAGCAGATGCTTTTTGTCTTTTAGGA
AGAACACTCCTTGATTTGCTTTCCATTTTTGAGAGAACAAATAAGTACAATAGGCATCCAATTGCAACAATAAAA
CCTATTCTCCACTCAAATGCTAAAATCATTATTGTAAATATTATCGTGTTTATAAAACCTGATAATGTATTTACC
ATAACCATTGCTGCGGTATTTTCTACATCTTCTAAAACAGTTGTAGAAATTCCAACCACTTCACCAATATTGTTT
TCATTGAAAAATCCCATAGGTATTTTTTTTAACATTTCACCTATTGCTACTCTTTTATTTGCCACCATGAAATAA
CTTGCATGGCATTGCTGTAATTGTGAAAAATAATTTGTAACACTTCTTCCGATTATGCTAATAACAAGAAGTATT

AACGCGATCCACGCGGGCATCATGCTCATGTCTTTTTGGACAATTGCTTTCACTATAAAATAAATAGCACTGATT
TGAAGCATATGAAAAATCGCAAACAAAAAACTTACCCATATAGAGTGATATACATTTTTCTTTTCATCACCTGAG
AAATCTAAAATCTTTTTTAAAGCACTTAACATTTCATCCCACCATCCTTTGTTCCTATATGAGCGTTCCACATAT
TTCGGTATAATTCACAACCTTTTAATAATTCATCATGCTTACCGTATGAAACAAGTTTTCCATTCTCAATTAAGA
ATATTTGTTCTGCATCTGTTATAGTTGATAACCTATGAGCAATAATAATCACCGTTTTATCTTTTATAAGCTTTG
ATAAAGCTTGCTTTATAATCACTTCATTTTCAGGATCTATATATGAAGTAGCTTCATCAAGTATTACAATAGGTG
CATTTTTGAGCATTGCTCTCGCTATTGATATTCTTTGCCTTTCTCCACCAGAAACATGGCTCCCACTACTTCCGA
CAACCGTATCGTAACCATGTTCCATTTTCATAATAAAATCATGACAACCCGATTTCTTAGCAATGTCTTCTACTT
CTTTATCACTGGCACTTGGATTACCCATTCGGATATTTTCCCTAATACTTTCGTTGAACAAAAAATTATCCTGCG
AAACAAAGGCAGTTAAACTATATAATTGCTTTAATGGAATTTCATTAAGATTGTATCCACCAATATTTATATTTC
CTTCCGTAATATCCCAATATCCAGCTATTAATTTTGCTAAAGTTGACTTGCCACTTCCACTTGGTCCAACAAAAG
CCACTGTTGTCCCTTCTTTTATGTTTAGGGATATTCCATGAAGAATTTCTTTTTCTTCTTCATATCCAAACTTTA
CATTTTGTAAATCAATATTATATTGCTGGATAGTTACTTCTCTATCAGAATGCTTTTGTTCTTTTCCTTCTAAAA
TTAAGTTAATAGAATTAGCAATAGTTCCTATCTTAGCAAGACCATCAACAAAATTGATTGTTTCAAGTAAAGGTC
CTGCAATACCTAAAGATAGAATAATTACAGATATAAATACTTCTGCACTTAAACTGCCGCTAATGTAGAAATACC
AACCAAATGGAAGCACTGTTATCATTGTAGTAGGAGATATATTTTTAGATAAAGATACAGGTAACTGACAGCTCT
TCATCCATTCATAAAAGTATCTTGCATTTGCAATGACTTTATCTTTGTACTTTGCGTACGATCTCTTGTCTTGAT
TAAATGTTTTTATCACTTCTATTCCATTGACGTACTCAATAATTGCAGAATTCATTTCTCGATTTACCTTTACAG
ATCCTTCATATTGAACAGCATAATTTTTCATTACGAGTCCCATAAAAAGCATTCCCACAGGAATGGATACTAATG
ATAAAAGAGCCATTCTCCAATCAAGAAATAATAAGTAAATAAATATACTCAAAGAACCTAAGAGATTCGATGTCA
TTTCAGGCAACAGGTGAGCGAGCGGTTTCTCCATACTTTCAACCTGATCCACTATAATCTGCTTAAAGTTTCCGC
TTGGAACAGAGATTATTTCTCCTAATGGCATCTTTGGAAGTTTTTCAAGCATTCTTAAACGAACATCTTTCAGTA
CACTAAAAGTTGCTTTATGGGATACAGATAAAGCCATAGAGTATAAAAATGATTTTAAGATGTAGGATAACAATC
CTATTCCTAACCATAAAGAATAAATTTTAAAATCCTCATTTCCCTTTAACAATTCAACTAAAATACGACTTGCTG
CAATATATGGAATAATCCCTGCAATAACACCGATACTCGCTATAAATACAGATGTTTTTAACTTTGAATGTTCCT
TTTTTGCAAGCTCCCACAATATAGTAGCAGGACTTTTTTGTTTCATAATCAACACCTCCAAATAAAATAACTTAA
TTAGCTAACTTAGTTATCTTTATTTTAAAAAATATAAGGGCGCTTAATACCCTTTCCCTTATATTTAAAAACCTC
GTAATTTCTCCCATCCATCATAAAAAAACTTCGAAACTATTCTTGTGTTTAACTTTACTTCTTCTTCTGTTTGAG
AATGTATTATAATTTCACACAATGCATTAATATAAGCATGGTTTAACAAATGCATTGCATTTTTAGGGACTTCTG
ATTTTATATTCTTTTTCTTTGAAATAATATGAAACGCTTTATGTCTATTTATATCTTCCTTTTCCACAAGAAGCT
CTATATAATTATCATATTTCGTACCATATGATTTATATATTAAAAGATTAAATAAATCTTTTTTGCTAAACATAT
ATAATGCAGATTCAATTGACCCTTCAATGTTAATTTTTGATAAATCTTCTCCACAATTTTTTTCTATATTTTGAA
AAGATTCCTCTTCAAACTTGTTATAAAAACCCAAAAGTTCTTTTGCCAATGGATCAACTAAACTGATAAATAAAT
CTTCTTTGTCATTAAAATGCCTGTAAAAAGCACCCGTTGTAACTCCAGCATCTTTACAAATTTTTCTTAAATTAG
CTCGTTCATATCCATCCTTCAAAAAATTCGCTTTACCACTATCCATGATTTTTTTATGTGTAAGCTCATATCCAT
TCAAATCTGCCATTACTCTGCCTACTTTCTCTTTACTGATCAATTTCCCGCCTCATCTTGATAACTTAGTTATCA
TTTTAATATTGTCGATCATGTATGTCAAGTATTTTCTGGAACTCTAATAAAAAAAATGGTACAATAAATACAAAT
AGTTTTATACACGCTTAATGCTTGCTACTCTAATTATTCTTTTGTAGCTTTTGTAGCTTTTGTAGCAAGCACAGT
AAGTGTACGGACACTTACGGAAAATTGACGAAGCAGCCCTTGGGGATCTGCTTCTTTTTTTATCAATTTTTAGAT
TGTTAGGGAGAACCCTAAGACCCCGAAATACATTCAAAGGAGGAACTAACTATGGCAAATAGATTAAGAAACGAA
AGACTTGAAATTAAACTAACTGAAGAAGAAAAGGCTCTTTTTGAAGAGAAAAAAAGACTTGCGAAGTGTAGAAAC
ATGAGCCATTTCATCCGCAAATGCGTTTTGGAAAAGGAAATTTATCAAGTGGATTTAGAGCCTTTCAGAGATTTA
CAAGGCTTACTTTCTAATGCAACAAACAACATCAATCAGATTGCAAAGCGAGTAAATTCGACAGGTGTAATCTAC
AAAGAGGACATAGGTGATATAAAAAAAGAGATTGAACATTTCTCAAAAGAGCTGTGGCAAATTCATTCACTACTT
CTGAAAAGAACATCTGAAACGGAAGGTGAATAATAATGGCTATTACAAAAATACACCCAATAAAATCGACTCTTA
ATCTTGCTATCGACTACATTGTAAATGGAGATAAAACAGACGAGCAGCTTTTAGTAAGCACTCATAAATGCCACG
AATCAACTGCTCACACTCAGTTTTTAAGGACACGAAATGACGCAGGAACAAAAGGAACCGTTCTTGCAAGACATC
TCATTCAATCCTTTTTACCGGGAGAAACAAGCCCTGAATTGGCTCACCAGATTGGTATGGAGCTGTGTAAAAAGA
TACTCAAAGAGGAGTACGAATTTGTCTTATCTACTCACGTAGATAAGGGGCATATCCACAATCACATCATCTTCA
ATAATGTAAATATGGTAACAGGTAGGTGCTACCAGTCTAACAAGAAAAGTTACCACCAAATCCGTTATCAGAGTG
ATAAACTCTGCAAAGAAAATAACCTATCCGTTATTGACGAGTTTTACGAAAGCTATAAGAAAAAAATACAAGACTA
ACGGTAAATCTTGGTATGAAAATGAACAGGCAAAACATGGCACTTCTTGGAAAAGCAGGCTTCAATTTGACATTG
ACAGAATGATAAAACAGTCAAAGGACTGGGACGAATTTTTAAAGAAGATGGCTGATCTTGGCTATGAAATCAAAT
ACGGTAAGCACATTGCTTTTAAGCCAAAAGATAAGGCGAGATTTACAAGGACTAAAACAATCGGAGAAGATTATA
CCGAAGAAAGATTAAAAGACCGCATTGCAGAAAGAGAGTTTATCAAGACTCCTGCCGTCAAAAAACGCATCAGCA
ATGTTATTGACATGAACACCAATGCAAAGGTAAAGGAAAGCAAAGGCTACGAATATTGGGCAACCAAACATAACC
TTCATACAATGGCTGAGTCTGTTATTTATATCAGAGAACATGGCATTAAATCCGTTAAACAGCTTGACGAGTATA
TTCAAAAAGCAGCTGATGAAAGGCAAAATATACAAGAGAAAATCAAGTCTATTGATAAGGAAATGCAGAAGCTTT
CCACCACTATGGAGCAAGTTCATACCGTTAAAAAATACAGAGCGTGCTACAAGGAATATACTGCTAATCCGTCTG
ACAAGGCATTTTTTGAAGAGTACAAAGCTCAGATTACCCTATATGAAAATGCTCTCTCAGAGCTTAAAAAATCCT
ATTCCAAACTCCCAAATTCAAAGGATATTTTAGCTGAACTTGATAAATTACAAGAAAAAAAGAACACCCTTATGC

AAGAGTATTCTTCCTCAAAATCCACTATGGACGAGCTTTATAAGATACGAAAAAATTACGGAATCTACATGGGTA
AGGAGATGGAGAGATAATCTTTATCTCCTTTTTTCGCAAATAAAAAAAGAGTCGGTGCAGTCCCAAGACCACACC
GACCATAAATTTATCTCTCAGCTTCTTTTGATGTTTCTTTCTTTTCTTTAGGCTTTTCCTTATCTTCAGCCTGAT
ATTTTTTGATAGCTCCAAGCACAGACTCTTTCTTTTCATCTCGACCTTTCATCTGTTCTTTTGCCTTTAAAAGCT
TTGAAGAAAGTATCCTGATGTTAGTATGTTCCTTGCCGTTATCGTCAACGGAAGTTCTGATCTGTCCAAAGAGTT
TTACAAAATCTCCCTGCTTAAAGTCTTTTGGAATATCACCCTTTTCTCCGTATGCGGAGCAATTATGATATACCT
TATTGCCCTCATCATCTTTAGATGCCACAGAGAAGTTTACTACTTGAAAGGCTTCGCCGTTCTTATTTTCCCTTT
CAACTACATCGATCTTTCCTACAATATTTCCAACGATATTGACAAGATTATCTTTCTCCTCTTGAACATAAGGAA
GATTTTTAATCTGCCCCTGTTCTCTTAAATCTTCAATCATGTAATCAAATTCCTCATGCAGTAAATTAACGGTGT
CATTGTCCATATAAGCCTCATAGAGCTTATCCAAAGCACTTTCATCGTTGATACCTTTTTCCATGCTGATAACCG
CCTTAACAAAGTCCTTGTGATTATCACTTACCATATCTTCTATCTGATTTCTCATTGTTTTGTAATCCATGTTTT
ATCTCCTTTCATGGTAAAAGGGAGGTTTCCCTCCCTTATCGTACATATTCCTGTTCTTTTGCAGTTTGTTTTTCT
TCTGTTTTACTTTCTTCCTGATAGGCTCTTATCTGTCCTAAGATAGATGGTTTATCACCCGTTTTCTGTGTGGCT
TCCTCTTTGGTATGCAAATTATCTTCAACATCATAGGTTGACTCAAAATAATCGCTGTCCTTAAAGTCATTGTCG
TATCTGTCAGCAATTCCATCATTATCAAGGTCTTTAGAAAGTGGATCATAGAAGTTACCTTCATCATCAATGTCC
AGTCCCAGAGCTGCCTTTAAGTCCTCGGAATCCACATAGACTAAATCTTCAAAAGAGGATAGCTCTATTTCATTT
TTCATATTCCTTAGAGCTTCATTTTCTCCTTTGTTTTCATAATCAAAACTCTCCGTTTTAATCGGTATATCTTCA
ATATACTGTGTCCATGTCTTATCCTCTAAGTTAAGCTCATATTGGATGCTGTGTCTTTCGTCAGGTGTATTGGTA
TAGGCGATGCCGATGTGCTTTAGATCAGGATATAGAGTATCAAACTCATCATAACTATGATTTTCTTCATACTCT
CTGTTGCAGAAGTCAATGATGGCTCGTTTTACATCTTCTACAAGGGGATTATCATTTCTCTTTTCTTCCACTTCT
CCCATATCAAGGAGCTTATTCAGTTCTGCCAAGCGAAGCACATTATCCTTTAGTTCATCAGCTTTTTCAAAAGGC
TTTTGTAATTCCTCTTTGGCATTTTCAAGCTGTTCTTTTGTGCCGAGGAGCTTTTCTTCAAGCCTCTTTAATTTC
TCAGGCATTTTCTCAAGAGCATTATCAAACCTTGTAATATTACCGTCCGCACTCGTTCCAAGCTCTCCTGAATGC
TTTGCAGCACCGTTTAAGCTGAAGTTATGCTCATTGGTGAAGAAGTTGTAGCTTACCTCTAAGTCCATGTTTCTA
TACTTACCGATAACCTTGCTTTCATTGATTTTCACTTTAGAAATAGCTTCAAGCAACTTTTCTCCGGCTAACTTT
TTATCTAATATCTTCTCACCACCGATAGTAATAGAAGTAAACTTTTCCTCTCCTTCAGCTTTTGCTTCTACATCT
TTAATATCTTTCTTTACAGCCTCAATGAGTTTTTCTGTCCTTGCAATTTCTTCCGGATAGTTTTTAGCAACCTTA
TCCTCTAATCTGTAACGGTTGGACTTATAGTTTGCTTCAAGCATTTTAAGTTTTGTAACCTCATTATCCAAATCC
ATCTTTTCCTTAATCTTCGGATCACCTGTAGCAAGGGCTTTAATCTCTGCATAGTTTAAGCTGCTTTCGTCCACA
TCTTCCGCCACTCTGACAGGTGTTTTACTTGTCATAATCTGAGAAATGAACTTCTGTTTATTCTCGATGGTCTGC
CAAAGGTACGCATCAAAGGTATTCTCCGTTACATATCTGTATATGCTGACTTCTTTGTTTTCATTTCCCTGTCTT
ACAATTCTGCCCGCACGCTGCTCAAGGTCGGCAGGACGCCATGGGACATCTAAATCATGAAGTGCAATCAGCTTG
TTTTGCACATTCGTTCCGGCTCCCATTTTCTGTGTAGAACCCATCAAAATACGAATTTCGCCTTTTCTTACCTTT
GCAAAGAGTTCATCCTTTTGCTTATCGGAATTTGCTTCATGGATAAAGGCTATTTCTTCTTTCGGTATTCCCATT
GCCACAAGTTTTTCTCTAATATCATCGTAAATATTAAACGCTCCATCTCCTTTTGGAGTTGACATATCGGAGAAA
AGAAGCTGTGTGGACTTATTTTCTTTTGTCTTATCCCAAATGGAAAAGACATTTTTCACGCACACATTGACCTTG
CTGTCAGGATTATCCGGAAGCAGTGGATTGATTAAACGCTGATCTAAGGCGAGTTTCTTGCCGTCATTAGTAATC
TTCAGCATATTATCTTCGTCAGGCTCTACTACCCTGTTTCTCACATCATCAGCTCTTTCCGATAAGCTCTTTAGG
ATTACCTTTTGCTCCTCACTTGGCAAAGTTTTAATAACTTCATAGTGTGCTTCAGGTGTTGGAAGATTTAGCATA
TCTGCTGTCTGAATATCCGCAACTTCTTTAAACATAGACATTAGTTCAGGAAGGTTATAGAACTTGGAAAATCTT
GTCTTTACTCTATACCCTGTTCCCTCCGGAGACAATTCAAAAGCTGACTGCGTTTCACCAAAAGTAGAAGCCCAG
CTATCAAAATGCTCCAAATTATTCTTTTTAAGGCTTTCATACTGAAGATAACGCTGCATAGTATAAAGCTCTGTC
ATCGAGTTACTGACAGGCGTTCCTGTGGCAAAGACAATTCCTTTTCCACCTGTCATTTCATCCATGTATCTGCAC
TTCATAAACATATCGGAGGACTTAAAGGCTTCAGACTGTCCAATACCTGCTACATTCCTCATTTTTGTGTAAAGG
TATAGATTTTTGTAATTATGTGCCTCGTCAATAAAGAGCTTATCTACACCTAATTCTTCAAAGGTAATGACATCA
TCCTTCTTAAAATCATCGTTTAACTTTTCAAGCCTTGTTTCAAGTTTCTTCTTTGTCTTTTCAAGCTGTTTTACC
GTAAAGTTTTGGTTTCTGTCATGCTTATATTCTTCTACATAGTTTATAATTTCATCAATCTGATCCTGAATATGC
TTTTCCTGATATTCCTTACTCATCGGAATTTTTTCAAACTGCGTATGCCCGATTACAACGGCATCATACTCTCCT
GTGGCAATTCTTCCGATAAATCTTTTTCTATTTTTCGGCTCAAAATCTTTCTTATCTGCAACCATAATGTTAGCT
GACGGATATAGCTGCATAAACTCACGACCGATTTGACCTGTTAAGTGATTGGGGACAACAAACAAGGACTTACTG
CACATTCCAAGCCTTTTACTTTCCATCGCAGACGCCACCATTTCAAAGGTCTTACCACTACCTACCACATGGGCA
AGCAAGGTATTTCCTCCATAAAGGCTTCTTGCTATGGCATTTCTTTGATGAGATCTTAAATCAATTTCTGTGGTC
ATTCCCTCAAGGGAGAGCTTGCTGCCGTCATATTCTCTGTTACGGATAGAGTTAAAACGCTCGTTATATATCTTT
ACAAGACGGTTTCTTCTTTCCTGATCGTTAAATATCCAGTTCTTAAATTCTTCTTTTAGAAGCTCCTGTTTCTGC
CCTGCAAGAAGCGTTTCCTTTTTATTTAATACAGAAGTTTTCGAGCCGTCCGGATTTACAATCTGATCAAATACC
TTTGTTTCTTTTAAATTTAGAGCATCTTCAATCAGCTTATAGGCATTTACCCTTGAGGTGCCGTAAGTCATCTCT
GCAAGGTCATTTCCTCTATCCCTGCTCTTTCCTTCTACATTCCATTCGCTTGTCAGATTTGAAAATTTAACCTTA
ATATCCCATTTGGCATATCCCGGAGTTTTAAGCGTTTCAAAGATGAATTTTTCAATATCCTTAATCGGTATCCAA
GTAGCTCCGAGTCTTACATTGATTTCACTTGCTTCAAGCTCTTTTGGAAGAACTTTTATTAGCTCTGCCTTTTGG
TATTCCAAACGGTTCATCTCATAGCTTATCAGCTCTTTTTCTTTGCCATCTTCCGCATAGCCAAGATGAGGCAAC
TCTCTTTCCGTTTGTCTTAGCTTTGCAAGATAGCTGTCTACAATAGCAATCTTATCTCTGATATTTCCGCTTAGG

TACTCATTCTTTGTTACATAGCCGTACTTATATGAATTACTGCCGTTTGCACAGGCAAAAGGTAAATCTCCATCT
TCAAGGTTAAAAGACAATGGTCTATAAAAGTTTTGTTCTTCTCTGATGTTTAGATAGATTTCCCCTCTAAGTTCT
TCTATCAAAGTCGGTCTGTCTTTTCCTGTAAGGCTTCCCATATACTCAAAATCCACATATCCTTTTTCTGATACC
GATAAAACAAGAGCTTCAAGGGAAGTGTCTACATGGTCGATAACCTTCGCCTTTGTGATTGTTCGCTTGGAGAAA
ATATCTCCCTTTGCTTTGAAGTTTTCTTCTTCATCAAGGATTTCAATGGAAGAAACAAGCGGGAAGTTGCTATCC
TCTTTTAAGGCTCTGGTATTAGAAAGGTTGTTGACATATCCATGCTTCTTAGAAAAGCTGTCATAAATTTCATTT
AGTTTTTCCTGAGCTTTCTTTACTTCATCATCTGAAAAATCTTCTTTTCTGTTTGTAAATAACGTCCTTTAAGGCA
GCATTTAACTCAAGATAGTCCTTAATCTTTTCCTTGTTTTTATCCGTTACTTCTTTCTTTACAAACAGGGAGTTT
TCTCTGTAATAAACTTCATCATCAATAATGGTGTAGGAGAAATTCTTGACATCGTCCGTTGCGGGTATTGTGCTG
ATTTCATCATCAAGTAGCTCTATTTCTTCATAATTAGCATTTTTAGCGATTTCTTCGCTTGCTTTTGTCAGTAGC
TCCTTTAAGTCTGTGTTTTCTTTTGGCAGGCAAGCTATTGTATTCCCAAATCTTCCGGATATTTCTTCCATAGAA
CCAAGCACCTGTTCAGGATGATCAACAAAGTATTTGTTATATAAAAGCCCGTTTTCATCTTCAGCAAGGTGAATC
CAATCCTCATCCCTTTCTCTGATACTGTCCCTTTTCTTTAGGAAGATAATATCTGAGGTTACTTCTGTTCCTGCT
ACGCCCTTAAAGGTATCGTTCGGAAGTCTTATCGCTCCTAAAAACTCTGCTCTTGCTGCAAGGTAGCGTCTTACA
CTTTCGTCCTTTTTATCCATCGTTCCGCTTGATGTAATAAAGGCAATAATACCTCCGTTTCTTACTTTATCAATG
GACTTGGCAAAGAAATAATCATGGATAAGGAAGTTATTTTTGTTATACTCCCTGTCATTTACCTTATATTCTCCA
AAGGGTACATTGCCGATAATCGCATCAAAGAAGTTATTTGAAAAGGAAGTTTCTTCAAGCCCCTTAATCTGTATA
TCACTTTCAGGGTATAAGAGTTTTCCAATTCGACCGCTTACTGAGTCAAGCTCTACACCATAAAACTTAGACTTG
TTCATTTCATCAGGGATATTGCCTATAAAGTTCCCTATTCCCATACTTGGCTCTAAAATGTTTCCCTGTTTAAAT
CCCATATCCAAAAGTGTCTTATACACTCCGTCAATCACTGTTTTCGGTGTGTAAAAGCTCGTTAAAGTAGATTCT
CTTGCAGCTTCATATTCTGCCTGCGATAAGTTCTCTTTTAGAAAGCTCCTTACTTCTTTCCACTGTCCGCCTTTT
TCTTCATCGAATACATCAGCAAGTCCTCCCCAGCCAACATACCTTGCTAAAACTTTCTGAGCGGTAATATCTAAA
TCTCTCTCACCTCTTTCAATTCGATTTAACATCGAGATTGCTTCAAGGTTATTGTTTAATCTCTCACTTGGAGAT
AACTTGTCAGGTAGCGTTTCTTCTTTAATTTTGAAGTTATGAGCTTCTGCTTTCTTTATTTCTACTTCTTCAGTA
GTCTGCTCTGGATGTTTATATGTTTGATTTTGAAATACCCTCTCAAGGTCGCCTTCCAGACGATAAGGAATCACA
TCGGAGCCTGTTATCATACCGCCAAGATATTCTGTATTGTCCTTAACCGTTACCGTCTTTAGGTTATTTCCCATA
TCATCAAAGCGTGTGATGGTATAGTCTTTATCCTTGTATTTGACTTCATCTCCGACAATAAAGTTCGGTCTTTCA
AGGCTTATCTGCTTCATAAGATCCTCATTATCTGTAAAGGCTACAATCGGAATTTGATGATTGCCTTGCCTTACA
GGATCAAGCCACAAGTCATTTTTTCCTGTGATTTCATTTTTAGAAATTTCTCTGACTTTGTATTCTTCATGATGA
AAGTAGACGGTATCACCTGCTTTAACTGCAAAATCCTCTAAAGGGATTTCCTCTTTTTTATTCAGTTCTACTTCT
TCTCTTTCTTTGAGGTAATCAAATAAGGTAGCTTGAACTGTGTCAGCCCTTCTAACTTCTTCTGTTTCTTCAGGT
GCTTCAAGTTCTTCTTCTACAAAGCTAAGTTCTCCATTAAAGACAGACTGAAGCTCCTGCTCATCCATCTGCTTT
TTAAGTTCTGTATCTTTCAGCTCTCTAAAGGTCTTTGGAAAATCTTCTAAAATAAGCCTTTGGGCATTTAATTCC
TTTAGTTCTTCAAGATTAAAATATCCCCATTCAGGTTCAATCCCAAGTACCAGTCCAAAGGCATCGCCACTTTCC
CTGTCATATTCCGTCATATACCAAGTCCAATTAGAACGGAAAGGAATGATATATGCAGCATGAACCTCTTTATCC
GCTAATGCTACATCCTCCTGTGCGTAAAGCTCCGGCACTCTTTCAAGCATTTCATCGGTCATTAAGTTTTCAGGA
GCATCTTTGGAATAATATTGTGGTTCTTCAATCTTTGACTTGTTTTCTGTTTCCTTTTCTTCAAGATAAAGATTT
TTAGAAAACAGCTCGTCGATATGCTTTGCAACACTTGTCCAAGTAAGAAACACATCATTACAATCATTTTTCTGT
AATTTAAGTCCCTTAGCATCGTGCCATTCATCACTTCCCATTGCTCCGGAAACAGCATGGGAATGTCCTCCAATT
CCATATTCGTCTTTTAAGAAATTCGCTTTTTCCTGAAGCGTATGATTTTCTTTAAAAAACTTGGTGATTCGTTCT
TTTCCTCTATCAACGCCACTTCCTCTTGAAAGACTTTCAAGGACTTCATCTTCTGTAATAAAGGACTTTACCTTC
GGAAGTTCTGTCAAATTGGTGCTATATTCCTTTCGTGGCAGCTCAAGTTCCTGTAATTTCTGATAAAGGCTATCT
ACCTTGTGATAGTGAAACCTTAGTACATTCCTATTTCTTTATATCCTGCTATAAATCTGTCATATTCTCTGATT
GTTTCTTTCAGATACTCAGGATTTTTTAATGCTTCAGATAATCTTTTTGTTTCTTCCGAAAAGCCCCCACCTCTT
TCAAAAAGTTCAAAATATCCTTGCTCTTTTCCTTCTTCACTTAAATCATGAGATAGATACCATAGAGATTCTGAA
ATCCGATCTCTTTCATAATCAAGAGCTTCAAAAAGCTCTACATTTGTAGCAAATTCCCCACTATTAAGAAGTTCA
TTTATCCTCTTTGCAGCATCATTCCAGTTTAAGACCTGCGTATCATCTTCTCTTGCAGAAGTTCCATAGGCTAAA
TGAATGCCTTTATCCGAATACCAGGAAGATACTTCTCTTTCATCAATGTAAAACCCGTTTCCGCCTTTAAAGGTA
TCTTTGAGGTATTCTCCCAATTCTTCTACTGTTTTTTCTTTGGAAAACTCAGCAATAACAGGAAGTCTGCCGCCA
TCGTGATTTCCTCCGTTAATAAGGACGGTATCTATATCATTCTCAGTTAGCGGAATCGTAAGGCGTGTCTGTCCA
TAGGAGTTTTCTGGTAAAGAAAAAGAAGCCTTTTCAGCTTCTCTTATCTCTACATCAATATTTTCTCTTAACCTT
CTACTATTTCCTTGATCGTCATCTCTTTCAAAGCTGAAATCATTGCTTTGTACTGTGGATTGCTCTCTCCCTCTA
TCTTCCAAGCTGCCATCAGCTTCGGCTTCTCTCTTCTCATAAACTCTATCGCCTGTTTCTGAATGTCCATCAGGT
GTCCTGTCAACTTCTTCTCCTTGTATAGATCTACTAACATCTCGAAGTTGCTCTGCTCCTCGCTCTGTGAAAGAA
ACTCCAGCCTCATCACTGCGTAGGTCGGATTTGGATACTTCCTTATGAAGTCCGTCTCTTCCTCCAAGCTGTTTA
GCGTATTCTCCCTGATTTTCTCTATTATCTCGTCTGTACTCTCCATTTCGGAGAACTCGCCCGTTTTCATTTCTT
CCCTGATCATCTCGTCGAAGTACATATTCTTCTACCTCCTCCAATTCTTCTTTAATCTTATTATATCCCGCTTCT
TTTCCTCTTAAAACTTCTTTTTGCAGCTCAAGTTCTTTGCTTTTTTGAATGGTCGCATCAATAATCTTTCCGCTA
ATATCCGATACGCTTTCACCAAGACTCATTAGAGATATGCTGTCAAGTCTTTGAAAATTCTCTCTTAAAAGCTCA
TAATCTATCGAATAATCTAACTTAAATCTTGATGCTACCGCATAGCTTACCGAGTCCCTTACAAACTTTGTAAAG
GATATTCTATCCTCATCCGCTATTCTAAGTTCATTCATTAAGGTATCTATTTTTTCATCACCGTAGAGTCTGCTT

AAAGAAAAGATGTTTTCAAGTGTGCTTTTACTTTCCTCATAGCCCTCGCTTTTTATCATTTCCTTTAACACATCT
TGATGATTTTCTTTGTCAAACTTCCAAAGATTTACTTCGTTGACATCTCTGTTTCTTGAAACTGTCTGACCTATA
TCAAAAATATAGTCCACTTTCTTGAATGTGCCATAGTCCTCTAAAATGGGGATACCTTTCTGTCCTCGCATTACT
GTTCTATTAAAACGTTCCCTCCAGTAGTCAAACTTGGCACAAGCTATTGCTTCAGGATTTTTATCATAGATACTT
AATTGACTTCTAAAGTCATATCTTTGATTGTTTCCGACAACCTTTAAGAGCTTCAGATACTCTGCTTCACTATGA
AGCACATCTTGTTTTATAAGCTCCAAAATGTTATGAAAATCATTTATTCGCATTTTTACCTCCTTCGTTTTTCCA
AACAAAAAAGGCGGTTAGATTTTACTCTAATCACCTTTTACGCATTATATTATTAAATTTTATTCGTTTGTTTCT
TCCGCAATATCTACATATTCTTTTAATTTTCGTTGCAACAAATTCTTATCTATCAGTTTTAATGTATACTCCGAT
ACCATTGTAGGCGAAAGACTTCTACTTAATGCAAATTCTACTACCTCATCATCTTTTGAAGCACAAAGGATTACT
CCTACGCTTGGATTTTCAGTTTGTTTCTTTACTTCTCTATCCAGTGCTTCTAAATACAGGTTCATTTTTCCGATA
TATTCCGGCTTAAACTCTCCAATTTTTAATTCAAATGCAACTAAACAAGATAGTCCCCTATGATAGAACAACAAA
TCTACATAGTAATCATGATTGCCAACCTGTACCCTATACTCATTACCTATAAAGGAAAAATCTTTCCCAATCTCT
AAAATAAAGTTCTTCAGATTCTGGAGAATTGACTTCTGAAATTCTCTTTCATTTCCAATCTTGGGAGCATCAAGA
AATTCTAAAACATAACTGTCCATAAACAAATTATGCGTCTCTTGTTTTGCCCTTTGTATAGCAGGCAAATCGTTA
CTATTTGAAAGCATATAACGTTCATAATAGCCACTGTCCATCTGCCTTACCAACTCTCGGTGAGTTAGATTCTCC
TTAATTGCTAAGTTAATGTAGAATTCTCTTTCTTCTTTACTTTTAGATCCTGACATTATTTTCAAATGATTAGAC
CAGCTCAATTGTGTCAACAACGTTGACACTTTTTCATCATCCTTATATAGCTCATAGAATTGCCTCATTCTATAA
AGCCCCCTACGGGTAAATCCTTTTAAATCAGGATAATTTTCAGAGAAAAAATCGGCAACATTTTCCACAAAATTT
GACCCATAACCTGCTTCTTTACTTTTCTCACTTATATACTTTCCCACTTCCTGATACATCAAAATCAATTCTTCA
TTGACTTTTCTATAAGCACGATCCTTTGCACTTTCGACAATCTTAACAATATCATCAAACTGATGATTATCTTTT
CCAATATCTGTATTCTGCAATACCATCTTATCCTTATTCATTGTTTCACCTACCTCTAAAAACGTGCTGTTATTT
TTTAATGTTTTCTTCAATAAAAGCCTTAGAATTTTGAATTTGCACTTTATCTTTATTAACTACAAAAGTAACATA
ATCTCCATTTTCTAAATTTAAAAGTTCCCTTATCCTTTTCGGAATAGTAACCTGTCCTTTTGCCATAACTTTTGC
AGTATCCATAAATATATCTTCCATATTTACTTCCTTTCGCCCTACTTTTCCTACAAAATTATAGCACTTTTGGAG
TATTTTTTCCACTCTGAATTAGTTATCTCTTACTTACTATATGTCAGAACAACCTCTTTTATTTCATTTTCAATT
GCTTCTAAAAATTCTTCCTTTGAAGATTTCCCTTTGGCAATATCCGCTAATTCCATTTCCCACTTTGCAGTTGTT
TCCGCTGACTTAAAGGTATCGGATACTATTGTTACAAGGCTGATCCCCTTATGCGTGGCAATCAAATTTTTCTTA
TCTCTTTCGACAAAACCCTTATAGATTAAGTTTTCAATAATTCCTGCCCTTGTTGCAGGTGTTCCAAGTCCCTTT
CTTTCCACTTCTACACCTTTTTCTAAGGCTTCATTTCCTGCAATCTCCATAGACTTTAGAAGCGTATCTTCAGTG
AAGTGTTTCGGTGGTTGAGTAAATTTTTCTTTAATCTCTTTATTTTCAATACTTAAAACATCACCGATACTTACA
TCAGGAAGCTCTATGAACTCATTCTTCTTGGACTTGTATTCTTTAAGGTATTTGCTAAAGCCCTCGTCTCTAATT
ACCCTTCCTGAACTTGTAAATTCAAATCCGTCAAATTCAGCTACAATCTTTGTTGTGTTTTCGACTAATGGATAG
CCTACACTTGCGTGAAGCTTATTAGAAATAAGCCTATATACCTTCGCTTCACTATCAGGGATACTCGATAAATCT
TCACTTAACGAGCTTACTGTCGGAATAATAGCGTGATGATCCGTAACATTTTTTGAGTTAAATACCGTCTTGATA
CGCTCTGTATCAAAATCATTTTTTCCTAAAATGTTATTGACCGTACTTACAATCATATCTTCCGTTAAGCATCTG
CTGTCTGTTCTTGGATAGGTAATTAGTTTCTTTTCATACAGGCTTTGAGCATAATCAAGCGTCTGCTTTGCTGAA
TATCCAAAATATTTATTACACTCTCTTTGAAGTGTTGTTAGATCAAAGGGTAAATCCGGCTTTGTAATCTTTTCT
TTTTGAATGACATCAGTTATTTCAATATTATCGCCTATTAAATTAATAAGCTGCTCTGCGGTTATTTCATCACCA
ATTTTGTCTGTCGATAATGTAAAGCCGTTCATAGAAAGCTCTACGGTGTAGTATTTTTCTTTCTTAAAATTATTT
ATTTCTTCATCTCTTTTTACAATCATGTAAAGCGTTGGGGTTTGCACTCTCCCGACACTGTAATTTTGCTTATAC
AGGCAAGAATATAGCCTACTGATATTCATTCCGACAAGCCAGTCTGCAATAGCTCTTGCCTGTGCCGATTCAAAG
AGCTTATCGTAATCTTTGCCATTCTTTAGATTGGAAAATCCCTCTTTGATAGCACTATCTTCCATTGACGAAATC
CAAAGACGCTGCATTTTCTTTTTGCATTTAGCTTCGTTATATACCAGTCGAAAAATACTTTCTCCTTCTCTTCCC
GCATCGCACGCATTGATAACTGTATCAATCTCCTTATCATTCATCAGTTTTTTAAGGATATTAAACTGCTTTTTT
GTAGCCTTTGCCACTTCAAATTTGTACTGCTTCGGAATAATCGGCAAATCCGATATATTCCACTTAGCGTATTTT
TCATCATAAGCGTCCGGATTTGCCATCTGAATTAGATGGCCTACGCACCAAGATACCTTATATCCATTTCCCTCA
TAGTATCCATCTTTCTTTTTTGTAGCTCCTATTACCTTTGCAATTGATATAGCTACACTCGGTTTTTCTGCTATC
ACAAGTATATGTTCCATGTATATTTTTCCTCCTGCTTTCATTGTAAAAGGGCGAAAGATTTTACTCTCCCGCCCT
GCGTTTGCACCCATAACAATATATTTTAAGATGCATTTATTCTTCATCATAAGCTTCTGTTTCTTCTGCTTCTCC
TATTTCTTGCTCACTTTCTGCTTCCGAAAAGAAATCATCATCTTCTTCCTCTAATGATTCAAGCTCCTTATCCTC
TTTCTTTTTTACGACCTTAAAGTAATAACCTGCTCCTAATGCTCCGCCTATAACAAGAAGCAGAATAATATATGT
TCCTAAATTACTCTTTTCTTCTTTTTTCTCAGGCTTTACTTCTTCCTTAGTTGGCTCCTCTTTGCTAATCTCCTG
CTTCGGAGCTTCTTTCTTTTCAACCATATTTAGAAGATCATCTTCAGATACTTCCGTTAAAAGCATTACATTTTC
TTGTGTCTCATCGTGGTTGATGATGAGATGGAAGGTCTTACCGTTTTTCGTTTGAAAGGTAATAAACTGTCTTGC
ATCAGCAGAATACAGATCTGTTTCTTTGTTATCACCGCTATCCCCGTGATGAATTGGATAGTCCTTATTCGCATT
GTCCTTATTTTCCGTAACGGTTCCTCTTGCCTTTGACGGAGCAGAAGCTACTCCCTTATTGGTATTTACAGTCTT
ACTTGTCCCGTCCATAGATGAATCCTGATTGTTATTGGCAGGAGCTTTCGGTGTCAGCTTATTAGGATAGCGAAT
ATCTTTCTCTTTCGTTTCAGAATTACTTGTATCTTTTACAGTATTTTCTGAACTCGCCTGTGTAGTTGTGCTTTT
TCCTGTATTGTTTACAGGTGTTTGATTTGAAATGCCTGAGTTTTTCTTAATTCCGGAAATAGGACTGTTCAGTGC
AGGCGGATTAGATATTACGGGAGCCTTGTTTGCTTCATTTGCCTTTTCCTCAAGCTCTTTAATCTTTTTATTCAG
CTTATCCATTTCCTTCTTCATTTCTTCTGATAAGTCCCTATTTTCCTTATCTTTTTTCATTTTTTCTTTCAAGCT

TTCAATCTGGGCTTCAAGTTCTTTGATTTTTTCTTTCTGATTATCGCTTAATTTGTCTTTATCCTTCATTTCGCT
ATTTAACTTATCAAGTTTTTCCTGAAGCTCCTTAGCATCTTTTTCCATTTTGAAAATATCATTTTTAGAAAGCTC
TGTCTGCGTTGATTTATCTTCCGTCTTTGGAGTTTTCGTCTGCGAGCTTTCATCCTGTTTCGGCTTTTCTTCTTC
TGTCTGAGTCTCTTTATCTTTAGCCTCAGTCTTTGCTACCTTTCGGATAATTTCATCATTTCCATCGCCTTTTAC
TTCATAAAATAGAAACTCATCAATAAATTTCATATTATCAGGGAGCATCGGCAGATCACCGCTGTCAAGAAGCTG
TCCTTTCTCAGCCTTTATCTTTTCTTCCTTATAAACTTTTTCATCTTCAAAGATATACTTTACATTTACTTCCAC
TTGTGTTTGGACAGCTTTATCGCTTGTAGGTGCTTCCGTTAAGCTTTGCTCATGAGCATAAACAACTGTCCAAAG
ACCCAAAAGGCAGCTTATACTTACAACGACTGCAAGTGCTGCTGTCCAAAACTTCTTATTCTTTTTCCAACTGGT
TTTCATTTGACTTCTCCTTTTTCATTTCTACTCTTTGTCTGTTTACCTTTGCCATCAAATCACTGATTGTAATGT
TGTTCTTTCTGCAAATGGCAATGATTTCTTCGTTTTCAAGTTCTTCTTCACGAATAAATAAAGGCTCCAATTCTT
CATCAATCAAAGCCTTTTTATCCTTCAACTTCTTTATTTTGTTTTTCACTACCGTTAATTCTCGTTTCACATTGT
TACCTCCTATTTCTTTACGTTTGGTGGGAATCCAAATCCTACGGGATGATGCTTGCAGAAAGACGCAATCCAATT
GTCTAAAGTGGTTACTCTTATTCGTCCAATATTATCCATAACTTTACCATCTCCAATATAAATTCCTACATGACC
ATAGGTAAGTCCTGCTGTGCTTCCACTACTACTGCTTTCCACCGCTACAAGCATTCCCACCTTGAGTTTTGACCT
GTCTGATGTAAAAGTATAGTTTCTGTACATATCGCAGGCATTTCCGCCAATATATCCAAGACCTGCATTTTGATA
GACCTGTGAAACCCACATGGCACACCAGCCTGCACCCGGAGATGGCGTGATGTATGCAGCATTAACAATCTTCTT
TTGAACTTCACTTGATGCTTCATACTCAACTCCACCACCTACGCCACCATTTGCACTGATAAGGTCAGAATTTCC
AAAGGCTTCTCCCATATTTCCTTGTGCAAGGAATAAGGCTTCATAGTGCTTTAAGTTATCGGGATAATCAGCAAA
GACCTTTCGGATAATGCTGTCCATCTCTTTTTTATGCAGCGTTACAATGAGCTTTTTATACTCATAAGGTTCTTC
ATGACTTTCGGTATATTCGTTGCCATCTTCATCGGTATAGGTGTCTGTAACTGTCCTGTATCTGATTTCAACTTC
TTCCCTATATTCAAGGTCATACATGGACTCAAACAATTCTTTTAATATGGATTCTACTTCCGATACACTCTTTAC
CTCTCCACATCTTGATGTAATATAGGATAAAAGCTCATGGACATTATGACCAATATACTCTGTATTATTTAAGAT
GTACTCGTCATAGCCGGGATAGTTATTTTTGACATGATCAACCTCATTTTGAAGCTCACTCTCCATAGCAGAAAA
GCTCTGATTGATTTCATTTAGGACATTTGGCTTTGACAAATAGCTTGTTGTAAGAATAGAACTTGTGGAGTTCAT
AAAGCCTGTCATTCCCGTTCCTGCAAAGTTGATAAAGAAAGTTCCCAAAATAATAAGACCTATGAAAATAATCAT
AAGTCCCTTTGCTTTTCTTAATATAAATCTCTTTGAGCCTTTCAACGAGCCAATGAGGTTTTCTTTAATCCGATC
TCTGAGCCTTGACTTATTCTCACGACGAATGGCTGCCTTTACCTGATTTTTCTTTTGAAATCTTTTATAGGCATT
TGCTCTTTTATATTCATTCGTCTTTTTCAGGTTCTCTTTGGCATCACGAAATTCAAGCTTCGATTTTCGCTTCCT
GATTTTATAATCCTTATTTGTAAGATCGTATCCTTTCTTCGCCTTTTTCTTATCAGAATATTTCTTTATGCCATG
AATGAGTTTCGAGCTTGCGTCCGCTGTCTTTTCTCCTGCCTCTACCCCTTTATTTTCATCACTTCCATGAGAAAG
ATAATCCCTTACGGTTTCACTTCCTTTGGCAAGTCCTGAAAGGGCAGATACCTTTACCATATCTTTCTTTAGCTT
TTTCCTCTGCTCTTTAGAAAGGCTACTATAAACTTTTTCGCTTGCAGCATCTTTTCCAGTCTTTTTACCTTCAGA
TTTATTTCCCTTTGACACATCTTCTTTTTTTCTTGTATAAAGGCTTTCGGTGTAGTTTTTCCTCTTATACTTTCT
CTTTTTAGCCTCATGAGTTATAGAGGAACTTCCAAAAAACTCATCTTTTTTATGGAGATTATCATCCACATCATA
GGTCGATTCAAAGTAGTCGCTGTCCCTAAAGTCATTATCGTATCTGTCTATAATCCCGTCATTATCAAGGTCTTT
TCCTAAAGGATCATAGATTTTTCCATCCTTTACCTCCGTAATATAATCCGATCTTCCTGTTTCACTTACAGCAGC
TATACTATCCTTATCAGAAGCTCTATATCTTGCATTTCTCTTGGATGTTCCATAAGCCTTTTCATTATCAGAACT
TACCTTGTTTATTTTCTTATGAACCTTGTCCTGAAATCTGTCTTTGTCATGGACGATTTTTCCTCTGTAATCATC
GTTGTGCTTTAGCTTACTTTCTTCAGATGTAGTGAATGTTTCACTATGGATCATTTCTCTTTCAAGACTTGCCTT
ATGCCTTTCCCTAAAATCCTTTTTCAGCTTTTTCCCCATAGGCTACCTCACTTCTTCAGGCTTGGTAGTCATCTT
CTGATAGAGAATTGTATCTTTCGGGAACTTATCAAGGAAAGGAACAATGGTATTTCCAAAGAACAGTAATCCCTC
GCCCTCATTTGAGTTGGTAACATATCTAAGCTGAGGAAGTGAGATTTTAAGTTTTCTTGCTAAAATTTCTCTGTC
TCCTGACGCTTGATTTAGCATTAAGACAAAGTCGGTATTATCAAAGATATTTTCAATTTCCTTACTCATAAGTAG
GTCTTTGACATTTTGCGTAATACCCGTAGGGATACCTCCCCACTTACGAAATCTTTTCCAAATCTCTACCGAATA
CGATGCTGTCTGTTCATCTTTTAGCAGCAAGTGGAACTCGTCGATATAGTACCTTGTAGCCTTGCTTCCTCTATT
TTGAGATACTTTATTCCACACCTGATCCTGTATAACAAGCATTCCTATTTTCTTTAGCTGACTTCCAAGCTCTTT
AATATCAAAGCACAGGAGTTTCTTATTTAAGTCCACATTTGACCTGTGATTAAAAACATTAAGGGAACCTGATAC
ATAGATTTCCATCTCCGTTGCCAGCTTTTTACCGACCTTTTCTTCCTGTCCTTTTAACATATCGTATAGGTCTTG
CAGTATTGGCATATTGTCAGGAGTTGGATTATCAAAATACTTTTCGTAAATCTTAGGTAAGCACCTGTCTATAAC
GGACTTTTCTTCTGCCGTAAGACCACTGCCGCCTACTACAAGCTCCAGCATACTCATAATGAAGTTTGCTTTGTC
TTTCAGTGGCGCATCCCCATCGCCATAGTTCATATTTATATCAAGGGGATTCAGGTAATCTTTGGACTTACTGCT
GACTTTAATGACTTCTCCATTAAATTGTCTTACAAGGTTTCCATACTCTCCTTCTGGATCGCAGATAATCACATC
ATCGTCCGTTACTAAAATTGCATTTGCCATTTCTCTTTTAGCACTAAAGGATTTACCACTTCCAGGAGTTCCGAG
GATTAGACCGTTTGGATTTTTGAGTTTTTTCCTATCTGCCATAATCAGGTTATGGCTTAAGGCATTTAGTCCGTA
ATAAAGACTGTTACTTGAATTGATAAAAAGCTCCTCTGTGGTAAAGGGCATAAAAACTGCCGTAGATGATGAGGT
TAGTCCTCTATCTATCTCAATCTTGTTTACGCCAAGAGGTAGCACGCTGATAAGTCCTTGTTCTTGCGTATGGTC
AAGTCTTTTCAGCTTGCAGTTATGCTTATTTGCAATGGAACTTATCTGAGAGATTGTGTTATCAAGCTTTTGCAC
CGTTTTTGCAAAGTTCATAAAGACGATACTTACCACAAACATTCGTTCATCTCTTGTCTGCAAATCTTTTAAGAG
ACTCTTTACATCTTCTCCATAGGTGATTAAGTCCGATGGAAGAATGTCCATATCATAACCGCTTCTTACCGCTTT
CTTATTTTCCTCAATTTTCATCTTGTCAATATCGGTATTTTTTCTCTTTACCATTTTAATAGCTTCCGATTGGTC
GATTGCCTTAATATGAAAGGAAATATTGATGTTATCATCAATATCCAAAAACTCGGCAAGCATACGGTCTGAAAG

CTCGCTGGCAAGAATTTGAAAATGGCTGACTGCCCCGATAAATTTTCCAAACTTAAAGTACCTTGAAGGCGTGAA
GTTAAATTCATCAGGTACAATGTATGTTTTAGTGCTTTCCCTTTTCCTCAAATTTTTGTATGAAAACTCAAAGGT
CTTATTGGGATTTAATATATCGTGAAGAATCTTGAGTCTTTCTTCCCCACTTAAACTTTCCGCACGAACTCCCAT
ACTCTTTAGATTAGATAATATATCTATCTCCAGTCTTTCAAGTTTTGATGTTGCCTGCTCTAAATTATCCGCTTC
TACTGTAAAGGTTACATACTTTGATTTTTTCAGTCCATTATTTCCCTTTACAATCTGACTTTTAAGCATCTCTCT
AAACTCAAGACGTATATCGTCAAAACCGTCCTTTTTATCTGGTATCTGAATTGCCGACTTCATTTCTTCGTTTCG
TCCAAGTTGATTGATATATGAAAACTCAATACTGATACTTGGATCAAAGGAGTTTAGAAAATTTGCAAACTGATT
AAAAATAAGATCCCTATCTTCATCTAAAGCAAGTTGGTAGTTAATATCCTGAAAGGCTATGCTTTTACTAAAGTG
CTTTTCGTCAAGCTGACATATACCACTTTTTAAGAGTCTATGATAGGGAATGGTATCTTCGACAGTATATCTTTT
TGGTTCTTTTCTAAAAATAAGGTCAAGTAGCCCGCCTTTATCTTTTTTAGATTTTCCCTTATTTTGCTTTAAGTC
CTGTCTTTGACTTCTTAACATCTTTTTGTTTTGTTCTAACTTTACCTGCTGAATCTTTCTTTTGTCTTTCAAGGT
AAACCTCCTTTCTCACTCTTTTTTGTGGTTGATAAAACTTATGAAGGTAAATATATTTAAAATATTTCTCAAATG
TCAGTCCGTCCTTTTCAAAAAGAGTGATGAAAAATATAGGGAGTGTGGACACAATGAGAAATATGACGGCTATAT
CATTTGGAACAACCTTTCGCATAAATAAATAGACTGGTAGTCCTACCAGTCCTGCGAGTGTGAAACCTATCATCT
GCCTTTTGGTTAAGTTAAAAGCTACCTTTGTTTTTACCTTCTTTAGGTCTTTTGGGATTGGTACATACGCCATAA
CTTACCTCCCATCTTCTTTTTCTTTTGAAAGCTCCGCCATGTGTTCATATACAGAGCCGATTTCTTCCTGAATGC
TTTCTATCTGTTTATCTGTATTTTTGTTATATCTTTCCTGCAAAATACAAAAGTCATTATGGCAGCGACCGATGT
CTTTTGTTCTTTCTTCCAAGTCATTAATCTTGCTAAAAAGTTTGATTCTATCAATCACCGCTATAACACCTGCTC
CGATTACTACTGATGTAAATACTGTTTTTCTTTTGATCATCCTATCCTTCCTTTCTGTTAGTGTGCATTTAATAC
GCTTTTGGCCAGAGTTCCGCTCTTTAGCATCATTAACCCCAGCAAAACCGCATATCCAAGTATCGTCATGGTACT
TGTGTGTATATCTGTTATCTGTATTGTCTTAACTAATACTGCGTATATTCCAAGACAAACCATTAAAAAGAGTCC
TTGTAGCCCAAGTGCAAATAGTCCTTTGATATAGTTTGTTCCAATTTGTCCCCACTCTTTGTTTCCCATTGTGGC
AAATGGAATGGCTGAAACCGATGAGTAAACATAAATCTCAAACATTCTTCCGTAAACCACAAGCATGATTACAAT
GGAAATTACCTCTATGGCAACCTTTATGAGTGAGGTTTCAAAGAGAATCATGACAAGCTCTCCAAGCCCCTTTTC
TTTTAAGGTATCCATCATTGCCACTATCTGATCTCCGGAAACGGTGGCAGAGGTATTTATCACCCCTGCCGCCTT
ATTTACCATATGTTGTGCCACATCAAAGACTGCCATTGAAAACTCAAAAGCATGAGATACTAACCATACAGCAAT
CCACATCTTGATGATGTACTTAAAAAATTCAAAGGTATCTGTATCGTGCATATTGTTCTTTTGCATTACCATATT
GATAAGCTCAATACAAAGGACTGCTGTGATGATTAGTCCCGCTATGGGAATAATGACGGAATCGTTAATGCTTTT
GATAAAGGCAAATACATCTCCGTTCCACCCCATGGGAGTTTTTCCTACATCTGTTGCAACTGCACCAACTTTATC
GTTGATGTCAAGAAACATGGACTCTAAGTTTGCTTGGATACCGCCCAGTAGAAGTTCCTTAAAAAATTCTTCTAT
CTTGTCGAATATTCCAAACATCTAAGCTCTCCTTTTCTTCTTACTTGAGTACATTGGCAAGTAGCGGAATCAGCT
TAAGTCCGATAAGGACAATACCGCCTCCTGCCATAAGCTGCTTAATACCTTGAGATTTTGCTGATGTGTGATAAA
GAAGTAGTAGAAGTGTAAAAAATTTTGCCGTTCCTATCCGGCGTTTGCGCATTGCGCCGGATAGGTCGGGCGGTC
ATTCGGGCAGGTCTACCTTGCCAACGAAAGAGTAATAAATATCAATGTCCTGTCTGCGTGTGCCGTTCTCGTCAT
AGCTGCACTCATGCACAACGATTTTCTCCACAAACTCACGCAAAAGGGTAGGGGTAAGTTCTTCAAAGCTGGTGT
ACTTGCGGACAACATTCATAAACTTTTCTGCGTTTACCGTGGCTTCCTGCGCTTTGGAAAGCTCTGCCCGGATAG
CGGCGGCTCTCTCTTTCAGTTCTTTCTGCTCGGCTTCATAGTCTGCCGACAGCTCCGTGAAACGCTCGTCTGAAA
TGCGCCCGGTCACGCTGTCCTCATACAGCCGCTTGAAGATAGCGGATAACTCGCTGATACGCTTCTCGGCGGCTT
CCAGCTCCTTTTTCCTTGCGGCGTTCCTGCGCTTGCCCCCGTCCTCGTTCTGCTCGATCAGCAGCTTCATAAACC
GGGCTTCGTGCTTCGCTGCATAGCTGGTGACTTTCCGCAGATTGTCGGTCACTCCGGCGGTCAACAGGTCGGTGC
GGATAAAGTGCGCCGTACAGTCACGGGTACGCTTCTTGTAGCTCCCGCAGATATAACAATCCTGCTTGCGGGTGG
CGTTCTGGTAACGCTGCTGGTAAAGGACGCTGCCGCAGTCGGCACAAAAGAGTATGCCGGAGAATAAGCCCACTT
CATCATAGCGGTTCGGGCGTTTGCGCTGCTTGCGTAACTCCTGCACACGCTCCCACATCTGGGTGTCAATGATAG
GCTCATGGTGGTTCTCGAAAATCGCCTGTTTCTCAATGGGGTTCTCTACGCTGTGCTTGGTCTTGTAGGACGGCT
TCTCCGTCTTGAAGTTTACCAGACAGCCCGTGTACTCCCTGTTTTCAAGGATATGTACCACGGTATTGGTCGCCC
ACTTGCACTCATAGCCGGGGTGGTAGCGGCGGGTGCTTCCCGTCCGACGGTATTCCAGCGTTCCCGGCGTGGGGA
TCTCCTGCTCTGTGAGCATACGGGCTATCTTGGTCGGCCCGTTCCCGGCAAGGCACAAGCTGTAAATCTGCCGCA
CAACAGGGGCGGCTTCCCCGTCAATGATAAAATTTCGTCCTCGTCCATAAAGTAGCCGTATACGGGTTTGCTCG
TGACGGGCTTCCCGCTCATACCCTTAGACCGTTTTACTGCTTTGATTTTCTTGCTCGTATCTCTCACCAGCCATT
CGTTAAAAATGTTCCGCAGAGGGGCAAAATCATTGTCGCCCTGTGCGCTGTCCACTCCGTCATTGATAGCGATAA
AGCGGACGCCTTTCTGTGGGAAAATCATTTCCGTATACATTCCCACTTGCAGATAGTTTCGCCCTAACCGTGACA
TATCCTTGACGATAACGGTGCCGACTTTTCCGGCTTCAATGTCCGCAAGCATGGCTTGAAAACCGGGTCTTTGGA
AGTTCGCCCCAGAATAACCGTCATCTGTGTACCATTGCACATTGGAAAAGCCGTTCTGCTTCGCATAGGTTTCAA
GAATACGCTTCTGGTTTGAAATAGAATTGCTCTCACCTTGCAGCTCGTCCTCGTGGGATAATCTCGGATAAAGGG
CGGTAATAAGGTTTCGGGTGGTCTGTCTTAACATAGTGTCCTCCATTTCCGACAGCCAGCCCCACTATTCCGTAT
GACTATCATACCACACGCCGGGGCTGGCTGTACAGTCCTTTCTGCTTCTTTACGTCCCGTCAAATTGCCGATTTT
TGTGTAGCAGCTTCCGCTTCCAGCACTTTCAGCATTTTATCGGCGGCGGTGGCGGTGGTGTCCTGCTTGAAATAG
CCGGAAACGACAAGGACAGAATTACCTATGCGGATTTCCGTCACGCAATCCGGGCGGCGGGTGCTGCGGTCATTC
TTTGGGGTGTTGGTCATAGGCGGCTCTCCTTTCTGTTCATCAGCTTTTTCAGTTGTCCCATTTTCTCCTGCGCCG
TGGCTTTTCGGAAGTTGCTCCCGGTAAAGCGGACAGGAGAACACATTTCAATCAGACGGTCATAAATGCGGGCGT
GGGCGGTGTCCTCCGGGTGCTGCAAGTCCTCCAGCGTGAGATTAGTCGTGACGATCAGCGGCCTGCCGCTGCGGT

AACGGCTGTCAATCACGTTGTAGACCTGCTCTAAGCCGTATTCCGTTCCCCGTTCCATTCCAAAATCGTCAAGGA
TAAGCAGCGGGAAGCTGCAAAGTCGGGAGATATATTCATTCCTGCCCTCAAAGCTGGCGGCAAGGTCACCCAATA
TCAATGCAAAGTTTGTCATGCACACGGGGATTTCACGCTCCATAAGGGCATTTGCGATACAGCCCGCAAAATAGC
TCTTGCCTGTGCCAACGCCGCCCCATAGCAGATAGCCAATGTTGCGCTCTTTCATGGTTTCCCAGTTCTCCACAT
AGAAATGGGCGTGTTCCATTTGCGGGCACTTGCCGTTGTCGTTCTCAAACGTCCAGCCCTGCATAGCCGGGTCTG
TAAAGCCCCGCCGCTTCAACCGCTCGACAGTTTCAAGGTGACTGCGCTGTTTCTCTGCGGCTTCCCGTTCCTCAC
GCTCTGCCCGCTGGCAGTCACATTCTGCCGGGTGGCGGTCACGCCCCAGCCATGCGGCGGTTTCTTTGGGGAAAT
AGCCCTCTTTGGGCTTGTGGCACTTCCCGCAGTATAAAAGCCCGTCCTCGCCGGTGTAGTCCTCCGGCTCCGGCG
TGGTGTCGGTCATATTCAAAATCATTTCATCAAATCCATTCGTCATAAGCTCTCGCCCTCCTTACAGGTATAATC
GGGTATGCCCTTTTTCGGGGCTTTCTTGGCTGCGTCCTCCTGCGCCCACTTGAAAATCGTGGCTGCATGGCTCTT
GTATTTCCTCCCGCTGGAAGCGATATGGCAGGATAGGCGGTCAATGTAATACTCCCACTTGCCGGGAAGCTCTGT
TTTCAGCCCGTCAAGCTCCGTATCGGAAAGAATGACATTGTGGTATCTGCCATAGGCGGCGGGGGCGGGGTGTCC
CGTTTCTAACTCTCTCTCTTTTTCTATTTCTATATCTATCTCTTTCTCTATCTCTATCTCTGGTGGACAAATGTC
CGCTCGATATGGTGGACATTTGTCCGCCCCTGTCTGCGGCAGGGCTTTTTGTTCCTGCAAAGCCAGCCGCGCCCT
GCGCTTGCGCTCCCCCTCGGTAGAGGACTGGCCGATTAAAAGCTCAATGTTGCTCATGTAGAGTGCGCCGCTTGG
CAAAGGCTCCACAAGCCCCAGCTTCATAAAGATTTTCAAAGCTCTCTCTACGGTACCTACCTGCTGGCGGGTAAT
GGTCGCAATCATCTGGGCGGTGTAGGGGATATTCTCGTCAAGCTGCAATTTCCCGCCGTTTTTCAGCGATTTCAA
GTACAGCTTCAAGAGAATGTTGGAATAGATAACGCCGTCCTGCATACTTTCCAGCAGAACGATTGCATCATCGTC
AAAATAGCTCTCTTTCAGCTTGAGGTAGTAATATTTGCGGTTATCTGCCATAGGCTGCGTCCTCCTTTTTCCAGC
GTTTGGAATAATAGCGAGGGCATAGTATGATAACCGCCCGGAAGCTCTGTTTGCAGTCACGGGTGCAGCCCCGGC
ATAGGTCGTTGTAAGTGATACGGTTGCGGTGGTTGAGGAAGAAAGACCATTCCAGCCGCCGCTTCTTGCTCATTC
TCGGCAATGGTAAGCTCCTTTCTGCCGTTCCTATCGGTGTAGCGGGATAGGGGGCATTTTCTGTATGTTCTCGGT
ATCATTTTCGGGCTTTTTCTGCCCTGTAAGCCCCGTTTGGAAGTCGGGGAGTATTGCGGTAAGGGTTCATATCAT
ACCTGTATTTTTGTAGGGTTTCGGTATCATTTCGGGGCGGTCTTTAACGCTCCTGTTCACGCTTTTTCTGCTGGC
TCGGTGCGCCCCTTAAAATCTGGTCGATATTGCGTCTGACTGTCTGAAGCTCCTGCGCCCGCTGGCGTTTCTCCC
GGTAGTCGTTATATCCGGCGTTCTTCTGGACGGTAAGCTGCTCAATCTCCCTTTGCAGGGCTTTGCTGGCTGGCA
GCTTGGTTATTCCATGCTCCCGGAAATAACGGGCGGCTGTGTCCGCTATGATAAAATCGCTTTCGTGCTGTTCCC
GGTAGGCTCTCCGGGCTTTCTCCGATTTCTGCGCTTTCAGACCGTCACGGGCGGGCTTCGTGCCGATATAGCCCA
AAAGGTTGCGCTGCAATTCCTTTTTCTCCCGGATAGCGGCTTCCAGCCCTTTCAGTCCGGCAAGGCTCTCCTGCG
TGGCGGTGTTGGCTGCGGAAATGGCAGCGTCCAGCTCGTCCGGGGAAGAAAAGCCATACTGCTGGTAGAGCATGA
GGGTAGCCGACATCTGTTTGAGGTTGTGCATGGTCGCCCACTTCTCATAGCCCCGTCCTTTCCCCTCGGCTCGCT
TGGCGGCTATGTCTACCATGCGCTGTACAGCGTCATTGTTCGGGGCGTTTTTCGCTTCTTTTTTCGTCCGTAAGC
GGTCTTTGATACTGCCGGGGTATTCCGCTATGGCTGCGGGTTTTTCGGCGGCTCTGGCGGCGTTCTGCTCCAAAA
CGGAGAGGACAGCAGCCCGGTCAAAATCGTCCCCCAGCTTCCGGGCGGTAATTGGCTTCGTCCTGTCCGGCGTGA
GGTAACTTAGCCGCCCCCGGCTCTCCTTGACGGTCACACCATGCCGGAGAAGCAGGGCGGCAAACTCATCAAAGC
CGGAAGCAGCGGCAAGGGCTTCCCGGATAGTCCGGCGCAGCTTCGCCTTATCCGTTTCAAACTTGGTCTGCCGGG
GCGCGATACCGTCCGCAGCAATAGGGGCGTTGGCTCTGTCAAGGGCAGCCTGTCCTTTCTTCTGCGCCCAATACT
CACGCTCGGTAATGCGTTCTTTGCTGCCGTTCAAAAGGTCTATCTGGTAAAGCCCCTCGCTGTGGCACATCTCCA
TGACTTCGGCTTTCAGATAGTTCATAGCTGCGTCCGTACAGCGGTGCTTGCAGCCTGCTTTCCTGTCCGCTGGCC
TGTCCATGTGGGGCAGCATGGGAACCTCCGCAATCCGCAGACTGTTAATGACGATATGCACATGGATATTTTCGG
TGTGGCTGTGTCCGTCCGGGTGGGTGCAGACAAGGGCCTGGTGTCCGGGGAAATGCTCGGCGCAGAACTTCTCGC
CCAGCTCCTGCGCCCGATCAACGGTCAAGCCGTTGTCCGGGCCGTCCCGTGGGTCAAAGCTGATGATGTAGTGGT
GGCTCTTTACGTCCTCCCGTTTCTGGTTCTTGCCGTAGCGGAGATTGGAGCGCATACAGGCAACGGCAAAATCCT
CCCCGCCACAATTCAGAGAGGATATGCGGTAGTCTACCCTCGGTATGAGCCGCCCGTCTGCGTCAAGGGTGGGCT
TCATGGTAAACTCGTCATGCTCGAATGTGAGGTATTTTTCAGCGTCCCCGTAGTTGGCATTTTTAGAGCTGATAT
GTTTGAGTATCGCCAACGGCTTCACCTACTTTCTGCAAGACTTCAAACTTCAAGGCGGCAAGGTCGGCGGCGGCT
CCCCGCACTTCCTTTTCCAGCCCCCGGTAGGGGCTTCCGTATTCGTTCAGGCTCCGGGCAATCTGGTTTAAGTTG
CCGCCGATTTTCCCGTACTCTGCCGTGAGCTTGGAGAGGGCGGCAAGCAGCCCGTCATTGACCGGGGAAACGGTG
ACAATGGGGCGTATGGTCGCTTTCCGTATGGCTTGCCGGATAAACTCGGACTGGCTGATTTCATAAGCCGCCAGC
CGCCCGGTGAAGTCGGCGTATTCTTCATCGGTCATGCGGGTCTTTACCACATGACGGCGGTGGGGCGTATTGTAT
TTCTTTCGCATGGTCTGTGACCTCCTTTCTCGCCCGACAGGGCGCATAGCAGGGTTTGGGGAAGGCACTCCCCAA
CAAGATTCCCGCAGGGGCAAAAATAAGCGGAGAGCGAATTTTGGCACCTCGGTAGAATCTTGCTCTGAAAAACTC
CGGCGTTCCCCGGCTCCCGTCCTTTCCGCTAACAAAACGTTTCAAAAGGGCTTTGCTACCCGCTATTCCGGCTTA
TCTTGAAAATTTTCCTTTCACTACCTACAACACCACGCAAAGCAAAATGGACGGAGATTTTTAGAATTTCCCCTC
TATTCCCTACAACACCACGCAAGCCGGAATAGGCGGACAATGGCAGTATTTTTTTCTTTGATACCCTCTACGGAT
AAGTAAGGGATTTTGCCAACTGCGGCGGCTATTTTCCCTTTTGTTTTTTCATACTGGGGATTTTCAAAAATGCCA
AACAGGAACGAAAAAAAGCAGCAAATCTGTTGAATAGATTTACTGCTTTCTGGTTGCCGGCGAAGCGGCGGTTAT
TCAGTTGTAGGCGGTAGGGCTATCTCCCAAAGCGGAACTTGAAAATAGCTGTGAGAAGCGTCTGGGTGATGTAGT
CCTCTGTATCTTGGTCTACCCGTCCATTCACACGGGCGGCACATTGTATGCGGCGGCGGTAATACTGCAATACAG
TTCCCACCGCTTCCGGCTCCCCGCTGGCGGCTTGGACGATTGTTTCATAGGGGAGAAGCCTATTATTTCTCATAT
GCCATTCCCTCCATTTCCGCTTGGAGCTGCCGTAGGGCTTTTCGGATATGGTAGCCCGCTGTGCTGCGGCTGCGC

CCGTACTGTCTGCCAATTTCGTGCTGTGGAATACGCTTGAAAAAGGACAGGTAAATCATTTCCCGCTCCCGTTCG
TCCAGCCGTGACAGGGCTTCCGCAAGTAAACCGCTGCTGAAAATGACCGTATCGCCGCAAAGGGTAAGTATGTAT
TCCTCGTCCGGCTCCGGGGCTTGGAAATATTCATCTGTCGTGCCTAAAGGGTAGTGCTTTTCGTCTGTGAGGTAT
TCAAGGGATATTTCTCTTTTGTGCTTCCTGCTCCGTGTCCTCGCTGCGTTGATCGTTGCATTTCGGATAACGACT
TTGCAAAAGGCATGGAAAGTGTACTCGATATGTTCCCGATATTCTTCTGTACAGGTCATGGAAAATCCCCCTTTC
CTCCCAAAAGGGCTGTGGCTGGTTAGTAGTTGCTTTTTATGATAGTAAGGGGCGGCAGCACTTTAGGCTGTCGCT
CCTTGACTGCCTAACTGCAAAACCGGGCGGGGCTGTCAACGGCGGGCGAAGCCCGTTCATCTTGACCGTTGACTG
GCTCGGCCGGGTTTGCTATTTATCCGGCAAACTTGAATTTATTTAAGCTATCACGTTTTACCTTTTTAAGCCTT
ACTATCATTACCATAGGAATTTCTTTATTGTTTTTGAAACATTCTTCATAAAATCCATCAATGACAAATCCAGCT
CTGAAACAAAGGTTAAAAATATCTTGTATGGAACGATGATAATAAATCTGCTCCTCCGGCTGCCCTTCGATCGCT
ATATCATAGTAACTGTGCGGTGTCATATATTTTTCAGTCAATGTGATAAAACAAGGGTGCTGCGTTGCAAAGACA
AAAATCCCGTTTTCCTCCAATAGTTCATAAACAGCCATAAAAAGCGGTTCAATATCCGTAATATCCATAATTGCC
ATATTAGAAACTGCTTTCGTAAAGGCTCGATTTCTTTTTAATCCTAATAAGCTTTCTCTATTGGTCGCATCCGCT
ACGCAAAACTCAATTTGTTTTGCATATTGTGATCGCCGTCTTTTAGCCAATTCTATCATTTTTTTGCTGTAATCA
AAAGCGACGACCGAAGCGCCTCTCTGTGCAAGATACGAAGAATAATTTCCATTGCCGCACGCAATATCCAAAATG
TAATCCGAAGGATCAGGAGAGAGAAGTTCCGTTACTTTGGGACGTACTACCTCTCTGTGAAATTCATTGGATTCG
TCACCCATTGCATCATCCCAAAATTGTGCGTTTTTCTCCCAGATCTTTTTGCTTTCCTCTGTTCCCATGTTCTCT
CCCACTCCCAAAATTTGCCTTTTTGCTTCCATTAAATCTTCCTTACTATATTCCATTGTTACCCTCCATAACTTC
TGATTGTTGCCGTCTTGACTATTATGTATCTTTTCTTTCCAAAATGGTATAGGCTTTTATTTTTATAATGCTGCA
AAACTTTTCTTCTGTAAGGTATTCAAAGGATATTTCCCTTTGCCGCCGCTATTGGAAAGCCAAAAGCAGCGGCTT
TTTTACGCGATATGACCGAAAAGACTAGAAAGCAGGATATGTCATCATTTGTCATTTCTTCTCAATGTAATAACT
GCATGGCATATCAAGCCAAATACAAGAGAAAAACAGCCACCGCCAAATAGTGATGCTCCCCACCCTGCACCCGAC
ATTGTCATAAAACCGCCAACAAAGAAAATACCAATGCCAAGAAATATCCCGACACCATAAAAAAGTCCAATTGCC
ATATCATACTTATTAAATTGTCGTTTCTCTTTTTTTCACATGTTTCCATTTTTGTTATTACCTCCTTCGCAAAAT
CGTCCATGTGGACTCCAAAAAGAAAACAAATTTTTTTCAACGTATTTAAATCGGGTTCATTAACATCTCTCTCCC
AATTCGATAGCGCCTGCCGGGTAACATTCAATTTCCCAGCCAGTTCTTCCTGTGTCATTCCCGATTGTGTTCGCA
GATGACGTATTTGCTTTCCTGTTGTAATATCCGTCTGTTTCTGGTTCAAAATGGTTCCTCCTCTCTGATGCTGAT
TTTATCAATGATATTTCGCAATAGCCAGCAATGAACGTTTGCATTGCGCAAGATGTTACATTATCTTCTGAAACA
TATGCCGGATCTTGTCTAAACGGCTGTTCGGGCGGCGTGGCTGAAACACAGGCTCGCCGGAAGTTTCCTGATACC
CTTTTAATTCTGTAATGCAGACACTTCTTCCATTTGTATAAAAATTCAAATCATTTCTATATTCACCAATACAAC
GGGCAGGGATTTCCCCCTTAAAAATAACTTCATCTTTTTCAAGTCTGGTTGATTCAATGATTGCGCAATACTTTG
GTGCGTCATTATAAGCCCGTGAAAGATATTCCTGCGGTGCAAAAAGGGTAAAGGAAAGGTATGGTTCCAACAGTT
GTGTTCCTGCTTTTTTCAATGCCTGCTCCAACACGACAGGCGCAAGAAAACGAAAATCAGCGGGGGTGCTGACCG
GGCTGTAATAAACTCCATAATCAAAACAAATTTGGCAGTCTGTCACTCCCCAGCCATATAAGCCCTGCTCCATTC
CATAACGCACACCCTCCATGACGGCATTTTGAAAACTTTGGTTTAAATAGCCGAGAGATACCTCGCTTTTATATT
GTGTTCCGCTTCCAACAGGAAGCGGTGTTACAGTCAAACCAATAGATGCCCAAAACGGATTCGGCGGCACTTCAA
TATGAATCGTGTAGCTCGCTTTTTTTTGCGGTCTTTCCAGATAAATAACCGAAGGCTCTTTCATAGCCACGCCCA
CATGATATTTTTCTTCTAATAGCGAACAAATAACTTCTAACTGTACTTTTCCCAAAAAAGATAATATAATCTCAT
GTGTAACAGTATCAATGTCAAAATGCAAAAGAGGGTCTGTATCAGCAATCTCTGTGAGGGCATTTAACAGGGCTT
CCCTTTGCTCCGGCTTTTGCGGCTCTACCGTTGTCCGAAGTAATGGCATGGGATTATCAATCCGTGTTTTGTGAG
GCAGGAGTTTTTCATTTCCCAGAATGTCGTTCAGTTTCAAAGTATCATCAGCTAAAATAACAATTTCTCCCGGAC
AGGCATGGTCAACCGGGACGATTTCACCATTTGACGGAATACACATTTCTGTAATCTTTATTTTTTCCTTTTTTG
ACAGCAGCAGGGTATCCCGTAAATGGAGCGTCCCATGATACAGGCGTAAATAAGAAAGCCGTTTTTTCCGCTCTG
TATACTCAACCTTAAAAACATATCCACATAATTCAGACTGAATATCGTCTGTTTCTGTAATGAAAGTTTCTGTAA
TCGCTTCAATCAGTTTTTCTGTTCCTAAATTGTCTTTTGCACTCCCATGATAAACAGGAAACAAAGAGCAGCATC
TGGTTCTTTTGCACTTTTCATATTGTAATTCCTGTATATCCAAAGAATCCTCTGCAACATATCGTTCTAATAGTT
CATCGCTTCCGGAAATAATCATATCCCATTTGTCCAAATCAGAAATATCGGTCATGGTTATCTTTGGCGACAGGG
AAACCTCCTGCATGACAATCATATCACTGGTAAGTTTATCTTTAATGCTTTGGTAAACACGCCGCAGGTCGATCC
CATTTTGGTCTATCTTATTTATAAAGATAATTGTCGGAATGTCCATTTTCTGAAGCGCATGGAATAATATACGGG
TTTGTGCCTGTACGCCGTCTTTTGCCGAAATGACTAAAACAGCTCCGTCAAGGACAGATAAAGAGCGGTATGCTT
CGGTTAAAAAATCCATATGACCGGGAGTGTCCACGATATTGATTTTATAATCATTCCAGCAAAAAGAAGTAACCG
CTGTCTGAATGGTAATTCCGCGCTGCCGTTCCAAAATCATAGTGTCTGTTCTTGTAGTTCCTTTATCCACGTTTC
CTTGTTCCGCAATCGCTCCACTGGTGTACAGCAGGCTTTCCGTCAATGTTGTTTTTCCTGCGTCTACATGGGCGA
GAATTCCAATATTGATTATTTTCATGTGATTGTCCTCCTTTTACAGCCCCAAAAGGGCATAAAAATCCCCAGCAG
TAAAATACTTTTACCACTGGGGATTATAATTTGCGGACATACACATATACAGCATACACCTGTTTGTGATTGCTG
TTTTTCGGATATGTCAAAATTGATAAGGCAAAAGTATTTTTAAATTGGGTACAAAAAACCAAGCCCCCACAAAAG
GGACTATCATAATCCTTTGTTCCCACTATTTGATTATAGTTTTATTTAAGAATACCTTGCCGCATATTTTTTACT
CCTTTTTTGGAATGATGCTATTATATCACATTAGTTTTAGGAAAGAAAGTACCTAAAAGAAATTTTTCTTCCCCT
TATATGTAACAATCATACCGACTTTCTGGCGTTCAGAATGGTTTCTGCTGTCTGCTGTGGTGTTTGGTTGGAATT
GTCCAGCCAAAAGCCGATCCGTGGTGTTGTCTGCATAAATGTATCGTATAAGGTTTCAACCGTAAAGCCGGAATA
GCCTGTTTTCTCCCTGTATCGTTCCCTTCCTTTTATTGTTTCCACATCTGGACAAAGAACGACAACCTCAACAGG

GTATTTATGCGGCACAAAAAATATGTACATGTAACTAATTCAACACATTTATAATTTGAATAGGGACATTATAGC
ATTTACTAAATACAAATTCAATGTATACGGAAATAAAGATATAAGGAGTGGGAAGGATTCCGCCGTAGTCGGCAT
TGTAGGAAAATCCAAAAGTTTAGATTTTCCCACAATGCTTATCTTTTGGTCTTTGGTTCGGAATAGTGTAGTGCT
GGCGGTCTATCTCTTGTTTTCGGTTGCTTGCTTCCTTACCGTACATGAGCATTTGCACCCGGATTGTCATTCCCA
TAACCTTCCATCAGGTTGATAACACCCCATGCTCCAAGTCCTGCACCTACTGCCATTACCAAAATCTTTAATACA
TTAACTGCCTGTGTAAAAAATTCCATAATTTATTCCTCCTCGCTTTCTTCTTTCTTTTCAATCTTGTTATATGAC
TTCACTACAAAGTTTTTGTAAGTCTTTCCCTCTTTTTCACGCTTCTTAAAGTAACCAAAGATATGAATCAAATCG
CCTTTTTCAAAGTCCTTTGCTTTCTCCGATTTTTCTCCATAGGCGGCACAGTTGATATACTCCTTGCCTTTCCCG
TACTTTTTTACAAGCGTGAAGTTTGCAACCTCAACAGTTTCTCCCTCTTTGTCAAAATTTGAGAAAGTGGGCTCT
GCCAATAAATTGGCATTGATGTTAATCATTTCTTGCTTCATTAAAAATTTCTCCTTTTCTTTCAATAAAAAAGCG
ATTGGAGTTTTTTCTTTTCCAATCGCTAATGCTCATCCTATTTACTTTTTTCTTAGTGGGACTTCGTATTCTTAT
CATCTTACCTCCTGGCTTTGAGTAATAAAAAAGCAGCAAATCTATGTTCTTTAGACTTACTGCACTCTTGTAATA
ACTGTTTCCCTATTCAGCTTTGCTTTTCCTTTTCGCTTCATATAGCTTTCTATGTCAAACAAATTTTTCTTGTCA
TAGTCCTCAAGCAGCTTATAGTTCTTGTGTTTTGTAATATCGAATTTATCTGATAGAAAAGGTCTGACACCACGA
AGCTGAAAAATACATTTACCGCCGTCCATGACCGTAATTTCATCTTGGCTCATAAGCTCCTTACCTGTCTTTTGA
TAATTAAGTCCAAAACTCTTTTGATTACTCCTTGTTTCCGATGTGTTGTAAAGGTCAATGGTTTCTTTACCAAGC
GTTTCAGAAAGCTCTTTAAGTGTTGTTTTCTCCTTTCCACCAAGAAATAAAGTGCTATCACAGTTGCCCACGATT
GTGTCAGCATTATCTTTATAGATTGCCTTTAGCTGAGATTGTGCTTGAAGAATTATACTCGCTGATATTTCTCTG
GAACGGATTGTCGCTATCAGTTTTTCAAACTTTGGAATTAAGCCGATGTTTGCAAACTCGTCAAGTAGGCATCTT
ACATGAACAGGTAATCTTCCTCCATACACATCATCTGCCTTATCACATAACAAGTTAAATAGCTGAGAATACATA
ATAGATACCACAAAGTTAAAAGTATCATCTGTATCGGAGATGATAACAAAGAGTGCTGTCTTTCTATCTCCCAGT
GTATCAAGCTCAAGTTCATCTTCACTCATTAGTTCTCTTAGCTCTCTTATGTCAAAAGGTGCAAGTCTTGCTCCA
CAAGATATTAGAATTGACTTGGCAGTTTTTCCAGCAGCCAATTTGTACTTTTTATATTGCTTCACCGCAAAGTGT
GTCGGTTCTTTCTTTTCCAATGCTTCAAAGAGTCTATCAATCGGATTCATGTAGGTTTCATCATCTTCTCTTACC
TCTGAAGCGTCTATCATATCAAGTAGTGTTGCAAAGTTCTTTTCTTCTCTTGGAGCTTCATACCAGATATATCCG
ATAAGTGCTGTATAGTAGAGTTTTTCAGCTTTCACCCAAAAATCCTCACCTGCTTTTTCTCCCTCGCCCTTCGTG
TTTGCGATAATTGTCTGAACAAGCTTTAGAATATCTTTTTCAGAACGAATATATGCGAATGGATTGTACTTCATA
GACTTTTTGAAGTTTATGGTATTTAGAATTTTAATCTCATAGCCATTATCTTCAAGCATCTTGCCACACTCAAGG
ACTATTGTTCCTTTAGGATCGGTTACACAATAGCTACTGTGCATTTGCATTAAGTTCGGTTTTACATAAAATCTT
GTCTTTCCGGAGCCTGAACCACCTATAACCAAAACATTTTTATTTCTTGCATACTTTGGATTAGCTGGTCTGCCG
TTCATAGTTAATCGTTCTGTTTGTGTAAGTAAGATATTGTTCTGGAACTTTTCATCCATATACGGTTCTATATCT
TTTCTCGTTCCCCATCTTGCTGAACCATACTCTTTTCCCTGTCTGAACTTTTTTGCATTTTTGCCTTTGCTGTAG
ACAATGAATTTAATTAAAGCTGCTACTCCTGCACCAATTAAAATATCCGTAGGATGAATACTTGGAAGAAAGCTC
ATGGTGTTAAGCTCTAATATCCCTTGAAAGATTTTGTCTATTACATCGCCACCAGTATAAGCTCGTACATGGTGA
GAGAAGATATTGCCAACATAGAAAAATGCAAGGTAGGGAATGTTCTGCTTTAGAAACTTTGCCTTATCCTGCACC
TTAAACAAGCCTTTGATGTCCTTTAGTATTTTCTCTATCATAGGCTCTGCTCCTTTTGCTTGTTCTTGATTTTAT
CTTTAGAAACAGAATTTTTAGCCATCTCCTTAAACTTCTCAATGTTTTTATGAATAGATTCTTTCCTTTCCGTTT
TCTTTTCTGATTCTGAAAGGGCGTGCTTAAATGCTTTGTCCATCACTTTCATATCTTTTGCTTGAAAGAACACTG
AGTATTTACCTGATTCTTTATCTTTCATTACCGAAAACTTAACTCCATATCTGTTCAGTTCTTTTTTCAGTTCCT
TCAATTCTGTTTCTTCTACTGGTATCTCTTCAAGCTGTCCCTTTTTAACCATATCTTTTAGTTTTACTTCATTCC
CTTTACTCCCAACGAGCTTTTCAAGTCCACCGATTTTTTCTGATTCTTTCAGTAACTTCCTTATCAAATCCAAAA
GTATCTTGCTTGTTACTTTGCCGGCTCTCACTTCCATGTTGAGCGTTTTTCTTGCAATTTCTTCATTGATCAAGT
CCTTTCACCTCCGTTCAGTAATCTATCTTTATTGCGAGTTAGCTTATCTCTTTGAATAAACTTTCTGAAATCCTC
GCCTACAACCATAACGGGAATACACATTTCAATAATTCTTGAATAGATTCTTTGATATTCAACACTGTCCTTGCA
GTTTTGAATTGTGTCGTAGGAAAGATTTGTTGTGAAAATTGTTGGTTTCTGTTTTAAGTACCTGCTATTGACAAT
GTTATATACCTGCTCTTTAGCATAGCTTGTATCCCTTTCAATTCCCAAGTCATCTAAAATAAGAACGGAAGTATT
TACAAGGGATTCAATATATGCGTTTTTATCAAAGTCAAAGCTGCTTTTTTGAAGTTCATTGATAATCTGTGCGAA
ATTTCTAATCTTAACACTTATTTGATACTGTTCAATAAGGCTATTTGCAATGGAGCAGGCAAGATAGGTCTTTCC
GCTTCCTACAGAGCCATAAAAGAGAAGTCCGATATTTTCTTTTTTCATTTTCTCATAATCTTCTACAAAATTCTT
TGCAATCATGAGGCTTTGATTTTCTTCTCCCTGATAATTTTCAAAGGTATATGACCACTCCCTCATGGAATTGAA
GCAGGTTCTTTTTAACCTTTCAATTTCCATCTGTTTTTCTCTTCGTTTCTTTTGTTGCTCTATTTCTCTATCGCA
TTTACAAGCTACCCTTAATATCATTTTATTCCCGAAAAATTCCATTACATCACCGTCTTTTCTTTCGTGGCAGAC
TTTACAATAGGCGTGTCCATCTTTGATATACTCTTTTTCAGAATCATAGGCAAATTCTACATCCTCTATCATTAT
TTTTCCCAATTCTCTTTTCATAAATGTTCTCCTTTGTTATATTCTTCCCATGTCGGGACATTTGATGTTTTCGGC
TGCTTGCTTTTGCCCTGATCTTTATAAAACCAAGAAAGGATCGTCGCTTTATGGTCTTTATAGATCTTTCCGGTA
CTCTTGATATATGCAGATAAGCGTTCAATGTAATTATCAAGCTGTGCATTTAGTTTGATTTGTAAATCAGAAATA
TCTTCATCAGTTAAAAATACATTTTGAAATGTTCCAAGTCCGTTTTCGCAAAAACTATATTCTCTCTTACTATAC
TTACTCTTATTATTCTCTATATAGTTAGAGTTAACATTTTTAACTTCCTGAAGTTCATTTTCTTTACTTCTGAGG
TCAACATCTTTTACTTCTGAAGTAAAGTTTTTTGACTTCTGAAAGTCATTTCCTTTTGCTTGAAATACACTCATA
AAGTCTTTTACATAAATGATATTAGGTTTTCCAAGTCCAAGCCTTACTCTTTCAATCAGTCCGATTCCTTTTTTA
CTGTCTAACTCATCCAATGTTTTTATGGCTGTTGGCTTTGAGATATTTCTTCTTCTCATAATTTCTTCGACGGTA

AAATAGATAAATACTCTGCCTTCCTTGTCTATCCATTTATTTTTAAAGGACATTCCTGTACGCTTTAGAAGCATA
GAGTATAGGATAATTGCTTCTGCCGATAACCCCTTAAATTCTTCTCCATCTACTAATATTTCAGGCACTTTCAAA
AAGTTAAATCGCTCTGCTTCTCTGTTATAGAAATAGTCAAAGTCCATCGCTTCACCTCCTCTCTTAAAATTTGCA
AAGAAAAAGACGATAGTTTTTCTATCGCCTTTGGTTACCATTTTGATGAAATTTTTTAAATTGATTTTATCTCTT
CATTGATTCCCTGAAAGAATGCTATAACGGTTGCTCCAATCATCGGTACGCCGTATGGACTAACTAAAAATCCGA
TAATCAATGCTTCGATTCCGATGGTAACTTCTTTTTGTAAGAAAGATGCTATTGCTCCAAATATGCAAAACATCA
TCAGCAAATATAGCAGTGCCGTTCCTATTCCGAGTAAGAATGTCAGAAATGCAGTGAGAATACTAAGCAGGAAGC
TGATTGGGAAAAAGATTATTTTTATTGTCCATCTCATAAAATATCTTCCCTTTCTATTCAATCATTTTGAAGTGT
CTCAAGGCATCCATGATAGCTTCTTCTTTTTCAGGTGTACACTGTGGAATAATTTGTTTCTCCTTTTTAGACTTG
TTGTAATGTTCTCGTAATTCTATTCCACATTTCCTTTTTATCTGTGCAATATATAATGTCGGAACTTTTAATTCA
AATTTATTCCAAACATATTCTTTGATTTGAGCATATGTTGCCTTACTTTCAGCACTTGTCAAATCAAGCTCATCC
AGCTCAATTTCAATATTTATATGCTTATCGACATCAAGTTTGGACAAAAGTGCTACCGTCTCAAC
